# 124th Annual Conference, Medical Library Association, Inc., Portland, OR, May 18-21, 2024 2024 Annual Business Meeting and Presidential Inaugural Address, June 5, 2024

**DOI:** 10.5195/jmla.2025.2356

**Published:** 2025-10-23

**Authors:** Kate E. Corcoran

**Affiliations:** 1 kate.corcoran@mlahq.org, Chief Operating Officer, Medical Library Association, Chicago, IL

## INTRODUCTION

The Medical Library Association (MLA) held its 124th annual conference May 18-121, 2024, in Portland, Oregon. The meeting was entitled “MLA '24: Stronger Together” and used a hybrid model with some events in person, and some virtually. The virtual conference was broken into segments, available using a variety of online platforms. Final numbers: total paid attendance for the conference was 1,035 attendees, with 821 attending in person and 214 attending virtually. Conference content, including the meeting program and various electronic presentations from special content symposia, plenary sessions, poster sessions, and program sessions can be accessed by conference registrants via the association's website, MLANET.

## PRECONFERENCE CONTINUING EDUCATION AND AI SUMMIT

May 18, 2024

### CE100 - Essential Searching Skills for Librarians on Systematic Review Teams

Instructors: Carrie Price, Health Professions Librarian, Albert S. Cook Library, Towson University, Towson, MD; David Farris, Research Services Librarian, Research Medical Library, University of Texas, MD Anderson Cancer Center–Houston; Rachael Lebo, Clinical Services Librarian, Wegner Health Sciences Library, University of South Dakota–Sioux Falls.

### CE200 - Master Data Cleaning and Wrangling with OpenRefine and Python

Instructor: Paige Scudder, Research and Instruction Librarian, Tufts Hirsh Health Sciences Library, Boston, MA.

### CE201 - Collections Budgeting: It's More than Spreadsheets

Instructor: Susan K. Kendall, AHIP, Head, Collections Strategies and Copyright Librarian, Michigan State University Libraries, East Lansing, MI.

### CE400 - The Joy of Project Management! Managing All Projects, Large and Small

Janet Crum, Director, Health Sciences Library, University of Arizona–Tucson; and Elisa Cortez, AHIP, Medical Education and Clinical Outreach Librarian, University of California–Riverside.

### AI Summit: “Why AI? Transforming the Health Information Profession”

Sponsored by Elsevier

Designed to be a high-level strategic summit for AI leaders and planners, the summit brought together health information professionals, representatives from vendors to health libraries, and National Library of Medicine staff to provide planners and leaders in the health information AI space with knowledge and insights needed to address the rise of artificial intelligence in all aspects of library operations.

## OPENING SESSION AND PRESIDENTIAL ADDRESS

May 19, 2024

Amy Blevins, 2023/2024 MLA President

Liz Kellermeyer, AHIP, MLA ‘24 Cochair

Emily Vardell, AHIP, MLA ‘24 Cochair

Amy Blevins: Good morning! Thank you for being here today on this beautiful morning in Portland, Oregon. Welcome to everybody who's attending both in person and virtually. My name is Amy Blevins. I am the MLA president for 2023 to 2024.

It's been a long road to get here to Portland. For those of you who remember, we were supposed to be here in 2020 but unfortunately, COVID19 had different plans for us, but we're finally here. How are you guys all feeling? Has anybody seen any Sasquatches around? Not yet.

As of this morning, we have 1,331 registrants and exhibitor representatives. We have 1,092 people in person, and I'm assuming they're all in this room right now. And then we have 239 virtual attendees. For those of you who are unable to attend in person, the National Program Committee and conference staff have planned yet another conference with a robust and interactive virtual component taking place at the same time that we are meeting in Portland. For those of you on the East Coast, it's a little later in the day than it is for us here on the West Coast, there are more live stream sessions, virtual presenters presenting live virtual sessions. And since none of us can be in two places at once yet, even though Gabe showed us yesterday you can have an AI version of yourself, there's going to be a lot of recordings and on demand sessions that you can attend later. We'll continue to experiment, so stay tuned for what may come next. We'll keep learning, adjusting, and moving forward, always with our professional needs and interest in mind.

And now it's my great pleasure to introduce Emily Vardell, AHIP, and Liz Kellermeyer, AHIP, your 2024 National Program Committee Cochairs. Please join me in giving them a warm welcome. [Applause]

Emily Vardell: Thank you, Amy. I am Emily Vardell.

Liz Kellermeyer: And I am Liz Kellermeyer.

Together: Welcome to Portland!

Emily Vardell: It is our honor and privilege these few days to conduct this conference on the traditional lands of many Indigenous Peoples, whose ancestors have stewarded this land for thousands of years. The Portland Indian Leaders Roundtable document “Leading with Tradition”[1] noted that “The Portland Metro area rests on traditional village sites of the Multnomah, Wasco, Cowlitz, Kathlamet, Clackamas, Bands of Chinook, Tualatin Kalapuya, Molalla, and many other tribes who made their homes along the Columbia River, creating communities and summer encampments to harvest and use the abundant natural resources of the area.”

We honor their history.

We also acknowledge the systemic policies of genocide, relocation, and assimilation that still impact many Indigenous and Native American families today. As guests on these lands, we respect the work of Indigenous leaders and families, and recognize their knowledge, creativity, and resilience.

We encourage you to discover more at the Naya Family Center website, nayapdx.org, or the Native Arts and Cultures Foundation, at nativeartsandcultures.org.

Liz Kellermeyer: This conference is governed by the MLA statement of appropriate conduct, which you'll find on pages six and seven of your official program. It applies to all of our activities, including conferences, meetings, workshops, online forums, social media, continuing education and all means of communication.

It applies to all of us at this conference. I encourage all of you to read it in the spirit of an open, inclusive and collaborative environment for diversity, equity, inclusion and belonging in professional practice; the leadership of information professionals; accessibility for all stakeholder groups; and the ethical standards that call us to conduct all professional relationships with courtesy and respect.

You will also have the facility in most sessions to chat and ask questions, but we've also been warned that [our meeting] software adheres to the scheduled times for sessions: virtual session moderators be warned!

Emily Vardell: As Amy mentioned, we are so delighted to finally return to Portland, for real! It's been 37 years since MLA was last here–that's a lot of water under this city's 12 bridges.

Liz Kellermeyer: In 1987, for some attendees and leaders, the MLA annual conference was 10 days long.

Emily Vardell: “The Internet”, which was really not yet public, was 5 years old and the web hadn't yet been invented.

And yet, medical librarians today would not find topics presented in 1987 unusual, such as “Teaching Information Retrieval Skills to Medical Students”.

Liz Kellermeyer: Or “The Contribution of Hospital Library Information Services to Quality Patient Care.”

Emily Vardell: But I think we can ALL agree that we have been blessed with better design sense in 2024! Many thanks to our MCI USA designer, Aubrey Dockery, for her brilliant and award-winning work for this conference.

Liz Kellermeyer: Please join me in giving a heartfelt welcome and thank you to our sponsors and exhibitors who have demonstrated their support for this conference, and for the value they see in information professionals.

They contribute to our organizations in so many ways and help us thrive

Exhibitors and sponsors also engage and collaborate with our members on multiple initiatives and provide education and knowledge sharing sessions that advance our profession.

We are grateful for their support and invite you to take time to personally thank them for their support throughout the meeting and afterwards.

I'd like to welcome to the stage, representing our gold-level sponsor, Michael Crossman, Vice President, Account Management at Elsevier, to share a few words with us.

Michael Crossman: I was going to introduce myself, but she took care of that for me. Elsevier, we really do appreciate the opportunity to be here with you and face to face in person to learn and hear, and learn from you over the course next couple of days The team is always ecstatic to come here to join this conference and to support our MLA, as we know that you're vital in the role of supporting your clinicians, your faculty and students, and providing the most evidence based information possible.

Throughout this week or the next couple of days our team is focused and really excited to engage with you, to learn more about your priorities, your successes, and your challenges. We're really just thankful for being here. So, thank you and have a great rest of your conference. Thank you. [Applause]

Emily Vardell: Thank you so much, Michael.

We are grateful to our Platinum-level sponsor Wolters Kluwer, who is sponsoring multiple areas of this conference.

Please welcome Rafael Sidi, Senior Vice President & General Manager of the Health Research segment of Wolters Kluwer, to the podium to share a few words with us.

Rafael Sidi: Thank you! Good morning, esteemed medical librarians. I'm Rafael Sidi. On behalf of Wolters Kluwer, it is my honor to welcome you all to MLA 2024. As the platinum sponsor for MLA, we are thrilled to be part of this significant event. This year, Wolters Kluwer is celebrating a milestone that is very close to our hearts: its 40th anniversary. Yes, we were here 37 years ago!

For four decades it has embodied the MLA mission, demonstrating that quality information and trusted information is essential to improve healthcare outcomes. As we develop the next generation Health Research Solutions, you, our dedicated medical librarians, are always at the forefront of our minds. Your unwavering dedication to your profession, your pursuit of excellence in medical research, your commitment to training the next generation of clinicians and your tireless efforts to advance patient care evidence-based practices inspire us daily. With your collaboration, we can ensure that Ovid continues to meet the needs of the ever-evolving medical information landscape. In line with this year's MLA theme, we truly are better together.

I invite you to visit our Wolters Kluwer booth. We would be delighted to meet you and hear your thoughts, and if some of you were here thirty-seven years ago, we would like to hear now your feedback on Ovid.[laughter] You are cordially invited to join our sunrise session tomorrow, where we will be discussing evidence based practice research and quality improvement, presenting our exciting new expert solution of its synthesis. Additionally, we have a lunch and learn session tomorrow afternoon, where we will take you on the journey of medical research, from boolean operations to Gen AI copilot, exploring the future possibilities of Ovid and how we can enrich research and discovery for our end users, on behalf of Ovid and Wolters Kluwer, I want to express our deepest gratitude for your ongoing support, for the invaluable work that you do. I wish you a very successful MLA, thank you and have a great day. [Applause]

Liz Kellermeyer: Thank you Rafael and thank you to the very supportive Wolters Kluwer team.

We are so energized by the enthusiasm of our members, presenters, speakers, MLA staff and technology partners who have been so committed in preparing this meeting. We salute the hard work and vision of the full NPC team for their creativity, building on the success of last year's hybrid conference to design yet another new and improved experience for all of us.

Our NPC team has been extraordinary in selecting programming to appeal to the diverse needs of all of our members. You can find all names on page 10 of your official program. Please join Emily and me in recognizing them and the dozens of content reviewers who reviewed the hundreds of submissions for this meeting. If you are on the NPC this year, please rise to be recognized or comment in the chat if you are [attending] virtual and if you are a content reviewer this year, please rise or comment in the chat to be recognized. Thank you! [Applause]

Emily Vardell: We also want to recognize the four special content committees who assembled impressive symposia and the AI Summit for this conference. We look forward to seeing all these sessions being held here in Portland over the next few days, with six symposia sessions being live streamed to virtual attendees and a summary of the AI summit being presented during our closing plenary. You can also find the names of these hard-working planners on page 10 of your official program. If you were part of planning one of the special symposia, if you could also stand to be recognized. [Applause]

Liz Kellermeyer: Those of us here in Portland have been amply prepared by our extraordinary Local Assistance Committee, led by LAC Cochairs Laura Zeigen, AHIP, and Sola Whitehead, AHIP. From blog posts to dining guide, from transportation advice to volunteer wrangling, the LAC has done an awesome job in sharing information about our host city. Laura and Sola, many thanks to both of you and your whole team.

Emily Vardell: We were delighted this year to inaugurate a new NPC subcommittee, the Virtual Assistance Committee, ably led by Chair Shalu Gillum, AHIP. We appreciate the virtual tips they've provided for our virtual attendees, and the upcoming virtual networking sessions later today and on Monday.

Please join us in thanking the whole group for their ideas and sessions. If you're attending virtually, add those emojis and shout outs to the chat! Thank you. [Applause]

Liz Kellermeyer: When you work so closely with the MCI staff conference team to organize this hybrid event, you get a real appreciation for the breadth and complexity of the task. This year, not only have they learned new software for this conference, they've been doing that at the same time as they are designing, building, and migrating MLANET to a new home.

Please join us in showing our appreciation for what they do. Thank you. [Applause]

Many of you have already been exploring the virtual portal and connected app. The portal and the app are the easiest ways to navigate and keep track of the large number of sessions at this conference.

For our virtual and in-person attendees alike: ALL sessions reflect the time zone your browser, tablet, or phone is currently in. Those of us who arrived from different time zones and who worked on our schedule ahead of time will notice that sessions now reflect Portland time. For our virtual attendees who haven't traveled, the times noted in the schedule will continue to be unchanged and accurate for your time zone.

Emily Vardell: The app will also let you play MLA '24 Quest – whether virtual or in-person–and keep track of everything onsite you want to see. The app or virtual portal also hosts session chat and Q&A, so you can ask questions throughout many sessions, whether virtual or in person. Look for icons at the bottom of the app, or on the right sidebar of the virtual portal.

There's no doubt: MLA '24 has a LOT of programming–I know I've seen comments on the [MLA '24 conference] padlet bemoaning the choices we all have to make–but I can't wait to get started later this morning!

We really thank our speakers and contributed content presenters for being so flexible and for going the extra mile to make this conference a success for both our virtual and in-person attendees. Your participation is essential to strengthening our community–and it's why we are “Stronger Together”.

And a final few words: make sure to NOT overdo, please take breaks, have snacks, or visit the Recharge room, and make sure to spend time with your “frolleagues”!

Liz Kellermeyer: It is now our great honor and pleasure to transition to the next and highly anticipated segment of today's program: Amy Blevins' Presidential Address.

Amy is the Associate Director for Public Services at the Ruth Lilly Medical Library, Indiana University School of Medicine, Indianapolis, IN. She is also a teaching faculty member in charge of two required medical school courses.

Amy's career has focused on teaching evidence-based medicine and critical appraisal as well as partnering on systematic reviews and meta-analyses. She has been involved in several continuing education initiatives as an instructor/facilitator and during the pandemic, used her skills in information retrieval and critical appraisal to support the WISE COVID-19 Expert Review of Relevant and Emerging Literature, COVID-19 Expert Responses to Key Questions, and as a member of the WISE Indiana Internal Advisory Team.

Emily Vardell: Amy's service to MLA has been wide-ranging. She has previously served on committees like the Professional Recruitment and Retention Committee and Annual Meeting Innovation Task Force, as a Rising Star mentor and Colleague Connection mentor, on the MLA Nominating Committee and on the Board of Directors and as MLA Treasurer.

She has been active in the Technology in Education, Public Services, Leadership & Management, and Medical Informatics caucuses and the Midwest Chapter of MLA.

As liaison to this National Program Committee, Amy embodies our theme, “stronger together.” During her election year, Amy called herself a servant leader, saying our members are our greatest strength. She continued, “My overarching vision for MLA involves giving people the right resources and authority to create meaningful change for the association…I strongly believe that the role of a good leader is to listen to those with greater knowledge and understanding of different issues rather than trying to solve problems in a vacuum or be an expert in all areas.”

Amy, it has been a pleasure to have you as our Board liaison–and we really DO apologize that we couldn't find a Sasquatch costume in your size, and my Ewok costume just didn't fit in my carry on, so we'll have to do with you looking great.

Please join me in welcoming my colleague and our 2023-2024 MLA President, Amy Blevins.

## PRESIDENTIAL ADDRESS

Amy Blevins: Maybe we'll have costumes in Pittsburgh next year!

So I'm going to do something a little different, if you don't mind, I wanted to give you all a choice this morning. How many of you would like to see the presidential address that I prepared for this morning? Raise your hands. How many of you would prefer to do a round of karaoke? All right, if I could get tenacious D's tribute queued up real quickly. [laughter] No, I'm just kidding. I told people I wouldn't really do it. I thought about it, maybe next year in Pittsburgh. Thank you everyone. Although it would have been epic. Also, I know we couldn't find a Sasquatch costume, as much as I was hoping that I would get here and they would have one ready. So if at any time during this presentation, you find yourself feeling a little bored, or you're starting to fall asleep, which I know would never happen. I want you to imagine that this is what I look like while I'm speaking to you.

So if you're not familiar, [slide] this is Bigfoot Bae. I follow her on Instagram, and she started posting these videos where she does all these fun dances in a Bigfoot costume, and I love it, so I put the Instagram handle here for you. This is her in Portland, so I felt like it was perfect. So yeah, that's kind of like what I was going for. But I do want to say thank you for being a friend all of my—Oh no, I'm going to get choked up—to all of the people who check in on me all the time and love me and look out for me, like Ryan [Harris], who's up front, who brought me my Diet Mountain Dew, because he knows that's how I like to start my day, in the classiest way possible. For Brandi [Tuttle], who's always checking in on me, and sometimes is my roommate at the conference. And I know that, you know, if I get nervous standing up here in front of all of you, I can be like, there's Brandi, she's got my back.

Shannon Jones, who I saw. Where are you? Shannon? There you are, front and center, who's been a mentor to me since my very first health sciences conference at the MAC [Mid-Atlantic Chapter] chapter meeting in 2006 in Atlanta. It's been wonderful following in your footsteps, except for that one time I was treasurer before you. I really appreciate your wisdom, your kindness, your advice and your support as I've been on this MLA presidential journey. Many thanks to Brenda Linares, who is the incoming president for this year, we meet every other week as an executive board to talk about how we can best support our membership, be strategic in how we're running the association, and solicit and receive feedback from all of you.

So many thanks to my boss and good friend Gabe Rios, who also ran the AI symposium. I picked the best photo I could find, Gabe, there is a photo booth this year too, so that you can also wonder if people are going to put your pictures in front of everybody on a big screen the following year. Many thanks to Tony Nguyen and Emily Vardell. I can say Emily Hurst, sorry, thanks Emily Vardell, for being on NPC. There's a lot of Emilys in this association! Emily Hurst and Tony Nguyen, who, you know, helped me with some back channel thinking during our board meetings. I mean, we never talk in the back channel. Don't worry about it.

Thanks to Dale Prince, thanks to Andy Hickner, who's on the board. Thanks to Dr. Joey Nicholson, who would have been here this year, but he decided to go to Sweden for a Taylor Swift concert. I guess that's fine. I still love him. Many thanks to Beth Ketterman and Jason Cottle, my colleagues from East Carolina University, who I got to see a while ago, for always being there for me and asking me questions about what I'm going to wear to MLA prom, medical librarian prom, also known as the Medical Library Association meeting.

Thanks to Rebecca Graves, who I know I've seen in here, who's been my roommate for years, and I've known (Hi Rebecca!) since back when I was in grad school at University of Missouri Columbia, who's mentored me in teaching and leadership and has just been a wonderful, fabulous, hilarious person.

Thanks to Liz Kiscaden, the Dean of Libraries in Cincinnati, she's too important to be here today, but I know she loves me, and she's thinking about me. And last year she had her “platonic life partner of the future MLA President” t-shirt on which is in that photo there for you [slide]. And to Michelle Malloy, I don't know if she's here this year.

I know there are more people I should be thanking so you can send me all of your angry comments on Twitter later, and I'll be sure to look at them while I can't sleep at night. And so many “frolleagues”, I went back through all of my photos from all of the MLAs and tried to pick the ones that were decent enough quality to put up here. So I've got pictures with so many of you. I hope you enjoy finding yourself in them. M.J. Tooey, who coined the term “frolleagues” for friends and colleagues, she sent me a photo that she said reminded her that we were together, and I put it in there, M.J., so there you are, and I always think of frolleagues whenever I think of MLA.

As Emily was saying before I came up here, our members are the most important part of our association, not only because we can't accomplish our goals without volunteer work, but also because we can't succeed in our lives and our careers without frolleagues, colleagues and friends who can support us, who can give us advice and who can tell us things like, “maybe don't do karaoke instead of a presidential address”. I guess sometimes the advice is good.

And thanks to all of you who I've served with on with different committees, different groups throughout the years. I know I've said this at the new members breakfast, and I've said it many, many times, but this is our association, and I can't say that enough. Like, look at the people who are sitting at the table with you today. If there are things that are missing from the Medical Library Association that you want to see happen, you don't necessarily have to wait for somebody else to do it. You can do it. You can reach out to people and say, “hey, who wants to start a new initiative for webinars, who wants to start a new program related to AI?”, and you can make it happen. So I truly want you to feel empowered. Don't wait for somebody to ask you, because they might not know that you have that expertise.

I would be remiss without thanking my colleagues at the Indiana School of Medicine's Ruth Lilly Medical Library. I know I saw Beth over here. She offered to feed me donuts earlier. Thank you, Beth. Here they all are, pictured [slide]. Some of them are at the meeting today, and some of them are back home. Again, I wouldn't be able to do the things that I've been able to do as MLA President without their support, without them stepping in when I'm feeling a little overwhelmed, and I wouldn't have even run for president if I didn't know that I had their support to back me up. So thank you. I won't cry again or get choked up probably, but I appreciate it.

And thank you to MLA headquarters and staff. I only had really blurry photos, so I'm sorry there aren't photos up here. So that's my reminder that as I'm attending these meetings, I just need to take all kinds of secret photos of people, in case I ever have an opportunity to flash them up here. But Kevin [Baliozian], we've met every other week since I took on the role of president, with the Executive Board, which includes me, Brenda Linares, Tony Nguyen, Shannon Jones and Kate Corcoran and Kevin, If I didn't already say that. We've met every alternate week to talk together. Many thanks to Deb Cavanaugh and Tomi Gunn and Barry [Grant] and Jim [Westwood] and all of you who've been so helpful. Again, we would not be successful without our MLA and MCI colleagues to help us.

This year, I got the opportunity to attend four different chapter meetings. A lot of chapters have moved to having online meetings to be more inclusive, and I think that's wonderful, but I was sad that I didn't get to meet as many people in person. But I did go to the Mid-Atlantic Chapter in person, and it was fantastic. They really rolled out the red carpet. So thank you to all of you in the Mid-Atlantic Chapter, which was my first chapter when I started back way back in 2006, not quite the late 1900s but getting close. Chapters are also important to our association. So find out what your chapter is. I have an image here for you get involved. It's another way to meet people.

So I went through my Presidential Inaugural Address, and I went through my presidential update, and I looked at some of my chapter meeting things, and the first thing I wanted to talk about is the MLA bylaws updates, because it was a truly memorable experience for me. I think Kate [Corcoran] told me that that's the longest business meeting that we've ever had that she can remember. Is that true? It was a long meeting, and while it was hard to talk to everybody for four and a half hours while I was sick, an engaged membership is a caring membership, right? Or an active questioning membership is important. So I think everybody who came with questions, hopefully all of your questions were answered and your situations were resolved. But don't ever stop asking questions. I think it's really important. If we have a 10-minute business meeting because nobody cares, I think that's much worse than four and a half hours. And Brenda, I'm thinking of you, because we have our business meeting coming up on June 5. I'm sure it'll be just the hour and a half that has been scheduled.

I worked on an RFI on public access to NIH supported research as a Board Liaison. I worked on an RFI on Interagency Technical Working Group on race and race and ethnicity standards. So we actually gave feedback to help improve medical subject headings because those are influenced by the demographic standards that have been set by a different organization. We worked on the MLA Role in Societal Issues Task Force to give people more guidance on how you can work with MLA on some of those pressing issues that are happening all around us constantly, more and more every day. And I will say, and I'll say this many times, like Emily and Liz were saying, we are stronger together, and now is the time when we need to support each other and protect each other as we go through these difficult times. I worked with the Censorship and Library Practices Work Group who put a toolkit that's online to support you, if you have questions about things that are happening in your state that might influence your ability, or affect your ability to provide quality health information to your folks.

Be Well MLA was established as a formal committee. This is something that Shannon [Jones] started during her presidency. Nobody told her to do it. She saw a need for us to think about wellness. She saw people suffering, and she was like, “I'm going to do it now”. It's an official committee that you can volunteer for. I'm very excited about this, because those of you who know me know that education is one of my many passions.

We have a new MLA/AAHSL Education Task Force, which will be looking at identifying and adopting standards for both conducting and reporting on educational research, and I will give a slight plug for the AAHSL Competency-Based Medical Education Task Force, who wrapped up their work later last year, earlier this year. We have a presentation on the scoping review that we did, and we're really good at partnering with folks at our institutions, but we're not as good at reporting back on the impact that we have on those people.

So the start to a beautiful new task force. Every task force can lead into a new task force, is what I'm trying to tell you. The fun is never ending! And we have a new MLA platform that's coming at the end of June. And I can't say this enough. I know Kate loves it. Every time I bring this up, it is going to solve all of our problems. So I'm excited to see it, lots of exciting things coming, I think document sharing is going to be on there, so it'll be great. So that's where we've been.

Where are we headed? So yesterday, we had our MLA board meeting. We were together from eight in the morning until noon, hashing out what kind of strategic initiatives we want to roll forward for MLA; thinking about what are the things that we have done in the past, that we continue to do, and where are the gaps where we need to respond strategically? And the things that we were thinking about is, where are we going to suffer if we do nothing, and where are we going to benefit the most if we do something?

So the two things that we were talking about at that time were creating a new leadership track that's focused on leadership at all levels. Because no matter where you are in your library, you are a leader. You are doing projects, you are influencing the strategic needs of your external stakeholders, and we want to give people more of the skills that they need to influence those around them. The other thing that we talked about, are there external audiences that would benefit from being part of MLA that we would also benefit from? So there are things that we do in the health sciences that are unique to us, but there are a lot of things that we do that are not unique to us, like collection development, leadership, data, artificial intelligence. So how do we bring those folks to the table so that we can collaborate with them to better our profession?

How many of you yesterday were at the AI symposium? Raise your hands. Looks like a fair amount. So we had five hours yesterday, which sounds like a lot, but there were interactive panels and table discussions, and AI was recording all of our conversations that were into the microphone, we were told ahead of time. So we started off with talking about this at last year's MLA at the board meeting. And then we put together a working group of folks who Emily and Liz recognized, including Gabe, with the captain's hat. They started with a fireside chat for survey results in the spring of 2024; it's recorded so you can go back and watch that. We had the MLA AI symposium, and for those of you who weren't able to attend, we are going to summarize that at the end of this meeting. And, there's a new working group that's been established to create strategic initiatives around AI, so we're not done with it yet. I believe the bottom line I heard yesterday is that AI is here to stay, and that Terminator 2 is a good movie when you're thinking about artificial intelligence. Did I get that right, Gabe? Okay, good, good. I don't want to misspeak. [Laughter]

I already told you a little bit about this, but I put it here twice, I guess because I'm super excited about it, the joint MLA/AAHSL Education Taskforce. It's being chaired by Dr Joey Nicholson, who wrote his dissertation on the impact that librarians have in medical education. We're going to be more expanded to health sciences, not just medicine.

On May 20th… tomorrow? What day is it tomorrow? I don't know. It's three hours earlier than I'm used to! We're celebrating “a Decade of Doe.” So we're bringing [Janet] Doe lecturers from the last decade, who will address how MLA's core values can serve as the foundation to build the future of our profession and our association. So basically, building upon the lectures that they gave us over the years, and talking about how things have changed and what we can do to be proactive in initiating more change. Because you know what I like to say, if you're not growing and changing, you're becoming stagnant. And I don't want to be stagnant. I don't know about you guys. I won't tell you what to do.

We have MLA Be Well things happening throughout the conference. So how many of you got to see some of the slides with the pictures of the pets and the plants as you were sitting here? Yay. Those are all your babies. And you know what I like to say? Plants are the new pets, and pets are the new children, and I guess children are still children. I don't know if you noticed, but my “daughter”, Wednesday, who I left behind to be here today, is a three year old Mountain Cur cocker spaniel mix, and she's really improved every aspect of my life, but I didn't think she would want to ride on a plane, because she's kind of scared of things.

MLA Reads was started by AAMLA [African American Medical Library Alliance caucus], which Shannon Jones was involved with as well, getting that kicked off. And I know Ryan's worked on this as well. So there's a virtual book club, but they do have a presence here at the meeting where we're going to meet in person. It's Tuesday, May 21, from noon to 1pm in room A105 and the book that they've been discussing is discussing is Algorithms of Oppression: How Search Engines Reinforce Racism, which is tied into our John P McGovern lecture, which is taking place tomorrow (because I know what day it is), May 20 from 10:20, to 11:45, right here in this room.

So last year, when I gave my Presidential Inaugural Address, I came to you with a call to action. I had just gone to the EAHIL meeting in Oslo, Norway, and got to hear from Dr. David Lankes, R. David Lankes, who was the keynote presenter, who talked about radicals, rebellions and saving our communities, and how we can deal with the challenges and sometimes attacks that we face as librarians and information professionals. And I want to remind you, We Are MLA. We need to think about what we want to do; we want to think about what we can do; and what we should do and how we will do it. And I say how we will do it, because a lot of times I think we have really great ideas, but we don't think about how we're going to put it into action. And I know that's something that we're trying to do as an association. Words are beautiful and fine, but when there's no action behind it, it feels empty and performative.

So what can we do to inspire and enact change and build our futures? Don't let the future happen to you. Don't be scared of AI stealing your job. Think about how you can leverage your expertise in AI to show people that you're invaluable. There are things that are out of our control, but there's a lot of stuff that we can still influence. I like to think of myself as an optimistic realist anyway, so that's my call to action as I'm wrapping up my presidential year, but still serving on the executive committee for another year. As Beverly Murphy says, who I know is right here in front, “I am MLA, you are MLA, we are MLA”. So every time you're like, “why is MLA doing this?”—You're part of MLA. You have a voice. Make sure that it's heard.

And then I had to put Wednesday in here again [slide].

Thank you for your time. Sorry we didn't really do karaoke. I hope you don't feel too horribly misled by that. I know we're ending a little bit early, so if you want, I could make up some other things to say. But otherwise, I appreciate seeing all of you this morning. Use this extra time to network, drink water, take breaks and feel empowered. [Responding to audience comment] Be well? Is that what you said? Bev, be well, and I'll see you throughout the rest of the meeting! [Applause]

Emily Vardell: Amy, thank you for updating us on the state of MLA!

And thank you all for joining us today to kick-off MLA '24. We look forward to spending time with you this week for a variety of fun events and engaging learning sessions!

## OTHER PLENARY SESSIONS

### May 15, 2024, Joseph Leiter NLM/MLA Lecture: “Responsible AI in Healthcare: A Practical Approach”

**Keynote Speaker:** Dr. Maia Hightower

The Joseph Leiter NLM/MLA Lectureship was established in 1983 to stimulate intellectual liaison between MLA and the National Library of Medicine (NLM). Lectures are chosen for their ability to discuss subjects related to biomedical communications. The lecture is presented every other year at NLM and in alternating years at the association's annual conference.

Artificial intelligence (AI) has the potential to transform healthcare and improve health outcomes, but it also poses ethical, legal, and social challenges. Dr. Maia Hightower, founder of Equality AI and former EVP, Chief Digital Transformation Officer at University of Chicago Medicine, discussed some of the principles and practices of responsible AI in healthcare, drawing on her experience as a CEO and a physician. Dr. Hightower shared some examples of how AI can be used to enhance patient care, clinical decision making, and health equity, as well as some of the risks and pitfalls to avoid. She also offered some recommendations on how to foster a culture of trust, transparency, and accountability in the development and deployment of AI in healthcare.

View the lecture online: https://videocast.nih.gov/watch=54584.

### May 20, 2024, John P. McGovern Lectureship

**Keynote Speaker:** Dr. Safiya U. Noble

The McGovern lecture series was established in 1983 in honor of John P. McGovern, MD, noted physician, educator, author, medical historian, and honorary member of MLA. Through the years featured speakers have addressed topics relevant to the health sciences information profession. The 2023 McGovern Lecture was a structured conversation with Dr. Noble speaker and the 2024 National Program Committee Cochairs Liz Kellermeyer and Emily Vardell.

Dr. Safiya U. Noble is an internet studies scholar and professor of Gender Studies and African American Studies at the University of California, Los Angeles (UCLA) where she serves as the faculty director of the Center on Race & Digital Justice and co-director of the Minderoo Initiative on Tech & Power at the UCLA Center for Critical Internet Inquiry (C2i2).

She is the author of a best-selling book on racist and sexist algorithmic bias in commercial search engines, entitled *Algorithms of Oppression: How Search Engines Reinforce Racism* (NYU Press), which has been widely-reviewed in scholarly and popular publications.

National Program Committee Cochairs Liz Kellermeyer and Emily Vardell talked with Dr. Noble about her information sciences degrees, her research journey, her book, how her background in library and information sciences offered a lens to understand and address complexities and nuances around algorithmic bias, how bias should be approached, and how search engine algorithms translate to AI applications. Audience members were also able to ask questions via the conference app.

The conversation was streamed for a virtual audience and is available to attendees on demand.

### May 20, 2024, Janet Doe Lecture: “A Decade of Doe”

**Moderator:** J. Dale Prince, AHIP

Panelists: BarbaraA. Epstein, AHIP, FMLA; Sandra G. Franklin, AHIP, FMLA; Michelle Kraft, AHIP, FMLA; Michael R. Kronenfeld, Ahip, FMLA; Margaret Moylan Bandy, AHIP, FMLA; Gerald J. Perry, AHIP, FMLA; Elaine Russo Martin, FMLA; Chris Shaffer AHIP; M.J. Tooey, AHIP, FMLA.

During this special event celebrating the 125th anniversary of our association, Doe Lecturers from the past 10 years were asked to share their thoughts on how MLA's core values as an association can help sustain our impact and help us thrive as health information professionals in the decades to come.

The panel was streamed for a virtual audience and is available to attendees on demand.

### May 21, 2024, National Library of Medicine (NLM) Update

**Speakers:** Dianne Babski, Director, User Services and Collection Division, National Library of Medicine; Martha Meacham, NNLM Project Director, National Library of Medicine.

Since its founding in 1836, the National Library of Medicine (NLM) has played a pivotal role in translating biomedical research into practice and is a leader in information innovation. As one of the twenty-seven institutes and centers at the National Institutes of Health, NLM advances research in bio-medical informatics and data science and is the world's largest medical library.

Dianne Babski discussed the new 3C framework (Collect, Curate, Connect) and organizational restructuring, including the launch of the Data Set Catalog and the Center for Clinical Observational Investigation (CCOI). She noted PubMed and PMC improvements, such as expanded proximity searching and updated indexing algorithms and emphasized NLM's commitment to open science and women's health research, along with the integration of Gen AI in various projects.

Martha Meacham highlighted the NNLM's regional network, training programs, funding, and efforts to support rural and tribal health.

## SYMPOSIA SESSIONS

May 19-21, 2024

Symposia sessions were held in person throughout the conference; with several streamed for virtual attendees.

### Collection Development & Resource Sharing Symposium

- Getting to Open: Everything You Need to Know about Crafting Open Access Agreements
- Collective Collecting and the Challenge of Preservation
- Getting Started with Collection Development: An Interactive Workshop on Selecting Resources for Your Library Community
- AI, Machine Learning, and Collections: New Approaches and Tools for Description and Discovery

### Leadership & Management Symposium

- Inclusive Library Leadership: Empathy, Care, and Compassion as Leadership Strengths
- Building Culture and Community in a Hybrid Workforce: A World Café Conversation
- Leading and Serving in an Unscripted World: Leadership, Librarianship, and Improv
- Inside and Out: Supporting and Empowering Your Staff by Promoting Their Achievements

### Data Management Symposium

- Data Equity: Exploring What It Is and Why It Matters
- Data Partners: Fostering Librarian-Researcher Partnerships
- Data Instructors: Providing Data Literacy as Part of Information Literacy
- Data Allies: Building Institutional Support Networks

## PROGRAM SESSIONS

May 19-21, 2024

During the MLA '24 conference, there were 18 immersion sessions, 120 papers, and 75 lightning talks. The live immersion sessions included interactive breakout sessions, Q&A, and virtual chat with presenters.

The final version of the presentation abstracts is included as an online-only supplemental file to the October 2025 issue of the Journal of the Medical Library Association (see Appendices).

## POSTER SESSION

May 20, 2024

The main MLA '24 Poster Session featured 127 posters, of which 14 were available on-demand (electronic-only).

### MLA Research Training Institute Ignite Poster Session

The MLA Research Training Institute had a dedicated in-person poster session. The sessions featured fast paced and engaging “ignite” talks by the RTI fellows about their research projects that are making meaningful contributions to their communities and expanding our health information research knowledge base.

The final version of the poster abstracts is included as an online-only supplemental file to the October 2025 issue of the *Journal of the Medical Library Association* (see Appendices).

## OTHER SPECIAL EVENTS AND RECEPTIONS

May 19, 2024

- New Member and First-Time Attendee Program and Breakfast
- Speed Date Networking
- Leaders' Recognition and International Visitor Reception
- Welcome Reception & Opening of the Hall of Exhibits

May 20, 2024

- Communities Lunch
- Research Training Institute (RTI) Poster Session
- MLA Fellows Luncheon

May 21, 2024

- Living Library Session

## EXHIBIT HALL AND EXHIBITOR PRESENTATIONS

The Exhibit Hall was home to 65 booths and 222 exhibitor representatives who presented various demonstrations of their products. The Exhibit Hall began with an opening reception on May 19, 2024, 5:30-7:30 p.m. with Entertainment provided by the Portland Unipiper. The Exhibit Hall was open on May 20, 2024, 9:00 a.m. - 5:00 p.m. and May 21, 2024, 9:00 a.m. - 1:30 p.m.

Exhibitors held both sunrise seminars, lunch and learns, as well as technology showcases to highlight new products.

### May 20, 2024

Sunrise Seminars

- Wolters Kluwer - Excellence in Evidence-Based Practice: the Library as a Catalyst in Optimizing Healthcare Outcomes
- Elsevier

Technology Showcases

- Elsevier Clinical Key - ClinicalKey AI and the Library: Advocating for Better Clinical Decision Making at the Point of Care
- TDNet - TDNet AI – A New Era of Knowledge Discovery
- Covidence - Covidence: Systematic Review Technology Now and for the Future
- BMJ - Understanding the Impact of US Medical Research at the Point of Care

Lunch and Learns

- Wolters Kluwer
- Springer Nature - Empowering Scientific Collaboration: Unveiling Protocols.io

### May 21, 2024

Sunrise Seminar

- American Psychological Association

Technology Showcases

- National Library of Medicine - Enabling Discovery of Biomedical Research Data: Introducing the Dataset Catalog
- Clarivate - EndNote, Clarivate, and the Systematic Review Lifecycle
- BMJ: BMJ sneak peek - Rising to the Comorbidities challenge
- National Library of Medicine - PubMed and PubMed Central Update

Lunch and Learns

- JOVE

## RESOURCES AND SERVICES

The online conference scheduler allowed the public audience to peruse programs and events online. Registered attendees could use the conference app or virtual portal to identify favorites to attend and receive tips and reminders onsite in Portland and use the virtual portal to view streamed or on-demand content. Individual messaging was available on Twitter using the hashtag #mlanet24. Conference blog posts are available on the MLA website. The MLA Professional Recruitment and Retention Committee (PRRC) sponsored the in-person MLA '24 Virtual Resume Clinic.

## CLOSING SESSION, AI SUMMIT REPORT

May 21, 2024

Amy Blevins, 2023-24 MLA President

Emily Vardell, AHIP, MLA '24 Cochair

Liz Kellermeyer, AHIP, MLA '24 Cochair

Brenda Linares, AHIP, 2024-25 MLA President

Emily Brennan, 2025 NPC Cochair

Mary Beth McAteer, AHIP, 202 NPC Cochair

Gabe Rios, Chair, AI Leadership Summit Working Group

Amy Blevins: Hello, everyone! If you forgot who I am, (which you can soon, because I'll be done with my presidency), I'm Amy Blevins, your 2023 2024 MLA President. Thank you all for coming to MLA '24 in Portland or virtually. It's been a really great time connecting with all of you.

It has also been a jam-packed conference with content contributed by MLA's talented members, and by content experts in new areas. I have really enjoyed seeing how all of you have shared your experiences and your research.

Nothing points more to “Stronger Together” than to experience the amazing creativity everyone is providing in institutions across the globe to our users and stakeholders.

But I think that right now we all are experiencing both exhaustion and exhilaration! Let's see a raise of hands in the room: how many of you heard or found at least one idea you want to take back to your own institution? Oh, my goodness, it's very dark, but I think everybody raised their hand. How about our virtual audience? Please let us know in the chat within the app what you learned and what you're taking back after the conference.

Any conference involves complex interactions between presenters, volunteers, staff, and venue – and that takes good leadership. Please join me in a round of applause for our extraordinary MLA '24 National Program Committee Cochairs, Liz Kellermeyer and Emily Vardell!

Emily and Liz, from the bottom of our hearts, thank you for leading your NPC team to bring us this extraordinary conference–and to bring us back to Portland after 37 (log or short, depending on how you feel about time) years–and a 2020 “false start”. [Applause]

We have a very busy session ahead of us today with lots of information to share–but we'll hear more from Emily and Liz a bit later. [Aside:] So don't get too comfortable on that couch.

We began this conference with traditional continuing education options, but also a focused Saturday afternoon summit on Artificial Intelligence and how we as health information professionals are using or can use AI (as a general term) in our workplaces to help share, help serve our stakeholders. I was forgetting what we were doing to our stakeholders, it's serving them. Don't worry. AI is, I'm sure, benevolent.

To review the progress of that session, helped by generative AI, please welcome Gabe Rios, chair of the AI Leadership Summit planning group, and director of the Ruth Lilly Medical Library, and consequently, my boss and sometimes friend.

Gabe take it away!

Gabe Rios: Thank you. Amy, well, so I heard we have two and a half more hours, right? So I'm going to fill that time. Let me go ahead and… I'm kidding! We're not going to do that.

But we did have a five-hour summit on Saturday. You know, it didn't seem like five hours until maybe the four-and-a-half-hour mark. Then I was kind of like, is this five hours? It took a second. So let's talk a little bit about the AI Summit.

First off, AI activities that we've been doing since November 2023: there is a committee. This was sort of an ad hoc committee that was put together in August, and we've been meeting since then. Back in the fall, there was a free webinar, which was a tool kit for evaluating AI tools. We also had a survey that we put out, this committee put out, and that went out November, December. We distilled some of the results of that. We did a fireside chat that was in March. We were planning February. Wanted a kind of cold and sitting around a fireplace sort of feel but, you know, we were a little late. It's still cold some places in March, so we kind of got the idea, but that was around how librarians are already implementing AI and supporting AI in their environment.

So it was kind of showing us like, these are folks that are already using AI. It may not be generative AI, it may be machine learning, but they're already using it in their environments. There was also another webinar. And then finally, we're to where we are this week, which is we had an AI Summit this last Saturday.

The committee is Michelle Cauley; she's the interim director at UNC Chapel Hill. Sarah Jewell from Weill Cornell University. Shannon Jones from MUSC. Amy Chatfield from University of Southern California. Lauren Jones from BMJ, as we also had vendor representation on this committee, because we want to see how librarians can work with vendors to make these products better. And then MLA staff, you know, Kevin [Baliozian] and Barry [Grant]. And actually, Kate [Corcoran] is not mentioned in there, but she was also on the committee with us.

So what we did again, five hours, is actually kind of a good amount of time. We broke the day up into three sections. The first section was a panel, and it was level setting, and set that sense of urgency we've all been starting to feel, I mean, last, last year at MLA, I would say there was a very different perspective on AI. There were some AI posters and papers out there this year. There definitely were as well too, and we've seen that perspective evolve over the past year.

So a little bit of a summary from that first panel. The speakers talked about the importance of ethical and responsible AI development in health care. They talked about how human oversight is needed, transparency in the development of AI, addressing bias in AI models. NLM talked about leveraging data science and Gen AI to provide tailored health information and infrastructure to global users. NLM also developed a generative AI Community of Practice, and I actually saw that in several presentations, they're developing these community of practices at different institutions around AI.

The speakers also discussed the potential for natural language processing and using synthetic data to improve data quality and accessibility in healthcare. So for synthetic data was new to me about a month ago. I had not read up on it and heard of it, but basically, synthetic data is data that's artificially generated. It's created through simulations, algorithms and models, rather than being collected from real world sources. So think about how handy that would be, instead of using real patients, real patient information, if you're working on a cure for a rare disease, and you're able to build a much larger sample set using synthetic data, being able to solve problems that way. That is just one example we heard during the summit.

Our second session—this was fun. This was basically a time for librarians to talk to each other and share challenges and opportunities in AI. There are several slides, and the way we set it up is we had people sitting at tables. They were generating responses to questions, they were feeding them into a Google form. It went into a closed AI system that MLA has, so it's not just going out into the wild. The AI system that MLA is using is called otter AI. That information was distilled down…This session was facilitated by Michelle and Sarah. I went ahead and highlighted some key points in here, because even that distillation, it was, it was a lot of information. Again, think about it, we had over 100 participants in this and each participant probably answered several questions, so hundreds and hundreds of responses went into the distillation here.

So the first thing was, what's happening with Gen AI at different libraries and institutions, a lot of faculty training and workshops, using cleanup for institutional data and institutional repositories, research assistance. One phrase I heard that stuck in my mind is we are the the original or OG prompt engineers, Michelle Kraft said that she always has a catchy phrase, I think, so—systematic reviews. You've already seen some examples of that.

Add to the fireside chat we had earlier this year, some education support, employing AI to develop lesson plans, to create rubrics, clinical trials. I'm just going to, like I said, I'm going to flip through some of these, because I think some of these [examples] get a little bit redundant and in the weeds. These slides will be available in the program, I'm assuming? So, yeah, Kevin just gave me a nod.

As far as library initiatives, there were several things that we can start out doing, so hosting AI literacy events or creating AI guides. I know that is something that we do. We create guides all the time. That's usually the first step.

Vendor collaborations and testing. That was the reason we invited vendors to this summit. We wanted to have some vendor input, we really need to have that relationship go both ways. Let's see. We've talked about a lot of these issues, like data privacy and security. I'm not going to go into all these, because, again, even the summary, when I tried to shorten the summary, it gave me a really long run on sentence. So I'll just let you know there's a lot of information here we'll be sharing. All right, let's go the last panel.

Kristi Holmes moderated the last panel. Dianne Babski spoke on that. Dr. David Doher from OHSU—he is a clinician who has developed a training framework on upskilling staff at OHSU and Seymour also, also was part of that panel from Hopkins, and then Natalie Bucha Smith, she's from LC labs, Library of Congress labs. They're doing a lot in the AI space. If you Google LC labs, there are some great links. There are some GitHub links to ways to evaluate AI tools, whether you need them or not. How should you do go about evaluating AI tools, etc?

But again, in general, not to go through another 10 slides. Here's one slide. So this panel, we focused on skills and expertise and roles that are coming out for librarians as a result of Gen AI. We continue to emphasize the importance of critical thinking and education as AI systems becoming more prevalent in healthcare, this is something they're doing at OHSU, the LC labs. They're using AI to generate cultural context for audio and video. We heard a lot of a lot a lot about that at the McGovern lecture, too, if you were attending that, we discussed some new roles that are created by new technologies and AI, and how these roles would require library leadership, but also would require librarians to be project managers. So if that is a new skill set for you, or a skill set you're thinking about, this is a good time to do it, especially with projects related to AI.

And then, just in general, AI can outperform humans in big data tasks, but humans are still, you know, better, I would say, at personal care and therapy. And I know that's going to be up for debate, because I know there are AI chatbots now that are helping with mental health issues, just because our health system, our for mental health care, is not quite up to what the needs are in our population. So this is the slide. So everybody you know get up and stretch and pay attention. This is the last slide. This is the slide I really want you to look at if anything. Take away from here. These are some action items that we came out with after this summit. Develop a guide. And I know again, that sounds kind of funny, but we always develop guides. But this is a good place to start that we are a player in this sphere.

Attend webinars. Continue to educate yourself. Gen AI is evolving so quickly. This is, though, a lot like most other revolutions that I've encountered in this field in the decades I've been in it. Experiment, we heard that throughout the AI Summit, take the time to download those tools you hear about, whether you hear about them from your fellow librarians, or if you attend other AI webinars that are not from librarians, you may hear about different tools that are out there. Hold events in your spaces that promote responsible AI use. We know our students are using it, at least in an academic health center. We know they've been using it. We know that sets off some alarm bells for our clinician educators, but we know it's here to stay, and they're already using it. Consider your research support services in a new light. Again, within the context of Gen AI, promote your knowledge. That's back to what I said, even starting to create the guide, you are inserting yourself into the sphere of AI. Find out what's going on around campus. Collaborate with people around campus that are doing AI projects.

Investigate impact on licensing agreements. You might have some of your patrons already using data that you have and putting it within closed AI systems, which they're not supposed to do without permission. So there are librarians that are working on navigating licensing agreements around that. Then, if you really have resources and have a larger shop, test large language models and develop prototypes. So again, that's probably out of the reach for most of us, but there are some institutions that have that.

There is an AI imperative strategic goal. This came out in March, was voted on by the board and approved. I don't know if you read it. I'm not going to read it today. It is on the website, though, so keep that in mind. And then my last words. I am excited. I'm also cautiously optimistic about this. You know, I know, if I think about all the things that happened in the decades I've been a librarian, and how excited we were about, oh, web 2.0 or whatever—hey, this is the next thing. So here we are, here's the next thing. I would rather jump on it than be on the sidelines. I'd rather participate. Because if we don't participate, there's going to be another entity within our institutions that is going to rise to that occasion, and we will be cut out of this.

That would be my advice. Remember yesterday what the McGovern lecturer said about the librarians role in technology advocacy and critical thinking. Critical thinking is really most important when you use these tools. Even during the Decade of Does, a lot of them mentioned AI and they were doing reflections. If you didn't attend that session, that is also recorded, and I would take a look at that and remember the importance of distinguishing between data and evidence. We're generating a lot of data, and even just looking at the tool we used in generating these summaries, it's not a perfect tool, and still it generated. Kevin sent me a lot of information and trying to distill that down into a short amount of time–daunting.

That's sort of why I said I need the next two and a half hours, because it was still a lot of information. Throughout that summit, we talked about the need for critical thinking. That is something we do. We also teach that of our students, and that's something we need to teach our patrons and our faculty. Faculty, student, staff, and then remember, this is pretty much the Wild West. This is, again, another innovation is created where there's not policy, there's not governance that's behind this. Listening to the news this morning, Sam Altman, ChatGPT, had released a voice for ChatGPT, and it sounded very much like Scarlett Johansson, which she didn't approve of, and she was asked by him to use her to license her voice. So there are a lot of issues.

There's a lot of policy and governance that is not there yet, and we have a chance to be a part of this–not necessarily all the creative endeavors, but creative endeavors within our own sphere. So I will stop there. Otherwise, I will keep running until and Amy, Amy works with me, so she knows I will keep a meeting going to the bitter end, so No, I won't do that.

Amy Blevins: I think we're supposed to high five now. Well, thank you very much, Gabe, for that extremely extensive summary of what happened on Saturday. Do you guys feel like you were there? I was there, so I feel it. Thank you very much.

And now an area near and dear, I think to all of us, is a new technology solution for MLANET So those of you who've heard me talk about this know that it is going to solve every single problem that we've ever had or could imagine. So I'm very excited about that. I know every time I say that, Kate [Corcoran] thinks, thank you, Amy, thank you for making these promises that we can definitely uphold.

In this we are partnering very closely with our association management company MCI USA, because we are both investing financially in the solution. So MLA is partnering with MCI. We're not the only organization or association that's using the new tool. We've been on the current system since way back in September of 2015 and lest we forget, that system enabled us to bring most of our communities under a single roof, introduced easier management of the website and allowed us to integrate our membership system, communities and website under a single system. How many of you were around for the platform coming out in 2015? Yeah, and it solved all of our problems.

But then we, I guess, got new problems. The board and staff began this process several years ago by looking strategically at what we wanted to accomplish with the new technology. The why? Because despite what the system could do, it has aged, shall we say, not so gracefully. Unlike all of us aging like fine wines, we saw four critical areas of focus for the new technology.

Is this the right slide? First is improving your user experience with the website, with interactions with MLA and with information you need to know front and center. Second was to improve and increase your engagement with MLA, and by that we mean with other MLA members, whether they're AI or real people, communities, and easier processes to do well, everything. For example, your education's transcript can inform your AHIP or specialization application and committee members are notified automatically when your application is submitted and ready for review. Third is to increase our staff's productivity, and by that, we mean to take them out of highly manual data operations or needing to support you on confusing forms or processes and instead give them the opportunity to focus their attention on ways to improve MLAs value to our membership, and fourth is to have all of these things improve MLAs bottom line and overall sustainability.

So how do we get there? To be clear, MLA is not using AI, but we spend a lot of time talking with members about processes and workflows as well as pain points and communications. So anytime you have a problem, you just submit that to somebody, and we will look at it and see how we can improve the process. Staff have taken that information to review Association business rules, user journeys, taxonomy, and data authority to better solve your experiences with all the things MLA offers. And let's be clear, all of this is happening now. So if there's a delay in staff getting back to you, they are not ignoring you but are probably stretched a little thin with both the meeting and the website build. We've had the benefit of MCI design staff in developing wireframes design and navigation and appreciate the members we've consulted to get initial feedback. So now, how about a quick pick at a few things? Do you guys want to see a quick peek? Oh, thank goodness, because you were going to get that anyway.

So, a primary objective that has been to reduce the clutter in a more modern and accessible site. If you're an active member, you may have to-do lists in your personal dashboard, like voting in the election or reviewing jury applications, or you might just want to see your communities and the latest posts in one place to decide if you want to read or respond or pretend you never saw it. We also want to provide seamless access without extra logins all the time to all of the services that MLA offers. We want to provide easier access for members to join public or caucus communities. And you can see a First Data Migration example here from a while ago. And this is not going to be your view, your view will depend on your login status and what communities you're already associated with, whether committees, councils or caucuses. Keep in mind you are only seeing pieces of the new site because the build is still in progress. Our timeline is critical. The current site will go offline at the end of June. So we're guaranteed to have a new site in July.

What I'm getting at that means you're going to start hearing from us about things we need from you, and more details about this timeline, the transition will be an orchestration for us all, as we disable current services to make sure we migrate to the latest data from all the sources on MLANET and MEDLIB-ED, migrate, rewrite, archive or retire more than 8000 pages of content and then restore access to you on the new platform. It's as easy as that.

Please watch MLA connect your email and the MLANET home page closely over the next few weeks. Here's some I am MLA stories. Here's the timeline that I told you about that you can pause in the recording later and totally look at.

So that gets us to, what can you do to make sure that we're successful in this new platform migration? As we remind you to make sure your information is up to date, that you're not in mid CE course, that you are approving your EFTS transactions. Your assistance will make the transition smoother for you and for us in MLA. And what are we? Who's MLA? That's right, We Are MLA so we're all, you know, celebrating together in the success of this new platform.

At this point, I would like to ask some special guests to join me on stage. I mean, the podium, they're already on stage, having a good time on the couch. Would you please welcome our NPC cochairs for 2025 Mary Beth McAteer, AHIP, and Emily Brennan.

Mary Beth McAtteer: We would actually love for our 2024 NPC cochairs to join us at the podium as well. Emily, Liz, and you know what? How about our Board Liaison, Brenda Linares, also, we're going to perform some podium choreography for you.

The attendees here in Portland and online have shared how inspired they are by our speakers and by the innovative and creative work of our colleagues and presenters, together, you and your team had created; created extraordinary content in immersion sessions, papers, education sessions, posters and lightning talks, we thank you so much for your selections. Thank you. [Applause]

Brenda Linares: To the four special programming committees. You put together three great symposia on collection, development and sharing, on leadership and management, on data services, and an AI summit that we heard about earlier. We received so many comments about your excellent selection and the topics that we cover with different speakers and really appreciate the live streaming too for the people attending the conference virtually. We thank you for contributing to the success of the 2024 MLA. [Applause]

Emily Vardell: Thank you so much. We've been receiving such wonderful feedback, and so glad to hear that this has been such a meaningful conference experience for everyone. To the 2024 Local Assistance Committee, thank you for graciously hosting us in your totally awesome and weird city. While we may not have gotten a glimpse of the giant Sasquatch, we do have Amy's tiny Sasquatch available for viewing. We've certainly enjoyed the TriMet passes to easily get around, the recommendations for sites and visits and the amazing interactive restaurant guide. I think we can all agree, we've had a fantastic time in our host city of Portland. [Applause]

Liz Kellermeyer: And new this year, we wanted to give a shout out to the 2024 virtual assistance committee who sponsored the wonderful Padlet, provided information for virtual attendees in advance of the conference, and created networking sessions specifically for our virtual registrants. Thank you for contributing to the success of MLA '24. [Applause]

Brenda Linares: And of course, we couldn't do all of this without the conference staff. You always help plan the logistics and help us have a great, terrific meeting. We all are still impressed by how you figure out what to do when we need to where things need to go. And you always help both the in person conference and also the virtual conference. And so if you saw them, they were all running in different places, here and there, and they were always doing a good job. And at the same time, they knew what they had to do. So if you interrupted them and talked, they would go to where they needed to go. Thank you for all your hard work, and you know it really paid off, because, as you can see, we had a great conference. So thank you. Thank you. Thank you, MLA staff. [Applause]

Emily Brennan: We extend our profound appreciation and gratitude to MLA exhibitors and sponsors and hope to see you next year at our meeting. Our huge thanks to our talented information professionals and people of Portland for this excellent conference: you provided us with unlimited possibilities and a high bar to meet for our conference in 2025. [Applause]

Liz Kellermeyer: Okay, so we know we're going to be in Pittsburgh, and we know that we're going to get the official invite in August. But is there anything you can share with us now?

Emily Vardell: Maybe just a little hint?

Mary Beth McAteer: Well, maybe a few details. We will be at the David L Lawrence Convention Center in Pittsburgh with an attached hotel, and our chosen theme, drum roll, is “Spanning Tradition and Innovation.”

Brenda Linares: So I hope to see many of you there, so start preparing, and hope to see you in Pittsburgh to have a lot of fun and enjoy the city. And so you're going to get more information about that conference in in coming months. Please be on the lookout for that information. Without further ado, this is kind of like the sad, happy part of the of the meeting, I would like to turn the podium to our president of MLA. Amy.

Amy Blevins: Oh, thank you so much. Incoming president, Brenda Linares, I can't wait to turn everything over to you. [Laughter]And thanks to all of you for coming up here and sharing that sneak preview of all the exciting things happening in Pittsburgh. Somebody told me in the exhibit hall that there is this thing that moves people up hills, and I was just imagining like this giant hand grabbing people and taking them up a hill. So I can't wait to find out if that's what it is.

I hope all of you who have been in Portland have had the opportunity to visit the displays about MLA's history and to get your photos taken next to the anniversary booth. If anybody right now is thinking, “Oh no, I did not get a photo with the anniversary booth!,” if you look back in the corner, it's right there. So you still have the opportunity to take pictures with your friends and find out if they're going to come back to haunt–I mean to enjoy later at future MLAs.

MLA officially started its 125th anniversary year on May 2, 2023, and as we have come to a close with this conference, we are also closing out our 125th anniversary year. Not all of our activities are complete. Our updated milestones and notables will still be in articles this summer, and that will likely be on the new MLANET. It's coming in July, which will be a wonderful transition. Now, before we finish waving our fans around and celebrating the 125th year, I would like to invite Mary Langman to join me up at the podium.

As many of you know, Mary retired officially in January of 2024 but we couldn't let her get off that easily without celebrating her in person in front of everybody. So thank you so much for being gracious and allowing me to invite you up here last minute so that we can all thank you for all of the hard work that you've done throughout the years, for MLA and for kicking off our 125th anniversary and working with so many of us to make things a success. So thank you, Mary. I'm so glad you joined us from Chicago. Would you like to say anything?

Mary Langman: Thank you. It's been a wonderful 35 years, and I couldn't have had a better group of people to work with and make friends with and learn from and mentor and just be part of your community. So thank you.

Amy Blevins: I promised Mary I wouldn't make her stand up here for that long. So for those of you in the room, we have treats. Did everybody find the cake? Yeah, okay, awesome. Is there any left? If you didn't get any cake, or you want to pretend you didn't, please grab some, go to the back corner, get a picture with the photo booth, and for those who of you who are joining virtually from online, thank you so much for attending MLA. We want to let you know that we missed seeing you in person, in our hearts and in our minds, but we are excited to hear and read all of your chats online, and maybe we will see you in Pittsburgh next year. So is everybody ready to leave the stage? Do you have your fans ready?

Happy sesquicentennial, I can't wait to see you all in Pittsburgh. [Applause]

## OTHER MEETINGS AND EVENTS

*Please note not all meetings are included due to system transition June/July 2024.*

**Academic Librarians**: Academic Librarians Caucus Meeting, September 25, 2024, December 4, 2024, UME Fall Chat, November 13, 2024; **African American Medical Librarians Alliance**: Navigating Disruption in Libraries, January 25, 2024, Strategies for Thriving Amidst Disruption, March 28, 2024, Words of Wisdom, February 1, 2024; **Clinical Librarians and Evidence Based Practice**: Leadership Meeting, August 6, 2024, Caucus Meeting, December 9, 2024; **Collection Development**: Leaders Meeting, February 12, 2024, Business Meeting, April 20, 2024; **Federal Libraries**: Caucus Meeting, January 26, 2024, Business Meeting, April 19, 2024; **Health Association and Corporate Librarians**: HACL Meeting, November 12, 2024; **Health Humanities**: Quarterly Speakers Event, May 30, 2024 Caucus Meeting, October 20, 2024; **Hospital Library**: “Why I Love Being a Hospital Librarian” February 14, 2024, Board Meeting, July 8, 2024, September 9, 2024, November 11, 2024; **Libraries in Health Sciences Curriculums**: Business Meeting, April 29, 2024, Caucus Meeting and Update, September 19, 2024; **Medical Library Education**: Mid-Year Business Meeting, December 13, 2024; **New Members**: October Meeting, October 17, 2024; **Pharmacy and Drug Information**: Caucus Networking Session, February 22, 2024, Business Meeting, April 20, 2024; **Public Services**: Open Forum, October 3, 2024; Science: Business Meeting, September 17, 2024, November 19, 2024; **Systematic Reviews**: Bi-Monthly Meeting, June 12, 2024, August 14, 2025, October 9, 2024, December 11, 2024; **User Experience**: Caucus Session, February 1, 2024, June 6, 2024, August 1, 2024, October 3, 2024, December 5, 2024; **Vision Science**: Business Meeting, April 17, 2024.

## 2024 ANNUAL BUSINESS MEETING, PRESIDENTIAL INAUGURAL ADDRESS

June 5, 2024

Amy Blevins: Good afternoon, everyone.

I'm Amy Blevins, your 2023-2024 President.

It's my pleasure to welcome you to the 123rd Annual Business Meeting of the Medical Library Association.

This is our fifth electronic business meeting. Our first business meeting was in 2020, as a consequence of COVID-19. We chose to continue holding our business meetings virtually instead of at the annual conference. This helps us be more inclusive for this meeting and frees up time for conference content.

It is wonderful that so many of you are participating with us today.

Here is today's agenda:

We will begin with the In Memoriam. I will present my Presidential Address.

We will then move into the business portion of our meeting which will include:

- the presentation of your current MLA board of directors
- reports by the MLA Treasurer and Executive Director
- election results and the presentation of your new MLA board
- and this year we will … not have any proposed revisions to the MLA Bylaws. That should save us about 5 hours!

We will conclude today's session with an Inaugural Address from your 2024-2025 MLA President, Brenda M. Linares, AHIP.

Before we get started, here are a few guidelines:

- All MLA gatherings and interactions need to respect MLA's Code of Appropriate Conduct. Please consult it online. If you need to report a violation, there is a link to do so on the web page.
- We are using Zoom webinar today, with which, by now, you are all likely extremely familiar.
- We will be using the “raise your hand” feature” for official business only. We'll walk you through procedures in a few minutes.
- We will NOT be using the Q&A feature today. During the year, we offer topical open forums on many areas of MLA to invite conversations and questions, and offer a better experience for dialog.
- Feel free to use the chat, but please note that we will NOT be monitoring the chat for questions. If you have a question that you would like addressed after the meeting, please email president@mlahq.org.

MLA upholds a tradition of pausing to honor our cherished members who have passed away over the past year. Their counsel and friendship will be deeply missed.

Kate Corcoran shared photos and stories of the following MLA members who had died since the last annual meeting. Please watch the JMLA for official obituaries.* Ted Thaxton Campbell, 1936-2023

- Rosalind Farnam Dudden, 1944-2023
- Ernesta Eunicia Greenidge, 1951-2024
- Joseph John Harzbecker, Jr., AHIP, 1959-2024
- Gloria Jane Bridges, 1949-2024
- Fred Wilburn Roper, 1938-2024

*Amy Blevins presented an update to her Presidential Address given in Portland for the MLA Business Meeting audience. It is available for viewing on MLANET.*

Amy Blevins: To get us started with the annual business meeting. I would like to recognize Chris Shaffer, MLA's Parliamentarian. Chris will assist us with the business portion of our meeting.

Chris Shaffer: Thank you, Amy. So hopefully, this won't be a five-hour business meeting. We don't have any bylaws this time. Hello, fellow MLA members. As Amy mentioned, this is our 5th electronic business meeting, so my explanation should be familiar to many of you. It'll be a little shorter this time around. Robert's Rules of Order allows business to be conducted by unanimous consent, which removes the need for discussion and a full vote. Any member present may object to unanimous consent, and require the President to open the floor for discussion and put the question to the members for a vote. Today we plan to use unanimous consent, and we'll use the raise your hand feature to allow members to register an objection. Note that all new business must be presented by a member in the form of a written motion, and submitted to the President, and of course members are strongly encouraged to submit motions in advance of the meeting. Back to you, Amy.

Amy Blevins: I am pleased to introduce Linné Girouard, AHIP, FMLA, and MLA 's Sergeant at Arms, who will assist us with the counting of the quorum.

Linné Girouard: I'm happy to be back and here to do my annual meeting duty. As you know the Sergeant arms is responsible for monitoring the logins and the counting of the membership [for this meeting]. We need 200 members for a quorum. You were counted as you logged in with your email address, and your name was recorded. So at this moment the count is 228. We have a quorum.

Amy Blevins: Fantastic! Thank you, Linné. I will now call our meeting to order.

I would now like to welcome Tamara M. Nelson, AHIP, MLA Secretary. Hi, Tamara!

Tamara M. Nelson: Hello! Hi, Amy and fellow MLA members, great to see you all again! I'm excited to be joining today again as secretary, and as MLA Secretary, I get to review board minutes. I also get to present the agenda for the 2024 business meeting which you can now see on your screen. It's also available on MLANET; we've done the first three items already.

And now I will turn it back to Amy.

Amy Blevins: Thank you. Chris, Linné, and Tamara, please don't run away anywhere, because we may need to call on you.

Now please welcome Kevin Baliozian, MLA's Executive Director. Hi, Kevin.

Kevin Baliozian: Hello, Amy! How are you? So it's great to be here, and you know my first duty here is to have the honor to introduce the 2023–2024 current MLA Board Directors wonderful group of leaders. And certainly this year has been exceptional, and they have steered us through the post pandemic years and other issues of global impact.

So thank you very much to Amy Blevins, AHIP, President; Brenda Linares President-elect; Shannon Jones, Immediate Past President; Tony Nguyen, Treasurer; Tamara Nelson, Secretary; and Tara Douglas Williams, Emily Hurst DeDe Rios, Jana Lawrence, and Keith Pickett. I also serve as a non-voting, ex-officio member. Here are the pictures of your board members, and by now you should be able to match the names to the photos. Back to you, Amy.

Amy Blevins: Thank you, Kevin. Now please welcome Tony Nguyen, AHIP, your MLA Treasurer, who will spend the next few minutes regaling us with an update on our finances. Tony, welcome to our business meeting. It's always great to see you, and I heard that you are dressed up just for us today. Is that true?

Tony Nguyen: Sure, we'll go with that. Hello everyone.

As your treasurer, I share the financial stewardship of our association with Kevin, MLA's executive director, and rely on the insights and review of the Finance Committee to ensure the Board of Directors can exercise its duty of care.

The Finance Committee was very busy this past year. We

- reviewed the budgets and financials prepared by the MLA staff,
- worked with independent auditors to ensure our association's compliance and best practice,
- set MLA's investment strategy with MLA's Independent Financial Advisor,
- examined Key MLA pricing models,
- analyzed contract terms with MLA's Management Company, MCI USA,
- ensured the continued financial sustainability of MLA and our pathway post pandemic,
- reviewed and approved funding requests from caucuses and domain hubs in collaboration with representatives of the Community Council, and
- communicated with members through MLAConnect and financial discussions during the MLA Presidential update earlier this year.

I am grateful to have been supported in my role as treasurer by an experienced group of colleagues. Please take some time to thank the members of the Finance Committee.

- Dr. Shannon D Jones, AHIP, FMLA, MLA's Immediate Past President
- Melissa De Santis, AHIP, an MLA member-at-large
- Andy Hickner, a member of the board
- myself
- Kevin Baliozian, MLA's Executive Director
- Kristie Hammill, MLA's Director of Finance

Let's start by reviewing financial numbers. Yay. [slide]

- 2019 was the last year prior to the start of the global pandemic, so it's useful to include it for comparative purposes.
- 2019 through 2022 numbers have been audited.
- 2023 numbers are pre-audit, so the numbers shown currently may be adjusted later by MLA's audit firm.
- The 2024 budget plan is in the last column to the right.

The top three lines are what we refer to as “operating”: that includes all financial activities except investment revenues and disbursements from the MLA endowment for awards and grants.

- Revenues decreased in 2020 and 2021 because of the loss of the in-person conference, partially recovered in 2022. Revenues are significantly higher in 2023 and in the 2024 budget plan.
- Expenses remain high, because we opted not to cut programs and invest instead. 2024 budgeted expenses are lower than in 2023.
- That in turn, creates a net operating loss for all 6 years, though significantly reduced to nearly breaking even in the 2024B budget plan.
- The non-operating net margin is the difference between the financial revenue of our (endowment and reserves) and the disbursements from the endowment fund. As you can see, MLA had excellent financial performance from 2019 to 2021, more than offsetting the operating losses in those years.
- The financial markets were down in 2022, so the non-operating loss exacerbates the operating loss rather than offsetting it for that year.
- In 2023, the financial markets were back up, with a gain that more than offset our operational loss.
- When you add operational with non-operational, you get to the net change in assets. In the 2019 to 2023 period, the total drop in net assets was $622,000.
- Though this is a large number, MLA's financial strength is more than able to absorb this extraordinary financial disruption due to the pandemic, and we are set for rebuilding net assets in the coming years.

The 6-year operating revenue graph is an exciting slide. We not only reversed the decrease in revenues related to the pandemic, we are breaking records in revenues, now above $3M.

As a reminder, operating revenues are the total of all MLA revenues excluding revenues from investments. Raising operating revenues is the primary measure of growth. Growth is essential to long-term sustainability. Growth was also the strategy set by the board in 2017 and again in 2020 and 2021 during the pandemic.

Where does that revenue growth come from?

- Increase in pricing in line with inflation
- Increase in the volume of purchases from members, as MLA offers more relevant programing
- Increase in the number of individuals participating in MLA programs
- Increase in the number of non-member customers
- New revenues, also known as “revenue diversification”

This pie chart illustrates the diversification of 2023 revenues. You can see that:

- A successful annual conference remains essential to MLA revenues, just under 50%. It is typical of professional associations like MLA.
- Membership contributes only 20% of total revenues.
- Continuing education, credentialing, and specializations are on a steady growth path, at 16%.
- EFTS, the MLA-owned billing platform for DOCLINE transactions, contributes 9% of revenues. It provides an essential service to libraries AND contributes to MLA's bottom line. *This in turn reduces the pressure on dues and conference price increases*.
- Note that vendors contributed 26% of 2023 revenues, which is *significantly lower* than the 35% pre-pandemic. Unfortunately, we expect this trend to continue.

This graph [slide] illustrates the 6-year variations in operating expenses.

Post pandemic inflationary pressures are significant, especially with labor. Labor costs include the cost of the MLA staff and the cost of indirect services at the annual conference, such as AV and hospitality.

In 2024, we reduced the MLA staff team by 1.25 FTEs which explains why total costs are going down.

We have an objective to increase our operating margin by $200k through continued cost management and new revenues.

MLA's turnaround is a result of a multi-year strategy. The bottom line is:

- MLA has a long-term strategy to balance revenues and expenses, and rebuild net assets. Revenue growth remains essential.
- Regarding MLA investments, even taking into account the large $523,000 loss in 2022, the average annual gain over the last 5 years is $207,000 per year. That gives us a small cushion and expands our path for long-term transformation.
- Our cash position is strong. We also applied for and received a $500,000 COVID-19 Economic Injury Disaster Loan in 2021 at a 2.75% fixed interest rate. That increased our flexibility. We are paying it down monthly, and can reimburse it at any time over the 25 years of its term. The last time we used funds from our reserves was in July 2022.
- Our net assets are at $3M as of December 31, 2023. That's one year of expenses and is considered a strong benchmark.

In summary, I am happy to report that the state of MLA finances remains strong. I will add that we could not have gotten this far without the support of MLA's finance committee, the MLA board and staff, and MCI.

Amy Blevins: Thank you, Tony. The board is grateful for your diligent stewardship of the entire financial team, and for those of you who may have missed it. Tony also was kind enough to write an *MLAConnect* article on finances. So if you want to learn more about the way that MLA operates financially, you can go to that link within the chat.

The next order of business is the Executive Director's report, and I believe Kevin is going to talk to us about the new platform, right?

Kevin Baliozian: The one that will solve all our problems.

Amy Blevins: That's right, Kevin. Your words, not mine this time.

Kevin Baliozian: So I really truly recommend everybody looking at the headquarters report, and, in fact, all of the annual reports, and we'll have a slide and a link on that. They are full of great data and activities on programs. On the screen is just a fun selection of some of that data. We have metrics on all of the different levels of participation in all of the programs of MLA. This is a time where we report the number of members and other interesting participation information we have as of May 30 of this year.

[We have] 2,339 members; we're on an annual year, January one to December 31. So we do gain more members [past May]. Last year we had 2,346 at the same time. So essentially the same number, 7 less. Can we say it's even Amy. I think we can. So no change in in in membership counts.

A few things are interesting here, is that twice as many individuals participate in MLA than our members. That's the customer number. That's the number of active individuals who are taking part in things that we do that aren't counted in the 2,339 number. So that's a, that's a big piece. We have a lot of people who are using MLA courses, come to conferences, do various things, but who don't necessarily join as members.

On the right. You see the participation of caucuses, 76% of MLA members participate in caucuses; those who participate in caucuses join 5.2 caucuses on average, and this number is growing each year since we removed the fees to join sections at the time, right when we everything became caucuses. And below, you can see the data for the MLA conference, it was very successful both in person and virtually.

And this next slide here actually shows in blue the in-person attendance of the conference; in orange, the virtual attendance. Clearly, 2020 and 2021 was the pandemic and you can see a gradual return to the in person conference from 2022 through 2024, with still a significant 22 or so percent of individuals attending the conference virtually.

Amy mentioned the board discussions and strategic goal discussions. And the AI [Imperative] has been officially approved as a strategic goal, and you can see the bullets there of what it is looking to achieve at a very high level.

There are 3 areas that the Board discussed in its main meetings. You mentioned Amy, the leadership at all levels. You mentioned expanding audiences and advocacy with the accrediting bodies was also something that the board MLA could focus its limited resources better. In influencing accrediting bodies, in appreciating the value of health information professionals.

So now the technology sneak peek. These are a couple of screenshots. I hope you speak Latin because some of the text in those were in wireframes, a normal live site that we're putting together. I understand that AI does not understand Latin as a translation language. So we will have things in English that should make things a little easier. Because we are making the live site as we speak. These are to illustrate the 4 areas that we are intentionally looking to improve and how we're doing it.

The first one is clearly improving your user experience. And that means a whole bunch of things which we define on the right. And I'm not going to read them, but improving the experience of customers and members through all of those different things is critical. The second is to improve the user engagement. Of course that's connected with the experience. But the engagement goes further. And there are some very specific items related to how do we keep people engaged. How do we get a new member engaged? How do we get people to participate through reminders to things, etc.?

Staff productivity is a very big piece of what we're looking to improve. We do have pressures and are trying to do more with less. And that's impossible to do without high productivity. We have identified all of the areas that we consider are too manual that could benefit from automation, so that the staff can focus on non-menial tasks and on value building. And we need to improve the return on investment. We define that in 2 ways. One is for you as the individual participating, that you have a much better return on your presence on the website and what your membership provides. And of course, the return on investment on the association that we achieve through productivity gains, and through having more people participate and be more engaged.

Last is the timeline. I don't expect you to memorize this, and we are going to be communicating next week, but because of our limited abilities to do 10 million things at the same time, we are going to switch the current site off on the end of the day of June 26 and move to the new site. But the logins will be disabled for a longer period. So all of the activities that people do such as take courses, renew memberships, join participating caucuses. Those types of things will be unavailable. For 10 days. We realize that that's rather large. It does include weekends and the 4th of July weekend. So I recommend you have a barbecue while we continue to work. But we will be doing this in stages.

Obviously, there won't be any dark time for the public side of our website. You'll be able to see the website, but you won't be able to log in for a few days.

It may be earlier than Monday, the 8th but we are giving ourselves enough time. There's a lot of data we're transitioning and we want to make sure that we don't lose anything. Your applications, your history, your AHIP history, all those things and that takes time to get the data. And then, if the data is not in, the things don't work quite right. So it's not just a website transition. It's all our database transitions, including the LMS and all of the satellite databases all being changed at the same time.

There you go. It will solve all our problems. We hope! Back to you.

Amy Blevins: Thank you, Kevin.

The next order of business is to present the 2023-2024 annual report. Our MLA annual reports are valuable for all of us to read, so you may discover parts of MLA you did not know, or incredible things that your colleagues have enabled. Take time to read them, even if it's just the executive summaries, although I'm sure those are so riveting you won't be able to stop yourself from reading the full text. Those reports show the immense diversity of our communities and our programs. They are also part of MLA's archives. So if your name is in there, you are officially recorded for posterity. So basically, MLA famous. This slide shows all of the MLA components that contribute to this comprehensive document. The reports are available on MLANET, along with reports from past years.

Those who are nominated each year as potential members of the MLA Board of Directors are selected by virtue of their experience and reputation to serve the Association, but few can imagine beforehand the level of commitment that election to the Board requires—and it seemed like even more so this year. Directors who have completed their term on the MLA Board have served our association with enthusiasm, dedication, grace, good humor, and perseverance. The Association and the Board of Directors express our appreciation and recognize each of you today for the extraordinary work and thoughtful leadership that you have provided during your term of service. Thank you for a job well done.

I'd like to recognize specifically Tara Douglas Williams and Jana C. Lawrence, AHIP, FMLA. It's been wonderful serving with you on the board. I also want to express my sincere gratitude to Shannon D. Jones, AHIP, FMLA, MLA's 2022-2023 President. And I want you all to know that Shannon has been on the Board of Directors for 6 long years, although I'm sure it flew by for her. Shannon, I'm pleased to thank you on behalf of the MLA. Membership for your leadership. During your Presidential year we were impressed by all the activities you undertook during your year as President, and this year, as Immediate Past President. You have been a calm and steady presence, as you encouraged our MLA community to strive for work-life balance during the post Covid pandemic. Your MLA Be Well series has gifted us with refreshment, emotional strength, and resilience. I know I have greatly benefited from participating in these sessions and share my gratitude to you for spearheading this program, which, as I mentioned earlier, is now an official MLA program with its own committee.

Shannon, long before and throughout your presidency you have championed MLA's vision to foster excellence and commitment to diversity, equity, and inclusion in professional practice, leadership of health sciences libraries, and information professionals. We can all learn a lesson from your book by reminding ourselves to be more humble, compassionate, empathetic, and have genuine care and concern for all humanity. You have been described as someone who takes chances on people, valuing those others might not see as valuable. This was visibly transparent when you appointed everyone who applied for a 2022-2023 committee.

And finally, we would be remiss if we overlooked some of the unique challenges you faced during your Presidential year, completing your doctoral degree, Dr. Shannon Jones, and rebuilding your library, all while serving as the director of your library and serving your community through your involvement with the Girl Scouts. Congratulations on a job well done, and thank you.

Shannon D. Jones: So, Amy? Wow! Thank you so much for your kind words it. It was truly a honor and a privilege to be trusted by our MLA members to represent and to serve them over the last six years, so to speak, and especially during the three for the Presidential term.

I am so proud of you. I am very confident that you will do a wonderful job as past President. And thanks to everyone who supported me throughout the year. And I did, I brought the gavel out because I've been waiting to receive this thing for 3 years, because it's so beautiful and I will cherish it. I had to work real hard to get it home, because Homeland Security said it was a weapon, and so I had to work hard to get it from Portland to here, and so I will cherish it forever. I also want to thank Amy for the beautiful cake set that you sent as a parting gift. if you were at the conference last year, you probably saw the interaction between Amy and I with the cake, and there's a picture circulating on the MLA website with Amy pointing a knife at me and the picture caption should say, “Shannon, either get your stuff together, or I'm going to cut you with this knife.” And so she sent me the most beautiful knife, and it's engraved with MLA on it, but every time I cut a slice of cake I'll think about you, Amy, and I'll make sure that I always get myself together.

But no, thank you all so much, and thank you again, Amy. Thank you to Brenda. I'm looking forward to supporting Brenda from the audience next year. Thank you all to the

MLA Headquarters staff, who also make the job of being the President so worthwhile, but also, they allow us to be able to do it because they really support us in the background. So thank you all so much, and I will hand it back to Amy.

Amy Blevins: Thank you, Shannon, and I will say I knew we had it under control that whole time, and people doubted us because it was a heavy cutting board under that cake, but we had it. We were not going to drop that cake! I was gesturing, you know, triumphantly at how well we were doing by managing the cake.

Shannon D. Jones: That's not what the picture tells. [Laughter]

Amy Blevins: Can you imagine if we dropped that cake? Oh, my goodness! So to get back to business!

The MLA 2024 election was conducted from March 4 to March 18 of 2024. Voting statistics can be seen on your screen, and election results were announced on March 28, 2024 in *MLAConnect*. Following are the election results.

Nine individuals were elected for a one-year term to the nominating committee. Their names appear on your screen:

- Nell Aronoff
- Lindsay E. Blake, AHIP
- Elisa Cortez, AHIP
- Diana Delgado, AHIP
- Kenya Iverson
- John Mokonyama, AHIP
- Joey Nicholson
- Michele L. Mason-Coles
- Shannon Shaunette Glover, AHIP

Heather N. Holmes, AHIP was elected to serve as president-elect. Welcome back to the board, Heather. Heather actually served on the board from 2020 to 2023, but it seems she could not get enough. Congratulations also to Kate Flewelling, AHIP and Liz Kellermeyer AHIP, who were each elected by the membership for a three-year term to the MLA Board of Directors.

And now it's time for my year as MLA President to come to a close. It's my honor and pleasure to introduce your 2024-2025 President, Brenda M. Linares AHIP, Associate Dean of Library Services at UMKC Libraries, University of Missouri-Kansas City, Missouri. Brenda is dedicated to fostering an inclusive and innovative environment that supports the academic and research needs of the university community. Her leadership emphasizes collaboration and continuous improvement, ensuring that KC Libraries remain a vital resource for students, faculty and staff.

Brenda has been actively involved in various capacities within MLA at the national level. She's been a member since 2008 and served on the board of directors from 2020 through 2023. Her contributions include participating in the continuing Education Committee, the *JMLA* Editorial Board and several juries, such as the Lucretia W. McClure Excellence in Education Award Jury, and the MLA Scholarship for Minority Students Jury.

Brenda is also a co-founder and co-convener of the Latinx Caucus (which just celebrated their 10-year anniversary!) and has been actively involved in the Leadership and Management Caucus.

In addition to her MLA activities, Brenda has engaged with other professional organizations such as Reforma and the Association of North Carolina Health and Science Libraries. Her contributions to the field have been recognized through several awards, including the NNLM R3 Emerging Leader Award in 2021 and the MLA President's Award in 2020. Brenda's work has also earned accolades such as the MCMLA Annual Conference Viewers' Choice Award Winner Poster in 2020 and the Majors/MLA Chapter Project of the Year for the Mid-Atlantic Chapter Diversity Task Force in 2018. These honors reflect Brenda's commitment to advancing library science and supporting diversity and inclusion within the profession.

On a personal note, I've known Brenda for a long time, and she is one of the kindest and most welcoming people I've met at MLA. It's been lovely to see her and Emily welcome Clara into the world and see them introduce her to MLA, and I've enjoyed serving on the Executive Board of Directors with her. I know that she will do wonderful things during her upcoming presidential year.

And now I am thrilled to pass the gavel to my distinguished colleague Brenda M. Linares, AHIP.

Brenda, are you ready for me to throw the gavel to you? Brenda Linares: Got it. Amy Blevins: Oh, good catch. I was worried you weren't going to!

Brenda Linares: I got it. That was a good throw.

Amy Blevins: I'm thrilled to pass the gavel to my distinguished colleague, Brenda M. Linares.

Brenda Linares: Amy, on behalf of the membership and headquarters staff, thank you for your strong leadership and countless hours of service this past year. I felt so fortunate to get to know you as a colleague, as a friend, and work closely with you on the board last year, and, as you can see, your cheerful demeanor and steady presence have encouraged a lot of our MLA community to make sure they pursue a work and life balance in the post pandemic era. We all can learn a lot of lessons from you and your patience.

You kindly guided us through that extended four-hour 2023 business meeting, encouraging all our members to make sure that they voiced their opinion if they had something to say, because our membership voices matter. And so you made certain that everybody was heard, and that we listened to them, and were able to accomplish all that we needed to do in making changes to the bylaws. So thank you for that, and your effective leadership facilitated the bylaws vote which successfully passed last Summer.

Knowing that there may not be enough opportunities for people to learn how to conduct educational research and assessment, you spearheaded the creation of a joint MLA/AAHSL Education Assessment Task Force. And we look forward to seeing what they do and seeing how that's going to make a big difference in our profession. And so also, as we learn more about AI, and how important it is to our profession, you were also leading voice in effectively guiding us, in making sure that we had that as part of our strategic plan and goal. And I look forward to hearing how that working group is going to do that. And so we are very pleased to have your leadership, your proactive, engaging leadership; how you led our board and your commitment, your energy, your positive attitude. Your commitment to the members making sure that we all had a voice in every discussion, not just with the business meetings or last year, but also in the MLA Board meetings that we've had. So we definitely enjoy working with you and having that encouragement when we had those meetings.

so, as part of your service to the MLA Board, I know that we at the meeting in May were delighted to present you with a silver cup as a token of our appreciation. So if you can show it to our members so they can see it, and they can see how grateful we are for your service. And I know that your service is not done yet, because I look forward to working with you as past president in the incoming year as a as we as I as I work in the MLA Board. So I look forward to doing that. A I hope that you would display that cup, probably in your office, which symbolizes a year of when you did a lot of great things for MLA. So thank you again for your leadership. I really enjoy working with you. I'm so glad to call you a colleague, a good friend, and I know that I have someone out there to reach out if I have any questions. Now that I'm President of MLA. And the same thing with Shannon. So thank you so much for your leadership.

Amy Blevins: Thank you, Brenda, and I'm very excited to have my silver MLA President's Cup in my office. And thank you so much to all of our MLA community who made this possible. It's been wonderful working with all of you, and I'm going to turn it over to Brenda.

Brenda Linares: MLA members. I'm very pleased to present you the 2024-2025 Board of Directors. These are the members. Congratulations to all of the directors on being elected.

If our names are not yet familiar to you, especially those who were elected this year, I hope that you can go up and look at their names and their backgrounds, and be able to get to know them better. And here's our photos. So you can have a picture of what we look like and know that hopefully, you'll get to see many of us in future forums, and committee or caucus meetings or chapter meetings, and be able to reach out to us if you have any questions or ideas that you want to provide about MLA as a whole, because again, we want to hear your voices and your ideas. And then here's our group photo o the Board of Directors with the outgoing and incoming members that we took in Portland last month. It feels like it's so long ago. But here's your leadership group. And I really have enjoyed working with all of them and look forward to working in this new year. So now I have the honor to finish the remaining item of business before we adjourn this business meeting.

So next are resolutions. We have no resolutions at this time, so I will move to new business. I'm very grateful to see that there is no new business in our agenda. So let's welcome again our board secretary, Tamara Nelson, for the final item of business today.

Tamara M. Nelson: Thank you, Brenda, and congratulations on your presidency. I'm so excited to be working with you. I moved to adjourn the 2024 MLA annual business meeting.

Brenda Linares: Thank you. It has been moved to adjourn. I propose to approve the motion by unanimous consent. Any member may object, in which case we will call for discussion on the vote. Please raise your hand if you have any objection.

Since there's no objection, the motion is passed and the meeting is adjourned. Thank you for attending the MLA Business Meeting. But wait, there's the last item to go through, and that is my space right now to share with you my presidential address. I'm hoping that you all can see my slides… Okay, great.

Well, thank you so much. To start with, I'd definitely like to thank all of the people that voted for me to have this opportunity to be your MLA President. I'm really excited about this opportunity. I'm looking forward to working with many of you. But before I get started on some of those things that I would like to do, as I'm working with MLA Board is some of you know me, but some of you might not. And so here's actually a picture of me that I was able to find from my first MLA that I attended in 2008 in Chicago, and it's interesting to think about it, that is, connections bridging the gap and I remember how excited about the conference I was because it was my first MLA.

And just to think about where I am right now, many years later, it feels like such an honor. And I'm very humbly accepting this responsibility that you all have been telling me.

So here's what you are going to be hearing about me today. Many of you know me, but some of you might not. And so here I'm going to tell you who Brenda is, share with you some of the values that I intel with my professional life. But I definitely want to make sure you know that we're good. What are the things that we're going to be working on together? And then, two things that I would like to work on that are part of our core values and our strategic planning as a whole, and something that I brought up when I was running for President two years ago.

So who is Brenda Linares? Well, as many of you know, I'm a Latina woman. But many of you might not know that my family and I are from a small country in Central America, Guatemala. Some people might not even know where that is located. So here's a map of that beautiful country where I was born. I'm very proud of that culture, very proud being “chapina” we call ourselves. That's how some of my personality has been influenced by where I was born and where my family is from. Many of you might know it, because it has a very famous location, Tikal, which is the Mayan ruins; or Antigua, where a lot of people tend to go for Holy Week because they have this beautiful procession. I haven't been to it, but I hope to go one day, and I'm actually part of the country that borders El Salvador. So I'm actually from part of the southeast area of the country itself. I'm very proud of that heritage. So that's where my family is from.

Some of you might know, some famous people that you might know from Guatemala are Rigoberta Menchú, who got the Nobel Peace Prize for her work with peace Guatemala with the Mayan community. And there, so it was really great that she worked on that. So I'm glad to have a strong woman from Guatemala doing that. And then, of course, we claim Oscar Isaac, because his mom was from Guatemala. So I'm really proud that he's someone that's well known from Guatemala, and if you're a Star Wars fan like I am, you might have seen Star Wars episode 4 A New Hope, and you can see the ruins there. So if you go back to the movie, you can see that that's basically they went to Guatemala and that's basically the minor ones that I'll just show you in the previous slide. So that's really cool a little bit up with the Guatemalan background.

So I said I was born in Guatemala. I came to the United States when I was 10 years old, and my sister was 7 years old, and so my parents brought us to this country for a better future, more opportunities for, for our family, and we were very grateful for that. But it was also a lot of change that we had to go through because it was a new culture, a new country, a new language. And I had gone to school in Guatemala, and I was at the top of my class. And when you come to another country with a new language, you need to work hard to reach what you need to do. I'm very grateful for my parents wanting my sister and I to have a better future in our lives.

Another place that I call home and dear to my heart is California, because I grew up there, and I have a little star there because I grew up in the San Fernando Valley. And some of you that are from Cali know where that is, it's in Los Angeles County. So I'm really proud of being a Valley girl. I have a picture here of myself at the sixth-grade graduation with Dr. Deborah Neal. She, if you're thinking about one of the things that I'm really fortunate is to have mentors and people that throughout my life have influenced me … and guided me to reach for the stars and never give up, no matter what people said or what got in your way.

And Dr. Neal is one of the people that I'm always grateful for. She knew that, you know, when I came to this country that I was learning this second language. She knew that I was bright, she knew that I was smart, and she told my parents, “You know what, Brenda is a bright student, she has a bright future, and we'll put her in the right direction with the right resources that she needs.”

And so she helped my parents fill out forms so I can go and attend honor classes, go to a magnet unit high and magnet high school and the rest as you said is history. So Dr. Neal was very influential in seeing that potential in me, as this little immigrant girl that was trying to fit into this new country, this new culture. And she has been such a great influence in my life that I'm still in touch with her. She came to my graduation from college when I got my master's in library science. I'm very fortunate that she has even met my family. And, so she's still part of my life, because she played a, a key part in who I am and leading me in that path of success. So Dr. Neal, you know, thank you for that.

Then, of course, I did, my undergraduate, Cal. State Northridge—again, Cali girl, and here's Karin Durán. She was my supervisor in the library. And I didn't think I was going to go to library school or be a librarian, but I enjoyed working in the library, and when it was time for me to think about a different career path, Karin was the one that told me, “Why don't you think about library school? You were so great at helping people, you enjoyed what you were doing, you should think about that—I can see a lot of potential in that.”

And so I followed her advice and I went to library school, and everything worked out so well. Because that summer I met with her, she told me about an information session at UCLA, I went, I took my GRE that fall, and early next year I was accepted to UCLA. I was one of the ALA spectrum scholars and that was able to help me with my library degree.

Karin Durán is always in my heart. Unfortunately she passed away in 2010. But I always think about her because she's the one that told me, “You should be a librarian.”

So, went to UCLA. And here's, you know, the Louise Darling Biomedical Library is also dear to my heart because on my last semester of library school I did an internship there and that's when I found my calling as a health sciences librarian. And because I knew that once I worked there, I liked helping people, I liked that I didn't have to have an MD to help researchers do their work, to help physicians work on patients, to help the community. There were so many things that you could do as a medical librarian that I really fell in love with that profession. And here's also where I learned about the NLM Associate fellowship—and that, of course, takes me to that year in my life.

I was really proud to be an associate fellow and I met so many great people. There were seven of us in that class who I still am in touch with. They came to my wedding. They're some of my best friends, and of course, when I tell everyone my story I always tell them that NLM changed my life professionally, but also personally, because that's where I was when I met my beautiful wife, Emily. And so I'm always grateful for the experience and the memories and the relationships that I built there. As you can see we were fortunate that Dr. Lindburg actually invited us to his home and we were very fortunate to have a group picture there, and I have so many great memories of that year. That led to another foundation of me, of being a health sciences librarian.

So I'm telling you all this, so you know how my road has been and how my path has come to where I am right now. I was fortunate after the fellowship to have had experiences in different libraries that built my skills, my experiences; I was able to get grants to do outreach projects, do research and collaborate with a lot of colleagues.

I worked at a Louis Calder Memorial Library in Florida. I had the opportunity to work as a school of nursing liaison in UNC Chapel Hill, in North Carolina; I had the opportunity to work as a school of nursing liaison at KU MED and currently in my job, the University of Missouri, Kansas City, I have the privilege of being in administration with the academic library but also working with the health sciences. And so all of those experiences, through my journey, as an immigrant little girl, through my schooling, my jobs, have made me who I am and where I am right now. Because I think through all of them, I've learned so much in my different experiences and that's my story that I'm here to tell.

But of course, you know, there's people that I met through MLA, through leadership training, through colleagues. And I'm really proud that I've been blessed to have people in my life that have seen that potential in me, and have spent the time to advise me, mentor me. And if I had issues about any career decision to make, I had those people to ask them questions about it because they were there for me in many different ways. And so I'm very blessed to have had people in every different level of the health sciences profession that I can call my colleagues, my mentors, and my friends.

And of course there's so many other people that I've met through the years. I have so many pictures that I couldn't put a lot of them in here, so here's some of them. But I'm so grateful that I've learned so much from my colleagues in the conferences. When we presented, see classes, we did research together. We even have written, I've had the opportunity to read papers and present posters and even book chapters. So MLA is about relationships, collaborations and working together. And I definitely have had the opportunity to have so many people that I learned from as I've gone through my career. There's so many, you know, people that I'm grateful to have in my life as colleagues and friends and to learn from, because I think that's the important thing: that we know that we can all learn from each other, from our experiences, from our differences and from our similarities, because it's really important.

And of course, one of the things that Amy mentioned I have been involved with MLA since I started in 2,008 in different caucuses, committees, juries, the MLA Board. But one of the things that I'm really proud of is being one of the co-founders of the Latinx caucus. It was Co. It was called the Latino SIG when we started, and just to see this group, and how far we've come in the last 10 years. It makes me very proud of the work we've done in the past 10 years, and I look forward to seeing all the work that we are. We're going to be doing in the future. And so it's great to see this community. It's great to see those librarians getting together, and I was really happy that in this conference last month I got a chance to meet a lot of new librarians, reaching out and saying, “You know, I have been intimidated to be in MLA. But meeting you, or seeing this group, or seeing the other people, I know I can do it.”

And I'm hoping that my story tells those people that they can do whatever they can. The sky's the limit, and there's a lot of resources available for them from colleagues and from the organization. So I'm really proud of that.

And of course you know that my family is really important and precious, and I couldn't be here without their support, their love, and their sacrifices. I think, I always tell people, that my work ethic comes from my dad. He was a truck driver for most of his life, and so he always, both of my parents have been caring, and had told me that I can do anything as long as I work hard. So I really thank them for the support. My sister, you know she's definitely my best friend. We're close, and of course I can't, you know, say enough about Emily, my wife, my colleague, who has helped me through my career—we both, you know, being involved with MLA.

She did a great job at the conference last month, so that has helped me a lot to keep going in my goals, in my profession, when I know that there have been instances that there have been obstacles or people that have tried to block that path. But I'm glad that all this, that I'm showing you, has been a great support system that I have throughout my life. And so this is what makes Brenda Linares, and of course Clara our daughter you know, our miracle baby that I'm so blessed to have. And Mickey our puppy, he's precious, so I value that a lot, and I'm so grateful to have them in my life.

I want to thank my staff, of course, because without them and their support, I wouldn't be taking on this role. So definitely, I'm really grateful to have a library supporting me and my staff for sure. And as I go through my speech I can never forget the people that have, you know, built that path for us to be here, or for me to be here. You know Beverly, the first African American president. I have reached out to her for advice. She's a great mentor, a great friend, and I'm so grateful to have her in my life because she has been a great mentor when I've had tough decisions to make so I'm very grateful that I had her, and I'm trying to follow her footsteps. And of course, you know, Naomi Cordero Broering, our first Latinx. President.

I stand here because of everybody else that has gone through being president of the organization. But I know that I really look up to these two people because they were the firsts and you know, I'm continuing to follow the path. And I know that I'm not going to be the last one. So I want to thank them for the hard work that they've done in the organization and for leading the way. I'm so glad that I got the chance to have both of them and know them.

And of course, working in the board. Thank you, Amy, for your leadership. Like I said, I'm going to do my best to lead my own way. But I definitely know that there's a lot of great foundation that the past [MLA] presidents have led for me to follow. And I look forward to working with the board as I start my new year, so now that you know who I am. I call myself the first immigrant Latina MLA President with a lot of honor because I see that as one of those America dreams that I'm here, as a success story, that anything is possible. If you work hard and are dedicated, and follow your dreams, and don't let anybody get in your way.

So, as the first immigrant Latina MLA President, here are some of the values that I mentioned before. I value teamwork, collaboration, inclusivity, and innovation. And I hope that that's what you'll see in my presidency this new year, because we can't do anything alone. We have to work together, and everybody has their own unique skills and experiences that make the team work. Everybody has to be part of that discussion at that table, and that's why I value inclusivity, because my goal is to not leave anybody behind.

And of course, as we think about the future in the next 125 years, innovation is very important. The profession, you know, what we do, the foundation is the same, but how we do it changes, and we need to be proactive. That's one of the things that we're doing as an organization which I'm proud of.

So what are we doing in the next year and in the future as part of my presidency?

So, of course, you heard some of those things that we've done already. I'm definitely going to be more involved with the AI initiative. I know that's really important. That's a thing that is not going away. And so we need to be proactive in making sure that we know what our users are doing we know what our colleagues are doing and that we keep ourselves relevant. So that's definitely going to be part of the next year.

Leadership. You also heard that mentioned in the previous speeches from Amy and from Kevin. We can be leaders from any part of our institution. You don't have to be a director to be a leader, to make a change, to make a difference. We know that we all have that skill and those talents. And so what do we want to do? We want to make sure we as an organization have the resources and the training you need for that to happen. So you're going to be hearing more about that. And as we're talking with the Board, with the staff, with our members, we want to make sure we have the resources and the training. You need to be the leader you need to be in your institution because a lot of times there's budget cuts. There's, you know, organizations trying to not let you have a voice.

So how can we, as an organization, provide that training? You need to make sure that you have the resources to do your job from any role you're doing in your institution. Because we are leaders. We all can be mentors. We all can be people that influence other people and so be in the lookout for that. So that's another of the things that we want to focus on, advocacy.

We know that's important in our role health literacy, information—some of that is being threatened by misinformation, misguided facts. And so I think as an organization, we want to work together with our members, with our chapters. And, like mentioned earlier, with other organizations, to make sure that we're advocating for each other, that we're not alone, and that our profession is heard, and we support the people that need us, and our users, our communities. And so that's one of the things that I want us to work on together in the incoming year. And again—we're going to do it together.

I'm going to put this here because it's been heard a lot. And so as part of this new incoming year. I'm probably going to be part of that communication and part of sharing some of the training about the new website and how great it is and how it's going to solve with our problems. But being serious about it, we hope really, that this new website will be more user friendly, and we'll be able to provide a tool for us to be able to collaborate better. And so I look forward to that transition and also getting feedback from all of you about the website itself and how it is solving your problems, or how we can even make it better, so that it's better as we do our work with MLA. So you've heard about those things.

So here are two key initiatives that I call to share with you that it's part of our core values. But it's also back to one of the things that some of the things that I mentioned when I ran for President of MLA. And I hope that we work on this together.

I wanted to go back to the MLA Diversity and Inclusion Task Force and the 2019 Survey report and looking at our membership. Now keep in mind those that's in 2019 things could have changed a bit, you know, now in 2024. But something to keep in mind is we really need to think about the membership of our organization and how it's changing, how the people that we work with and the people that we provide services to, it's also different. And how I mentioned before, being innovative, being creative, being collaborative and having team work. If you have different ideas and different people that have diverse backgrounds, diverse skills, it creates a better future for the profession itself.

And so what I'm what I'm trying to see with some of these statistics is thinking about, how do we want to see our organization moving forward. We want to have more of that inclusivity. We want to see our membership as us. It's aging, too. How can we make it more inclusive and get more members involved?

We also think about the composition of it, too, you know. Being different kinds of sexual identities, sexual expressions. How can we provide support to our members? Knowing that some of the states that we are part of, unfortunately taking some of those rights away. So we definitely have to think about that. And how can we provide support to our members?

And of course, the different abilities. We definitely, you know, when we think about diversity, equity, and inclusion, there's so many things that that includes—it's not just race and ethnicity, there's so many other things. And so my commitment to you is saying we need to think about our membership together, and that there's so many concepts that that includes.

So what is on me?

I think, as part of, as you see, the statistics and how that is really important, and one of the things that we want to do. This was also part of the some of the conversations we had with the MLA Board, we know that there's a bit of a timeline with some of the DEI committee timelines within MLA and MLA chapters. I always find myself really proud of being part of the MAC [Mid-Atlantic] MLA Chapter, because we were the first chapter that started a DEI taskforce in 2015, and I was fortunate to be the chair of that task force because we wanted to see what the membership was. And based on the work we did, as you can see, we became a committee and we got the chapter award for that work that we did with our members, and that chapter, in 2018.

And then, as you saw, MLA created their DEI Taskforce in 2016-2017. Then it became a DEI committee in 2020, and I'm really proud to see that in 2024 we have a lot of chapters that have their own version of the diversity, inclusion, and equity type of committees, and I feel fortunate to have been part of three of those. I was part of MAC as a co-chair of the task force, and then when I moved to Kansas, I was part of MC-MLA [Midcontinental Chapter of MLA] and was part of that task force also, based on my experience from the previous chapter. And currently, I'm part of the South Central Chapter working with a group to have that task force become a committee.

Because I think we definitely know that it's important for us to have our organization feel inclusive to our members, but also to the people that we serve and represent, when we go into our work. And so there's a lot of work that we can do there. And why? Why do it separately? You know MLA is doing so many great things with a committee nationwide. And I know that the chapters are doing great things where they're programming and what's happening in their States.

I reached out, you know, to Michael about the MLA DEI Committee, and how we wanted to work together, and reaching out to the other chapters and saying, “Hey, what can we do and work together, you know, in terms of advocacy, communicating the resources we have, MLA as a whole organization? What kind of resources do our members need for them to have, support and information and working together so that we are not reinventing the wheel and the chapters feel supported by the big organization?” And so I look forward to having those conversations with our different MLA chapters and also our big MLA DEI committee, because I think there's a lot of things that we can do together, and a lot of communication that will happen. And so I'm looking forward to that work in the following year.

And then, of course, we think about recruitment and retention because we want to make sure that our organization is retaining the new the new members that we get or staying relevant. We pursue stories of some libraries that feel like you know what I'm not getting, what I need from MLA. I need to go to another organization. Well, what is it that you need? We want to make sure we provide that. That's why we're focusing on AI, that's why we're focusing on leadership. That's why we focus in on inclusivity.

My goal is to hopefully work and do more recruitment and outreach for our organization. I know that some of the committees, such as the Recruitment and Retention Committee, Education Steering Committee, the Medical Education caucus. I know that even the Latinx caucus, AAMLA [African American Medical Alliance], and other groups have had those conversations within their small groups. Well, I want us to have that conversation nationally and say, what can we do to recruit more members into our organization?

Do we need to go to library schools? Do we need to join other organizations like ALA and recruit people to be health sciences librarians. They need to know that “health sciences librarian” is not something to be intimidated by. It's a great opportunity. There's a lot of difference that our profession makes. And also maybe reaching out like Amy and Kevin mentioned earlier, to other professions. We can create relevant resources and training for other professionals to join MLA. I think there's a lot of potential and I look forward to working with many of these committees and caucuses. And if you have other ideas of how can we do more recruitment and outreach to MLA, I look forward to having that conversation because I think there's a lot of potential for that. Because our profession is aging, what we do is hanging, and we need to be proactive and be able to retain and recruit people into our organization.

And what else am I looking forward to in this new year?

I also look forward to meeting many of you that I haven't had a chance to meet on, and so I'm hoping to be able to attend some chapter meetings, either in person or virtually. So please, if you see me, say Hi, or send me a message, because I'm all about building relationships, building community, working together. So I want to meet you and get to know you. And if you have ideas, share them. Send me an email because we're all in this together, and it's a team effort. I cannot do this alone. The MLA board cannot do this alone. We need everybody to do it together, because we have so many skills, so many experiences and resources that we all can combine so that we can, you know, think about the future.

We've had a great 125 years of MLA, we've had our ups and downs. Well, let's think about the next 125 years of what we want to see in terms of our membership, our core values, our profession as a whole. And that requires a lot of work—but not just from the MLA board or myself or the MLA staff. It comes from all of us together.

And know that I'm here as a team player. I'm here as your servant leader. I am here to make a difference as much as I can, but I cannot do that alone. And so I look forward to getting to know a lot of you and working on these initiatives together. You'll be getting more information about that in the incoming year. But please reach out if you have any questions, and so I look forward to working with all of you and for you.

Thank you so much for attending this business meeting. Thank you so much for listening to my presentation, for your vote, for your support, and I look forward to seeing all of you in Pittsburgh, Pennsylvania, for MLA 2025.

Enjoy the rest of your day, and I'm so glad that you were able to stay to hear this presentation. And again, we are all MLA, like Beverly said, and we can make a difference if we work together.

Thank you so much.

## SUPPLEMENTAL FILES

**Appendix A:** MLA '24 Program Session Abstracts

**Appendix B:** MLA '24 Poster Session Abstracts

