# Supplementary material for: 124th Annual Conference, Medical Library Association, Inc., Portland, OR, May 18-21, 2024: 2024 Annual Business Meeting and Presidential Inaugural Address, June 5, 2024
Source: J Med Libr Assoc. 2025 Oct 23;113(4):E1–E28. doi: 10.5195/jmla.2025.2356 (PMC12604072; doi:10.5195/jmla.2025.2356)
Supplement: Supplementary file 1 — Appendix A: MLA '24 Program Session Abstracts [file jmla-113-4-E1-s01.pdf]

Medical Library Association
MLA ’24 Hybrid Conference & Exhibits

Immersion Session, Paper, Lightning Talk, & Symposia Abstracts

MLA ’24

Portland, OR

STRONGER TOGETHER

MAY 18-21 | #MLANET24

Abstracts for contributed content (immersion, paper, lightning talk) sessions are reviewed by members of the Medical Library Association National Program Committee (NPC) and volunteer peer reviewers. Designated NPC members make the final selection of content to be presented at the annual conference. Symposia were selected by MLA special content committees.

Information is as provided by abstract submitters.

IMMERSION SESSIONS

We have sorted content in this section by title in alphabetical order.

An Ally Is an Activist Is an Advocate: An LGBTQIA+ Panel Discussion & Workshop

Track(s): Education, Health Equity & Global Health

David Keddle, Medical Library Services, Director, Woodland Hills Medical Center

Soph Myers-Kelley, Liaison Librarian to the Brody School of Medicine, Laupus Health Sciences Library, East Carolina University

Catherine Lockmiller, Health Science Librarian, Northern Arizona University

Session Overview:
We will have a 30-minute panel with three leaders covering the role that harmful legislation and policy play in impacting LGBTQIA+ people on a local, national, and global scale, how to conduct meaningful outreach (external to your institution) and education (internal to your institution), and taking an expansive view of allyship as active, reflexive, and oriented praxis. We will end with 40-minute small group discussions where we brainstorm, grapple with, and explore scenarios that may impact library workers both inside and outside the workplace.

Instructional Methods:
Participants will learn through group discussion and be asked to help with note-taking and share back. Key readings on a research guide will be shared before the session. Group discussion prompts may also be shared.

Participant Engagement:
Group discussion, role-playing and share back will engage learners.

Session Learning Objectives:

- Expand definition of Q+ activist allyship and set a goal for application to medical librarianship
- Brainstorm possible Q+ allyship events, outreach to politicians, and meaningful ways to have hard conversations.
- Learn how to identify material harms LGBTQIA+ people face and the specific ways that librarians can minimize those harms.

Sponsor(s): LGBTQIA+ Caucus

Bringing the New MLA Research Agenda to Life...Together

Track(s): Innovation & Research Practice, Professionalism & Leadership

Karen Heskett, Medical Librarian, UC San Diego

Heather Holmes, Associate Director of Libraries, Medical University of South Carolina

Jon Eldredge, Professor, University of New Mexico Health Sciences Library & Informatics Center

Margaret Hoogland, Clinical Medical Librarian, Mulford Library, University of Toledo

Marie Ascher, Lillian Hetrick Huber Endowed Director, New York Medical College

Session Overview:
The session will begin with a brief description of the process used to develop the MLA Research Agenda, followed by an unveiling of the "most important" questions that emerged from the process. Groups will break off to discuss methodologies that might be used to answer their chosen question(s). Ultimately we hope that some research collaborations form from the discussion. If you are interested in research, this will be a great session to attend.

Instructional Methods:
This will be a very hands-on participatory session. After presentation of the brand new MLA Research Agenda, participants will have the option to move to a topical table to discuss questions of interest and work out potential research plans. Facilitators will provide prompts (What does this question mean? What would be some potential study designs? Who would be your participants? Consider biases. What outcomes would you measure?) to drive discussion. Participants will be encouraged to report out in order to engage others with their emerging plans and potentially recruit further collaboration.

Participant Engagement:
The session will begin with a presentation of the recently completed MLA Research Agenda, which will be a list of about 15 research questions. Participants will self-select questions of interest and in small groups discuss potential methodologies for answering the questions. Ultimately we hope that collaborations are formed and participants who are so inclined walk away with a plan to begin research on the questions.

Session Learning Objectives:

- Participants will be able to describe the Delphi process undertaken to create the MLA Research Agenda.
- Participants will be able to list three questions that make up the final MLA Research Agenda.
- Participants will work with others to design a study plan to address one research question posed by the MLA Research Agenda.

Sponsor(s): Research Caucus

Building a Strong & Supportive Workplace: Negating Incivility & Bullying in the Library Environment

Track(s): Professionalism & Leadership

Maggie Albro, Agriculture Librarian; Interim Assistant Head, Health, Wellness, & Professional Programs Unit, University of Tennessee, Knoxville

Rachel Keiko Stark, Health Sciences Librarian, Sacramento State University

Session Overview:
Utilizing evidenced based techniques, this session will provide participants with tools, skills, and materials to

identify, call-out, and address lateral violence in the workplace. Presenters will provide definitions, example scenarios, and interactive, hands-on practice appropriate for everyone who works in health sciences librarianship. Engaged and active participation by attendees is a requirement of this session to allow for thorough skill-building and effectiveness of the rehearsal technique employed.

Instructional Methods:
We will use community-building via working groups to establish shared language and definitions surrounding lateral violence in the workplace. Participants will be required to exercise skills learned during the session and practice intervening in bullying/incivil scenarios provided by the facilitators. Participants will have access to asynchronous online materials, which they will have access to upon completion of the session. Participant feedback will be gathered to improve upon said materials to continue learning and growing as a community.

Participant Engagement:
Participants will be placed into small groups, which will work together and then report back to the larger community. Small groups will be modify provided materials to better reflect their individual institutions and situations to have ready-to-implement anti-bullying materials they can bring back to their libraries. Individuals will practice utilizing their developed materials with other individuals, and the entire group will be provided with scenarios with which to actively role-play the witness intervention experience in workplace bullying.

Session Learning Objectives:

- Participants will develop a shared understanding of bullying and incivility in the workplace.
- Participants will practice effective techniques for identifying and stopping lateral violence in the workplace.
- Participants will utilize cognitive rehearsal therapy to prepare to bring these learned skills to their workplaces.

Career Planning Bootcamp for Health Science Librarians

Track(s): Professionalism & Leadership

Brenda Linares, Associate Dean of Library Services, University of Missouri Kansas City

Julie Bolin, Library Director, Richard D. Haines Medical Library, Baylor Scott & White Health Central Texas

Katie Prentice, Executive Director, Texas Medical Center Library, Houston, Texas

Rachel Helbing, Head of Health Sciences Libraries, University of Houston Libraries

Susan Steelman, Head of Education & Research Services, University of Arkansas for Medical Sciences Library.

Katie Pierce Farrier, Data Science Strategist, Network of the National Library of Medicine, Region 3

Laura Haygood, Health Sciences Librarian for Public Health, Brown University

Lindsay Blake, Clinical Services Coordinator, UAMS

Sandra Desjardins, R & I Librarian, Texas Medical Center

Session Overview:
The career bootcamp will focus on career development, including writing detailed cover letters, understanding tenure and promotion processes including Academy of Health Information Professionals (AHIP), building professional networks, and developing skills to better support career goals. The first half of the session will have short talks on each focus area. Participants can then choose to join one of three breakout rooms to discuss selected topics in further detail. Participants are encouraged to bring questions to get more personalized feedback.

Instructional Methods:
Panel members will give a short presentation on career development topics, followed by an activity and Q&A in the breakout sessions.This session will emphasize panel questions and facilitate discussion around career development interests. Participants will be provided with an accessible copy of presentation materials and handouts.

Participant Engagement:
Participants will break out into three groups that focus on (1) resumes and cover letters, (2) tenure, promotion, and AHIP processes, and (3) professional goal setting, networking, and skill building. Participants will have the opportunity to ask more in-depth questions and delve further into career planning topics with activities and materials related to each area of interest. Participants will also be encouraged to exchange contact information with a “buddy.” Buddies will agree to contact each other after the conference and provide support to each other as they navigate their respective careers.

Session Learning Objectives:

- Identify key parts of job listing and write detailed cover letters that address the job description. Gain a greater understanding of the types, roles, and paths in health science libraries.
- Differentiate and compare faculty rank, tenure, promotion, and AHIP processes.
- Practice setting SMART goals and determining the training, skills and/or networking opportunities needed to meet these goals.

Sponsor(s): Early Career Librarian Initiative of the South Central Chapter of the Medical Library Association, MLA Leadership & Management Caucus, MLA Professional Recruitment and Retention Committee, MLA Public Services Caucus

Case by Case: A Panel Discussion and Workshop on Developing Case Scenarios for Health Sciences Related Searching Demonstrations in Instruction

Track(s): Clinical Support, Education, Information Services

Bradley Long, Embedded Health Sciences Librarian, Penn State College of Medicine

Brandi Tuttle, Research & Education Librarian, Duke University Medical Center Library & Archives

Elisa Cortez, Medical Education & Clinical Outreach Librarian, University of California, Riverside

Katy Smith, Medical Center Library, University of Missouri

Lisa Marks, Director, Library Services, Mayo Clinic in Arizona

Suzanne Fricke, Medical Librarian, Washington State University Health Sciences Library

Cynthia Chelf, Academic Medical Librarian, Mayo Clinic

Erin Reardon, Public Health Informationist, Emory University

Laura Zeigen, Health Sci Ed/Research Lib, OHSU

Michelle Knecht, Senior Medical Librarian, Schmidt College of Medicine, Florida Atlantic University

Stephanie Schulte, Director; Professor, The Ohio State University

Session Overview:
We will start with a warm-up exercise (5 minutes), followed by a 30-minute panel of 4-5 librarians discussing various aspects of developing and finding appropriate case scenarios for instruction. Participants will be divided into small groups (4-6 people) to work collaboratively to create scenarios or discuss a related theme (30 min). A volunteer from each table will share a few highlights from the small group discusion (15 min), then participants will engage in 5 minutes of individual reflection and writing to connect workshop ideas to their everyday contexts and practices, ending with a 5-minute wrap-up that includes further reflection on what aspect of creating/writing scenarios for group search demonstrations remains most challenging for them and how confident they are feeling in writing relevant and searchable research question examples.

Instructional Methods:
We will start with a warm-up exercise (5 minutes), followed by a 30-minute panel of 4-5 librarians discussing various aspects of developing and finding appropriate case scenarios for instruction. Participants will be divided into small groups (4-6 people) to work collaboratively to create scenarios or discuss a related theme (30 min). A volunteer from each table will share a few highlights from the small group discusion (15 min), then participants will engage in 5 minutes of individual reflection and writing to connect workshop ideas to their everyday contexts and practices, ending with a 5-minute wrap-up that includes further reflection on what aspect of creating/writing scenarios for group search demonstrations remains most challenging for them and how confident they are feeling in writing relevant and searchable research question examples.

Participant Engagement:
In addition to 1-2 audience participation polls, 30 minutes will be dedicated to having participants work in small groups on developing their case scenarios on a variety of topics (which would have been touched on by the panelists) and 15 minutes of large group discussion sharing what came out of these small groups, as well as some time for participants to reflect on what from the session might be actionable for them to apply when back in their own work environments.

Session Learning Objectives:

- Describe essential elements of a case scenario
- List one best practice and one worst practice when developing a case scenario
- Name one place to locate a possible case scenario

Sponsor(s): Libraries in Health Sciences Curriculums (LiHSC) Caucus

Creating Community: Librarians Growing Stronger Together in Communities of Practice

Track(s): Professionalism & Leadership

Kay Strahan, Assistant Professor/Northwest Campus Librarian, UAMS

Laura Haygood, Health Sciences Librarian for Public Health, Brown University

Sandra Desjardins, R & I Librarian, Texas Medical Center

Shannon Jones, Director of Libraries, Medical University of South Carolina

Tamara Nelson, University of Tennessee Health Sciences Center, African American Medical Librarians Alliance Member

Session Overview:
Community exists in many ways, shapes, and forms. It can be intimidating or alienating to exist in the profession without a sense of community. Finding your support system can come in a variety of ways that will be discussed in this session.

Instructional Methods:
During the session, the panelists will present 5-minute presentations about their communities of practice within health sciences librarianship in a moderated format. These talks will include the provenance of their communities, what their community has meant to them, and how they believe communities can be curated in all spaces of health sciences librarianship. They will also present questions to participants of the immersion session to assist participants in thinking about how they fit within their own communities.

Participant Engagement:
After the lightning talks, panelists will engage attendees in small group discussions and group sharing, where attendees will discuss the communities in which they are situated and the types of professional support they need to flourish in their positions.

Session Learning Objectives:

- Observe existing communities of practice in health sciences librarianship to inform their practice.
- Examine their own needs in order to explore communities in health sciences librarianship.
- Learn strategies for establishing common ground to build a meaningful community.

Sponsor(s): African American Medical Librarians Alliance (AAMLA), Northwest Arkansas Library Association (NWALA), Early Career Librarians Initiative (ECLI)

Creating Interdisciplinary Interest in the All of Us Researcher Workbench Across Campus Communities

Track(s): Education, Information Services, Innovation & Research Practice

Brianna Chatmon, Instruction and Scholarly Communications Librarian, Marymount University

Pamela Buzzard, Cline Library

Sarah Joseph, Health Research/Data Librarian, Grand Valley State University

Kristi Sadowski, Program Manager, All of Us Data Training and Engagement for Academic Libraries Program, ORAU

Session Overview:
The first 20 minutes will review key features of the All of Us Research Program’s potential benefits and how the library is the key to cross disciplinary awareness and education benefiting the individual researcher and the institution.

The next 30 minutes will focus on engaging campus communities with biomedical and public health data. Participants will rank which research audiences to review as a group. Presenters will lead a discussion of research field needs and wants, followed by 5-minute breakout sessions for audiences to brainstorm how to achieve campus engagement and determine ideal partners. A digital toolkit will allow attendees to explore additional research fields.

The final 40 minutes will introduce a panel of library workers participating in the All of Us Academic Libraries Program, discussing the Researcher Workbench utilization at their institutions and data engagement activities held for their campus communities. 20 minutes will be reserved for audience questions.

Instructional Methods:
Participants can visit researchallofus.org to gain a better understanding of the All of Us Research Program and Research Hub in advance. During the session, a visual presentation will be accompanied by a digital toolkit of resources for post-session review and engagement implementation. Certain files from the digital toolkit, primarily worksheets, will be provided in hard copy format for live completion and note taking. There will be a moderated session discussing different research audiences and researcher information needs.

Participant Engagement:
Participants will have the opportunity to direct among a preset list what resources would be most beneficial to review as a group –this will be determined using an online collaborative poll software that will be employed throughout the session. Short breakout discussions will facilitate ideation of how to engage researcher audiences. There will be an opportunity for questions to be answered by library workers who have already started engaging their campus communities with the All of Us Research Program through the All of Us Data Training and Engagement for Academic Libraries Program.

Session Learning Objectives:

- Gain knowledge of the All of Us Research Program and Research Hub.
- Understand how to employ the Research Hub and Researcher Workbench to grow research capacity including biomedical and public health data skills within your campus community.
- Ability to apply tools and resources provided to develop tailored messaging for promoting the workbench to both emerging and established researchers of various disciplines. promoting the workbench

Deciphering the Editorial Peer Review Process

Track(s): Innovation & Research Practice

Kaitlin Throgmorton, Data Librarian for the Health Sciences, Yale University

Kerry Dhakal, Associate Professor, Research and Education Librarian, Health Sciences Library, The Ohio State University

Jon Eldredge, Professor, University of New Mexico Health Sciences Library & Informatics Center

Session Overview:
Overview of the editorial peer review process from the perspective as an author, peer reviewer, and editor. This will include definitions, characteristics, and varieties.

This immersion session will equip participants to either serve as editorial peer reviewers for journals or to respond productively to peer reviewer feedback.

Instructional Methods:

Breakout Tables following introduction, brief panelist speakers, and Q&A: different groups will be given different exercises. Participants interested in learning how to peer review will be given sample text excerpts to peer review. Prospective or experienced authors will be asked to address concerns following receipt of responses from peer reviewers to a sample manuscript

Participant Engagement:
Participants will be given enough variety of active learning modalities to maintain their interest and engagement over this 90-minute immersion session.

Session Learning Objectives:

- Be able to outline the key questions asked by an editorial peer reviewer as they approach evaluating a submitted manuscript;
- Describe at least three (3) ways that an editorial peer reviewer offers constructive feedback to authors on how to improve their manuscripts
- Explain at least three (3) methods for authors to respond positively to editorial peer reviewers’ comments about the authors’ submitted manuscripts

Sponsor(s): MLA Research Caucus (Lead Sponsor), Medical Library Education Caucus, Scholarly Communications Caucus, Systematic Review Caucus

Designing Health Sciences Library Instruction for Scalability

Track(s): Education

Caitlin Plovnick, NYU Health Sciences Library

Gregory Laynor, Systematic Review Librarian, NYU Health Sciences Library, NYU Grossman School of Medicine

Juliana Magro, Education and Research Librarian, NYU Langone Health

Justin de la Cruz, Associate Director, NNLM NCDS, NYU Langone Health

Session Overview:
Health Sciences Libraries are responsible for meeting the diverse information needs of many different user groups such as Medical Students, Clinicians, and Researchers. We often find ourselves teaching similar content across different modalities and at different levels of expertise. This immersion session will look at intentional design of health sciences library instruction resources for adaptability and scalability, using examples from several different cases. Participants will have the opportunity to develop their own scalable instruction plans.

Instructional Methods:
We will briefly present several different cases of library instruction adapted and scaled from one format or audience to another. These include classes in Evidence-Based-Medicine, Systematic Reviews, and Data Management.

Following the presentation, participants will work in small groups, facilitated by presenters, to draft their own project plans for adapting and scaling instruction.

The session will conclude with a full group discussion and Q&A.

Participant Engagement:
In small groups facilitated by presenters, participants will work from a template to draft project plans for adapting and scaling instruction. The session will conclude with time for a full group discussion and Q&A.

Session Learning Objectives:

- Describe key questions to consider when scaling and adapting library instruction sessions
- Identify available resources to help design instruction sessions for scalability
- Develop goals and begin drafting plans for scaling instruction sessions

“Do You See Me?”: The Lack of Representation of Diverse Skin Tones in Medical Education

Track(s): Education, Health Equity & Global Health

Katresa Gardner, Public Services Librarian, Philadelphia College of Osteopathic Medicine

Stacie Fairly, Associate Professor of Microbiology and Immunology, Philadelphia College of Osteopathic Medicine

Valerie Cadet, Assistant Dean of Health Equity Integration and Associate Professor of Microbiology and Immunology - Philadelphia College of Osteopathic Medicine

Session Overview:
Explore ways to develop inclusive visualization resources for medical students to acquire to detect important clinical signs and disease manifestations in all patient skin tones. Learn how the power of faculty collaboration can achieve the goal of DEI in medical education.

Find out how you can incorporate these powerful resources at your campus library.

Instructional Methods:
A case-study/presentation will be the format for the session.

Polls will be used to invoke audience participation.

A Q/A session will also be held.

Participant Engagement:
Polls will be used.

Q&A/panel discussion will be held.

Session Learning Objectives:

- Foster awareness of issue and learn ways to develop inclusive visualization resources for medical students and faculty.
- Learn how to collaborate with stakeholders and faculty to incorporate and embed diverse images in classroom presentations for curriculum use.
- Learn what PCOM Library has done and understand the value and feedback gained from PCOM learning community.

Sponsor(s): African American Medical Librarians Alliance (AAMLA), Philadelphia College of Osteopathic Medicine (PCOM)

The Image of the Medical Profession in Film

Track(s): Professionalism & Leadership

Jeff Slagell, Library Director at the University of Arkansas for Medical Sciences (UAMS)

Session Overview:
This session will examine the role film has played in the perception of medical professionals and its collective impact throughout history. The cinematic depiction of professionals, practices, and institutions can produce a powerful influence on health-related behaviors and decisions among the general public. Greater awareness of these issues can minimize bias, stereotypes, and misinformation and will offer broad areas of interest to all participants.

Instructional Methods:
Using the flipped classroom approach to learning, selected films will be recommended for viewing ahead of time. During the session, the facilitator will provide brief topical overviews of the films focusing on their historical, cultural, and social context. Guided small group discussion will encourage dialogue and reporting will enhance overall collaboration. In addition, Kahoot! gaming software will be utilized for real-time polls and quizzes to review and reenforce information and concepts.

Participant Engagement:
The flipped session format encourages participants to become actively involved in the content before the session begins. Facilitated small and large group discussion provides deeper conversation of the material and Kahoot! gaming software creates an interactive learning experience.

Session Learning Objectives:

- Identify the historical context of the medical profession in film and trace its evolution over time.
- Analyze the impact of film on public perceptions including stereotypes and positive or negative portrayals.
- Recognize how film can influence health-related behaviors and decisions among the general public.

Sponsor(s): History of the Health Sciences Caucus

Ladder or Lattice? Redefining Career Success Based on Your Values

Track(s): Professionalism & Leadership

Rich McIntyre, Emerging Technologies Librarian, UConn Health

Beverly Murphy, Assistant Director for DUHS Nursing Library Services; Liaison to Watts College of Nursing, Duke Health

Elizabeth Whipple, Assistant Director for Research and Translational Sciences, Ruth Lilly Medical Library, Indiana University School of Medicine

Molly Montgomery, Assistant Director of Medical Education & Access Services, Indiana University School of Medicine

Session Overview:
Each panelist will briefly share their career path and how their values have informed their career choices and how they define professional success. We will have panelists who represent a variety of career path options in medical librarianship from those happy to not climb the traditional career ladder to director roles, to those who left director roles, to those who are following a more traditional management path. We will encourage discussion via pair and share, group work, and asking participants to share their views and experiences. We will also have a worksheet where participants can reflect upon their values and how that may shape what success at work looks like for them.

Instructional Methods:
Each panelist will share their own case study from their experience. Attendees will be provided with worksheets for reflection of their own experience. Question and answer period will also be employed to share knowledge with all attendees. Pre readings will be provided but not mandatory for completing the learning objectives.

Participant Engagement:
We will encourage discussion via pair and share, group work, and asking participants to share their views and experiences. We will also have a worksheet where participants can reflect upon their values and how that may shape what success at work looks like for them.

Session Learning Objectives:

- Participants will work to identify their core personal and professional values and describe how these influence their definition of success.
- Participants will reflect upon times when their personal and professional values may not have been aligned with their career choices or decisions.
- Participants will learn techniques to prioritize their values to determine what professional success looks like moving forward.

Sponsor(s): Leadership & Management Caucus, Osteopathic Caucus

Lessons in Slow Librarianship: Making Room for Thought, Intention, and Kindness

Track(s): Professionalism & Leadership

Jill Deaver, Head of Clinical, Academic, and Research Engagement, University of Alabama at Birmingham

Lorin Jackson, Executive Director, NNLM R2/MUSC

Session Overview:
This session will involve defining what Slow Librarianship is and what it means. During a panel session, we'll

hear from at least three different library leaders, each at different stages of their leadership journeys. There will be a moderated Q&A discussion where these leaders will share insights and offer practical strategies for implementing Slow Librarianship. Audience members will have the opportunity to ask questions and share insights from their own experience. There will be an interactive reflection activity where participants can practice implementing thoughtfulness, intention, and kindness. Participants should leave this session with a stronger understanding of Slow Librarianship and how they can bring back what they've learned to their respective libraries.

Instructional Methods:
1. Pre-Reading: Farkas, M. G. (2021, October 18,). What is slow librarianship? | information wants to be free. https://meredith.wolfwater.com/wordpress/2021/10/18/what-is-slow-librarianship/

2. Pre-Work: Bring a case study to discuss with table mates a challenge with Slow Librarianship

3. Interactive Activities: Moderated Q&A, Gallery of Concerns, using chart paper & post-it note activity. Will include time for reflection and application of takeaways at the culmination of the session, Case Studies discussion in breakout groups

Participant Engagement:
Encourage participants to prepare a case study in advance of the session and participate actively in discussion at their tables. Request that participants ask questions during the panel session.

Session Learning Objectives:

- Describe the role that intersectionality and honoring diverse identities play in fostering an inclusive, supportive work environment that celebrates everyone’s uniqueness
- Discuss practical strategies for implementing Slow Librarianship within different library contexts
- Leave session with experience from a reflective exercise that encourages the tenets of slow librarianship

Sponsor(s): African American Medical Librarians Alliance (AAMLA)

Novel Librarian Collaborations: Stronger Together

Track(s): Information Services

Caroline Marshal, Senior Medical Librarian, Public Services, Cedars-Sinai Medical Center

Ellen Aaronson, Mayo Clinic

Layla Heimlich, Medical Librarian, MedStar Washington Hospital Center

Michelle Bass, Clinical Information Librarian, Pennsylvania Hospital

Michelle Kraft, Library Director, Cleveland Clinic

Susan Keller, Research Librarian, Children’s National Hospital

Sydni Abrahamsen, Librarian III, Mayo Clinic

Andrea Harrow, Clinical Librarian, University of Southern California

Basia Delawska-Elliott, Medical Librarian, Providence

CeCe Railey, System medical Librarian, Norton Healthcare

Lindsay Blake, Clinical Services Coordinator, UAMS

Session Overview:
Attendees will join a table discussion on their topic of interest and be given several prompts to help facilitate dialogue. Each table will document and synthesize discussion to develop a project(s) or research question(s) to present to the wider group. The table topics are: Solo librarians, Consumer health & patient education in the EHR, Librarians in hospital-wide committees, Cyber-attacks and IT blocked platforms; Librarians in Magnet designation, Collaborating with AI, Clinical rounding; Librarians in well-being and mentorship. The first 45 minutes will be dedicated to discussion with the final 30 minutes saved for presenting results.

Instructional Methods:
Participants will learn through discussion and presentation. Prompts for the discussion will be shared before the session and include: describe what worked well in their particular situation, what did they try that may not have succeeded as intended, what lessons did they learn that they would like to communicate to others engaged in a similar situation, how context-specific or widely-applicable was their model, and did they collect any measurements or other results? We will need a large presentation notepad and markers, and access to Jamboard to also collect discussion notes and thoughts. Tables will be asked to assign a timekeeper, notetaker, and someone willing to present to the wider group. Tables will be capped at 6-8 participants to allow for everyone to have time to contribute and movement between tables will be allowed.

Participant Engagement:
Group discussion and presentation will engage participants. We will share MLA rules regarding mutual respect and good conduct.

Session Learning Objectives:

- Evaluate colleagues' descriptions of collaborative projects to apply to your own user base and services
- Assess collected information to design project(s) or research question(s) for further development and collaboration
- Evaluate final results shared by each of the tables for additional collaborative ideas and possible applications

Sponsor(s): Hospital Library Caucus & Research Caucus

Pause on the Play: Prioritizing Wellness and Wellbeing in Health Sciences Libraries

Track(s): Professionalism & Leadership

Beverly Murphy, Assistant Director for DUHS Nursing Library Services; Liaison to Watts College of Nursing, Duke Health

Kelsa Bartley, Education & Outreach Librarian, Librarian Assistant Professor, Learning, Research, and Clinical Information Services, Louis Calder Memorial Library, University of Miami Miller School of Medicine

Shannon D. Jones, Director of Libraries & Professor, Medical University of South Carolina

Tamara M. Nelson, Director of Library Engagement & User Experience, Medical University of South Carolina

Session Overview:
Though framed through the lens of Black women library leaders, the session is relevant to leaders at all levels in librarianship. This facilitated discussion will provide a platform for leaders to openly share their insights, experiences, and challenges in navigating the demands of academia. From battling the stereotype of being "presumed incompetent" to contending with unfair performance expectations, this session sheds light on the unique difficulties faced by Black women leaders in the academy. By fostering dialogue and sharing personal anecdotes, attendees will gain a deeper understanding of the toll that stress can take on their personal and professional lives. This session will advocate for health sciences librarians from all backgrounds to prioritize wellness and well-being in their lives.

Instructional Methods:
A short presentation on the impact of trauma and stress on personal and professional well-being in which presenters will share personal anecdotes. Participants will engage in facilitated group discussions followed by large group sharing. Time will also be allotted for questions throughout and at the conclusion of the session. Participants will be asked to read prior to the session:

- The different types of trauma explained. https://www.talkspace.com/blog/types-of-trauma/

- Being anti-racist is central to trauma-informed care: Principles of an anti-racist, trauma-informed organization. https://www.nctsn.org/resources/being-anti-racist-is-central-to-trauma-informed-care-principles-of-an-anti-racist-trauma-informed-organization

- Two black women university presidents have died, spurring heartrending accounts of workplace discrimination.https://www.choice360.org/tie-post/two-black-women-university-presidents-have-died-spurring-heartrending-accounts-of-workplace-discrimination/

Participant Engagement:
Participants will engage in group dialog with other participants and presenters to discuss strategies and practices to prioritize and enhance wellness and well-being in health science libraries. Participants will be invited to summarize their group discussion using Padlet. One or more live polls will be embedded throughout the session using live polling, and there will be time built into our schedule for audience Q&A.

Session Learning Objectives:

- Differentiate between various types of traumas and the nuanced ways they manifest in individuals' daily lives.
- Discuss the impact of trauma and stress on personal and professional well-being, specifically focusing on the health sciences library environment.
- Describe strategies and practices to prioritize and enhance wellness and well-being.

Sponsor(s): African American Medical Librarians Alliance (AAMLA)

Reenvisoning Libraries in Uncertain Times: Attracting, Retaining and Growing Highly Talented Teams!

Track(s): Professionalism & Leadership

Catherine Soehner, Executive Director, Univ of Utah

Heidi Heilemann, Associate Dean/Director, Stanford University

Hsinliang Chen, Chief Library Services Officer, Philadelphia College of Osteopathic Medicine

Melissa De Santis, Director, Strauss Health Sciences Library, Univ of Colorado Anschutz Medical Campus

Teresa Knott, Associate Dean, VCU Libraries, and Director, VCU Health Sciences Library, Virginia Commonwealth University

Maud Mundava, Campus Head/Assistant University Library Director, A.T. Still University

Session Overview:
This session will highlight ways to strategically and collectively discuss and share solutions to increased organizational challenges such as staffing issues --- high turnover, recruitment and retention of a highly diverse competitive skilled workforce. The presentation will serve as a blueprint bringing together a list of best practices and practical solutions to this topic. Even though its discussion will be centered on what is going on in academic health sciences libraries, the discussion is applicable to other types of libraries.

Instructional Methods:
The participants will have a question and answer session -- submit questions before and during the panel discussion via either via poll everywhere or in person. There will be an opportunity to share their opinions, experiences via breakout groups and report. Findings from the literature will also be briefly presented and a bibliography of recommended readings will be passed out. Twitter, to assist with discussion and to post final conclusions. Finally, a short questionnaire will be completed to evaluate the effectiveness of the session

Participant Engagement:
Audience will be engaged by having a well organized moderated question and answer session. Findings from literature will also be briefly presented and a bibliography of recommended readings will be passed out. Furthermore, discussion will be enhanced by incorporating web 2.0 technologies (such as Facebook, Twitter, etc.) to assist with discussion and to post final conclusions. Finally, a questionnaire will be used to evaluate the effectiveness of the session.

Session Learning Objectives:

- The participants will gain insights and best practices on how to lead workplaces that successfully attract the most qualified employees, match them to jobs for which they are best.
- Explore ways to navigate and strategically implement change that address topics such as DEAI, how to stay relevant, and demonstrate value.
- Identify tools / strategies to effectively onboard new employees, grow.

Sponsor(s): New Members Caucus, Academic Libraries Caucus, ACRL health sciences group, African American Medical Alliance Caucus

Speed Dating Through Evidence Synthesis Education: Finding Your Instructional Match

Track(s): Information Services

Jenessa McElfresh, Interim Associate Director, University of Tennessee Health Science Center

Rachel Walden, Reference & Instruction Librarian, Vanderbilt University

Cozette Comer, Evidence Synthesis Services Coordinator, Virginia Tech

Leila Ledbetter, Research & Education Librarian, Duke Medical Center Library

Rachel Whitney, Research and Education Informationist, Medical University of South Carolina

Sally Smith, Health Sciences Librarian, Clemson Libraries

Session Overview:
The first half of the session will be a panel presentation where we answer questions about our user base, institutions, and how we provide ES education (what has and hasn’t worked). The The second half of the session will be interactive, where participants can choose an ES topic, such as systematic searching or critical appraisal and engage in a group discussion with a panelist. Each group will brainstorm an instruction activity around the chosen topic.

Instructional Methods:
The panel discussion will be formatted as a Q&A session akin to speed dating, where attendees can learn about various ES instruction approaches. The breakout session of small group discussions will allow participants to articulate how they see their role in ES instruction at their institution and will allow them to brainstorm activities/approaches they may adopt. We will also provide a handout for guided note-taking on criteria (resources, time, accessibility) participants must consider before implementing an ES instructional approach.

Participant Engagement:
We will incorporate interactive polling during the panel discussion to engage audience feedback. We will also have a breakout session of small group discussion to talk about activities/approaches discussed in the panel discussion.

Session Learning Objectives:

- Discuss the role that the librarian or information professional has in evidence synthesis education
- Identify evidence synthesis instructional strategies or approaches that suit the needs of your users
- Create a plan for how to provide evidence synthesis instruction at your institution

Sponsor(s): Systematic Reviews Caucus

Teaching for the Non-Teacher: Creating a Lesson Plan

Track(s): Education

Amy Houck, Informationist, Geisinger College of Health Sciences

Session Overview:
Medical librarians are often tasked with creating instructional materials and engaging with faculty to support effective teaching and learning in academic and clinical settings. This session will help participants to communicate with faculty to ensure clarity of scope for the instructional session (10 min), identify the various factors, including the location or setting, the class participants, time allotted and more, that need to be considered prior to the actual instruction (20 min), and create a lesson plan (15 min). Through an interactive lecture (45 minutes=10+20+15) and working groups (25-45 minutes), participants will leave this session

equipped with newfound teaching skills, advanced communication techniques, and a practical understanding of how lesson plans can transform their instructional sessions.

Instructional Methods:
I will begin with an interactive lecture, walking the participants through steps of the planning process. Topics will include the initial email/request and meeting with the facilitator (10 min); describing, explaining, and eliciting from participants what factors should be considered for their lesson plans (20 min); and demonstrating components of an effective lesson plan (15 min). The participants will then be split into groups to work on 2 assignments-1 on creating a lesson plan with provided information and 1 on a hypothetical conversation with a facilitator (25-45 min).

Participant Engagement:
I will engage participants during the interactive lecture by eliciting responses about their personal experiences and needs in preparing for instructional sessions or presentations and tailoring the discussion appropriately. I will facilitate working groups on the given topics and monitor participant involvement and progress on creating a lesson plan as well as “pitching” the session to a facilitator.

Session Learning Objectives:

- Participants will be able to discuss the lesson they will teach with a facilitator.
- Participants will be able to identify factors that they need to consider before teaching.
- Participants will be able to create a lesson plan incorporating the factors they’ve identified.

Sponsor(s): Medical Library Education Caucus

PAPER PRESENTATIONS: RESEARCH ABSTRACTS

We have sorted paper presentation research abstracts in this section by title in alphabetical order.

Advancing Digital Equity in Rural Agricultural Communities: An Applied Research Project

Track(s): Health Equity & Global Health

Jamie Bloss, Research Librarian, Laupus Library - East Carolina University

Leslie Cofie, East Carolina University

Mary Roby, Asst Director for User Services, East Carolina University

Roger Russell, Associate Director, East Carolina University

Objectives:
There are profound barriers to digital inclusion for farmworkers which adversely affect health outcomes. Our applied research project focuses on discovering and documenting barriers to internet access for migrant farmworkers. The research questions asked include the topics of accessibility of internet access and devices for farmworkers, the information and digital literacy levels of farmworkers and farmers, and to what extent do public libraries support or provide outreach to these communities and what barriers they face.

Methods:
This project is divided into three working groups, or Aims. For Aim 1, farmworkers across the state are surveyed by community health workers assigned to work directly with migrant farmworker communities. These surveys focus on discovering needs and barriers regarding internet access. We anticipate 1000 participants as our sample group. For Aim 2, three focus group discussions (FGDs) were held with farmers and up to fifteen interviews are being conducted with farmworkers. These interviews and FGDs focus on needs and barriers of farmers and farmworkers in accessing the internet. For Aim 3, a survey was distributed to NC public libraries to determine the current level of engagement with the farmworkers across the state, interest in being more engaged, and the role of public libraries in establishing internet access for farmworkers.

Results:
In Aim 1, so far we have over 600 respondents of a total projected 1000. 26% of survey respondents reported sometimes having internet access, 4.6% reported no internet access, and 69.4% reported having internet access. 45.6% reported the internet was sometimes fast enough to watch a video. In Aim 2, farmers expressed lack of access to internet and 5G. Some felt confident in their online search skills and others wanted further training. In Aim 3, 37.9% of librarians have participated in providing outreach to agricultural communities. 5 respondents out of 58 had provided outreach to farmworkers. 11 respondents had already participated in county broadband internet planning or implementation and

Conclusions:
A need for reliable internet service persists for both farmworkers and farmers in the state of North Carolina. Several barriers for public libraries to outreach include lack of personnel/time, lack of advocacy skillset, and lack of budget or funding.

Assessing Reproducibility Among Recent Trainees

Track(s): Education, Information Management

Fred LaPolla, Lead, Data Services, NYU Langone Health

Robin Champieux, University Librarian, Oregon Health & Science University

Objectives:
We aimed to understand if the NIH's rigor and reproducibility training requirements are reflected in and impacting early career researchers' publications. Assessing the reproducibility of early career publications could highlight gaps in reproducibility practices and educational opportunities that medical libraries and librarians are well poised to respond to. To evaluate if current rigor and reproducibility training results in transparency in publications, we analyzed publications tied to K01 grants funded by the National Cancer Institute for new researchers in the past three years.

Methods:
We used NIH Reporter to identify Individual Career Development (K01) funded studies from 2021-2023 awarded by the National Cancer Institute and extracted associated publications. We focused on papers where the K01 award recipient was listed as a first or last author and pulled these citations from PubMed, as

these papers were most likely to be primarily directed and written by awardees. As our primary interest was in preclinical and clinical research, we excluded population health interventions and policy-related research. To guide the classification and description of reproducibility best practices in the assembled corpus, we developed an evaluation rubric based on Star and Nature guidelines, explicitly focusing on the information required by both checklists (e.g., providing sample size, providing details on randomization, etc.). The resulting rubric comprises 12 domains, including information on blinding, randomization, population characteristics, and study ethics. The evaluators then reviewed the methods sections for the publications associated with the K01 awards, using the rubric to assess for reproducibility and classifying the resulting papers according to strengths and weaknesses with regard to reproducibility. Prior to the review, we assessed agreement among the evaluators with a pilot sample of articles.

Results:
This research is currently being conducted, but the authors plan to share the following results about their research: aggregate data about the performance of the corpus of papers against the transparency rubric described above, data about the performance of the corpus of papers against each domain in the rubric, and data about the relationships between study types and rubric performance.

Conclusions:
This research is currently being conducted, but the authors plan to relate their results to the following areas of discussion: overall areas of strength and opportunity, the relationships between the reproducibility scores we observed and the scope and content of current NIH rigor and reproducibility requirements; and, the relationships between the reproducibility scores we observed and potential librarian-contributed interventions.

Assessing the Preclinical Evidence-Based Medicine Curriculum: Results From A Novel Pre- And Post-Test Instrument

Track(s): Education

Juliana Magro, Education and Research Librarian, NYU Langone Health

Joey Nicholson, Chair, Medical Library / Director, NYU Health Sciences Library, NYU Langone Health

Caitlin Plovnick, NYU Health Sciences Library

Objectives:
To evaluate the impact of the longitudinal preclinical Evidence-Based Medicine (EBM) curriculum on medical student EBM knowledge and skills via a new pre- and post-test.

Methods:
EBM is a crucial skill for medical students, and research has shown that building EBM skills into the preclinical curriculum has a positive impact on the application of these skills in clinical practice. However, assessing learner outcomes from an EBM curriculum is challenging; some tools do exist, but they are primarily focused on epidemiology content. To evaluate the development of students’ ability to formulate appropriate questions and efficiently search and evaluate information, we developed a pre- and post-test to be completed before and after the preclinical EBM curriculum takes place.

The preclinical EBM curriculum consists of six modules presented over the course of several months, featuring a mix of asynchronous and in-person learning activities focused on developing students’ ability to formulate answerable questions, search for evidence efficiently, and appraise evidence. This curriculum was developed to be consistent with USMLE exam questions on Interpretation of Medical Literature. The new test instrument consists of 16 questions created and peer-reviewed by faculty. We collected data over two

periods: the pre-test was employed in December 2022, and the post-test in June 2023. To analyze the data, we included data only from consenting students, de-identified it, and ran a paired t-test to test our hypothesis.

Results:
Out of 105 students who were required to take this course, 62 completed both sets of pre- and post-tests (59%). The paired t-test showed a statistically significant difference (p-value >0.001) for the pre- and post-tests, with a mean difference increase of 9.9 points (95% CI: 8.2–11.7).

Conclusions:
The results suggest that the EBM curriculum was effective in improving medical students’ EBM-related knowledge and skills. However, the sample size was relatively small, which limits generalizability. To that end, we will continue to apply the pre- and post-test to incoming classes and will seek to validate the test in the future. In the long-term, we will explore the feasibility of expanding the test to capture the retention and development of EBM skills from students who are farther along in their medical education.

Beyond Traditional Publication Metrics: Expanding Impact Measurement Of Librarian Co-Authored Publications Using Altmetrics

Track(s): Innovation & Research Practice

Emily Jones, Health Sciences Librarian, University of North Carolina-Chapel Hill

Rebecca Carlson, Eshelman School of Pharmacy Librarian, University of North Carolina, Chapel Hill

Fei Yu, Assistant Professor, School of Information and Library Science, University of North Carolina at Chapel Hill

Objectives:
This paper builds on a recently completed bibliometric analysis of librarian and pharmacist co-authored publications. The original study focused on traditional publication impact measures of the publication set and found that librarian-coauthored papers had a higher impact than comparable papers. Now, this study seeks to deepen understanding of these collaborations, particularly their social impact using altmetrics — a modern methodology designed to monitor and quantify online attention and engagement related to academic content.

Methods:
The authors conducted a comprehensive literature search of six databases and hand searching by author names. All unique citations were screened, and all 533 relevant papers will be analyzed. Altmetrics data for these articles will be sourced from Dimensions, utilizing their DOIs, PubMed IDs, and PMC IDs to ensure precise matching within the database. Upon successful matching, these records will be extracted from Dimensions to create our primary data set. Using this data set, we will compute descriptive statistics, focusing on the mean and median of the Altmetric scores, and juxtapose the mean Altmetric score with publicly reported benchmark scores in the fields of Pharmacy and Medicine. As an additional step, we will pinpoint institutions that have contributed to the highest Altmetric scores and analyze the geographical regions and media source where notable social impact was observed.

Results:
Our analysis will report the average and median Altmetric scores for librarian-pharmacist collaborative papers, which we plan to compare to scores reported in previous studies from other disciplines. Additionally,

we will identify and list the librarians and their affiliations that consistently contributed to the highest Altmetric scores. Furthermore, the specific social media sources driving these scores will also be delineated.

Conclusions:
By embracing altmetrics, we aim to offer a more comprehensive perspective on impact, capturing real-time digital interactions and enriching our understanding of how librarian-contributed scholarship resonates within a broader context. This research will be the first to illuminate the social impact generated by joint papers authored by librarians and pharmacists. We expect it will confirm the invaluable nature of interprofessional collaboration in enriching the pharmacy literature.

Building a Consensus for Tracking the Impact of Libraries: A Delphi study

Track(s): Clinical Support, Education

Gwen Wilson, Head of Library Research Services and Outreach, Mayo Clinic

Objectives:
Reach a consensus of impact variable data point terms and definitions that could be incorporated into existing reference library tracking systems.

Methods:
Utilizing the Delphi methodology, informational professionals were recruited to participate in focus groups. Based on the literature and a pilot focus group impact variable data point terms and definitions were drafted. The structured focus groups applied an adapted version of the norming process. The norming process utilized example reference service scenarios; participants were asked which impact variable data point best to fit the scenario. If conflict arose, the participants were asked what revisions to the terms/definitions would provide clarity. Focus groups continued building on the revisions of the previous focus group until the Delphi technique showed a saturation for consensus.

Results:
Between three rounds of focus groups there were 17 participates. The first focus group (n=5) made several edits to the impact variable. Following the Delphi technique, the second focus group (n=6) started with the revised impact variable data point terms and definitions from the first focus group. The second focus group made a few edits to the naming of the terms, definitions and added a new impact area. The third focus group (n=6) only made one small edit to a definition rewording the phrasing. Participants agreed that these terms and definitions are acceptable for implementation as a new data category.

Conclusions:
This Delphi study is the first with the objective to develop a consensus of impact variable data point terms and definitions to be utilized as a category within reference library tracking systems. The impact terms and definitions developed through this study have reached a saturation of consensus. This consensus is a great foundation for libraries to implement these terms and definitions as a new category within their reference library tracking system.

Building a Stronger MLA Together: Analyzing Member Engagement with MLA Caucuses

Track(s): Professionalism & Leadership

MLA Rising Stars Cohort, 2023-2024:

Kimberly Powell, Research Impact Informationist, Woodruff Health Sciences Center Library, Emory University

Michelle Knecht, Senior Medical Librarian, Schmidt College of Medicine, Florida Atlantic University

Rachel Walden, Reference & Instruction Librarian, Vanderbilt University

Rachel Whitney, Research and Education Informationist, Medical University of South Carolina

Objectives:
In 2019, the Medical Library Association (MLA) moved from a two-tiered community participation structure (Sections and Special Interest Groups) to the current Caucus model where members can join multiple Caucuses for no additional cost. However, the effectiveness of this change on member engagement has not yet been explored.

This study aims to examine how members engage with Caucuses and perceived barriers to engagement, with the ultimate goal of increasing sustainability and engagement of the MLA Caucuses. Our study addresses the following question: What factors significantly influence member engagement and commitment to an MLA caucus?

Methods:
This study will review data from a survey open to all MLA caucus participants along with data from MLA regarding caucus membership and engagement. Data from MLA includes how many members are in each caucus and the mid-year and annual activity reports. Caucus membership numbers and group activity reports were gathered beginning with the May 2019-April 2020 annual reports through the most recent May 2022-April 2023 reports. Presenters created a summary matrix of engagement activities documented by caucuses in their annual activity reports. Additionally, a membership table was created evaluating the number of members in each caucus from 2019-Present.

A survey assessing membership engagement with the caucuses and potential barriers to engagement was created and will be administered to all MLA members. To standardize the data received from this survey, demographic questions were pulled from previous MLA membership surveys. The survey was approved by MLA. This presentation will provide an overview of the activities and survey results, along with preliminary recommendations to increase the sustainability and engagement of the MLA caucuses moving forward. Quantitative results from the survey will be presented as descriptive statistics. Qualitative responses will be coded for thematic analysis.

Results:
Final results of this study are forthcoming pending statistical and thematic analysis of the survey data. Survey data being analyzed includes: demographic questions, such as how long a respondent has been an MLA member and whether they have held leadership positions in MLA; questions on participatory behavior in MLA caucuses, such as how many caucuses a respondent is involved with, how they engage with those caucuses, and any barriers to engagement; and wrap-up questions including future involvement in MLA. Study parameters require that all analysis will be complete by Spring 2024.

Conclusions:
Final results of this study are forthcoming pending statistical and thematic analysis of the survey data. The results of this study will be used to develop recommendations for increasing the engagement and

sustainability of the MLA caucuses moving forward. Additionally, this study may identify solutions to potential barriers for member engagement and sustainability.

Capturing User Feedback on Library Spaces

Track(s): Information Services, Innovation & Research Practice

Hannah Norton, Chair and Fackler Director, University of Florida

Lauren Adkins, Assistant University Librarian, University of Florida

Jane Morgan-Daniel, University of Florida

Mary Edwards, University of Florida, Health Science Center Library

Michele R. Tennant, Bioinformatics Librarian at University of Florida, Retired

Ariel Pomputius, Health Sciences Liaison Librarian, University of Florida

Courtney Pyche, Public Health Liaison Librarian, University of Florida

Nina Stoyan-Rosenzweig, Senior Associate in Libraries, University of Florida

Objectives:
Leaders of the [library] at the [institution] plan to renovate one of three floors in the library to improve efficiency and create additional study space. To inform planning for this renovation, a team of librarians solicited input from library users (and potential users) through an online survey and a series of in-person and online focus group sessions. A previous study conducted over 10 years ago at [library] proved helpful in contributing to the designs for renovations of two other floors of the library; therefore, the team used similar methods for this study.

Methods:
Using the previous study’s survey as a template, the team created an 11-question survey asking students, residents, postdocs, faculty, and staff what they want and need in an ideal library space. Added questions included a general open-ended question at the end of the survey, a question about what other spaces respondents use to study, and a question specifically for respondents who indicated that they rarely use the library about what changes would make the space more useful for them. The survey was open for eight weeks and disseminated via email from liaison librarians to their units, social media posts, digital signage in the library and nearby campus buildings, fliers placed in the library, student centers, and handed out at student-centric events, and announcements in some library-hosted workshops. Focus group sessions consisted of up to 10 participants with two librarian facilitators. Fourteen questions guided discussions of 30-60 minutes, with follow-up questions added based on the content of the conversation. Prior to data collection, the study went through the Institutional Review Board’s automated determination tool for exempt research and was approved as exempt.

Results:
Although data collection is not yet complete, over 275 survey responses have been received thus far, and two focus group sessions have been conducted. Respondents represent each of the six health sciences colleges and a variety of user types, including professional students, graduate students, undergraduate students, faculty, residents/fellows, staff, and postdocs.

Conclusions:
In the past, our library has found it especially helpful to seek input from library users before engaging a designer or architect for a renovation; we anticipate a similar utility for this data. Comparison to past data is

expected to provide insights into how user views of physical library spaces have changed over the last decade.

ChatGPT and Information Literacy Instruction: A Multi-Site Case Study of Possible Models for Introducing Students to AI LLMs in the Classroom

Track(s): Education

Alexander Carroll, Associate Director, Science and Engineering Library, Vanderbilt University

Jason Reed, Associate Professor of Library Science, Purdue University

Objectives:
While there is extensive literature on using information literacy training to introduce students to technical literature databases, there is less known about how to introduce artificial intelligence (AI) tools powered by large language models (LLMs). Likewise, there is limited evidence on if students are already using AI tools for information seeking and retrieval. We sought to explore different methods for integrating LLMs into the classroom and promoting responsible use of these tools in research and practice. As part of this exploration, we assessed students prior experience with using these tools and interest in using them in the future.

Methods:
This paper discusses the integration of LLMs into information literacy training at two different institutions, [Redacted Institution 1] and [Redacted Institution 2].

Institution 1: A module on using ChatGPT for information seeking was integrated into existing information literacy lectures within several undergraduate and graduate courses in science and engineering. Through didactic lecture and discussion, students considered which information related tasks were LLMs successful at completing, and which tasks did it struggled to complete. Following the session, students completed a post-session assessment reflecting on their previous use of LLMs for coursework, and their likelihood of using these tools in the future.

Institution 2: The AI LLM activity was a homework assignment for a second-year Doctor of Pharmacy, required class, at a large, land grant university, which followed an in-class discussion about AI LLMs and an overview of the assignment rubric. Students were tasked with using a drug information question they received during their rotations and asking a chatbot of their choice to provide an answer and then critically evaluate the response for accuracy. Students were also invited to complete a survey about their perceptions of AI LLMs for extra credit.

Results:
Institution 1: 102 students completed the post-session assessment. The assessment included two Likert-scale questions on previous and anticipated use of LLMs for coursework and two open response questions on possible utilities and limitations of using LLMs for information seeking.

Institution 2: 139 students completed the chatbot activity; extra credit survey collection is ongoing. The homework assignment will undergo a qualitative thematic analysis related to the perceived difficulty rating of the question asked , type of drug information question, and students critical appraisal of the response. The post-activity survey is a validated survey for ChatGPT based on the TAM model.

Conclusions:
Based on the early experiences at these two institutions, librarian-led instructional interventions may be able

to help students recognize the advantages and disadvantages of using LLMs for completing their coursework. By incorporating these tools into the classroom and selecting disciplinarily relevant examples, librarians can enhance the relevance of their existing information literacy instruction programs by inviting students to critically appraise the potential uses of AI LLMs in their coursework and future careers. While generative AI may feel like a daunting new tool that threatens to upend traditional information seeking practices, these experiences suggest the possibility that LLMs will instead become additional tools within a health information professionals existing toolkit for teaching information seeking, information retrieval

Clinical Librarian Starter Pack

Track(s): Clinical Support, Professionalism & Leadership

Ansley Stuart, Clinical Librarian, University of Louisville

Objectives:
What resources best support incoming clinical information librarians and what training gaps are standing in their way?

Methods:
An eight question online survey was taken by health science librarians (or former health science librarians) on what resources they found most useful in their career but particularly as they were onboarding with their first position as a health science librarian. This survey was undertaken in December 2021 and again in December 2023 to see if answers varied across the two years. Participants were asked to choose specific resources that they use to answer clinical reference questions, those that best supported their early career training, and those that helped them to become more knowledgeable as a clinical librarian. There was also an open ended question for participants to offer advice to incoming librarians or that they would have wanted to know themselves starting in their informationist role.

Results:
There was a wide range of responses from participants with 1 to over 31 years of health science experience. The majority of participants stated that they did not receive any formal or individualized training upon starting as a clinical librarian from their institutions. The most common training they best felt supported by were provided by the National Library of Medicine or one of its branches. The most common advice participants would offer their past selves or new librarians would be to build relationships with other librarians and to not be afraid to ask questions.

Conclusions:
There has been very little or poor training offered to incoming health science librarians from the institutions in which they work. Gathering opinions and information as to what were the most useful or valuable resources for early career librarians to glean professional knowledge from brings the medical library community closer to providing effective training. There could be less wasted time with librarians having to train themselves and therefore more time supporting health science and library users with accurate resources.

A Comparison of an Academic Health Sciences Library Director Profile in 2009 and 2024

Track(s): Professionalism & Leadership

Ana Cleveland, Regent Professor and Director of Health Informatics Program, University of North Texas

Jodi Philbrick, Principal Lecturer, University of North Texas

Objectives:
Fifteen years ago, the authors researched and presented a profile of an academic health sciences library director, and through this research, they will update the profile for 2024 by identifying the following of academic health sciences library directors:

- Sex distribution
- Professional titles
- Membership in the Academy of Health Information Professionals (AHIP)
- Publication record
- Elected positions within MLA

The objective is develop a 2024 profile of an academic health sciences library director based on the data collected and compare and contrast the findings from 2009 and 2024.

Methods:
The authors will use the 2024 Membership Directory of the Association of Academic Health Sciences Libraries to identify academic health sciences library directors in the United States and Canada. They will analyze the academic health sciences libraries' websites and MLANET to collect the sex, professional title, AHIP membership, and MLA elected positions of the academic health sciences library directors. The publication record of the directors will be identified using Library Literature and Information Science Source (EBSCO database). The collected 2024 data will be analyzed using descriptive statistics and compared and contrasted with the 2009 data.

Results:
In 2009, the majority (73%) of the 146 academic health sciences library directors in the United States and Canada were female. The most commonly used term in the professional titles was director (84%). Less than half (49%) of the directors’ were members of AHIP, with of which most being distinguished members (97%). Most of the directors (71%) had no elected positions within MLA. Lastly, the majority (75%) of academic health sciences library directors had at least one publication, and the median number of publications was 2. The 2024 results are to be finalized in Spring 2024.

Conclusions:
The 2009 profile of an academic health sciences library director was a female holding the title of director without an AHIP membership or MLA elected position who had published at least one article. The authors will present a 2024 profile of an academic health sciences library director and draw comparisons to the 2009 profile.

Database Usage Patterns in Existing Systematic, Scoping, and Rapid Reviews

Track(s): Information Services

Jennifer Westrick, Research Engagement Librarian, Rush University Medical Center

Objectives:
Conducting a thorough literature search is an integral part of a biomedical systematic review. These literature searches are done primarily in biomedical databases such as MEDLINE, Embase, and Scopus. When beginning a systematic review, health science librarians must decide how many databases to search, and which databases those should be. This dissertation will examine current practices in database usage by examining 600 such reviews, including scoping reviews, systematic reviews, rapid reviews, and systematic review meta-analyses.

Methods:
A search was conducted for reviews that were indexed into PubMed in a given week in 2023. Over 800 reviews were returned and 600 were randomly chosen. These reviews had to meet specific inclusion and exclusion criteria, such as including a search strategy that listed the databases used, and excluding animal and dental studies. 473 reviews met these criteria. Data was collected that specified which databases were used by these 473 reviews, along with ancillary information such as the type of review, librarian involvement, and the authors' country of origin.

Results:
The five most popular databases were, in descending order, MEDLINE, Embase, Web of Science, Scopus, and the Cochrane Library. 89 separate databases were identified. The average number of databases used was 4.14 per review. China produced the most reviews, followed by the United States and then the United Kingdom.

Conclusions:
This research is a snapshot of current practices. It is not meant to provide recommendations for database usage, as every review is different and requires individualized attention. The information gleaned from this research will be helpful to researchers and librarians who are the decision makers on database usage, and also to library administrators who make decisions on which databases to purchase.

Decoding the Misinformation - Legislation Pipeline: How Misinformation and Legislation Reproduce Harm and What Librarians Can Do About It

Track(s): Education, Health Equity & Global Health

Catherine Lockmiller, Health Science Librarian, Northern Arizona University

Objectives:
The author developed the theory of the Misinformation - Legislation Pipeline by studying the flow of anti-transgender misinformation from online echo chambers through a peer-reviewed article and into policy enacted to ban medical treatments for transgender people in the state of Florida.

Methods:
The theory is informed by a close read and analysis of a guidance document published by the Division of Florida Medicaid in June 2022. The document's references are further analyzed in an effort to identify how transphobic concepts find their way into policy via traditionally authoritative avenues.

Results:
The analysis is precluded with a literature review of currently accepted best practice in transgender healthcare, after which, the author analyzes the key report leveraged by Florida’s Department of Health in its ban. A critical analysis of the report is followed by a secondary analysis of the key peer-reviewed article upon which the Florida Medicaid authors relied to make decision. The paper culminates with a summation of the trajectory of anti-transgender misinformation.

Conclusions:
Misinformation plays a key role in producing legislation harmful to transgender people. Medical and Health Science Librarians have a role to play in identifying misinformation as it flows through the Misinformation - Legislation Pipeline and enacting key practices to identify, analyze, and oppose the spread of harmful misinformation.

Disaster Informatics Over Time: A Bibliometric Study from 2016 to 2022

Track(s): Information Management

Haihua Chen, University of North Texas

Ngan Tran, University of North Texas

Ana Cleveland, Regent Professor and Director of Health Informatics Program, University of North Texas

Objectives:
To analyze the disaster informatics literature regarding publication productivity, authorships, and collaborative research patterns between 2016 and 2022.

Methods:
A bibliometric study was conducted to address seven research questions, exploring the number of publications, languages, authorships, publication sources, the collaboration patterns among countries, authors, and institutions, and the keywords used in the literature before and after the pandemic (until 2022). A search was conducted on PubMed Central, Web of Science, and Scopus from 2016 to 2022 using the terms: “disaster informatics”, “crisis informatics”, and “pandemic crisis”. Data cleaning was conducted using Python and human annotators. Duplicates, non-English documents, and documents without an abstract and authors were removed. The data analysis phase involved descriptive analysis, word cloud visualization, and bibliometric analysis – VOSViewer.

Results:
After processing, the pre-pandemic publications (2016-2019) were 148, while the post-pandemic publications (2020-2022) reached 10,850. The number of publications increased in 2020 and peaked in 2022. English was the only language of pre-pandemic publications, thereafter 25 additional languages were represented. The United States was the leading contributor before and after the pandemic. The collaborations among authors, countries, and institutions expanded post-pandemic. The main sources of publications changed from conference proceedings (pre-pandemic) to journals (post-pandemic). The keywords changed from crisis and social media (pre-pandemic) to COVID-19 and pandemic (post-pandemic).

Conclusions:
Overall, COVID-19 had an impact on the disaster informatics literature (2020-2022). Publications on disaster informatics sharply increased in 2020 and after. The language of publications and collaboration among

countries indicated a global approach to the field of disaster informatics after the pandemic. Collaborations among authorships, institutions, and countries showed an increase in productivity in this field. The most active countries in contributing to the post-pandemic literature were among the highly impacted by COVID-19. Changes in the keywords that were used in the pre-pandemic and post-pandemic literature indicated a shift in the focus of the field.

Discovery Systems and Academic Health Sciences Libraries 2015-2024

Track(s): Information Management, Information Services

Hal Bright, University Library Director, A.T. Still University

Objectives:
In the last decade, what are the discovery tools used by (Academic Health Sciences Libraries) AHSLs and at what frequency at which they are deployed? What are the attitudes of Library Directors towards these tools and have these attitudes changed over the past decade?

Methods:
We identified 158 AHSL (Academic Health Sciences Libraries) institutions from the annual AAHSL (Association of Academic Health Sciences Libraries) survey and the AAHSL member web site. Several data points were collected in 2013 and updated in 2018 from each AHSL site, the use/nonuse of a discovery tool being one. We will update this data in 2024 and present the results discussing trends inside this field. A survey instrument developed in late 2018 will be modified and sent via Qualtrics to all AHSL library directors in 2024 to measure attitudes toward discovery tools. Separate tracks with similar questions will be used for institutions that have and do not have discovery tools. We will identify themes and attitudes of these directors towards discovery tool use in academic medical libraries.

Results:
Not available

Conclusions:
Not available

“Do I Really Need to Work With a Librarian?”: Exploring the Reporting and Reproducibility of Comprehensive Reviews Published on an Academic Medical Campus

Track(s): Information Services

Christi Piper, Research Informationist, University of Colorado Anschutz Medical Campus Strauss Health Sciences Library

Ellie Svoboda, Education Informationist, University of Colorado Anschutz Medical Campus

Hannah Pollard, Scholarly Communication Informationist, University of Colorado Anschutz Medical Campus

Kristen DeSanto, Clinical Informationist, University of Colorado Anschutz Medical Campus Strauss Health Sciences Library

Ben Harnke, Research Informationist, University of Colorado Anschutz Medical Campus Strauss Health Sciences Library

Objectives:
Many systematic review guidelines (e.g. Institute of Medicine Finding What Works in Health Care) state that a librarian should be included in the project team to assist with the search strategy, at a minimum. On our academic medical campus we find that many reviews do not include a librarian in the project team, despite the availability of a search service. We anticipate that the lack of a librarian is affecting the quality and reproducibility of published reviews from our campus. To validate this hypothesis, we reviewed the reproducibility and reporting of reviews with authors from our campus published in 2022.

Methods:
Ovid MEDLINE ALL was searched using an institutional search hedge and the CADTH Systematic Reviews and Meta Analysis and Guidelines search hedges, modified to include scoping reviews, meta syntheses, rapid reviews, umbrella reviews, and living reviews, without date limitation. Covidence was used to remove duplicates and for screening. Title/abstract screening was done to identify appropriate reviews. Included citations were moved into EndNote 21, divided by publication year, and full text was retrieved. Reviews published in 2022 were moved back into Covidence for full text review by two independent reviewers with disagreements resolved by discussion with the entire author group. Reviews that had methodology guidelines (e.g. systematic reviews but not narrative reviews) and included a campus author were included for data extraction. Two independent researchers completed data extraction and quality appraisal for the first forty citations. Once consensus was reached about extraction elements, individual researchers completed extraction and data was confirmed by the primary researcher (CP). Data extraction included elements like campus author name and affiliation, presence of librarian author or assistance, protocol registration, databases used, etc. Quality assessment was focused on elements of the search methodology that directly align with librarian’s roles in reviews using the

Results:
The Ovid MEDLINE search returned 4127 citations for reviews from all years. Covidence identified 19 duplicates, leaving 4108 unique citations for review. 1777 citations were identified for full text review. 285 of those citations were published in 2022 and included for this portion of the project. 84 citations were excluded during full text review, leaving 201 for data extraction and quality assessment. Results are still forthcoming, but we plan to report on percentage of studies that included librarian involvement (and degree of involvement), an overview of PRISMA adherence, and PRESS-assessed quality of the search strategy used.

Conclusions:
With the demand for systematic review assistance increasing, it is important to know how the involvement of a librarian on these projects impacts the quality of the reviews being published. For our campus, in particular, we anticipate using the results of this study to advocate for better systematic review practices and increased instruction about best practices. We do not have the staffing to assign a librarian to every review project done on our campus, but we can use these results to identify areas of weakness in searches and reporting to create targeted instruction and support.

Does Medical Terminology Support Equity? It’s Not What We Say, It’s How We Say It

Track(s): Education, Health Equity & Global Health

Gisela M. Butera, Biomedical Librarian, National Institutes of Health (NIH)

Cynthia Sheffield, Biomedical Librarian, National Institutes of Health Library

Dera Tompkins, Biomedical Librarian, National Institutes of Health Library

Corina Galindo, Program Specialist, NIH

Deborah Duran, National Institute of Minority Health and Health Disparities

Kimberly Middleton, Principal Investigator, NIH

Vence L. Bonham Jr., Acting Director of the National Human Genome Research Institute, NIH

Objectives:
Health disparities currently is a major focus in health science research, however inadequate terminology poses challenges to identify appropriate literature. Three experienced librarians well versed in literature searching explore how MeSH and Emtree terminology structures might impact literature discovery. This paper will examine the organization, structure, and volume of terminology used within the literature surrounding maternal health care, with a specific focus on the terminology related to health care access, affordability, preventive health, and navigation of health care systems. The study’s objective is to identify how indexed terms may be influencing research within health disparities.

Methods:
This investigation will use sample questions about ‘maternal health care’ to identify literature. Search strategies will be used to explore four sub concepts within maternal healthcare: 1) health disparities, 2) health care access, 3) health care system/delivery of health care, and 4) preventive medicine.

An evidence scan will be conducted, and an analytical framework will be developed and reviewed by preventive health and health disparities subject matter experts to confirm a comprehensive study design. PubMed and Embase databases will be searched, and records screened in Covidence. Relevant articles will be examined quantitatively and qualitatively. Studies will be assigned to one of the four subcategories, most appropriate for the context of that article. Thesauri terms from each of the concepts will be quantitatively analyzed in terms of how often a thesauri term was used for an article. Titles and abstracts from citations will be used to identify new terms, phases or variant terms used to describe the literature, currently not within the thesauri. Existing and emerging terms will be used to determine whether abstracts from the citations mention “health care disparities”, “health care administration” or both concepts, as factors contributing to patient care in each sub concept.

Results:
The evidence scan results will be presented in a flow diagram. A map of tree structures will be used to display the frequency of terms used within the literature, and their relationship. New terms will be presented in a similar fashion. The qualitative analysis of abstracts will show the frequency of focus in each article, in terms of “disparities” vs. “administration” vs. both. The focus frequency measures will be presented along with observations about how each controlled vocabulary is meeting the needs of investigators who need to describe patient populations and the health systems designed to care for them.

Conclusions:
Authors will present the study’s findings showing potential challenges of existing terminology within the literature describing the maternal health care environment. Maternal health care is a universal indicator of health. This analysis is designed to understand if there are challenges unique to specific populations or geographic areas. Authors will identify and report what inferences can be drawn to improve health disparities economic terminology as well as suggested changes to be made to indexing terms in databases to help medical librarians when searching for literature.

Donor Weight Criteria in US Body Donation Programs: Website Analysis, Results, & Next Steps

Track(s): Health Equity & Global Health, Innovation & Research Practice

Heidi Reis, Liaison Librarian to the Brody School of Medicine, East Carolina University

Soph Myers-Kelley, Laupus Library, East Carolina University

Jenna Hagerty, Assistant Professor, Thomas Jefferson University

Kerri Flinchbaugh, East Carolina University

Kerry Sewell, Research Development Director, East Carolina University

Malli Barremkala, Oakland University

Marisa Langton, Medical Student, Drexel University College of Medicine

Maureen Helgren, Quinnipiac University

Rebecca Pearl, Associate Professor in the Department of Clinical and Health Psychology in the College of Public Health and Health Professions, University of Florida

Sophie Orr, UC Davis

Objectives:
A cross-institutional team of anatomists, public health and medical school faculty, and library faculty seek to determine the extent to which body donor restrictions—listed on the websites of U.S.-based body donation programs—reflect U.S. population norms for BMI, along with the extent to which explanatory information is provided for any explicit or implicit weight criteria described on body donation program websites. With this exploratory research, the authors will evaluate the public facing, online body habitus-focused (weight, height and other body composition descriptors) body donation criteria across the US to notice any trends/areas for improvement related to weight bias.

Methods:
Definition: Body Donation is the process where people can donate their whole body upon death for educational dissection in a gross anatomy laboratory class.

Information on possible weight bias was procured by assessing 133 US body donation program websites for weight- and height-related terminology, with a focus on body donation criteria and exclusion justifications and recording the data in a secure Redcap survey. Results from the Redcap survey of body donation program websites were exported as a CSV file and uploaded to GoogleSheets for data cleaning. Data cleaning involved changing website survey responses with nominal data to 1 or 0, with responses changed to 1 if the survey response was yes/present for a question or 0 if the survey response was was no/not present for a question, to allow a frequency analysis. Survey responses were double checked by a second reviewer for accuracy, by viewing the body donation website and confirming the survey responses provided for the website were correct. Visualizations contrasting height/weight limits, implicit and explicit weight restrictions, and weight restrictions in pounds and BMI with US averages were generated using Prism (https://www.graphpad.com/features)

Results:
Weight criteria are commonly included in body donation criteria. Upper weight criteria are listed more frequently than lower weight criteria. Height is mentioned with the lowest frequency among body habitus criteria for donation. Upper weight criteria are described more frequently than lower weight criteria, both explicitly and implicitly. BMI restrictions clustered around the average BMI for the current U.S. population, while upper weight criteria, when not differentiated by sex, were clustered at or above the current U.S.

average weight (interquartile range: 200-250 pounds). Justifications for the body habitus criteria are rarely provided on body donation websites.

Conclusions:
Anecdotal information from anatomists indicates that upper weight criteria for body donation programs may be related to affordances of the equipment and storage available to the program and that changes to the equipment may constitute exorbitant expenses. However, the reasoning is typically not explicitly provided through websites. Therefore, the authors next steps involve surveying program personnel to assess the reasoning behind these restrictions, the frequency of website/policy updates, and interest in participating in interviews. The impact of the lack of explanatory text accompanying explicit and implicit weight criteria for potential donors not meeting criteria, or their family members, cannot be elucidated here, but would also be worthy of further study.

An Empirical Study Evaluating ChatGPT’s Performance in Generating Search Strategies for Systematic Reviews

Track(s): Information Services, Innovation & Research Practice

Rebecca Carlson, Eshelman School of Pharmacy Librarian, University of North Carolina, Chapel Hill

Heather Kincaide, University of North Carolina, Chapel Hill

Fei Yu, Assistant Professor, School of Information and Library Science, University of North Carolina at Chapel Hill

Objectives:
With the advent of Generative Artificial Intelligence (AI) technologies, such as ChatGPT, there is an interest in applying these tools to academic and research endeavors. Systematic reviews demand rigorous and exhaustive search strategies. This study seeks to empirically assess ChatGPT’s capabilities in formulating effective search strategies tailored to the needs of systematic reviews.

Methods:
Following guidelines from the Cochrane Handbook for Systematic Reviews of Interventions, we recreated the typical librarian’s systematic review search process for use with ChatGPT. This encompasses five primary tasks:

1. Formulating a research question based on research objectives: specifically, conducting a systematic review on the application of ChatGPT and other Generative AI technologies to support systematic review processes.
2. Directing ChatGPT to derive synonyms of concepts and generate appropriate Medical Subject Headings terms.
3. Tasking ChatGPT with the creation of a search strategy suitable for comprehensive database searches.
4. Prompting ChatGPT to recommend suitable bibliographic databases for systematic search.
5. Requesting ChatGPT to customize the search strategy in line with each recommended bibliographic database.

Our study will contrast the responses produced by the free version, ChatGPT 3.5, and its paid counterpart, ChatGPT-4. Since developing search strategies is a dynamic and iterative task, we plan for two rounds for each ChatGPT version. Following the initial round, expert searchers will evaluate the results’ quality using Peer Review of Electronic Search Strategies (PRESS) guidelines. Feedback from this first evaluation will shape more refined prompts for the second iteration. A final evaluation will be constructed to gauge ChatGPT’s effectiveness.

Results:
Results will be include (1) a detailed breakdown of the PRESS evaluation for ChatGPT generated search strategies, (2) a performance comparison between ChatGPT-3.5 and 4 regarding five tasks undertaken, and (3) feedback and recommendations from expert searching librarians on the use of ChatGPT for systematic review searching tasks.

Conclusions:
We will discuss the overarching findings from the study and suggest implications for future research and recommendations for the practical applications of the utilization of ChatGPT in systematic review processes.

Evaluating Tools/Instruments for Assessing YouTube Videos on Surgical Procedures for Patient/Health Consumer Education: A Systematic Review

Track(s): Clinical Support, Education

Amy Lin, Medical Student, Oakland University William Beaumont School of Medicine

Misa Mi, Director of Medical Library, Oakland University William Beaumont School of Medicine

Manasa Pavuloori, Oakland University, William Beaumont School of Medicine

Objectives:
How effective are the tools/instruments used to evaluate YouTube videos regarding surgical patient and health consumer education?

Methods:
In June 2023, a comprehensive literature search was conducted on PubMed, PsycINFO, CINAHL, and Scopus. Primary studies with empirical data that evaluate English YouTube videos to educate patients about surgical procedures in all specialties were included. Two reviewers independently completed title/abstract and full text screening, and data extraction in duplicate. The data extracted includes the number of videos evaluated, assessment tools, outcomes of significance, specific objectives, and features examined.

Results:
A total of 41 studies were included for review. The most commonly used tools were DISCERN (21), Global Quality Scale (11), JAMA (11), and 23 articles used a unique assessment instrument. Of the total studies included, 88% of the articles determined that patients were not adequately educated by YouTube videos per the ratings of the assessment tools, and 19 out of 41 articles mentioned that videos from professional sources were most useful.

Conclusions:
This systematic review reveals that the educational qualities in YouTube videos are substandard. Patients should be cautious when relying solely on YouTube videos for medical guidance. Surgeons, primary care providers, and health information professionals are encouraged to direct patients to high-quality patient education sources and create accessible medical content. As there is variability in the quality assessment tools used for evaluation, a standardized approach to creating and assessing online videos would improve patient education.

Evaluation of Assessment Tools Used for Judging the Quality of Online Patient Educational Information on Cardiovascular Diseases

Track(s): Clinical Support, Education

Amy Lin, Medical Student, Oakland University William Beaumont School of Medicine

Misa Mi, Director of Medical Library, Oakland University William Beaumont School of Medicine

Objectives:
The objective of this research is to evaluate the assessment tools used for judging the quality of online patient educational information on cardiovascular diseases.

Methods:
A comprehensive literature search was conducted with PubMed, ERIC, PsycINFO, and Scopus using subject headings and keywords representing concepts of CVD, psychometrics, Internet, and consumer health information. Primary studies were included if they were published between 2013 and 2023, focused on evaluating the quality of web-based text educational materials on CVD, freely available on the internet, and containing evaluation tools. Title and abstract screening followed by full-text article selection were conducted to select the final set of articles for inclusion. Data charted from selected studies included names of evaluation tools and criteria used for judging the quality of web-based resources (credibility, readability, content accuracy, understandability, accessibility, currency). Data was synthesized and the use of measurement tools and criteria were analyzed for frequency and mean.

Results:
Of the 22 studies included, 16 articles used assessment tools to evaluate the quality and reliability of CVD online materials; the most popular tool used is DISCERN Instrument followed by HONcode Certification and JAMS Benchmark criteria. Nineteen articles analyzed readability of information, and the most frequently used assessment tools are the Flesch-Kincaid Grade Level and the Flesch Reading Ease Score. The mean reading grade level of articles from each individual topic was 11.24. Only 1 study included a tool to assess understandability and actionability, 2 articles assessed accessibility, and 1 article assessed cultural sensitivity.

Conclusions:
A variety of tools was used to evaluate the quality of online patient education materials on CVD. Most materials assessed on CVD topics have poor credibility and are written well above the sixth-grade reading level suggested by the American Medical Association and the U.S. Department of Health and Human Services. This review sheds light on different assessment tools used to evaluate online patient education materials and reveals the quality of these materials. Content creators and clinicians should employ quality assessment tools prior to publishing to ensure readable, trustworthy information for patients and families.

Evaluation of Empathy Development Using Graphic Novels in a Medical Humanities First-Year Seminar

Track(s): Education

Brittany Heer, Health Sciences Librarian, Butler University Libraries

Susanna Foxworthy Scott, Assistant Professor, Butler University Department of Health Sciences

Objectives:
Cultivating empathy is an essential component of a liberal arts education, especially for those pursuing healthcare professions as it is well documented that empathy levels drop during residencies and fellowships. The medical humanities first-year seminar (FYS) at a private liberal arts university aids students in fostering a deep understanding of the humanistic dimensions of healthcare through the usage of a diverse range of reading materials. This study seeks to explore the efficacy of graphic novels as a tool for enhancing empathy, gauging the development over the first year of a college course.

Methods:
Utilizing the Toronto Empathy Questionnaire (TEQ), a pre-test consisting of 18 questions was administered to a cohort of 18 first-year undergraduate students enrolled in the university's FYS Medical Humanities course at the beginning of the academic year. Following each assigned graphic novel, 3 open ended questions were administered, totaling 4 throughout the course. A post-test TEQ was administered in addition to 3 open-ended questions at the end of the academic year. A mixed methods approach was used for data collection, allowing for a broader understanding of empathy development.

Results:
At the time of this proposal, the study is being conducted and yet to be analyzed. The post-test will be administered at the end of the 2023-2024 academic year (late April/early May 2024) allowing for initial results at the time of the conference. The authors anticipate that there may be a modest increase in empathy development when analyzing both the quantitative data demonstrated in the TEQ pre- and post-tests and the qualitative data revealed in the open-ended survey questions following each graphic novel.

Conclusions:
Empathy development is essential for future healthcare professionals. Introducing graphic novels as unique tools for enhancing and developing empathy in a first-year undergraduate seminar is one novel approach for preparing undergraduates for the reality of caring for and relating to their patients in their future healthcare careers.

Evidence Updates: Using a Performance Comparison to Evaluate Four Distinct AI-Assisted Citation Screening Tools

Track(s): Information Management, Innovation & Research Practice

Jing Su, Vanderbilt University Medical Center

Mallory Blasingame, Information Scientist, Vanderbilt University Medical Center

John Clark, Senior Application Developer, Center for Knowledge Management, Vanderbilt University Medical Center

Nunzia Giuse, Vice President for Knowledge Management; Professor of Biomedical Informatics; Director, Center for Knowledge Management, Vanderbilt University Medical Center

Taneya Koonce, Deputy Director, Vanderbilt University Medical Center

Jerry Zhao, Vanderbilt University Medical Center

Objectives:
Maintaining evidence syntheses poses a challenge for information scientists due to the need to complete labor-intensive filtering of citations published since the original search. To speed this process, our team developed a semi-automated approach to rank and facilitate screening of new citations based on relevance using two artificial intelligence (AI) components: an internally-developed rules-based algorithm and an open-source natural language processing tool (ASReview). The goal of this study was to assess performance and workload reduction of our semi-automated approach and compare it to three AI-assisted systematic review screening tools (Abstrackr, Colandr, Covidence) currently used for evidence updates.

Methods:
In a previous study, we evaluated the performance of our semi-automated approach compared with manual screening. In this study, we extended the analysis to three other AI-assisted screening tools (Abstrackr, Colandr, Covidence), which sort citations by predicted relevance based on the reviewer’s previous screening decisions. First, we exported all new search results for ten topics through September 1, 2023, and manually labeled the relevance of each new citation to use as the reference set. We established training sets, methods for marking relevance, and stopping rules for each AI tool to ensure maximum consistency while also leveraging their distinctive features.

Unique to our approach is the ability of the information scientists to capture, through metadata, their knowledge, that, in turn, is then leveraged by the rules-based algorithm to generate an initial ranking of the citations. The top and bottom citations from this list are then used to train ASReview. This empowered, throughout the process, the information scientists; the same cannot be said for the tools we used for our comparison. Performance metrics, including sensitivity, specificity, false negative rate, proportion missed, and work saved over sampling were calculated for each of the tools with the manual screening set as the reference set.

Results:
Results forthcoming.

Conclusions:
Conclusions forthcoming.

Expert-Recommended Tasks for Hospital Librarians During a Healthcare System Merger or Acquisition: An e-Delphi Consensus Statement

Track(s): Professionalism & Leadership

Jaclyn Morales, Senior Librarian, North Shore University Hospital

Stacy Posillico, Senior Librarian, Northwell Health

Objectives:
Little empirical research is available to guide hospital librarians through the healthcare system merger or acquisition process. In order to address this knowledge gap, a literature review and the e-Delphi research method were used to develop expert-driven recommendations to prioritize those tasks that librarians should undertake when consolidating the delivery of library services to a newly merged, geographically distributed healthcare system.

Methods:
A modified e-Delphi study was conducted and reported according to the Guidance on Conducting and REporting DElphi Studies (CREDES). The expert panel, composed of 29 hospital librarians and library professionals, responded to four rounds of questionnaires during April to December 2022. In the first round, they identified tasks that librarians should undertake during the merger process. Tasks were then eliminated or prioritized in Rounds 2 through 4, based upon the experts’ rating of each task using a seven-point Likert scale in which 1 equaled “not recommended” and 7 equaled “essential recommendation.” Those tasks rated as either 5, 6, or 7 by ≥75% of the panelists at the conclusion of the round were included in the final statement of recommended tasks.

Results:
A consensus-based statement of 329 recommended tasks for librarians to consider during a healthcare system merger or acquisition was created. The final set of recommended tasks were grouped into four main domains: Healthcare Organization Tasks, Library Collections & Information Systems, Library Administration, and Library Staff Integration & Interconnection. Tasks related to information technology and services, vendor relations, and library organizational structure were more likely to be prioritized than tasks related to marketing and outreach and the physical library space. Expert insight highlighted the importance of understanding context and culture when undertaking any recommended task.

Conclusions:
The recommended tasks can be immediately used by hospital librarians to create an action plan for consolidating and delivering library services in the event of a healthcare system merger or acquisition. Future research on the utility of the recommendations is needed.

An Exploration of Basic/Life Science Information Professionals: Educational Background, Liaison Roles and Carnegie Classification

Track(s): Information Services

Jeremy Kupsco, Research Informationist, Emory University

Laura Lipke, Health Science Librarian, Binghamton University

Stephanie Schulte, Director; Professor, The Ohio State University

Margaret Hoogland, Clinical Medical Librarian, Mulford Library, University of Toledo

Objectives:
Since the early 2000’s and the explosion of electronic information resources, information professionals have been struggling to re-connect to the Basic Science community. The information profession has expanded its roles beyond collection development to knowledge regarding the data service and research needs of this specialized community. As late as 2022, literature acknowledges that the outreach struggles continue and that this population is still unaware of the “capabilities of a science liaison librarian”. Scholarship has supported the ability of information professionals to fulfill the roles of training and instruction.

Methods:
The purpose of this study is to identify differences in collaboration between information professionals with and without degrees or training in basic science who work with the basic science community. The study will also examine whether Carnegie classification of the institution impacts information professionals' interactions. Participants for this study were identified via their institutional websites as well as through the email lists for the Basic Science Caucus of the Medical Library Association, the Science and Technology

Section of the American Library Association, the Health Sciences Special Interest Group of the Association of College and Research Libraries and the STEM Librarians Collaborative on Discord. They were recruited via direct email. The study used semi-structured interviews via Zoom to gather data. This data will be analyzed thematically. In addition, basic demographic information from the recruiting survey along with publicly available data about their institutions will provide context for addressing the research questions.

Results:
To date, the team has interviewed fifteen Basic Science librarians from various backgrounds and institutions and transcription of the recorded Zoom interviews has begun. A few noted emerging themes are that librarians that attended Basic Science program events were more successful in building relationships, most liaison skills for these programs were learned on the job, many reported that scientific intellectual curiosity is a skill needed to be successful and that librarians with a science degree were able to connect with these programs because they spoke "their language". A detailed thematic analysis of the results will be provided at the presentation of this study.

Conclusions:
Based on the initial findings of this study, it appears that liaisons with a Basic Science degrees do have an advantage, when connecting to the Basic Science community. More importantly, though, is for the liaison to look for opportunities to interact/support grant writing, research and sponsored programs and to demonstrate curiosity regarding the research of these programs. Further thematic analysis and findings regarding the Basic Science liaison librarians role with this population, and if Carnegie classifications play a role in successful collaborations will be presented.

An Exploratory Inquiry of the Trends and Practices of Using AI Tools in Systematic Reviews and Their Adherence to Methodological Quality Guidance

Track(s): Information Management, Information Services

Casey Phillips, Biomedical Libarian, University of Kansas Medical Center

Prasanna Vaduvathiriyan, Research & Learning Librarian, University of Kansas Medical Center

Objectives:
To identify the frequently used artificial intelligence (AI) tools in systematic reviews (SRs) and the methodological quality adherence in the review process.

Methods:
Two biomedical librarians in an academic medical center, whose primary role is education and research, will review the trends and practices of using AI tools in SRs. Based on a search protocol, they will conduct a comprehensive search in major bibliographic databases to identify frequently used AI tools in systematic reviews published between January 2020 and April 2024 in the English language. The search results will be exported to Covidence software for screening and identifying the frequently used AI tools. Selected reviews will be further analyzed to identify the methodological quality and best practice recommendations by PRISMA, Cochrane, and JBI. The focus areas of methodological quality analysis include adherence to study protocols, research questions, screening, use of quality assessment tools, and review reproducibility. Along with the quality review, they will also list the selected benefits and drawbacks of using AI tools in SRs.

Results:
The librarians will continue the evaluation until April 2024, when the most up-to-date results will be shared.

Early results show that while some AI tools may save time with some tasks of the review process, AI still has limitations and gaps that do not cover their methodological quality.

Conclusions:
If used carefully, the rapidly evolving AI tools are beneficial to librarians in saving time and workload in the review process. However, the human decision-making process in each step of the process is important and weighs more compared to the AI tools.

From Instruction to Health Sciences Librarianship: Coming Together to Support Career Fluidity

Track(s): Education, Professionalism & Leadership

Molly Maloney, Pharmacy Liaison Librarian, University at Buffalo

Jocelyn Swick-Jemison, Data Services Librarian, University at Buffalo

Nicole Thomas, Undergraduate Nursing Liaison Librarian, University at Buffalo

Emily Carlin, Hospital Circuit Librarian, WNY Library Resources Council

Objectives:
The objective of this study is to identify how a background in instruction librarianship impacts recruitment and retention into health sciences librarian positions by investigating the following questions: What barriers and supports have impacted instruction librarians of at least five years transitioning to health sciences librarian positions? How do core instruction skills and teaching experience impact this transition? What do librarians who experienced these transitions identify as central to the success of onboarding in these types of roles? We hypothesize that individuals who made this transition relied upon their instruction experience and skills as a foundation while building discipline-specific skills.

Methods:
We intend to survey health sciences librarians who have transitioned from an instruction to health sciences librarian role after at least five years of professional employment. This will be followed by a semi-structured interview to elicit further details on experiences and perceptions. Participants will be recruited via membership listings from the Association of Academic Health Sciences Libraries (AAHSL) in the US and Canada as well as by emailing Medical Library Association (MLA) caucus listservs. The survey will be administered using Qualtrics. Interviews will be conducted via phone or Zoom. Instruction librarian positions will be defined broadly as those whose primary job responsibilities are information literacy instruction, across any academic discipline and degree program, requiring pedagogical and assessment expertise. Health sciences librarian positions will be defined as those who offer library services directly to faculty, clinicians, staff, or students in a health sciences field in either an academic or hospital setting. These services may include but are not limited to instruction, reference, and data services.

Results:
Forthcoming

Conclusions:
Forthcoming

Health Sciences Cancellations: Lessons Learned

Track(s): Professionalism & Leadership

Leslie Gascon, Collection & Research Services Librarian, University of Washington Health Sciences Library

Objectives:
A large academic health sciences library collection supporting six health sciences schools and two medical centers was projected to be over budget. Thus, the goals of the 2021 collection review were to reduce spending and plan for inflation by canceling subscription resources. The collection librarian was tasked with accomplishing this however they saw fit.

Methods:
The collection librarian reviewed the budget, including future budget issues, and set a goal of canceling $160,000 worth of subscription resources in fiscal year 2022. Based on the coverage of journal packages and interdisciplinary value of combined resource packages, they decided to limit cancellations to single journal titles. The collection librarian selected 97 subscription journals worth over $130,000 to cancel in consultation with health sciences subject librarians, campus partners, and the institution's faculty, students, and staff.

Results:
When the cancellations were acted upon, the e-resources librarian discovered several resources were in packages that could not be canceled. In addition, the usage data and subscription prices were not accurate for every selection, resulting in last minute renewals. A few vendors were sympathetic and worked with the collection librarian to find an agreeable price point while retaining subscriptions to some of the planned cancellations.

Conclusions:
This collection librarian learned the hard way that one should check all data before analyzing it as well as work closely with other collection librarians to avoid canceling important, but unfamiliar, resources. While consulting health sciences faculty, staff, and students was an important part of moving toward a more involved and engaged community, it also introduced users to the library with a negative experience.

How Do Journals and Publishers of Highly Selected Journals React to Submissions of Artificial Intelligence Assisted Manuscripts?

Track(s): Education, Information Management

Misa Mi, Director of Medical Library, Oakland University William Beaumont School of Medicine

Lin Wu, Professor/Assistant Director for Research & Learning Services, The University of Tennessee Health Science Center (UTHSC)

Wendy Wu, Librarian IV, Shiffman Medical Library Wayne State University

Yingting Zhang, Research Services Librarian, Robert Wood Johnson Library of the Health Sciences, Rutgers, The State University of New Jersey

Objectives:
Generative artificial intelligence (AI) is having a profound impact on research and scholarly communication. Given the evolving application of ChatGPT and AI in scholarly communications, health sciences librarians have pressing needs for becoming cognizant of any existing journal publishing guidelines about manuscripts created or assisted with AI. The purpose of the study is to examine how scholarly journals and publishers respond to submissions of these manuscripts and what requirements or policies have been put in place in guiding and instructing authors on the AI use.

Methods:
We retrieved and consolidated a list of journals representing disciplines in biomedical /health sciences from Scopus, Journal Citation Report (JCR), Google Scholar (GS), and Directory of Open Access Journals (DOAJ). To consolidate and finalize journal titles for the study, we selected all journal titles overlapped among Scopus, JCR, and Google Scholar, and retained several journals unique to Google Scholar. Also included were the top 50 journal titles from DOAJ given the wide acceptance to open access journal publication in the current academic climate and the increasing influence of open access journals in the scholarly publishing arena. We reviewed journal authors instructions available on the Internet to identify any specific policy, requirements, guidelines, and terms, related to AI generated or assisted content. We created and piloted a standard data charting form. Using the revised charting form, two authors, independently and in duplicate, charted data on AI statement, permission of AI in manuscript writing, restriction on AI authorship and citation, and requirement of disclosure or acknowledgement of AI use in manuscripts. Charted data will be analyzed and synthesized in a dual process for any trends and emerging patterns of requirements regarding the use of AI in journal article submissions.

Results:
We identified the top 50 journals in medicine ranked by CiteScore from Scopus, the top 50 journals in Medicine, Biology and BioChemistry, by Journal Impact Factor from Journal Citation Report, the top 20 journal titles in Health and Medical Sciences in Google Scholar, and the top 50 journals in medicine from Directory of Open Access Journals. A final consolidated list of 25 journals was selected for the review. The process of data charting and synthesizing is underway, and the review results will be shared and presented at the MLA conference.

Conclusions:
Varying applications of AI chatbots have been emerging in different fields, one of which is scholarly communication. Given the uncertainty about the impact of AI and dire need for guiding principles in scholarly communications involving the AI use, it is necessary for health sciences librarians to keep pace with emerging AL requirements of scholarly journals. The results shared and presented will shed light on these requirements and help librarians better guide, support, and teach users about the judicious use of AI as well as any ethical ramifications of its application in users’ scholarly activities and output.

Improving PICO Search Strategies with Causal Inference Methods

Track(s): Innovation & Research Practice

Madelyn R. K. Glymour, Medical Librarian, Duquesne University

Objectives:
Defining the population of a PICO question too broadly or narrowly may result in misleading outcomes, as demonstrated by Simpson’s Paradox, a statistical phenomenon in which an association that holds in a general population is reversed in all subpopulations. In one classical example, open surgery is more

effective at removing kidney stones than percutaneous nephrolithotomy in both large-stone and small-stone patients, but PN is more effective in all patients combined. Causal inference methods resolve this paradox.

I wish to determine if a PICO question whose population is defined with these methods returns results that are more accurate to the patient.

Methods:
A directed acyclic graph depicts the causal structure underlying data. In the case of the kidney stone data, the causal structure is known: physicians were more likely to prescribe the more invasive but more effective treatment (open surgery) to patients with larger stones.

I applied the backdoor principle, which identifies whether a variable should be considered when determining the effect of a treatment on an outcome, to the kidney stone DAG to determine whether stone size should be included in the population.

I will run searches in PubMed and Embase that do and don’t account for stone size, using keywords and controlled vocabularies. Although stone size is difficult to include in the search directly, strategies such as proximity searching on PubMed increase the likelihood of results that take stone size into account, and limiting the study type to RCTs with a validated filter returns results in which the causal influence of stone size is eliminated.

I will exclude results that are irrelevant to the PICO question or don’t provide data by stone size. I will examine the conclusion of each study, and determine whether it supports open surgery, percutaneous nephrolithotomy, or neither for a patient with a small stone.

Results:
The data is not yet compiled, but I would anticipate that results in which the majority of searches that do not account for stone size support percutaneous nephrolithotomy as more effective at removing stones, while the majority of searches that do account for stone size or limit to randomized controlled trials support open surgery as more effective, would reject the null hypothesis.

Conclusions:
This research will represent a first step in learning whether and how causal inference methods can be applied to medical librarianship problems. Future research may apply this strategy to different, more current clinical questions, or consider the implications this strategy might have for defining populations in systematic reviews.

In Their Own Words: Surveying GME Trainees on Meeting Their Program Requirements and Accreditation Milestones

Track(s): Information Services, Innovation & Research Practice

Emily Johnson-Barlow, Regional Health Sciences Librarian & Associate Professor, University of Illinois Chicago

Deborah Lauseng, Associate Professor & Regional Head Librarian, University of Illinois Chicago

Emily Gilbert, Assistant Professor and Liaison Librarian, University of Illinois Chicago

Tina Griffin, Associate Professor, Information Services and Liaison Librarian, University of Illinois Chicago

Holly Hudson, Asst Prof and Regional Health Sciences Librarian, University of Illinois Chicago

Objectives:
Graduate Medical Education (GME) programs are the bridge between freshly minted medical students and full fledged doctors. GME interns, residents, and fellows must reach certain accreditation milestones to demonstrate their understanding and mastery of their disciplinary practice. Librarians often address these needs per individual request, but may have limited opportunities to broadly consider the programmatic needs when planning library outreach, teaching, or resource acquisition. A survey of GME populations’ that is a part of a larger needs assessment (NA) study, will investigate library support opportunities that align with these milestones and uncover potential partnerships with various GME administrators

Methods:
We aim to survey our institutions' GME populations. This population makes up over 1,000 trainees spread across multiple campuses. Investigators constructed a 61-question instrument to address the trends and gaps uncovered in initial literature investigation. The questions are grouped by theme which includes feedback from GME trainees on relationships with the library/librarian, support needs related to scholarly projects and research, information needs and information seeking behavior in clinical settings, and motivations for learning. We will analyze results using descriptive statistics and thematic coding for open responses. We aim to code responses to specific milestone competencies to support our decision-making process in designing, implementing and evaluating library services.

Results:
The results and conclusion are not yet complete. The authors will update the abstract prior to the deadline.

Conclusions:
The results and conclusion are not yet complete. The authors will update the abstract prior to the deadline.

Influence from eHealth Literacy and Two Types of Online Health Information Seeking Behaviors on Health Behaviors among Diabetic Patients

Track(s): Innovation & Research Practice

Yue Ming, Research Support Librarian, Tulane University

Miriam Matteson, Professor, Kent State University

Objectives:
This study aims to examine the impact from eHealth literacy and two types of online health information seeking behaviors: intentional online health information seeking and incidental online health information seeking on two important health behaviors: shared decision-making and diabetes self-care among people who are diagnosed with diabetes from 18 to 44 years old in the U.S.

Methods:
Participants of this study are those who are in the U.S., diagnosed with diabetes and ages ranged from 18 to 44 years old. An online survey questionnaire was distributed through Qualtrics to participants after receiving Institutional Review Board (IRB) approval. Participants were recruited through Centiment (a third-party online survey platform) in March 2023. In the survey questionnaire, there are 6 items for intentional online health information seeking, 5 items for incidental online health information seeking, 8 items for eHealth literacy, 9 items for shared decision-making, and 4 items for diabetes self-care. For each measure, a Likert scale was utilized to indicate how much participants agree on each item or the frequency of each activity. Additionally, demographic variables including age, gender, race, education, general health, and number of types of information sources were also collected.

To analyze the data, we first conducted descriptive analyses and bivariate analyses among all variables. Next, linear regression analyses were performed to identify relationships between independent variables and outcome variables after controlling for all demographic variables. All analyses were conducted in R.

Results:
Results suggest that intentional online health information seeking ($B = .187$, $SE = .052$, $p < .001$) and eHealth literacy ($B = .493$, $SE = .051$, $p < .001$) are positively associated with shared decision-making. Additionally, incidental online health information seeking ($B = .166$, $SE = .056$, $p < .01$) and eHealth literacy ($B = .214$, $SE = .050$, $p < .001$) are positively associated with diabetes self-care. The overall model contributes to 33.1% variance of shared decision-making and 17.7 % variance of diabetes self-care.

Conclusions:
It is important and urgent for information professionals and public health practitioners to work together to effectively distribute accurate health information regarding diabetes, especially through online platforms. The study also reflected that different types of online health information seeking behavior could affect health behaviors differently among the target population. Moreover, eHealth literacy is a crucial factor to promote positive health behaviors. Information professionals such as health sciences librarians, health providers, and government agencies could consider collaborating to provide training programs and workshops on finding reliable health information or improving eHealth literacy. This could enhance the effectiveness of health information communication process and promote positive health behaviors.

Lasting Impact of COVID-19 on the Programs and Services Provided by Academic Health Sciences Libraries

Track(s): Information Management, Information Services, Professionalism & Leadership

Bart Ragon, University of Virginia

Melissa Rethlefsen, University of New Mexico

Elizabeth Whipple, Assistant Director for Research and Translational Sciences, Ruth Lilly Medical Library, Indiana University School of Medicine

Objectives:
This study examined the longitudinal impact of COVID-19 on Academic Health Sciences Libraries. Objectives include: Investigate the lasting effects on budgets and financial management. Analyze the evolving internal communication strategies. Identify and assess the evolving lessons learned from the influence of COVID-19. Evaluate the long-term impact on library facilities, management, and future planning. Explore the adaptations made in library services to meet the evolving needs of library patrons. Examine the enduring impact of the procedures and strategies associated with the reopening of libraries. Reflect on the impact of staffing dynamics and workforce management.

Methods:
This study used a multisite, qualitative data analysis approach to capture the direct experience of academic health sciences libraries as they evolved since the beginning of the pandemic. Academic health sciences library leaders from the 2020 – 2021 study were invited to participate in a focus group session to review the current state of their libraries and the ongoing impact of COVID-19 on the delivery of services and resources. The phenomenological approach used in this study sought to understand the experience of academic health sciences libraries longitudinally by gathering data about the state of libraries. The use of focus groups allowed the study to capture a broad range of perspectives from multiple organizations. During the focus groups, cognitive interviewing techniques were employed to elicit not just feedback about the current state of health sciences libraries but also to explore the context for how health sciences libraries have evolved since 2021. The qualitative data were coded to analyze the data and identify emergent themes.

Results:
Study is currently underway, results will be updated in early 2024.

Conclusions:
Study is currently underway, conclusions will be updated in early 2024.

Library-Led Interprofessional Education using NNLM grant funding examples

Track(s): Education, Innovation & Research Practice

Kay Strahan, Assistant Professor/Northwest Campus Librarian, University of Arkansas for Medical Sciences (UAMS)

Lindsay Blake, Clinical Services Coordinator, UAMS

Objectives:
Interprofessional Education (IPE) has become an increasingly important component of many health sciences colleges over the past 20 years. Since IPE classes focus on two or more professions learning with, about and from each other, subject matter can and should focus on topics important to all health sciences involved. Health literacy and consumer health information are two areas in which all health science students will need education and librarians can provide expertise. Librarian IPE courses are hypothesized to result in positive gains on evaluation in all colleges and provide valuable educational benefits to participants.

Methods:
A librarian developed and led IPE Quadruple Aim Project (QAP) course focusing on consumer health information was taught twice in the Spring 2024 semester to students in five health sciences colleges (Health Professions, Nursing, Medicine, Pharmacy, and Public Health). The IPE QAP project walks students through creation of SMART goals in the process of applying for grant funding. Librarians created brief scenarios based on three Network of the National Library of Medicine (NNLM) grant funding opportunities. Students in both IPE classes had the option of coming up with training ideas and creating SMART goals on two of the three options. The Interprofessional Collaborative Competencies Attainment Survey (ICCAS) instrument was used as a pre-test and post-test evaluation tool with additional activity specific questions included on the post-test. All ICCAS data from both sessions will be analyzed using SPSS for descriptive statistics and paired t tests to look for differences overall, by college, and by ICCAS domains. Activity specific post-test questions will be evaluated for themes and future course modifications.

Results:
Will be added, but it is expected that the librarian-led IPE activities will produce statistically significant changes based on the paired t tests from the ICCAS data. Additional post-session data will be reviewed to see what changes or updates could be made to improve the activity for the coming semester.

Conclusions:
Not available

The Library Value in LSU Health Shreveport Grant Associated Publication Citations

Track(s): Information Management

Julia Esparza, Director, LSU Health Shreveport

Ava Pollard, Student, Northwood High School

Elliott Freeman, Research/Writing Librarian, Louisiana State University Health Shreveport

Justin Smith, Allied Health Professions Student, Louisiana State University Health Shreveport

Leah Bryant, Student, Grambling University

Madison Coleman, Student, C.E. Byrd High School

Montie' Dobbins, Assistant Director Technical Services, Louisiana State University Health Shreveport

Prerana Ramesh, Medical Student, Louisiana State University Health Shreveport

Objectives:
There is a common anecdotal perception that “everything is free on the internet” and library resources are no longer needed. To date there is no study that analyzes the library's contribution to grant-associated publications. The purpose of this study is to measure the library's contribution to grant-associated publications by identifying the citations used and then the percentage available through library subscriptions when compared to the percentage that are available without subscription.

Methods:
The LSU Health Shreveport Library used the Tracking Accountability in Government Grants System from the United States Department of Health and Human Services to identify grant numbers associated with LSU Health Shreveport from 2015-2019. After eliminating duplicate listings, grants were sorted by Funding FY (fiscal year). Each grant number was searched in PubMed and Web of Science. The resulting grant-associated publications were searched in Web of Science and iCite, and reference citations exported to Excel. Citations were coded based on six questions: Did LSU Health Shreveport purchase the item the citation is in? Was the article electronically available without a subscription? If purchased, how was it accessible (electronic, print, or both)? What was the electronic vendor platform? Was there an identifiable embargo? If purchased, was the citation also freely available? Next, we calculated a per-article cost to determine library value. Due to license limitations on price sharing, we averaged the cost of ten randomly selected licensed journals to obtain that cost and applied that number to all purchased articles.

Results:
Our hypothesis was that 60%-75% of publications referenced in grant-associated publications would be licensed by the library. There were 34 grants for Funding FY 2015-2019. These grants generated 409

articles, which collectively referenced 27,657 citations. The library purchased 78% (21542) of the citations, 19% (5319) of items were electronically available without a subscription, and 3% (786) were inaccessible through electronic or print means, but could still be obtained through interlibrary loan. The final estimated dollar value of purchased and the percentage of those freely available even with paid subscription, is being compiled and will be added if abstract is accepted.

Conclusions:
The library provided access to 78% of the references for these grant-associated publications, while only 19% were available without subscription. The dollar value of the library contribution was LSUHS. A limitation is that we don’t know when articles were accessed. This means some of the citations counted as free may be available only through subscription. In addition, due to our averaging the per-article cost the dollar value identified may be higher or lower than stated. Still this data demonstrates the library’s clear value and supports the importance of library funding for a diverse and wide date range of publications to ensure that grantees have the resources they need to create grant-associated publications.

More Than the ABCs: Assessing the Information Needs of School Nurses

Track(s): Education, Information Services

Annie Nickum, Assistant Professor and Liaison Librarian, University of Illinois Chicago

Rosie Hanneke, University of Illinois Chicago

Objectives:
The objective was to determine the information needs of practicing school nurses in Illinois.

Methods:
CINAHL, LISTA, PubMed, and ERIC were searched for literature on the information needs of school nurses within the US and barriers to their professional development. The authors adapted a validated information needs survey on the needs of public-school nurses and conducted an in-state survey through the Illinois Association of School Nurses and the Illinois Department of Public Health school nurse list servs. Data was collected regarding the school nurses’ information preferences, behaviors, and barriers to searching and accessing this information. This was followed up with one-on-one interviews wherein participants were asked about their opinions on the survey findings and experiences searching for information in the course of a typical day. This data will be analyzed and combined with the survey findings to determine how librarians can best address knowledge gaps.

Results:
Our findings indicate that school nurses are an underserved population. They rarely have access to paid resources and are expected to seek out professional development in their own time with their own resources. The research suggests they want to learn but lack the time and resources to do so.

Conclusions:
School nurses need evidence-based information to provide quality services to their students with complex healthcare needs as well as addressing the public health needs of their school communities. The barriers to

finding, synthesizing, and applying relevant and scientifically sound information to practice include heavy workloads and limited time, lack of awareness of or access to evidence-based information, and limited searching skills. Medical librarians are well-poised to support the complex information needs of public-school nurses. Work is underway to follow-up the survey with one-on-one interviews. Once all data has been analyzed, an accessible intervention will be developed.

Onboarding Experiences: Through the Looking Glass of Early Career Health Sciences Librarians

Track(s): Information Services, Professionalism & Leadership

Kay Strahan, Assistant Professor/Northwest Campus Librarian, University of Arkansas for Medical Sciences (UAMS)

Lindsay Blake, Clinical Services Coordinator, UAMS

Caraline Annichiarico, Academic Health Sciences Librarian, UAMS

Objectives:
As health sciences libraries look to the future, one area of focus should be the onboarding of new hires. Onboarding includes not only basic orientation, but training in job duties, organizational socialization, access to mentors, and provision of tools, resources, and the knowledge to succeed in their positions. This project investigates the onboarding experiences of newly hired health sciences librarians through use of a survey and personal interviews. Special attention is paid to how health sciences libraries approach orientation, teaching of job duties, cultural integration, and introduction to faculty status.

Methods:
This study will use a mixed method design to quantitatively and qualitatively analyze the attitudes and perceptions of new health sciences librarians towards their most recent onboarding experiences. A survey is being designed at the researchers’ university with both new and existing faculty. The survey will focus on onboarding experiences of health sciences librarians and how these experiences have affected their career in positive and/or negative ways. After initial review of the pilot survey and incorporation of feedback from participants, the survey will be distributed to relevant health sciences listservs. Participants will be asked to submit names and emails if they are early career librarians who would like to further discuss their onboarding experiences in a structured interview format. Individual interviews will be arranged with 15-20 early career librarians who are within the first three years of their first professional health sciences librarian position after graduation.

Results:
N/A

Conclusions:
N/A

The Power of Networking: Creating a Stronger Medical Librarian Community in Uganda

Track(s): Health Equity & Global Health, Information Services

Onan Mulumba, Academic Librarian, Makerere University

Objectives:
To assess and demonstrate the transformative influence of networking and community-building initiatives among medical librarians in Uganda, emphasizing their role in addressing healthcare information gaps and improving knowledge dissemination. The study aims to highlight the specific benefits of these networks in facilitating the exchange of best practices, specialized expertise, and limited resources, ultimately contributing to the enhancement of evidence-based healthcare, research initiatives, and patient care within the Ugandan context.

Methods:
The study will employ a mixed-methods approach, involving qualitative data collection through in-depth interviews and focus group discussions with key stakeholders in the Ugandan medical librarian community, as well as a comprehensive review of existing literature. Quantitative data will be gathered through structured questionnaires distributed to a representative sample of medical librarians across different Ugandan regions, focusing on the impact of networking initiatives, resource sharing trends, and perceived improvements in knowledge dissemination and healthcare service provision. Additionally, observational analysis of networking events and community-building activities within the medical librarian community will provide insights into the dynamics of information exchange, collaborative efforts, and knowledge-sharing practices. This multi-faceted approach will facilitate a holistic assessment of the networking landscape and its pivotal role in fostering a stronger medical librarian community in Uganda.

Results:
The results of this study will offer potential enhancements to the Ugandan medical librarian community. Qualitative data is expected to reveal insights into networking strategies, challenges, and benefits. Quantitative findings may highlight improved knowledge sharing and resource access. Together, these data sources provide a comprehensive understanding of networking's impact, enhancing evidence-based healthcare and patient care. The study aims to provide practical recommendations for a stronger and more collaborative medical librarian community in Uganda, fostering improved knowledge exchange, healthcare services, and a resilient healthcare system.

Conclusions:
This study unveils the vital role of networking and community-building among medical librarians in Uganda. The anticipated results suggest that these collaborative efforts significantly impact the dissemination of healthcare information, knowledge sharing, and resource accessibility. This research aims to serve as a stepping stone for strengthening the medical librarian community in Uganda, offering practical recommendations to address unique healthcare information challenges and enhance evidence-based practice. By fostering a more connected and resilient community, this study envisions an improved healthcare landscape in Uganda, where robust knowledge exchange leads to better patient care and ultimately contributes to the development of a more effective healthcare system in the country.

Power through Agency and Empowerment: An Observational Study of Equity in Credentialing and Hiring Practices in Libraries

Track(s): Professionalism & Leadership

Jamia Williams, Assistant Librarian, University of Utah

Peace Ossom, Associate Director, Nat'l Center for Data Services, NYU Langone Health

Ayaba Logan, Associate Professor, Medical University of South Carolina

Christian Minter

Xan Goodman, Health Sciences Librarian, Associate Professor, University of Nevada, Las Vegas

Objectives:
The objective of this study is to evaluate the following research questions: (1) What is the percentage of libraries hiring librarians without an ALA-accredited master's degree? (2) Is there a significant difference between hiring of white professionals without an ALA-accredited master's degree as librarians in comparison with people of color? (3) Do the trends differ by library type and location?

Methods:
The team is releasing an IRB-approved questionnaire to academic libraries that are members of the Association of Research Libraries, medical libraries that are members of the Association of Academic Health Sciences Libraries, and all U.S. public and federal libraries. The questionnaire specified that respondents must be either the head of the library or a selected delegate to complete the form, such as an HR person. The respondent was then asked to quantify the number of librarians without an ALA-accredited master's degree and the number of those who identify as people of color, based upon their own self-identification through HR. R and RStudio will be used to analyze findings.

Results:
The study is currently ongoing. Results will be shared at the conference.

Conclusions:
The study is currently ongoing. Conclusions from findings will be shared at the conference.

Python Programming for Medical Librarians: Harnessing Data Analysis and Visualization

Track(s): Information Management, Innovation & Research Practice

Onan Mulumba, Academic Librarian, Makerere University

Joachim Ssenkaali, Lecturer, Lira University

Objectives:
To investigate and demonstrate the impact of Python programming in medical librarianship by analyzing its role in data management, analysis, and visualization, aiming to enhance the accessibility and utility of medical information, facilitate evidence-based practice, and improve healthcare research outcomes.

Methods:
The study will employ a multifaceted approach, including literature review, practical case studies, and the application of Python libraries such as Pandas, NumPy, Matplotlib, and Seaborn to exemplify the implementation of Python programming in data management, analysis, and visualization within the context of medical librarianship

Results:
The study expects to demonstrate that Python programming enhances data management and analysis for medical librarians. This is through practical cases employing Python libraries. It also envisions the creation of interactive data visualization resources to aid medical professionals and researchers. Incorporating Python into medical librarians' skill set is anticipated to bolster evidence-based healthcare practices and research, while addressing the challenges posed by growing medical data. Ultimately, the study aims to empower medical librarians to enhance healthcare outcomes and knowledge accessibility through Python's capabilities in data analysis and visualization.

Conclusions:
This study highlights Python's crucial role in medical librarianship, specifically in data analysis and visualization. The envisaged outcomes underscore its potential to streamline data management, offer valuable insights, and create engaging information resources. By integrating Python, medical librarians can advance evidence-based healthcare practices and research. This research signals a pathway for librarians to be proactive knowledge facilitators, contributing to improved healthcare outcomes and enhanced medical information accessibility in the data-intensive landscape.

A Qualitative Mixed Methods Exploratory Assessment of the Role of Alabama’s Public Libraries to Address the Opioid Crises

Track(s): Innovation & Research Practice

Bharat Mehra, EBSCO Endowed Chair in Social Justice and Professor, School of Library and Information Studies, University of Alabama

Misha Viehouser, Instructor, Department of Management, University of Alabama

Objectives:
The last decade has seen an acceleration of Opioid deaths making it a serious national public health crisis. Alabama has not been immune to increased age-adjusted drug overdose death rates, with limited Opioid health prevention, treatment, recovery, and relapse-prevention solutions. This paper reports on a qualitative mixed methods exploratory assessment of the role of Alabama’s public libraries to address the Opioid crisis. The emerging taxonomic framework, including Roadmap & Strategic Action Plan, provides public librarians and health information providers "how-to's" in operationalizing contextually relevant and culturally applicable strategies to address the Opioid crisis in the state.

Methods:
The following qualitative data collection methods provided feedback from library staff in Alabama’s public libraries about their experiences and perspectives: (a) An online survey (with open-ended questions) through Qualtrics provided rich responses from 36 Alabama’s librarian staff about their community engagement experiences and initiatives to address the Opioid crises, and what past and current role public libraries play in community health building processes and community health development; (b) Semi-structured narrative interviews and focus groups with fifteen state librarian staff yielded information about their role as community hubs to address the Opioid crises. Data analysis involved application of grounded theory and open, selective, and axial coding to both datasets.

Results:
Results include development of contextually relevant strategic information tools from the data collected (e.g., roadmap, action plan, programming taxonomy). Developed from the experiences/perspectives of Alabama’s library staff about their information offerings, initiatives and community engagement to address the Opioid

crises, details focus on type of activities, collaborators, experienced challenges, outcomes, potential actions, resources, and best practices. These have been shared with state library leadership to strengthen the role of Alabama’s 230 public libraries in Opioid-related community engagement, social justice, and inclusivity efforts in various facets of community health.

Conclusions:
A granular state-by-state approach highlighted in this research in its focus on Alabama is key (and can be replicated) to effectively address each region’s unique and complex socio-cultural, sociopolitical, socioeconomic, and socio-environmental problems surrounding the Opioid crises.

A Scoping Review of Librarian Involvement in Competency-Based Medical Education

Track(s): Education, Information Services

Amy Blevins, Associate Director for Public Services, Indiana University School of Medicine

John Cyrus, Research and Education Librarian, VCU Health Sciences Library

Molly Knapp, Training Development Manager, University of Utah

Laura Zeigen, Health Sci Ed/Research Lib, OHSU

Objectives:
The Association of Academic Health Science Libraries Competency Based Medical Education task force was assigned several charges. The primary charge is to “create a collection of case studies, vignettes, best practice stories, or other representations demonstrating the beneficial roles and positive impacts of librarian engagement in [CBME].” In discussing ways to accomplish this, members of the task force identified a gap in literature addressing the ways in which librarians are directly involved with CBME. In order to address this gap, we conducted a scoping review to answer the question: Is there demonstrable evidence of librarian involvement in competency-based medical education?

Methods:
This scoping review followed the Joanna Briggs Institute methodology and PRISMA-ScR reporting guidelines. A search of medical and library and information science literature databases was conducted in the following electronic databases up to December 31, 2022: Medline (Ovid), Embase (Ovid), ERIC (Proquest), CINAHL Complete (EBSCO), SCOPUS (Elsevier), and Library & Information Science Source (LISS)/Library Literature & Information Science (LLIS)/Library, Information Science & Technology Abstracts (LISTA) via EBSCO. Two reviewers screened each study for inclusion. Inclusion criterias included: described librarian involvement in the planning, delivery, and / or assessment of competency based medical education in LCME-accredited undergraduate medical education programs. Studies that were not published or available in the English language were excluded. Outcomes of interest included characteristics of the inventions (duration, librarian role, content covered) and characteristics of the outcomes and measures (level on Kirkpatrick Model of Training Evaluation, direction of findings, measure used).

Results:
Of the 11,051 studies screened for inclusion, 50 were included. Forty-six articles were empirical research or program evaluations, four were literature reviews. Included articles were published in eight journals. Two-thirds of the articles were published after 2010. Duration of interventions varied from 30 minutes to semester long. Librarians were often collaborators as opposed to leader, curriculum designer, or evaluator. The majority of studies covered asking and finding information for clinical questions and assessed reaction or

learning outcomes. Few studies assessed behavior and results outcomes. More than 95% of outcomes were positive; however, no study addressed clinical com

Conclusions:
This scoping review found that there is a strong base of literature on the involvement of librarians in CBME. However, few included studies assessed outcomes related to the application of knowledge or skills taught by a librarian or used validated measures as part of the research. When librarians are communicating their value to internal and external stakeholders, having landmark studies with demonstrable evidence of the contributions of librarians is essential. While librarians are publishing articles related to their involvement in competency based medical education, existing literature may not capture the extent of work done in this area. Additional research is needed to quantify the impact of librarian involvement in competency based medical education.

Seeking Solutions for Global Challenges: Furthering Global Librarianship with a UN SDG-Aligned Research Project

Track(s): Health Equity & Global Health, Innovation & Research Practice

Bethany Mcgowan, Associate Professor, Libraries and School of Information Studies, Purdue University

Objectives:
While the U.S. works to adjust to a rapidly changing information ecosystem, some EU member countries provide established examples of how governments can use policy to shape the information environment and protect the population from misleading communication, while respecting civil liberties. For this study, I focus on Finland. My research questions are: In Finland, how are policies or policy tools used to help balance the need between propagating accurate information, curbing dis/misinformation, and ensuring human rights of access to information? How can the successes and challenges associated with these policies be used to inform public policy efforts in the US?

Methods:
The World Health Organization Public Health Research Agenda for Managing Infodemics informed the development of the research question. The project methodology utilized a community-based participatory research (CBPR) approach. CBPR is a partnership approach to research that equitably involves community members in all aspects of the research process. CBPR was ideal for this project because of its usefulness in conducting rapid assessments, its helpfulness in the formative phases of research when little was known about the topic area, and its equal engagement of researchers and community partners. CBPR is an approach to research, not a specific method or research design. Thus, this project used the Delphi Method, a qualitative approach for generating quantitative results that involves gathering expert opinions through a series of progressive and iterative questionnaires to reach a consensus. 30 experts were recruited, from disciplines including public policy, public health, library science information studies, and education. Questionnaires were distributed via email, over two rounds to gain consensus. A literature review was also conducted. A decision on these specific research designs was based on a negotiation in which the researcher introduced methods, and community partners considered their practicality and contributed local expertise.

Results:
Policies related to health literacy are a focus of Finland’s national agenda. When the national core curriculum for basic education was introduced in 2016, the term health literacy was formally adopted to describe the teaching objectives and learning criteria for the subject of health education in grades 1–9. Including health literacy as a curriculum-based component ensures that all school-aged children can acquire

the competencies needed to promote and sustain their health and well-being. Relatedly, Finnish law requires that university-level studies in health education clearly approach and focus on health literacy principles and pedagogies during the teacher training program.

Conclusions:
According to a 2019 WHO regional report, Finnish students rated among the best informed about health in Europe, partly due to the Finnish approach of integrating health literacy instruction in public education. A new Finnish national literacy strategy, released in 2021, targets people of all ages. The goal is to identify models to promote literacy at local, regional, and national levels. Although the new strategy does not focus on health literacy explicitly, it discusses the importance of multiple literacies. As states in the U.S. begin to require K-12 instruction in information literacy--e.g., the passage of the New Jersey Information Literacy Standard in early 2023--a global librarianship perspective should inform librarian advocacy, outreach, and instruction efforts.

Strengthening Community Engagement: Collaborative Outreach Initiatives

Track(s): Information Services

Irene Nakazibwe, LIS Graduate student, Makerere University

Objectives:
This study will investigate the impact and effectiveness of collaborative outreach initiatives at Makerere University Medical Library in expanding service reach, improving service quality, and empowering the local community.

Methods:
This study will employ a mixed-methods research design, combining quantitative surveys to gather structured data, qualitative interviews to obtain in-depth insights, and case studies to analyze specific collaborative outreach initiatives at Makerere University Medical Library. The quantitative surveys will provide numerical data on the effectiveness of outreach strategies, while qualitative interviews will capture the perspectives and experiences of stakeholders involved in these initiatives. Additionally, case studies will offer a detailed examination of select outreach programs, allowing for a comprehensive understanding of best practices and challenges unique to the University Medical Library. This multifaceted approach will facilitate a thorough evaluation of the library's collaborative outreach efforts and their impact on service enhancement and community empowerment.

Results:
The prospective results of this study aim to reveal the effectiveness of collaborative outreach initiatives at the University Medical Library, highlighting successful strategies and potential areas for improvement. The study anticipates demonstrating the impact of these initiatives on expanding the library's service reach and enhancing service quality. Through analysis of quantitative survey data, qualitative interviews, and detailed case studies, the research seeks to provide actionable recommendations for optimizing future outreach efforts, thereby facilitating improved access to vital healthcare information and services for the community.

Conclusions:
This study demonstrates the effectiveness of collaborative outreach initiatives at the University Medical Library in expanding service reach and improving quality. It provides valuable insights into the experiences and perspectives of stakeholders involved, offering actionable recommendations for future efforts. Strengthening the library's connection with the community enhances access to crucial healthcare information

and services. This research contributes to the empowerment of individuals and the overall improvement of the healthcare information landscape.

Strengthening Our Understanding of Librarian Roles in Supporting Medical Image Consent Standards

Track(s): Information Management

Rachel Keiko Stark, Health Sciences Librarian

Sarah McClung, Head of Collection Development, University of California, San Francisco

Megan DeArmond, Jay Sexter Library, Touro University

Objectives:
The objectives of this research are to:

1.) build on the presentation that was done on the same topic at MLA ‘23,

2.) assess and understand health sciences library workers' current knowledge of best practices and recommendations for informed consent for the publication of medical images,

3.) and determine if and how knowledge of these standards informs the professional practice of health sciences library workers.

Methods:
The authors developed a survey to measure 1. Was the librarian aware of the informed consent standards for publishing medical images and 2. How they incorporate the standards into their professional responsibilities. The survey was tested for face validity and was approved through the IRB at one of the author’s institutions. The survey was distributed through multiple librarian listservs in December 2022. The initial hypothesis was that librarians were unaware of the standards as the authors did not find current literature on the topic in librarian-centered publications. Survey results were collected and the data was tabulated utilizing R.

Results:
This paper established that librarians, particularly those with responsibilities in instruction and collection development, are aware of the standards, but <50% of librarians with instruction responsibilities and slightly >50% of librarians with collection responsibilities incorporate the standards into their work. Librarians with digital initiatives responsibilities did not appear to be as aware and generally did not incorporate these standards into their work. The results indicate that there is a need for increased awareness of informed consent standards for medical image publication amongst librarians and more discussions about how to incorporate these standards into library work.

Conclusions:
This paper establishes a baseline from which further discussion and research can emerge regarding health sciences library workers’ roles, as resource purchasers and educators, in supporting standards for informed consent for the publication of medical images. With the strengthening of open science initiatives and advancement of computational analysis of large datasets, there is an increased need for resource creator accountability. History features many examples of unethical medical acts that disproportionately affected and continue to affect marginalized individuals. Awareness of how technology is radically changing the meaning of informed consent in the twenty-first century is an imperative first step towards improving professional practice and decreasing the likelihood that librarians are contributing to unethical behavior.

Textbook and Board Exam Prep Resources: Trends in Academic Health Sciences Libraries Serving College of Osteopathic Medicine Programs

Track(s): Information Management, Information Services

Joanne Muellenbach, Director, Health Sciences Library, California Health Sciences University

Lori Fitterling, University Library Director, Kansas City University

Kyle Robinson, Librarian Manager, Systems and Technical Services, California Health Sciences University

Harold Bright, University Library Director, A.T. Still University

Molly Montgomery, Assistant Director of Medical Education & Access Services, Indiana University School of Medicine

Objectives:
Our study aimed to identify the required and recommended texts and board exam prep resources used by U.S. Colleges of Osteopathic Medicine. Our primary research question focused on identifying the trends in using texts and board exam prep resources in academic health sciences libraries serving U.S. Colleges of Osteopathic Medicine Programs. A secondary research question aimed to identify which texts or exam prep resources are most prevalent and used by osteopathic medical students both within and outside of the formal medical school curriculum.

2. At what points during their medical education do osteopathic medical students use the texts or the exam prep resources?

Methods:
The study investigators consulted the list of medical schools from the American Association of Colleges of Osteopathic Medicine (AACOM) website to identify all U.S. College of Osteopathic Medicine (COM) libraries. The environmental scan of COM libraries' publicly facing websites initially started with collecting the library directors' contact details on an Excel spreadsheet, along with their lists of required and recommended textbooks, but after further review and reflection, we decided to include the lists of the board exam prep resources to which they subscribed.

Results:
The study investigators compiled a list of the library directors, their institutions, and contact information, and then developed an exhaustive list of the required and recommended textbooks. In addition, the investigators created a breakdown by top required and recommended texts, top subjects, top required texts in top subjects, top publishers, and characteristics of the required texts by year within the curriculum. Finally the investigative team compiled a list of the board exam prep resources, including the top resources that were identified and the percentage of College of Osteopathic Medicine (COM) institutions that subscribed.

Conclusions:
The study investigators found that there was some consistency in the top required textbooks and main textbook subjects across osteopathic schools, but there was considerable variability in texts lower on the list. Libraries and Colleges of Osteopathic Medicine vary in the range of board exam prep resources they provide and in who funds and supports those resources.

Phase Two:

Include the required and recommended textbooks that were identified, including a breakdown by top required and recommended texts, top subjects, top required texts in top subjects, top publishers, and characteristics of the required texts by year within the curriculum.

The 100 Top-Cited Articles in Osteopathic Medical Education: a Bibliometric Analysis

Track(s): Innovation & Research Practice

Harold Bright, University Library Director, A.T. Still University

Kyle Robinson, Librarian Manager, Systems and Technical Services, California Health Sciences University

Laura Lipke, Health Science Librarian, Binghamton University

Sloane Kelley, Library Director, NYITCOM at Arkansas State University

Mary Ying-Fang Wang, A.T Still University

Lori Fitterling, University Library Director, Kansas City University

Objectives:
Building on prior research, this study identifies the 100 top-cited articles in osteopathic medical education authored by faculty from U.S. Colleges of Osteopathic Medicine. The objectives were to not only shed light on the most impactful research on this topic, but also to serve as a guide for future research directions in osteopathic medical education, providing a valuable resource for researchers, educators, and policymakers in this field. Metrics were analyzed and cluster analysis was used to find thematic relationships and trends in the literature.

Methods:
Three search strategies were used to extract articles for this analysis using the Web of Science database. (1) Various search terms related to “osteopathic medicine” and “medical education” were used to retrieve relevant articles; (2) Combined search #1 with a search of authors affiliated with osteopathic medical schools to ensure that articles from experts in the field were included; and (3) A search targeting 7 journals which are known to publish osteopathic articles and are not indexed in the Web of Science database. These 7 journals were searched with the combined terms for search #1. Additional bibliometrics were gathered through Google Scholar and other methods. Inclusion and exclusion criteria for all articles were identified and applied to the final results.

Results:
This analysis had several key findings, with "Interventions to Cultivate Physician Empathy" by Kelm, Zak, et al., BMC Medical Education (2014) emerging as the top-cited article. Academic Medicine had the highest number of top-cited articles and shows the complex relationship between Impact Factor and citation counts as Academic Medicine, (IF 1.998), ranked 6th when considering citations per publication, while BMC Medical Education had the highest citation-to-publication ratio at 37.8 despite a lower Impact Factor. Cluster analysis identified 4 thematic clusters based on the total weighted degree of centrality, and key topics were identified.

Conclusions:
This research contributes to a comprehensive understanding of the scholarly landscape in osteopathic medical education. It emphasizes that impact is multifaceted, extending beyond impact factors, as other variables play a vital role. The thematic relationships and dynamic trends unveiled through cluster analysis provide a roadmap for future research. It's important to note that this field's research is distributed across diverse journals and subject areas, not confined to a single central platform. These findings offer valuable insights for researchers, educators, and policymakers, shedding light on the most impactful research and guiding future directions in osteopathic medical education while also giving weight to the notion that a COM educational journal should be explored.

The Reality of Reproducibility: Evaluating the Capacity of Database Interfaces to Support Searching for Systematic Reviews

Track(s): Information Management, Information Services

Roberto Silva, Head, Fralin Biomedical Research Institute Health Sciences and Technology Library, Virginia Tech

Cozette Comer, Evidence Synthesis Services Coordinator, Virginia Tech

Jackson Hoch, Evidence Synthesis Librarian and Library Liaison, Newman Library, Virginia Tech

Objectives:
This study evolved from the underlying query - are thoroughly documented and transparently reported search strategies, particularly in systematic reviews, truly replicable? In this study, we evaluate eight database interfaces commonly used in health sciences systematic reviews for the capacity to replicate complex search strategies within a single institution.

Methods:
Building primarily upon Gusenbauer & Haddaway’s (2020) study of academic search systems’ systematic search qualities, we have designed a single-institution case study with two phases aimed at evaluating search replicability within eight bibliographic databases including, PubMed, Academic Search Complete, and Scopus. In phase one, each databases’ user interface is examined for the presence and usability of features essential to systematic, replicable searching. Unaddressed or ambiguously answered questions will be discussed with vendor representatives to confirm accuracy of results. These descriptive findings will inform our assessment of the usability of each interface for running and documenting a complex search. In phase two, we evaluate replicability of three independent search strategies within each interface. We will test between-searcher replicability (searches executed by two independent searchers at the same time) and within-searcher replicability (searches executed by the same searcher, six months apart). Replicability will be measured by (a) yield and (b) content. Searches will be considered replicable if these measures are consistent across the search-interface pairings, respectively. Nearly replicable searches will present minor differences in yield and/or content (≤1% of original yield), while unreplicable searches will show major differences (>1%).

Results:
In this session, we will provide an overview of methods and findings. Findings from phase one will describe key characteristics of the search guidance documentation (e.g., FAQ, help guides), character limits, field codes, “smart search” or other advanced algorithms, search history and refinement, and downloadability of results. Findings from phase two will focus on replicability of yield and retrieved content for each search-interface pairing, respectively. We will also draw qualitative conclusions about the replicability of all three searches by interface. Our full protocol will be made available for those interested in replicating this study at their own institution.

Conclusions:
Conclusions will be informed by the presence of interface features required for transparent, replicable search strategy documentation (phase one) and the actual replicability of test searches (phase two). We hope this study will inspire others to test replicability of searches in commonly used interfaces at their institution. These studies could provide an evidence-base to demonstrate the challenges of search replicability and consistency to vendors. Ultimately, search replicability between institutions should be tested, considering replicability is an underlying assumption of systematic review methods.

There's Strength in Numbers: Evaluation of a Cohort Internship Program for Data Librarianship

Track(s): Education, Information Management

Peace Ossom, Associate Director, Nat'l Center for Data Services, NYU Langone Health

Erika Martinez, NYU Langone Health

Verónica Hoyo, Executive Director, NNLM Evaluation Center

Alisa Surkis, Deputy Director, Health Sciences Library, NYU Grossman School of Medicine

Objectives:
The objective of this study is to evaluate the following research questions: (1) What is the participant satisfaction upon immediate completion of the internship program? (2) What is the difference in self-reported skill level in five areas of data librarianship (data wrangling, data visualization, data analysis, coding, and data curation) based upon pre- and post-internship assessment, and (3) what impact did the internship have on the participants’ interest in the field and post-graduate school career path.

Methods:
We used pre and post surveys to assess the changes in interest and perceived knowledge/skills, and an anonymized satisfaction survey to gauge the interns’ opinions (Year 1, N=8; Year 2, N=12) about their experience, with a ten-point scale. A long-term impact survey was sent to interns approximately one year after completion of the internship. To analyze the pre/post changes, we calculated means and medians across all responses (year 1 and year 2 interns), and also looked at means for changes in knowledge/skills within internship host sites, due to the distinct nature of the projects for each site. We also used basic descriptive statistics to analyze the satisfaction data and long-term impact data.

Results:
Response rates were 87.5% and 100% each cohort for the pre-internship survey, 50% and 100% for post-internship and satisfaction surveys, and 62.5% for the long-term impact survey. Respondents scored how the program met expectations (mean was 8.8), and mean likelihood to recommend to a friend was 9.3. Median interest level in data librarianship increased from 9 to 10. Increases in mean comfort for knowledge/skills ranged from 1.25 (data analysis) to 2.56 (coding). Year 1 interns do not yet have data librarian positions but reported the internship had prepared them for their desired career.

Conclusions:
The reported positive change in abilities in every area for an eight week internship may be due to a focused,

structured schedule of lessons and practice. However, because the projects focused on different skills, a more fine-grained analysis may reveal even greater gains and areas of opportunity in the specific areas of host site focus. The overall satisfaction level was high, which may be due to the incorporation of networking opportunities along with skill-building. While none of the first year interns had a job as a data librarian yet; it may be too early to assess the impact of the program, since all agreed that it had prepared them for their desired career.

They Don't Have Time for the Library - Understanding What the Literature Says About GME Trainees and Their Needs

Track(s): Information Services, Innovation & Research Practice

Emily Johnson-Barlow, Regional Health Sciences Librarian & Associate Professor, University of Illinois Chicago

Deborah Lauseng, Associate Professor & Regional Head Librarian, University of Illinois Chicago

Emily Gilbert, Assistant Professor and Liaison Librarian, University of Illinois Chicago

Tina Griffin, Associate Professor, Information Services and Liaison Librarian, University of Illinois Chicago

Holly Hudson, Asst Prof and Regional Health Sciences Librarian, University of Illinois Chicago

Objectives:
Medical librarians are often assigned multiple levels of medical trainees for library services and support. One such group is the residents and fellows, who comprise the Graduate Medical Education (GME) in medical schools and hospitals. This population has extensive, competency-based milestone requirements from the Accreditation Council for Graduate Medical Education (ACGME) to complete their training. To understand the unique needs of this population and how the librarians can support this audience, the investigators initiated a needs assessment (NA).

Methods:
NA’s use multiple data sources to inform on a population’s needs. Our initial phase of the NA project performed a comprehensive literature review about GME education, adoption of the milestones by GME programs, and librarians' support of this audience. We reviewed a selection of ACGME milestones corresponding to GME programs currently available at our respective campuses. Once we had identified milestones where we believed library instruction or assistance might be integrated, we began our literature review. Our team split the literature search into various categories such as GME scholarly needs, GME clinical needs, and health science library need assessments. All results were uploaded into an evidence synthesis software, Covidence, for data extraction. Our extraction includes documenting the trainee program and audience, the paper’s study methods, results, and conclusion, and if there was librarian involvement.

Results:
Our initial findings uncovered many opportunities for library/librarian partnerships within the GME curriculums and administrators. From the early analysis we have found three areas of possibilities: demands around scholarly projects in GME, efficiently finding clinical evidence to apply to clinical care, and an overlap of information literacy competencies and the ACGME milestones. We will report additional results when data extraction is complete.

Conclusions:
We performed a comprehensive literature review to inform our needs assessment project and our understanding of GME populations. Findings will determine our decision-making process about library

services and capacity to address needs. We believe that librarian-designed services would help GME trainees successfully fulfill their competency milestones, placing these services firmly in GME curriculums.

This is How We Do It: Tenure & Promotion in Academic Health Sciences Libraries

Track(s): Professionalism & Leadership

Erin Reardon, Public Health Informationist, Emory University

Objectives:
The traditional three arms of the tenure-track professor’s duties are teaching, service, and research. Tenure-track academic librarians, though their numbers are dwindling, are expected to meet the same or similar requirements to achieve tenure. Whether academic librarians should have tenure or tenure-like status is a matter of some debate, but what has not been examined are the criteria by which academic librarians are evaluated in order to be awarded tenure. This study aims to examine and compare these criteria across academic health sciences libraries’ promotion and tenure documents.

Methods:
Data from the Association of Academic Health Sciences Libraries (AAHSL) descriptive statistics from Fiscal Year 2021-2022 was used for this project, specifically item D.14a, Faculty Appointment Status. Of the 130 AAHSL member institutions who responded to the survey, there were 29 institutional health sciences libraries who reported a tenure-track faculty appointment system for their staff librarians. This study will not examine the promotion requirements for libraries that reported other faculty-like appointment with tenure-like systems such as continuous appointment, non-tenure-track faculty appointment, faculty appointment outside the library, or no faculty appointment.

Through internet and institutional website searching, documentation of the tenure criteria will be obtained for as many of the 29 libraries with tenure-track faculty librarians as possible. Then, a qualitative and quantitative content analysis of this documentation will be undertaken using NVivo to examine the similarities and differences between these institutions’ requirements. This documentation will also be examined under the framework of the ACRL Standards for Faculty Status for Academic Librarians, particularly point 4: Promotion and Tenure.

Results:
n/a

Conclusions:
We hope that this examination will provide insight into the expectations for tenure and promotion for academic health sciences librarians.

Understanding Transitions of Public Librarians to Health Science Librarianship.

Track(s): Education, Professionalism & Leadership

Christopher Eaton, Health Science Librarian, University of Florida

Courtney Pyche, Public Health Liaison Librarian, University of Florida

Objectives:
Transitioning between types of libraries throughout a career is not uncommon in the library profession. Today, librarians continue to do so to adapt to the changes in the professional environment. The literature has explored the varying backgrounds and transitions made between libraries, yet gaps exist in information for those transitioning from public libraries to health science and academic health science center libraries. To investigate, two librarians are designing a survey to inform on the factors that contribute to public librarians transitioning to health libraries, the professional development they utilized, and what gaps exist in their understanding of their new specialization.

Methods:
Drawing on previous surveys of the profession, the librarians created a ten-question survey asking current health science librarians, who were previously public librarians, about their professional and educational backgrounds as well as the experiences they have had in their transitions. The survey will be open for one month and disseminated via listservs, specific professional organizations and groups selected through purposive sampling, and snowballing sampling. The study will be submitted to the Institutional Review Board for approval before launching.

Results:
Data collection will begin in January 2024, and the group hopes to obtain at least 30 responses for analysis within a month of launching. Results will be presented in tables and analyzed narratively.

Conclusions:
Understanding the professional and education backgrounds is an important factor for creating professional development curriculum and forecasting the future of the profession. This survey aims to inform the literature and the profession on the state of public librarians transitioning to health science librarianship.

Update and Expansion of an Interprofessional Education Search Strategy Validation Study for NLM PubMed, Ovid MEDLINE, and Elsevier Embase

Track(s): Information Services

Luke Barron, Clinical Librarian, University of North Carolina at Chapel Hill

Rebecca Carlson, Eshelman School of Pharmacy Librarian, University of North Carolina, Chapel Hill

Anne Sophie Nachman, Graduate Assistant UNC Health Sciences Library

Objectives:
The interprofessional education (IPE) literature continues to grow and it has become increasingly difficult for researchers to find relevant IPE papers and keep up with the latest research. This paper builds on our previous study, a relative recall validation of an IPE search hedge to find literature in NLM PubMed. Now, we have expanded the search hedge for Ovid MEDLINE, and Elsevier Embase and updated the hedge for NLM PubMed. This study aims to maximize recall of IPE articles and improve effectiveness and efficiency of searching for IPE literature across databases.

Methods:
We translated the search hedge for Ovid MEDLINE and Elsevier Embase formatting, added appropriate

subject headings and proximity operators. Additionally, the search hedge for PubMed was updated to include a version with proximity operators, since this PubMed feature was added after the development of the original search hedge. We validated each search strategy using relative recall methodology. This validation measure tests the recall of the search strategy against a gold standard set of articles; our IPE gold standard was developed in the previous paper and includes 256 MEDLINE-indexed articles from published systematic reviews.

Results:
A high-level summary of results from the original search hedge validation study, the updated results from the PubMed search hedge with proximity operators, and the new (pending) results from the validation in Ovid MEDLINE and Embase will be presented with the relative recall numbers and percentages.

Conclusions:
This study expands the availability of current, validated search hedges for IPE literature in Ovid MEDLINE, Elsevier Embase, and NLM PubMed to improve the efficiency of locating studies for reviews and other research.

Utilizing the Newest Vital Sign (NVS) to Assess Health Literacy at a Regional Academic Medical Center’s Family Medicine Clinic

Track(s): Clinical Support, Health Equity & Global Health

Kelsey Grabeel, Associate Professor, University of Tennessee Graduate School of Medicine

Eric Heidel, University ot Tennessee

Sarah Burton, US Department of Energy Office of Scientific and Technical Information

Shauntá Chamberlin, University ot Tennessee

Alexandria Wilson, Associate Professor / Senior Medical Librarian, Preston Medical Library / Health Information Center

Objectives:
The study examined the correlation between the physician’s subjective assessment of health literacy rates and actual health literacy rates amongst patients at an academic regional medical center’s family medicine clinic as determined by the Newest Vital Sign method. Our hypothesis was that there would be no agreement between the physicians’ perceived health literacy risk of their patients and their patients’ risk score.

Methods:
A sample of n=150 patients that were 18 years of age or older were verbally interviewed using the Newest Vital Sign tool before seeing their physician. After the treating physician met with the patient, the physician was asked to measure that patient’s level of health literacy on a Likert-type scale and a “yes/no” scale. Frequency and percentage statistics were performed in SPSS to describe the distributions of patient and physician responses. Between-subjects statistics were used to test the hypothesis.

Results:
Analysis of the patient surveys revealed one in four patients have a high likelihood of low health literacy. Analysis also revealed there were significant positive correlations between physician response to perception of a patient’s low health literacy risk and NVS survey responses. When using the NVS scoring criteria as the “gold standard,” physicians correctly rated n=83 patients (55.3% of the time) to have a certain level of limited

health literacy. Despite the risk of limited literacy, 97.3% of physicians perceived the patient to understand what the physician was saying.

Conclusions:
Physicians at the medical center have a one in four likelihood of seeing a patient with low health literacy at the clinic. Through this research, we found that the physicians’ ratings correlate moderately and significantly with how the patients scored on the NVS, meaning there was a significant positive correlation between the physicians’ perceptions of patients’ risk of low health literacy and actual patient NVS score, which is the opposite of our hypothesis. Librarians can provide health literacy support to physicians to improve physician/patient communication and customized patient education materials for the clinic’s patient population.

PAPER PRESENTATIONS: PROGRAM DESCRIPTION ABSTRACTS

We have sorted paper presentation program description abstracts in this section by title in alphabetical order.

An Academic Health Sciences Library's Experience Providing Training in Generative Artificial Intelligence

Track(s): Education

Fred LaPolla, Lead, Data Services, NYU Langone Health

Genevieve Milliken, Data Services Librarian, NYU Langone Health

Catherine Larson, Associate Director of Systems & Technology at the NYU Langone Health, Health Sciences Library

David DeSimone, Branch Manager, Robbins Library, NYU Langone Health

Loida Pan, Research Impact Librarian, NYU Langone Health

Nicole Contaxis, Head of Data Sharing and Metadata Management, NYU Langone Health

Caitlin Plovnick, NYU Health Sciences Library

Background:
The appearance of generative artificial intelligence tools such as Chat GPT, Google Bard and Perplexity AI has created immense interest among medical institutions, patrons, and librarians. Our academic medical center has invested heavily in generative AI technology, and accordingly is expressly interested in ways that the library can incorporate new technologies to serve our community. To meet this institutional demand, we partnered with institutional stakeholders to investigate employing generative AI, focusing on providing introductory educational training to our institution.

Description:
Taking the lead from a broader institutional push to find ways to utilize generative AI throughout our

academic medical center, the library instituted a working group to help investigate use of generative AI in the library and the research life-cycle. To assist in educating patrons at our medical center, we created a library guide of generative AI information and resources, and held workshops in partnership with institutional stakeholders. Information on the libGuide includes links to an internal, HIPAA compliant generative AI instance, Microsoft Azure, as well as details on other generative AI models. Workshops for our community have covered prompt generation, overview of generative AI, and ethics of generative AI, and have included instructors both from within the library and institutional partners. Building on past successes in data education, we are now additionally partnering with our institutions’ predictive analytics unit to provide on-going entry-level training in generative AI to a broad audience with the goal of increasing community understanding and streamlining work within the analytics unit.

Program Conclusions:
Our project is on-going. We intend to provide descriptive information on: class enrollment, resource page views, class evaluation data, and data from a pre- and post-test on AI knowledge. We also aim to assess challenges and opportunities experienced by librarians in providing education on generative AI.

Academic and Hospital Libraries: Stronger Together With Shared Resources

Track(s): Clinical Support, Education

Ruby Nugent, Biomedical Research Librarian, National Jewish Health

Catisha Benjamin, Manager of Library Services, Children's Hospital Colorado

Marie St. PIerre, librarian/Informationist, Children's Hospital Colorado

Ellie Svoboda, Education Informationist, University of Colorado Anschutz Medical Campus

Emily Petersen, Family Health Librarian, Children's Hospital Colorado

Jerry Carlson, Medical LIbrarian, UCHealth

Kristen DeSanto, Clinical Informationist, University of Colorado Anschutz Medical Campus Strauss Health Sciences Library

Liz Kellermeyer, Library Director, National Jewish Health

Melissa De Santis, Director, Strauss Health Sciences Library, Univ of Colorado Anschutz Medical Campus

Michelle Schonken, Electronic Resources Librarian, University of Colorado Anschutz Medical Campus

Yumin Jiang, Head, Collection Management, University of Colorado

Ben Harnke, Research Informationist, University of Colorado Anschutz Medical Campus Strauss Health Sciences Library

Background:
An academic health sciences library, along with two large hospital systems and a standalone hospital serve overlapping populations in a large urban area. All the institutions are separate organizations, and the hospitals have independent libraries. Employees, students, and residents regularly travel between these independent institutions. Librarians at the institutions were motivated to create opportunities to collaborate in order to provide better services and increase efficiencies while maintaining autonomy of their individual libraries. This paper will describe a few of the many solutions that have been created.

Description:
This collaboration is a unique partnership between academic and hospital libraries, enhancing educational programs, subscription purchasing, and consulting between library systems. In an environment where hospital libraries are closing and administrators can have incorrect assumptions about resource access, this collaboration has been beneficial to both the hospital librarians and the academic librarians. The medical librarians are familiar with each other’s resources and programs through their state’s medical library organization and through their shared medical staff. The hospital librarians are conferred adjoint library faculty status with the academic health sciences library, allowing them to more effectively help shared staff. These connections led to lightened workloads and shared expertise between libraries.

The shared subscription model between some of the libraries has been one of the most challenging and impactful collaborations. The model has changed over time and required flexibility. Most subscriptions are evaluated annually between the institutions to determine usage and cost effectiveness, which helps keep the bottom line cost in check.

Formal feedback on collaborative efforts was not done, but the librarians talk about and review endeavors informally over time. This paper presentation was a call to evaluate and provide after-the -fact feedback.

Program Conclusions:
The collaborative efforts between the academic health sciences institution and hospitals has been highly beneficial, creating a network of collaboration and resource sharing, as well as opportunities for librarian professional growth. The librarians’ cooperation, fostered by their familiarity with each other’s resources and shared medical staff, has led to successful joint initiatives, educational programs, and subscription management. While formal evaluation mechanisms were not initially established, ongoing informal discussions and proactive feedback during program planning have been instrumental in refining and enhancing partnerships. Working closely together, each institution brings its unique perspectives and experiences to the larger group through regular communication, training, and learning opportunities. These joint efforts creat

Adaptive Space: A Student-Centric Approach to a Health Sciences Library Wellness Room

Track(s): Information Services

Varina Kosovich, Student Support Coordinator, UNM Health Sciences Library & Informatics Center

Background:
Prior to establishing this project, there was no dedicated space for wellness activities in the library, nor on the health sciences campus. The objective for this project was to create a space that students could use for wellness needs including yoga, stretching, having a quiet place to unwind, prayer, etc. A secondary objective was to assess interest for a wellness space through student focus groups and ultimately feedback forms placed in the room.

Description:
Health sciences students experience mental health issues, demoralization, and burnout at greater rates compared to other students and often do not report these issues for fear of marginalization. Additionally, many neurodiverse students struggle in higher education due to environments that are not accessible. Considering these information points, authors surveyed space in the library to determine an appropriate area to create and pilot the Wellness Room. Funding was established through an internal grant and a study room was approved for use in the pilot. Resources were provided with autonomous space, feminist ethics of care, and trauma informed care tenets. Feedback from student focus groups guided decisions about supplies that

were purchased and furniture selections. Authors also conducted a literature review to explore what other institutions had implemented and to examine strategies that would benefit health sciences and neurodiverse students. It was determined through student feedback gathered from the pilot that the room was not only needed, but appreciated. Authors quickly realized students had outgrown the pilot space and expanded the Wellness Room in order to offer additional supplies and space for growth.

Program Conclusions:
While the nature of this space requires consistent adjustment, in terms of the grant funding and pilot, the authors are confident in the success of the project. Student feedback received through various channels prompted the institution to prioritize the space and establish regular funding opportunities. Due to the student centric and student led nature of this project, authors continue to monitor feedback and fulfill requests for wellness tools. Additions to the room are under consideration, including a small collection of wellness books. The Wellness Room has proven to be a cost effective, popular, and beneficial library service.

All About Systematic Reviews: Making Quality Learning Equitable and Freely Accessible to All

Track(s): Education

Poppy Krump, Information Scientist, Vanderbilt University Medical Center

Lori Harding, Information Scientist, Vanderbilt University Medical Center

Nunzia Giuse, Vice President for Knowledge Management; Professor of Biomedical Informatics; Director, Center for Knowledge Management, Vanderbilt University Medical Center

Taneya Koonce, Deputy Director, Vanderbilt University Medical Center

Annette Williams, Senior Information Scientist, Vanderbilt University Medical Center

Jerry Zhao, Vanderbilt University Medical Center

Sheila Kusnoor, Senior Information Scientist and Associate Director for Research, Center for Knowledge Management; Research Assistant Professor, Department of Biomedical Informatics, Vanderbilt University Medical Center

Background:
In recent years, there has been a growing awareness of the need to promote knowledge sharing through open access initiatives to help address social inequities. Following this trend, our team adapted a systematic review training series created for internal use into a format to allow for freely available, online asynchronous instruction. To help reach all information professionals and other potential systematic review authors, we chose to disseminate the content through a platform that the team had previously developed as a tool to guide decisions about publishing. The overall goal was to expand the role of information professionals in supporting evidence-based medicine by helping them develop the knowledge and skills needed to advise and lead systematic review efforts at their organization.

Description:
The self-paced systematic review instruction tool was created by adapting a virtual training series developed and administered to a team of academic information scientists through eight, hour-long sessions in Spring 2023. Instructors included individuals experienced in designing, conducting, and publishing systematic reviews. The sessions were adapted into a video series format, with a target length of 10 minutes or less per video. The intended audience included individuals with no prior knowledge and those seeking in-depth details on systematic review processes. In total, 18 videos were created addressing a variety of topics, such

as the planning needed for the development and implementation of a systematic review; the types of literature reviews to consider (e.g., critical, scoping, umbrella, realist); an explanation detailing all steps needed to conduct the review process; a comparison of currently existing systematic review management tools (e.g., Rayyan, ASReview, Covidence); details on how systematic review protocols can be registered on platforms such as PROSPERO and OSFRegistries; and instruments for quality assessment of the primary literature (e.g., AMSTAR, ROBIS). To allow learners to check their understanding, quiz questions addressing key learning objectives were created. The questions were formatted to offer feedback on responses and allow for unlimited retakes.

Program Conclusions:
The training initiative aims to expand access to instruction on how to conduct a systematic review. Outcomes that will be assessed include the number of visitors to the training website, the number of views each video receives, and completion of quiz questions. A feedback questionnaire will also be made available through the training website to better understand learner satisfaction with the training materials, perceived usefulness, and characteristics of the course participants. Questions will be collated and answers made available through a FAQ. Through the provision of freely available instructional content, this project aims to ensure equitable access of quality learning to all information professionals, as they engage in the effort of supporting best practices through evidence-based medicine at their organization.

Applying Machine Learning to Prioritize Literature Search Results: A 5-year Review at an Academic Medical Library

Track(s): Innovation & Research Practice

Michelle Cawley, AUL for Health Sciences, University of North Carolina, Chapel Hill

Background:
Since 2018, library staff at an academic medical library have partnered with researchers to apply unsupervised and supervised machine learning (ML) to systematic reviews, comprehensive evidence syntheses, and bibliometric analyses. This application of ML saves considerable time and resources for research teams by reducing the volume of articles that must be screened manually. It also provides an opportunity for researchers to expand the scope of their questions without reviewing thousands of additional articles. To date librarians have partnered on over 30 projects with researchers from health sciences schools and the medical center using these technologies. This session will describe our ML service, including project summaries and outcomes, evidence for efficacy, tips for building capacity, lessons learned, and next steps.

Description:
A department chair with ML expertise established the service using pre-existing tools. Staff developed AI literacy through internal training and had opportunities to practice applying tools to projects. As staff gained experience, they began engaging with research teams on projects that included a significant number of search results (five thousand or more) to inform researchers about ML techniques, implement ML processes, consult on projects, and co-author publications. Onboarding for new staff includes AI literacy training and opportunities to shadow experienced staff before they lead projects.

To evaluate the service, library staff track projects including project type and description, project partners, ML techniques used, outcomes, and timeline. Individual projects are evaluated by comparing precision expected at random to precision achieved using ML tools. For supervised ML, graphs with the distribution of relevant results by probability score are used to show that studies receiving a higher probability score from ML are more likely to be identified as relevant during manual review. Lastly, we regularly run simulations testing the approach on projects where all results were screened manually to demonstrate efficacy and calculate time saved. Recall is used to evaluate efficacy with the goal of achieving 95% or greater recall.

Program Conclusions:
This service is ongoing, and we are building awareness at our institution through webinars and other outreach. To date, we have applied ML on 19 systematic and scoping reviews, 4 evidence syntheses, 3 bibliometric analyses, and 6 other projects across multiple research domains resulting in 8 publications or presentations with at least 9 more expected. We are currently developing custom versions of these ML tools that will be hosted locally. Planned next steps include developing case studies for public health curriculum, sharing code that can be hosted at other institutions, and training staff at other academic libraries. As this part of the program is developed, we will also measure the number of students and library staff trained in these methods.

Being Stronger by Being Present: The Role of Liaison Librarians

Track(s): Information Services

Mina Ghajar, Education & Research Librarian, Rutgers University

Background:
Rutgers School of Health Professions offers more than 35 degrees at graduate and undergraduate levels. More than 1300 students are enrolled in various programs with 150 faculty teaching. Programs offered by the School are physically located in three locations across NJ and have a global classroom present with students participating from all over the world. The Library Office Hours Program aims to create visibility and promote library services.

Description:
The Office Hours program has offered various services: webinar-based instruction series, in-person and online student consultations, subject-specific LibGuides, librarians embedded in online classes, and faculty/student research and systematic review assistance. This presentation will review the services offered, resulting in increased visibility for the library.

Program Conclusions:
This program is ongoing. We expect to measure the program's success by the increased requests we receive from faculty and students. We use the data compiled from the SpringShare products, RefAnalytics, LibCal, and LibInsight, to measure the program's success.

Building a Research Data Services Program: Lessons in Community and Identity

Track(s): Information Services

Carolyn Dennison, Librarian, University of Hawaii at Manoa

Jonathan Young, Librarian, University of Hawaii at Manoa

Background:
Data management is an increasingly fundamental skill for students and researchers in the biomedical

sciences, especially as National Institutes of Health (NIH) and other agencies now require researchers to include data management and sharing plans in their funding applications. Approximately 22% of the grants awarded to a university’s medical, nursing, and public health programs were from the NIH, yet there was little support for data services at a geographically isolated R1 university. Anticipating that these programs and others will need to address data management and sharing plans in their applications, the library looked for a way to rapidly enhance its capacity to support this area of need.

Description:
With grant funds, the library aimed to establish a data management and sharing instruction service by conducting a community assessment, creating an asynchronous online course, and hosting a data management and sharing event to promote the library as a resource for data management and sharing needs.

The initial assessment confirmed that there was a need in the health sciences of the university for organized data services support, and that there was particular interest in cultural sensitivity when asked about topics related to research data being collected and used in our geographic region. Based on the assessment, a self-paced, online, asynchronous open curriculum was developed to cover four topics: 1) research data concepts; 2) working with research data; 3) data sharing and planning; 4) data in the region, particularly data related to indigenous populations. The final topic integrated with a library hosted symposium on the same topic, where university researchers shared experiences and concerns within the topics of data sharing, community consent, and data sovereignty. This interdisciplinary event brought together participants from health sciences, basic sciences, economics, and engineering and generated a conversation about data sharing, positioning the library as a locus of expertise.

Program Conclusions:
The feedback from the symposium was universally positive, and the library is in the planning stages to continue the event in future years. Lessons learned from the symposium, especially on community data sharing and indigenous data sovereignty, continue to inform the library’s staff on data projects, such as our engagement with the NIH All of Us dataset, and to improve the library’s responses to the concerns of researchers and the community. Following the success of this project as a foundation, library and campus administration approved the creation of a dedicated data services position in the library, which will build upon the open curriculum and develop further research data services to support the university’s needs.

Building A Research Ethics Protocol Data Archive at the Kamuzu University of Health Sciences: Challenges and Opportunities

Track(s): Information Management

Patrick Mapulanga, Scholarly Communications Librarian, Kamuzu University of Health Sciences

Background:
The creation of a research protocols data repository for Kamuzu University of Health Sciences' Research and Ethics Committee IRB involves vital objectives to ensure effectiveness and ethical adherence. These include establishing a centralized repository for easy access, enforcing security measures, adhering to regulations, and designing a user-friendly interface. Other objectives involve version control, metadata standards, advanced search capabilities, collaboration tools, integration with existing systems, training resources, and long-term data preservation. The repository will also feature reporting tools, streamlined ethics review workflows, researcher feedback mechanisms, and scalability to accommodate growth. These objectives collectively aim to enhance research management while maintaining ethical standards.

Description:
Developing, implementing, and evaluating a research protocols repository for Kamuzu University's Research and Ethics Committee IRB involves key steps:

1. Identify needs and data types.
2. Set objectives and create a project plan.
3. Design repository's architecture and user interface.
4. Ensure security and compliance.
5. Develop software with features like submission, version control, and search tools.
6. Test thoroughly and address issues.
7. Launch to users, provide training and support.
8. Migrate existing data accurately.
9. Monitor usage, gather feedback, and assess objectives.
10. Improve based on feedback, enhancing user experience.
11. Ensure long-term data preservation and sustainability.
12. Utilize reporting for insights and efficiency assessment.
13. Monitor scalability and make adjustments.
14. Align submission and review with ethical workflows.
15. Offer ongoing user training and support. These steps collectively enable the IRB to establish an effective repository supporting ethical research, collaboration, and streamlined reviews.

Program Conclusions:
Developing a research protocols data repository for Kamuzu University of Health Sciences' IRB offers numerous benefits. The repository would centralize research protocols, enhancing efficiency, accessibility, and transparency. It enforces standardized formats, aids version control, and ensures data security. Monitoring, training, and streamlined communication improve the review process. Data analytics identify trends, aiding IRB operations. The repository fosters collaboration, reduces paper usage, ensures compliance, scales with growth, and facilitates a feedback loop for improved research. Yet, successful implementation demands careful planning, resources, and support. Regular updates, user training, and technical issue resolution are vital for maximizing the repository's positive impact.

Building a Stronger Systematic Review Service One Intervention at a Time

Track(s): Information Services

Becca Billings, Systematic Reviews Coordinator, UAB Libraries

Emma O'Hagan, Clinical Services Librarian, University of Alabama at Birmingham

Jill Deaver, Head of Clinical, Academic, and Research Engagement, University of Alabama at Birmingham

Background:
Strong workflows are of growing interest for librarians wanting to ensure their Systematic Review Services (SRS) operate efficiently. In 2022, a reference unit at an academic Health Sciences Library conducted a needs assessment to determine what their librarians needed for an efficient, manageable, and sustainable Systematic Review Service. The results of the needs assessment determined implementation of the following: Define role of Systematic Review Coordinator, implement accessible tracking system to the entire SR team, establish weekly SR status meetings, hire a new SR team member, and assign specific tasks to staff members. The purpose of this project is to report the results of these interventions and to detail next steps in the overall needs assessment to improve our SR service.

Description:
A follow-up survey was conducted of librarians who work on systematic reviews to assess the five workflow changes implemented after the 2022 needs assessment. Surveys will be distributed to the seven members of the systematic review team to determine the success of the current workflow, and if there are any areas that still require changes. Once survey results have been submitted, responses will be categorized under the appropriate workflow implementation, then analyzed for both positive and negative input. Further changes will be implemented if any responses are negative.

Program Conclusions:
Based on the results of the follow-up survey, data will be gathered regarding the five major workflow changes including: the role of Systematic Review Coordinator, implementation of a tracking system, weekly SR status meetings, the hire of a new SR team member, and specific tasks assigned to staff members, the systematic review workflow will either require further improvement or be left alone.

Building the Capacity of Health Librarians for Data Management services in Africa: A National Library of Medicine and DSI-Africa Initiatives

Track(s): Information Management

Grace Ajuwon, College of Medicine, University of Ibadan

Oluwaseun Abiodun-Asanre, Systems Librarian, Lagos State University College of Medicine

Voilet Ikolo, Delta State University, Abraka, Delta State Nigeria

Background:
Health librarians are expected to update their job knowledge and skills to enable them to compete effectively in our changing society. They need knowledge and skills acquisition in emerging areas. However, lack of funds is a major hindrance to the acquisition of new skills and competencies by health librarians in Africa.

The overall objective of the NLM and DSI-Africa sponsored project (training workshops) was to develop and enhance the knowledge and skills of African health sciences librarians to provide research support to DS-I Africa consortium members, students, faculty, scientists, and health care providers in academic and health institutions in Africa. In addition, the paper highlights programs sponsored by these organizations and their impact on the health library space in Africa.

Description:
The NLM in partnership with Data Science for Health Discovery and Innovation in Africa (DSI- Africa) sponsored two training workshops for African health librarians during the DSI-Africa Consortium meeting held 1-3 Nov 2022, Cape Town, South Africa, and at the 17th Biennial Congress of the Association for Health Information and Libraries in Africa, 16-20 October 2023 in Gaborone, Botswana.

A total of 35 health librarians attended the Cape Town workshop physically while 29 others joined virtually. The participants were from 15 African countries. The contents of the workshop included data science, research data management, evidence synthesis, and systematic reviews facilitated by the Network of Africa Medical Librarians (NAML).

Also sponsored, was a two-da pre-conference workshop on data science facilitated by librarians from North Western University, Illinois, USA and attended by 50 participants from 16 African countries. The contents of the workshop included data, data visualization, data repository, metadata, and Redcap.

A pre and post-training evaluation was carried out to determine the immediate outcome of the training. It assessed the change in knowledge levels of participants before and after the workshop. The participants were mostly females, and a quarter were above 45 years of age. Most of the participants (92%) showed knowledge improvement at the end of the workshop.

Program Conclusions:
The paper concludes that the performance index of African health librarians through training will enable them to acquire the skills and competencies needed to render appropriate and quality data management services for quality health information delivery in Africa. Training will enhance the capacity of African health librarians to provide adequate data services support to researchers and students in their institutions and also provide systematic review support services to members of their communities.

Collaboration in Cost-Sharing: Establishing Good Foundations for Long-Term Partnerships

Track(s): Information Management, Information Services

Zemirah Ngow, Collection Strategist, UC San Diego Library

Laura Schwartz, Program Director, Content Acquisition & Resource Sharing, UC San Diego Library

Background:
For years, the University Library had slowly developed several unique cost-sharing opportunities with our School of Medicine and Medical Center, in an ad-hoc format without any regular procedure, or documentation indicating roles and responsibilities of each respective party. New Librarian positions were back-filled in areas primarily affecting subject specialty and outreach, finance, collection development and management, and content acquisition resource sharing. With fresh eyes, we began evaluating our current structures, or lack thereof, regarding cost-sharing, ultimately leading to many discussions spawning a myriad of questions, opportunities to challenge pre-existing structures and procedures, and explore new opportunities to collaborate between the University Library and external partners in academic programs and health systems.

Description:
University, research libraries are central places of information for a variety of many users: clinicians in our teaching and learning hospitals, faculty and students in our academic programs, and researchers working in expanding knowledge and scholarship in their respective fields in life and health sciences.

Post-pandemic, many vendors began reconfiguring pricing models based on factors such as staffed bed counts, or numbers of physical locations of our hospitals and/or clinics in addition to the usual survey of FTE for certain students and faculty who our vendors consider primary users of their products. Many times, these new pricing models quickly began to price the Library out of resources and subscriptions deemed essential for maintaining accreditation for our academic programs and health systems.

The Library began to formalize procedures for contact-chains between Collection Strategists; Subject Liaisons; Finance; Library, Academic, and Hospital Administration. Licensing agreements and memorandum of understanding documentation became essential when entering into negotiations for multi-year contracts.

Program Conclusions:
Strategizing cost-sharing in a consortial environment, cost-sharing between the Library and outside programs (both academic and health system), and developing a standard language and expectation in

transparent negotiations will help shift our collective bargaining power thus enabling us to maintain our standard of excellence in information resources.

The Library will continue to monitor new program starts, increases in new student FTE, and how the mergers or additions of new hospital acquisitions will affect our ability to quickly pivot to negotiation-ready stances as the Library moves into balancing budget shortfalls and the increasing costs of accreditation essential resources institution-wide.

Creating a Graphic Medicine Book Club to Promote Interprofessional Education and Community Building

Track(s): Education

Michelle Knecht, Senior Medical Librarian, Schmidt College of Medicine, Florida Atlantic University

Tiffany Follin, Medical Liaison & Outreach Librarian, Florida Atlantic University

Background:
The purpose of this program was to develop an interprofessional healthcare book club that would promote interprofessional education and a sense of community among students, faculty, and staff involved in the health sciences at a state university. “Interprofessional education occurs when students from two or more professions learn about, from, and with each other to enable effective collaboration and improve health outcomes” (World Health Organization, 2010). Interprofessional education is a key curricular component of many health science fields including nursing, medicine, pharmacy, dentistry, and public health. The book club was marketed to students, staff, and faculty in these areas through a series of Instagram videos promoting the graphic medicine collection and targeted emails promoting the book club.

Description:
Each semester the book club reads one graphic medicine novel on a healthcare topic from the patients’ perspective to produce a robust discussion among the participants. "Graphic Medicine is a field that explores and supports the interaction between the medium of comics and the discourse of healthcare" (Graphicmedicine.org, n.d.) Graphic medicine novels were chosen to stimulate interest in the book club and the library’s graphic medicine collection. Additionally, due to the limited free time of the book club participants, graphic medicine novels were selected to create a low barrier for participation as the novels selected could be read in a short amount of time.

The first book club meeting will be held in person and virtually on November 29th, 2023. Snacks and drinks will be provided by the library for in-person participants, and the book club members will discuss Depression Part 1 and Depression Part 2 from Allie Brosh’s graphic novel Hyperbole and a Half: Unfortunate Situations, Flawed Coping Mechanisms, Mayhem, and Other Things That Happened. The book club will meet again in February 2024 to discuss a graphic medicine novel tied to Cancer Awareness Month.

Program Conclusions:
This program was created to address the required Interprofessional Education Collaborative core competencies (Interprofessional Education Collaborative, n.d.) within the field of health sciences. The author and her colleagues will survey the book club participants at the end of the first book club meeting to capture the demographics of the attendees as well as interest in and future direction of future meetings. Results from these surveys will be included in the presentation.

World Health Organization. (2010). Framework for action on interprofessional education & collaborative practice. http://whqlibdoc.who.int/hq/2010/WHO_HRH_HPN_10.3_eng.pdf.

Graphic Medicine International Collective. (n.d.). About graphic medicine. https://www.graphicmedicine.org/about/

Interprofessional Education Collaborative. (n.d.). IPEC core competencies.

Creating a Publishing and Impact Service

Track(s): Education, Information Services

Terry Selfe, University of Florida

Background:
Many health science center libraries support Clinical and Translational Science Award (CTSA)-funded institutes which provide resources and training opportunities to advance clinical and translational science. Dissemination of research findings is necessary for promoting impact and an expectation of funding agencies. However, a scan of Clinical and Translational Science Awardees websites revealed only a limited number offer services supporting this portion of the research lifecycle.Our purpose was to develop a publishing and impact service to help maximize the dissemination and impact of the research conducted by our CTSI-affiliated investigators.

Description:
Our library-based publishing and impact service supports the dissemination and impact of the research conducted by CTSI-affiliated investigators by promoting and supporting the creation of high-quality manuscripts publishable in peer-reviewed journals; thereby broadening the reach and increasing the impact of this research. Services include: training pre- and postdoctoral trainees and early career researchers in creating manuscripts following good reporting practices; providing consultations regarding journal selection and manuscript submission; facilitating peer writing groups; providing training and consulting services regarding research metrics; advising authors on NIH public access compliance issues; and providing consultations and training regarding research impact. Training workshops are offered biweekly. The Lunch and Learn series of one-hour workshops covers a variety of topics of interest to the CTSI audience, such as choosing a journal in which to publish, navigating the manuscript submission process, and complying with the NIH public access policy. The Good Reporting Practices series consists of one-hour workshops designed to introduce the use of reporting standards to ensure complete reporting of study findings. Finally, the 20 Minutes to Impact series comprises 20-30 minute sessions focused on providing step-by-step instructions attendees can actively follow to acc

Program Conclusions:
While it is still a developing program, early indicators suggest the new publishing and impact service is being well received. Immediately after presenting the idea to the CTSI’s Translational Workforce Development Directorate we were asked to present two workshops for the upcoming 2023-24 seminar series for early stage investigators. Additionally, the CTSI recently promoted our new service in their monthly newsletter and linked to the web page we created for additional information. [Note: we will update the abstract with relevant stats such as web page visits, number of consultation requests, and workshop stats]

Creating Research Culture in a Physician Assistant (PA) Program

Track(s): Education

Katie Hoskins, Health Sciences Librarian, Assistant Professor, Touro University Nevada

Megan DeArmond, Jay Sexter Library, Touro University

Oksana Matvienko, Professor, Research Associate, Touro University Nevada

Sherli Koshy-Chenthittayil, Data Analyst, Touro University Nevada

Julie Horwath, Systems Librarian, Assistant Professor, Touro University Nevada

Background:
The Physician Assistant Program requires its students to complete an Integrative Master’s Project. This project is equivalent to a master’s thesis and is completed by student groups. The initial phase includes activities such as forming groups, choosing a topic, completing CITI (IRB) training, and learning how to search databases and gather resources. In the past four years, faculty members from the PA program, the Office of Research, and the Library have worked closely to develop and implement library training that PA students complete within the first three months of the program. The main focus of the collaboration was on the content of the training to ensure that it meets the needs of PA students who traditionally enter the program with very limited, if any, research training.

Description:
The training consists of two parts. The first part entails each student’s completion of an online interactive training module. The second part is a group meeting with a librarian (in person or virtually). Individual group meetings offer targeted instruction, brainstorming on individual topics, and assistance with formulating a research question. Overall, the library training has a dual impact: it helps students develop skills to locate appropriate information and compels them to get an early start so that they have some basic knowledge of their topic before their first meeting with a PA faculty member assigned to supervise their IMP. Multiple groups representing four PA cohorts with research topics ranging from a clinical focus (wilderness medicine, ultrasound, opioids and pain management) to education (virtual medical education and educational apps) and a community focus (access and quality of care for vulnerable and underserved populations) have completed the library training in the past four years. After completion of the training, students are asked to complete a 4-question survey administered via Qualtrics. The survey is designed to gather feedback about students’ experience with the online databases as well as elicit comments for improvement. The average response rate for the surveys was 63% from 2020-2022.

Program Conclusions:
The results of the student survey are used to make improvements to the library training to support the next cohort of students. As a result of student feedback from four different cohorts, changes have been made. For example, we shifted focus to library resources that aligned with their topics rather than taking time for instruction related to citation management. Further results and analysis will be shared during the presentation. The library training portion has evolved with the development and growth of the IMP and has helped the transition to a more established research culture. The training is also a model for effective collaboration between the library and an academic program that provides customized education to meet the student and program’s needs.

Cultivating Values of Diversity, Equity, and Inclusion (DEI) Through a Book Club in Health Sciences Education

Track(s): Professionalism & Leadership

Hannah Schilperoort, Head, Wilson Dental Library, University of Southern California

Kim Austin, Assistant Dean of Diversity, Inclusion & Access, Herman Ostrow School of Dentistry of USC, University of Southern California

Background:
A librarian and health sciences faculty and staff created a book club with a focus on diverse authors and themes of equity and inclusion. The purpose of the book club is to increase community engagement, interpersonal communication, mindfulness, and cultural humility through reading and discussion. This presentation will summarize the development, implementation, and assessment of the book club and provide resources and guidance for starting and sustaining a book club. This program will appeal to health sciences librarians interested in innovative partnerships with liaison groups that cultivate values of diversity, equity, and inclusion (DEI) in health sciences education. The program aligns with MLA goals of fostering DEI within the association and health sciences librarianship.

Description:
The organizers consulted resources on book clubs in academic libraries and literature on the value of reading for empathy and cultural awareness. The book club was promoted to students, faculty, and staff via email listservs, newsletters, social media, and in-person communication. A research guide was created as a central hub with information about the books and authors, meeting dates and times, purpose of the book club, registration and book suggestion forms, and community agreements and guidelines. Book club titles are made available via ebook access and a display of print copies in the library. One book is read and discussed over three online meetings each semester. Members suggest and vote on upcoming books. Assessment of the book club is done through informal polls and discussion during book club meetings and anonymous feedback surveys at the end of each semester. Participants provide feedback on the strengths and weaknesses of the book club structure and discussion format with a particular focus on sense of belonging and psychological safety. Organizers meet regularly to review feedback and plan changes to maximize participant engagement and inclusion.

Program Conclusions:
The presentation will include outcomes from Fall 2022 through Spring 2024. Forty-five faculty, staff, and students have registered with 10-15 attending each meeting. Participants prefer online discussions with occasional opportunities for in-person events. Participants value agency in selecting books, inclusive community guidelines, and discussions that are informal yet structured to provide space for everyone to speak. Participants express an increased sense of belonging, self-reflection, cultural awareness, and a desire to apply new understandings to healthcare contexts. Organizers use participant feedback to increase active participation and engagement. Outcomes indicate that book clubs can be effective in cultivating values of DEI and increasing a sense of belonging and cultural humility in a health sciences educational context.

Data Core: a Library Service for the Analysis of Sensitive Datasets

Track(s): Innovation & Research Practice

Sarah Ben Maamar, Associate Director for Research Services, Weill Cornell Medicine

Alice Chin, Data Core Manager, Weill Cornell Medicine

Terrie Wheeler, Library Director, Weill Cornell Medicine

Background:
Our academic institution confronts persistent challenges in the management and analysis of large, sensitive research datasets. Researchers at our institution urgently require secure environments for the analysis of extensive datasets containing sensitive information, including patient health records and external data sets from external entities such as the Center for Medicaid and Medicare Services (CMS). In response to this demand, our library partnered with the infrastructure and cloud engineering teams to establish a secure computational data enclave named "Data Core," which serves as the sole institutionally approved enclave to host sensitive or restricted data. The library staffs and administers the Data Core.

Description:
Data Core offers computational power within a controlled environment, as well as scientific software and scalable memory, to cater to research projects with substantial computational demands. Its primary focus was to serve Population Health Science researchers dealing with massive healthcare datasets. Today, Data Core supports nearly 100 projects across various departments, accommodating individual users, research teams, and entire classrooms. Researchers are charged based on their usage, covering expenses related to staffing, software, and servers, ensuring sustained support for the service. In the past year, Data Core library data custodians led the coordinated effort to achieve certification from CMS for both its on-premises and cloud environments, enabling our institution to offer the most cost-effective and appropriate computational environments.

The library team administers the computational environments, ensuring that technical settings align with data governance agreements for each project and dataset. They also ensure that current Data Use Agreements (DUA) are approved by our Institutional Review Board (IRB). A member of the library team additionally serves as the Data Custodian and the Data Core team act as intermediaries between data providers and users. The Data Core program is evaluated through user feedback and response time to user requests.

Program Conclusions:
In summary, Data Core at our institution stands as a pivotal resource, offering a secure and efficient platform for the analysis of large and sensitive datasets. Under the library administration, user feedback has been positive and is reflected in the increase of the number of projects and users using the Data Core service over the years. Data Core is a growing service and its administration exemplifies a successful partnership between librarians and infrastructure personnel.

Developing a Team-Based Approach to Medical School Liaison Services

Track(s): Clinical Support, Professionalism & Leadership

Ryn Gagen, Medical School Librarian, University of Minnesota

Lisa McGuire, Associate Director for Education & Research Services, Health Sciences Library, University of Minnesota

Background:
Our library has used a liaison model for providing librarian expertise & connection to our academic health center’s colleges and schools. We are an R1 institution with one liaison assigned to each college/school. This liaison model provides autonomy and choice in working styles and prioritization but leaves gaps in sustainability and workload inequities. We developed a new team-based approach to liaison work with our Medical School that utilizes three liaison librarians who manage workloads in instruction, consultation, research support and evidence syntheses work. The purpose of this program is to create sustainable service models, balance workload equity, and collaborate to create a consistent service experience.

Description:
Our library experienced significant staff turnover and a recent reorganization that allowed us to hire three new liaisons for the Medical School. We approached this as a cohort hire and onboarding experience. Our new hires were all in place by late spring 2023 and we began onboarding and training as a cohort in all aspects of librarian work. By using this model we were able to allow the liaisons to collaborate to create new workflows, documentation, and work sharing, all of which continue to be adjusted as needed. In addition to these tools, the liaisons have structured meetings every week, to make sure all work is covered and no one is overwhelmed. Each liaison has different work and education experiences, and the cohort model has allowed the liaisons to learn from each other as they are getting comfortable in their positions. One of the major aspects of the liaison’s work will be co-authoring systematic reviews, and the cohort model allows the liaisons to complete systematic review training together, discuss challenges and compare search strategies, and shadow each other as they begin working on systematic reviews.

Program Conclusions:
We expect to begin evaluation of the new liaison cohort model beginning in late Spring 2024, one year after we launched the new cohort model. Because the implementation of the medical school liaison cohort model primarily involves changes to internal staff workflows, our evaluation goals will be focused on metrics that center the experience of liaison librarians. These metrics include individual medical school liaison job satisfaction, collaboration and cross-training between liaisons, and projected workload sustainability. We will also discuss how we plan to ensure this team-based model is cohesive with our Libraries' evaluation and rewards structure for continuous appointment.

Doing It Together: Integrating A Clinical Education Librarian Into A Continuing Professional Development (CPD) Team

Track(s): Clinical Support

Lindsey Gillespie, Medical Librarian, Dignity Health

Michelle Lieggi, Clinical Education Librarian, Dignity Health

Background:
Many librarians provide support for continuing education (AKA continuing professional development, CPD), yet are not fully integrated into a CPD department. When our library became part of our division’s CPD program, the medical librarian discovered a strong need for librarian support related to the planning and development of CPD activities. When the division expanded, demand for library support grew, but the medical librarian was unable to support the increased number of requests related to CPD. With support from leadership, the Director and the medical librarian got approval for an additional librarian, the clinical education librarian (CEL). This new position reports to the Director of CPD and is fully integrated into the CPD department.

Description:
The CEL was hired in 2022 and began working directly with the medical librarian and the CPD team. The CEL provides literature support for the CPD team and faculty, and participates in the planning, development and execution of CPD activities. The CEL conducts searches related to the topics of educational activities and for references related to cultural/linguistic competency and implicit bias as required for accreditation. The role also fully participates in the CPD planning process, providing expert guidance on the need to incorporate evidence-based literature. Beginning in 2023, the CEL partnered with the medical librarian to begin synthesizing data on the topics of all librarian-led literature searches requested by clinicians to determine if any could be developed into CPD activities. In evaluating this data, the medical librarian and CEL can determine if their practices in conducting literature searches can lead to CPD activities that address the practice gaps and needs for education of its clinicians division-wide.

Program Conclusions:
Integrating a clinical education librarian into a CPD department can be a means of promoting the use of best-evidence and determining continuing education needs. Data analysis has shown some correlation between librarian-led literature searches and needs for continuing education, but more longitudinal data is needed to truly identify practice gaps. The CEL and medical librarian are working together to collect more robust data on all librarian-led searches, as well to conduct proactive literature searches to determine emerging topics where continuing education may be warranted. With a more proactive approach, it is hoped that more practice gaps can be identified and developed into education that supports clinician and organizational needs.

Embedded Librarianship in Pilot Online MPH Program

Track(s): Education, Information Services

Kristen Sheridan, Head of Education and Information Services, Boston University

Background:
This program focuses on the successful partnership between a health sciences librarian who liaises with the school of Public Health and the educational design team as they build a fully online MPH from the ground up in early 2021. The goal was to embed library services into this new program with recorded tutorials on various library resources, provide access to course materials, and offer continued research and access support for students. The library is situated on an urban medical campus that supports students, faculty, and staff in the health sciences fields.

Description:
The health sciences librarian was invited to collaborate with the education and instructional design team on providing access to articles, eBooks and other library resources and materials for the first cohort of the newly created online MPH. They implemented tools like Leganto to embed course materials directly into Blackboard, as well as find OER materials that students around the world could access and keep costs at a minimum. This program has just started with its third cohort of students, so it has been integral to get feedback from students on their wants and needs for the program, as it differs from the in-person MPH that is already well established on campus. One of these suggestions was more opportunities for programming, and the librarian been able to provide social support for students in the program by hosting library drop-in programs and well as live demonstrations and tutorials on databases and citation management tools. Supporting students in this way has been crucial, as it gives them the opportunity to connect with their peers in a virtual setting.

Program Conclusions:
This program is ongoing and evolving, and the librarian has been able to offer continued support to students

and staff to assist with research, finding resources, accessing materials, and facilitating instruction on library resources. Some long-term goals are to continue integrating new technologies into the program and provide instruction where applicable, as well as measure continued engagement with students by finding new and exciting ways to encourage learning, offer research support for coursework and provide a community through programming and outreach as the program grows.

Enhancing Library Services for the Virtual Medical Library Community Through Creative Event Planning

Track(s): Education, Information Services

Katelyn Angell, Medical Librarian/Associate Professor, CUNY School of Medicine

Background:
1.To teach key medical research skills to faculty, students, and staff

2.To celebrate and promote the scholarly accomplishments of medical faculty and students by inviting them to give monthly lectures to the community

3.To expand the professional development opportunities of the community by referring them to free virtual conferences, workshops, lectures, and more happening outside of the medical school

Description:
The CUNY School of Medicine Medical Librarian is a fully virtual library, housed within the CUNY School of Medicine and staffed by two full-time librarians. It can be difficult to recruit users and promote services at any library, particularly if it exists in cyberspace. As such, the medical librarians decided to create a three-tiered virtual events service to help faculty, students, and staff learn about available resources and collections, receive more assistance in conducting medical research, promote their own research and scholarship, and gain access to a wealth of free professional development opportunities. We secured the support of the associate dean, and created a page on our website dedicated to events. The initiative will be rolled out over the course of the 2023-2024 academic year, with the MediScholar series kicking off in October with a workshop on MESH headings. Students and faculty are currently being invited to give lectures to the community on past and present research projects, and we’ve created a repository for free, virtual professional development opportunities. The latter will be updated on a monthly basis. The services will be evaluated by post-event assessment polls, as well as a feedback page on the events website.

Program Conclusions:
The first outcome we expect to measure is event attendance. We would like more evidence that our services are being utilized by the community, as we primarily only have database usage statistics. We’ll be able to include these numbers in reporting for our next LCME accreditation review.

Second, we want to poll attendees at our monthly workshops to ensure that the structure, format, and content were useful to their development as medical researchers. Their feedback will allow us to improve our content delivery. They’ll also be able to suggest workshop topics, ensuring that their voices will be heard when developing outreach and programs that best serve their needs.

The Evolution of an Electronic Lab Notebook Community

Track(s): Information Management, Innovation & Research Practice

Elizabeth Whipple, Assistant Director for Research and Translational Sciences, Ruth Lilly Medical Library, Indiana University School of Medicine

Levi Dolan, Data Services Librarian, Ruth Lilly Medical Library, Indiana University School of Medicine

Background:
Electronic Lab Notebook (ELN) products are intended to replace physical lab notebooks in basic science and clinical research labs. As part of supporting rigor and reproducibility in biomedical research practices, our library supports ELN implementation at our institution. Our institution has provided an ELN option for five years and the library has led the project since inception, including developing and teaching workshops, offering consultations, re-negotiating the license for the institution, and serving as our institution's ELN site administrators. Our role gave us access to backend usage data which we analyzed using a Python script we wrote. Using this analysis, we were able to identify ELN super users.

Description:
In late 2022-early 2023, we conducted a series of informational interviews with our identified electronic lab notebook (ELN) group of super users. Based on feedback from these interviews, we organized and sponsored an in-person networking event in the Fall of 2023 to allow super users to meet each other, compare techniques and pain points in ELN workflows, and provide feedback to us on desired enhancements and avenues of support. Based on feedback from the networking event and follow up consultations with individual researchers, in late 2023 we created a shared ELN with reproducible digital templates and widgets available to all our affiliates. We also organized an advanced ELN training session led by ELN product vendor staff to be held in early 2024. This sequence of library outreach and programming activities has increased the library’s understanding of our ELN community and diversified our methods for advancing best practices in data management.

Program Conclusions:
We expect to measure the number of requests for creation of reproducible ELN templates and widgets, number of attendees for our advanced LabArchives straining session, and the number of users who access our institution-wide ELN. From the time we started these outreach activities up until the Spring of 2024, we expect to be able to report monthly statistics on the increase in number of ELNs created, terrabytes of data storage, number of new users, and the number of monthly user logins.

Forming a Campus Wide Systematic Review Working Group

Track(s): Education, Information Services

Carrie Adams, Program Director, University of Florida

Lauren Adkins, Assistant University Librarian, University of Florida

Background:
A group of health sciences librarians decided to form a working group where we would be able to learn more about systematic reviews and other evidence synthesis literature reviews. Individually, we all had experience and some familiarity with working on these types of reviews both with faculty members as co-authors but also in the role of educators with graduate health professional students looking to learn the basics. The purpose of this working group was to share our experiences and learn from each other. Then it was

suggested that we expand the scope of the group and ask academic librarians at our institution if they would also like to participate. A number of our non-health sciences librarians colleagues expressed enthusiasm about joining.

Description:
With the ever-increasing demand for complex evidence synthesis literature reviews especially systematic reviews, scoping reviews, and meta-analyses in the health science colleges, it became apparent there was an opportunity to share our knowledge and practices with one another. Since these review types are becoming more popular outside the health sciences discipline, our non-health sciences librarians colleagues colleagues were excited to participate. The group is comprised of eleven health science librarians and eight non-health sciences librarians from a variety of disciplines including: education, science, history, and journalism. The goal of this working group was to be a safe space for everyone of all skill levels to discuss the current research and the evolving practices of conducting and leading systematic reviews. Another goal of this group is educating other colleges and departments of the best practices when conducting these reviews. Group members are encouraged to ask questions, discuss challenges, share helpful resources, brainstorm educational outreach ideas for patrons, and anything else they would like.

Program Conclusions:
The enthusiasm of this group was evident from our first meeting. One member shared that this was the first Zoom meeting they had attended where everyone was engaged in the discussion. Since the first meeting, there has been in increase in collaboration among the members where we ask for advice and helping resolve challenges that arise. Future plans for this group include creating a campus wide outreach program to share best practices for conducting complex evidence synthesis reviews and continuous information sharing. In addition to this much needed outreach we would like to work together to create campus wide standard operating procedures for handling these review projects. Members have also discussed investigating future research opportunities in systematic reviews.

A Gamified Design Process for an Academic Health Sciences Library Remodel

Track(s): Professionalism & Leadership

Janet Crum, Director, Health Sciences Library, University of Arizona Libraries

Altaf Engineer, Associate Professor of Architecture, School of Architecture, University of Arizona

Annabelle Nuñez, Interim Director, University of Arizona

Bo Yang, Associate Dean for Research, School of Landscape Architecture and Planning, University of Arizona

Sandra Bernal, Lecturer, University of Arizona, College of Architecture, Planning, and Landscape Architecture

Background:
This program will describe an innovative partnership between an academic health sciences library and the university’s College of Architecture to develop plans for a library renovation, including a gamified approach to obtaining input from students and campus stakeholders. After students indicated they found the Health Sciences Library facility dated and not aligned with their needs, the library contracted with the College to develop renovation plans with the following objectives: 1) Develop a vision for the facility to be shared with stakeholders and used to solicit funding; 2) Create a compelling package of architectural plans and visuals

with three scenarios with increasing scopes and costs; and 3) Center student needs and feedback throughout the project.

Description:
Library staff and architecture team gathered information about current trends in library design and summaries of recent student feedback. Then we hosted two design charettes, using a gamified process to engage participants and encourage detailed and specific feedback. In the first charrette, students played a Monopoly-like boardgame. Students rolled dice (to randomize prompts), and when they landed on a space, they were asked to respond to one of three questions related to the topic associated with that space. Responses and preferences were collected and analyzed to inform the second charrette, in which stakeholders played a solitaire game to sort student input into a matrix (under stakeholder control or not, a project priority or not). The architects then synthesized feedback from the charettes and other sources to develop preliminary sketches, which were presented to key library stakeholders in mid-fall 2023. Final plans will be delivered by the end of December 2023, after which they will be presented to library and campus stakeholders and development officers to secure funding to move forward with the remodel. Plans will detail three levels of renovation: improvements possible with existing funds; improvements requiring moderate additional one-time funds; and a full remodel requiring significant external fundraising.

Program Conclusions:
The project has already netted two major successes: 1) engagement with health sciences students, who have responded positively to our approach and indicated they feel heard; and 2) the close collaboration between library staff and the team of architects and architecture students who understand the university environment and the student experience in ways that an external team might not. Project deliverables will be evaluated in part by the level of external engagement and enthusiasm generated by the designs. As work progresses, we will seek additional student feedback as needed to inform decisions (e.g., traffic flow, furniture purchases) and evaluate work performed as stages are completed.

Going Further Together: Collaboratively Building a Brand New Research Impact Challenge

Track(s): Education, Innovation & Research Practice

Jenessa McElfresh, Interim Associate Director, University of Tennessee Health Science Center

Jess Newman Mcdonald, Research Data & Scholarly Communications Lead, University of Tennessee Health Science Center, Health Sciences Library

Megg Doolin, Lead Digital Projects Analyst, The University of Tennessee Health Science Center

Sarah Newell, Research and Learning Services Librarian, University of Tennessee Health Science Center

Annabelle Holt, Research & Learning Services Librarian, University of Tennessee Health Science Center, Health Sciences Library

Background:
Inspired by the University of Michigan’s Research Impact Challenge, UTHSC faculty librarians created a unique five-day Challenge tailored to a health science research audience. Nine librarians and one institutional research colleague leant their diverse expertise to designing content based on common researcher requests and available subscription-based and open-source resources. The Challenge was aimed at early career researchers and faculty preparing for promotion and tenure, who were most interested

in establishing themselves professionally and showcasing their scholarly accomplishments. The objective of the Challenge was to help researchers better understand and manage their online scholarly presence and the impact and reach of their research. The Challenge also served as an opportunity for internal collaboration and a unique means of m

Description:
Our team held both small and full group meetings to assign, discuss, share, and review our plan for marketing, creating, and displaying content for the Research Impact Challenge. In developing the challenge, we collaborated with our marketing team to prepare a communications plan which included social media, email communication with colleges, branded Challenge email templates, and advertisement within the library. Participants received Daily Challenge emails that included a written overview of that day’s activities, a video introduction, and a link to the Challenge LibGuide. A team of librarians created a custom LibGuide to serve as the primary platform to host all instructional material for the Challenge. The LibGuide included a daily overview of each challenge and tasks to be completed by participants through written instructions, interactive how-to guides, handouts, and external resources to further support learning and engagement. Daily themes included: Claim or Enhance Your Online Presence, Understand and Locate Your Research Impact Metrics, Reach a Wider Audience with Open Access Publishing, Learn How and Why to Share Your Research Data, and Stay Informed and Build Your Network. Program evaluation included a post-Challenge survey and registrants received a Certificate of Completion as a result of completing the survey.

Program Conclusions:
Assessment of the Challenge included both concurrent and post-Challenge measures. “Read statistics” were collected for Daily Challenge emails, LibGuide views were monitored in the days leading up to and through the Challenge week, and a post-Challenge survey was distributed to registrants. Additionally, reference emails and consultations regarding Challenge topics were collected throughout and immediately following the event. Overall, the Challenge received 64 registrations and 20 survey responses. Impact Metrics, Scholarly Profiles, and Data Sharing were the most highly rated days, although all days received above average ratings. 79% of survey respondents indicated that they were highly likely (7 or above out of 10) to recommend the challenge to a colleague.

Health Misinformation Resilience Programming in Public Libraries: Community Outreach by Academic Librarians

Track(s): Education

Kelsey Cowles, University of Pittsburgh

Rachel Suppok, University of Pittsburgh

Rebekah Miller, Research & Instruction Librarian, University of Pittsburgh

Background:
As research and instruction librarians, we regularly present programming on health misinformation to our campus academic and clinical audiences. A consultation with local public library colleagues revealed a need for health literacy-related programming in public libraries; health misinformation was identified as an area of particular concern and public interest. Beginning in spring 2023, we partnered with public libraries to offer a selection of health misinformation programs adapted for their audiences. We have now presented both an interactive lecture style program and an escape room session several times each and in various public library branches in neighborhoods within our city and surrounding areas. Our goal is to share strategies and skills for identifying and responding to health misinformation, especially online.

Description:
We used existing misinformation presentations originally developed for an academic and clinical audience as the basis for our lecture-based session. However, we significantly modified the material to make it appropriate, relevant, and engaging for a general audience. For instance, instead of covering misinformation related to scientific literature, we increased the content on recognizing common tactics used to promote questionable health information in various types of media. We also selected misinformation examples that were unlikely to be perceived as political as our goal is to reach all community members regardless of their personal beliefs.

For the escape room program, we utilized an existing misinformation-focused escape room game created by the University of Washington Center for an Informed Public. After testing, we made minor changes to the puzzles and gameplay to better suit our needs.

Community outreach is essential in addressing the proliferation of health misinformation. We have found that these two program styles each have advantages and disadvantages related to ease of implementation, audience engagement, depth of content, and opportunities to assess participant learning. This reflection will provide inspiration and guidance to other academic librarians looking to engage their broader communities around the issue of health misinformation.

Program Conclusions:
We have offered both programs once to date and will be offering each several more times through the end of 2023 and in the first half of 2024. We will present informal qualitative data on participant reactions to the programs, instructor impressions, the mechanics of running each type of session, the pros and cons of each type of session, and recommendations for academic librarians looking to explore similar partnerships in their areas.

The Health Sciences Library's Role in Embedding Family Resource Centers in Federally Qualified Health Centers

Track(s): Health Equity & Global Health

Rebecca Birr, Director Health Sciences Library & Family Resource Centers, Valleywise Health

April Aguiñaga, Library Information Support Coordinator, Valleywise Health

Background:
This paper describes the implementation and evolution of a family resource center strategy by the Health Sciences Library (HSL) team in response to the health systems’ need to address the social determinants of health. The organization is the metro area’s only public teaching health care system. It is governed by a publicly elected board of directors focused on exceptional care. As the safety net health system for the county, the organization provides services to predominantly underserved, low-income and ethnically diverse populations. The organization includes the medical center, the regional burn center, three behavioral health centers, and 11 Federally Qualified Health Centers (FQHCs) located throughout the county. The eight Family Resource Centers (FRCs) are embedded in the Federally Qualified Health Centers (FQHCs).

Description:
With a focus on health disparities and patient-centered care, the organization is dedicated to addressing the social determinants of health (SDOH) for families. The organization recognizes the obstacles families encounter and that they need support to achieve healthy lives. Families face issues and challenges well

beyond attending doctor visits. Economic, educational, and environmental needs also weigh heavily on families. Helping to address these stressors can be a catalyst to better health outcomes. As such, the organization has embraced family support programs as a method to alleviate SDOH needs by embedding FRCs in its FQHCs. The organization’s family support strategy began in 2003 with a planning grant, with the first FRC opening in 2007. To date, there are now FRCs in eight FQHCs. The implementation and continued operations of these FRCs are managed by the HSL team. FRC services includes classes, resource distribution, information assistance, and referrals. In addition to the HSL staff, the FRCs have fourteen full time coordinators that are bilingual in English and Spanish and have a bachelor’s or master’s degree in social work, health education, family studies, education, or a related field.

Program Conclusions:
The FRCs are an integral part of the care team and help providers address a family’s SDOH. The FRC work has ingrained the HSL staff into the clinical environment and made them valuable assets to the system. Funding comes from a variety of sources including support from our foundation through private gifts and grants, state grants, and HRSA funding. Data related to key outcomes including classes, referrals, material distribution, surveys, and fundraising are collected to tell the story and outcomes of FRC work in the health system.

“Hello World!”: Helping Medical Librarians Up-Skill with Python Basics

Track(s): Education

Justin de la Cruz, Associate Director, NNLM NCDS, NYU Langone Health

Genevieve Milliken, Data Services Librarian, NYU Langone Health

Background:
Computation and computational methods are ubiquitous in academic research. As a result, it is important that information professionals in Data Services be comfortable, and literate, with programming and computational modes of analysis. Having an understanding of how to program will enable librarians to read, write and understand human and machine-generated code and better be able to meet the needs of students and researchers. Most importantly, the knowledge and skills gained learning how to program will also enable information professionals to be intelligent partners and contributors in the research life cycle, research data management, and open science.

Description:
This 9-week class introduces medical librarians to computer programming in Python and is designed to provide information professionals with the entry level knowledge needed to understand how programming works and to enable them to start writing programs of their own. The class content is based around using Python in Jupyter Notebooks hosted in Google Colaboratory, which are self-contained virtual environments that provide access to computing assets online (similar to Google Docs but for coding). Class modules contain instructional videos that lead students step-by-step through the contents of each lesson, as well as weekly readings and interactive discussions. Students begin with the fundamentals of programming (data types, control structures, containers) and progress into data analysis and visualization. The class, which will be offered annually, is free and offers a unique blend of asynchronous learning (through videos, readings, assignments, and discussion boards) with synchronous mentorship from library workers who are experienced in Python. The project is currently underway and will be assessed by student feedback, surveys, and analysis of final project presentations. Mentor and instructor feedback will also be used for course assessment. All course analysis will be used for continuing improvement of the curriculum.

Program Conclusions:
The course is in-progress and student evaluations and feedback are being collected for analysis once it is finished. It is anticipated that this course will be shown to help increase programming literacy among participants. While specifically geared for Medical Librarians, this course follows prevailing trends in librarianship in which practical skills such as learning to program are becoming more relevant, as seen with the popularity of the Carpentries and other types of continuing education.

High School Push-In Program to Encourage Pursuit of a Career in Library Science

Track(s): Education, Innovation & Research Practice

Diana Delgado, Associate Director, Information, Education and Clinical Services, Weill Cornell Medicine

Terrie Wheeler, Library Director, Weill Cornell Medicine

Andy Hickner, Education and Outreach Librarian, Weill Cornell Medicine

Michael Wood, Head, Resource Management, Weill Cornell Medicine

Sarah Jewell, Assistant Director, Clinical Services, Weill Cornell Medicine

Background:
The literature indicates a plethora of programs aimed at increasing the number of underrepresented minorities in librarianship, however the vast majority of the profession is still white (https://www.ala.org/aboutala/offices/diversity/diversitycounts/divcounts). Interventions have been made with undergraduate students and graduate students of other disciplines, but few with high school or middle school programs. The career occupational literature indicates that individuals gravitate towards career choices at a young age. Our aim in this study is to assess whether learning about the field of librarianship would make underrepresented high school students more likely to consider it as a career.

Description:
[Blinded name of organization] introduces underrepresented in medicine (URiM) high school students to health professions through outreach and classroom teaching. Our Library has partnered with this organization to add medical librarianship to its portfolio of professions taught to students. We will present five modules on information and health literacy basics. Students will participate in focus groups to help design the curriculum to their interests for superior engagement. Pre and post surveys will be administered to gauge student learning and engagement. An Advisory Board of leading librarians from various underrepresented backgrounds will provide feedback on each project deliverable, as well as tell their story to the students of how they became a librarian. The curriculum will be administered in spring 2024, then student focus groups will identify what worked and what did not, and the curriculum will be modified to incorporate student recommendations. This curriculum will be administered again in fall 2024 and evaluated in spring 2025. The evaluation component will consider how well student input and feedback is integrated into the curriculum, as well as Advisory Committee feedback. Student pre and post surveys in the second year will evaluate student engagement and learning. The grant will culminate in 2025 with a publicly available final report and an online toolkit.

Program Conclusions:
Introducing high school students to the profession of librarianship with an emphasis on health sciences librarianship may increase their interest in pursuing a career as a librarian. Further grant funding may enable this high school education component to become part of a pathway program for underrepresented students to explore a career as a librarian. Successful outcomes of this grant will align with MLA’s strategic goals to

“Build a Better Future”, “Ensure Diversity is a Thread that is Reflected throughout MLA Education”, and “Build on the Diversity and Inclusion of MLA Communities”.

If You Don’t Count It, Did It Happen?: Centralizing and Standardizing Library Statistics

Track(s): Innovation & Research Practice, Professionalism & Leadership

Alisa Surkis, Deputy Director, Health Sciences Library, NYU Grossman School of Medicine

Background:
Organizations like the Association of Research Libraries (ARL) and the Association of Academic Health Sciences Librarians (AAHSL) request annual statistics on library services such as online resources, education, and consultations. Additionally, statistics on usage and impact are used to demonstrate the value of the library to institutional leadership, to provide information for stakeholder reports, and to make decisions on how to more effectively employ resources. These statistics come from a variety of sources, (e.g., Google Analytics, manual counts of physical space usage, self-report and a ticketing system for library consultations) and vary in their ease of collection and reliability. Our aim was to centralize and standardize this data to simplify reporting and facilitate the development of dashboards.

Description:
The first step in centralizing and standardizing library data was to inventory the data collected. We identified and evaluated roughly 200 existing data elements and assessed whether additional data elements were needed for existing or aspirational use cases. We arrived at roughly 100 data elements that library leadership agreed provided value and that we were able to measure. Next, we designed an instrument to capture library consultation and teaching data, for which the data collection process varied widely across the library. This instrument, built in REDCap, was iteratively developed using feedback from everyone who would be using the forms. We worked with the team that managed LibAnswers, the library ticketing system, to determine how data on consultations that was collected through LibAnswers could be harmonized and pooled with consultation data collected with the REDCap forms. Data collected on a quarterly basis on physical space usage was also captured through a REDCap form. Data elements from systems (e.g., Iliad, Google analytics) were downloaded from those systems as csv files. The final step in updating the data collection process was the development of a data pipeline which could process the raw data from all systems to create a centralized, standardized dataset.

Program Conclusions:
There are several desired outcomes of this process. One is efficiency in reporting, so that requests for AAHSL or ARL stats, or requests for data from institutional leadership can be satisfied more quickly and accurately. A second is facilitating the development of a dashboard for real-time analysis of library statistics for internal decision making. A third is better measures of long-term library impact by using more complete data about consults to track publications and grants that have benefitted from library support. These outcomes will take months or, in the case of the long-term impact, years to come to fruition, and achievement of them will rely on developing reliable data pipelines, and engaging in an ongoing process of checking data quality.

Implementing Mental Health First Aid (MHFA)

Track(s): Education, Professionalism & Leadership

Mary Margaret Thomas, Clinical Education Librarian, University of Iowa

Background:
Mental Health First Aid (MHFA) training can help libraries educate staff, destigmatize mental illness, and provide de-escalation techniques to better meet their community’s needs. MHFA is the gold standard among mental health trainings because it's the most comprehensive training, requires a certified instructor, and eight hours minimum of training. This talk will provide a detailed description of the process for how MHFA training was implemented in the Library System so that others may borrow this model as well as highlight the benefits of the training.

Description:
MHFA training was successfully implemented in the the Library System in the summer of 2023. This presentation will cover the entire process of how the Library System successfully implemented voluntary MHFA training for all library staff; it was offered to all library workers, public facing as well as technical services staff. It will discuss the collaboration involved and how the author worked with the the Library System Human Resources Director, the the Library System's Interim Director of DEAI, and partnered with an outside certified MHFA instructor. Elements such as the initial interest survey, Getting Help LibGuides creation, marketing and promotion, and impacts of the program will be included. Procurement of funding for the training will be explained since one of the biggest barriers to providing MHFA training can be the price tag.

Program Conclusions:
Our first MHFA training was extremely well received. Library Staff appreciated that MHFA training helps raise awareness about mental health challenges, reduces stigma, and equips individuals with the skills to support others who may be experiencing mental health challenges. Additionally, MHFA training provides a safe space for discussions about mental health and enhances the overall mental health literacy and well-being of the Library System.

Implementing Redcap Research Data Collection Services at Your Library

Track(s): Education, Information Services

David King, Web & Technology Librarian, University of Louisville Kornhauser Health Sciences Library

Dani LaPreze, Assistant Professor, University of Louisville, Kornhauser Health Sciences Library

Background:
Research Electronic Data Capture, or REDCap, is a web-based platform designed to capture data for research purposes through the use and distribution of survey-like instruments. Researchers can easily create projects to collect and store sensitive data in secure, project-specific databases.

Clinical librarians develop and maintain relationships with researchers and contribute to the research process at all levels. These contributions include research planning, finding and evaluating resources, manuscript support, and everything in between. Though REDCap is intuitive to use, there are service and educational aspects to it that the library is in a perfect position to provide.

Description:
During conversations with the current REDCap administrator and institutional researchers, a clinical librarian and a technology librarian learned of vast disparities in the current service model of the management of the

institutions REDCap instances. The current model consisted of a single administrator who managed user accounts and controlled the information technology side of the program. There was a clear lack of education and service in the current model as it was being run.

The goals set forth by the team of librarians were to provide researchers with more robust training, implement an enhanced and more informative onboarding experience, and create a centralized hub that would meet the needs of the institutions research community from start of project to completion. Additionally, this would provide back-end access to librarians so that they could collect their own data on usage and satisfaction rates. This data would be used to continuously improve the user experience and training models.

The initial phase of the project focused on the overall enhancement of the current service model. This included implementation of REDCap training sessions for library personnel, enhancing the user onboarding and training experience for researchers, and collecting relevant data to be used to improve user experiences.

Program Conclusions:
Key performance indicators for implementing this service include (1) satisfaction with REDCap training, (2) interest in new REDCap instances, and (3) overall satisfaction with REDCap. The librarian team has developed new surveys in REDCap that are being used to request new accounts, onboarding or training, a process for requesting help, as well as implementation of a new, more consistent onboarding process. Over the course of the next year (Fall 2023-Fall 2024), data will be collected and analyzed, and adjustments to the program will be made as necessary.

Integrating AI Large Language Models into PubMed Searching for a Medical Student Grand Rounds Course

Track(s): Education

Sheila Green, Research & Scholarly Communications Librarian, Texas A&M University Medical Sciences Library

Steve Maxwell, Associate Professor, Texas A&M University

Catherine Pepper, Senior Associate Professor, Texas A&M University

Background:
Librarians teach PubMed searching via lecture and graded exercise in a Medical Student Grand Rounds (MSGR) course at {redacted} School of Medicine. In this semester-long course, students work with mentors to discover and present about translational basic sciences research on a clinical topic at Grand Rounds Day. ChatGPT and other Large Language Models (LLMs) are intriguing options to streamline the search process but require discernment for proper use. The teaching team set out to incorporate guided exposure to LLM opportunities and challenges into the existing search training and exercise process.

Description:
Students in MSGR must use PubMed to search the biomedical literature to inform a semester-end presentation about translational research with potential to impact clinical care. Librarians have taught PubMed search skills in the course for several years, including the use of MeSH terms and subheadings, filters, and keywords. These skills are reinforced with a graded exercise taking students through the steps to gradually refine a search. Recognizing that students would likely experiment with ChatGPT to save time on their searches, librarians worked with course directors to revise the lecture and exercise to emphasize the process of searching, to highlight where artificial intelligence (AI) is already used in PubMed, and to suggest

both uses and caveats of current LLMs to generate usable PubMed searches. The manual searches in the exercise were condensed to accommodate the additional generative AI search instructions. Students were then asked to use the LLM of their choice to generate search terms for the same topic, critique the LLM response, and share their experience with peers in a course discussion board. Grading and feedback centered on good-faith efforts to perform the search process and to evaluate thoughtfully the LLM tool.

Program Conclusions:
The recent emergence of publicly available tools for generative AI based on LLM presents new challenges for instruction, particularly when students are required to submit “original” work in assignments. The PubMed searching instruction and exercise have traditionally garnered strong positive feedback in prior student course evaluations. This paper will present student feedback on the inclusion of generative AI tools in the exercise and will describe differences between students’ work with and without the use of generative AI for their PubMed assignment. Librarians and course leadership will review the Spring 2024 course evaluations as well as the next developments in generative AI to continually improve the relevance of the course content. We expect the approach to evolve regularly.

Integration into MyChart: A Pipe Dream?

Track(s): Clinical Support, Health Equity & Global Health

Elizabeth Frakes, Associate Librarian for Clinical Services, Eccles Health Sciences Library - University of Utah

Shawn Steidinger, Associate Librarian for Clinical Services, University of Utah

Background:
Clinical librarians at a large academic medical center were tasked with reimagining a consumer health service line after the permanent closing of the patient library in the hospital. An initial proposal and presentation were given to the donor family in June in order to get buy-in and financial support to proceed with an online patient library presence. This presentation proposed an online prescription information program that allows for a more equitable delivery of information throughout the service area. The donor family were enthusiastic about the proposal but required a pilot to be conducted before moving forward. Members of the administrative suite suggested the cardiovascular department as a possible partner for this pilot initiative.

Description:
The clinical librarians currently have a service line embedded in the health system’s electronic health record (Epic) catering to information needs of clinicians. The initial vision was to expand this service in Epic to allow clinicians to request consumer level information for their patients. After meeting with the cardiovascular department leaders in August, they expressed a desire for this mechanism to be available in the patient portal, MyChart, to satisfy consumer health information needs. They indicated that a clinician-initiated mechanism in Epic would not be feasible with current demands on clinician time. Additionally, it would allow patients to take control of their health plan and make information requests on their own. Most importantly, patients are able to request information wherever they are located and are not required to make an appointment with their clinician. Recently, support was provided through the Senior Vice President of Health Sciences to make the creation of this mechanism in MyChart a top priority for Health IT. A six month information prescription pilot project is planned that will randomize a select portion of the patients of the cardiovascular department to either have or not have the ability to request health information from the librarians via MyChart.

Program Conclusions:
Data collected will include the number of clinicians who refer patients to the service, as well as the number

of patients who utilize the service. Also collected will be anonymized user satisfaction data from both clinicians and patients. This data will help to inform the clinicians and librarians about the effectiveness and clarity of the provided information in order to offer a better patient experience. The aim is to use this data to scale up the patient information prescription service to encompass more departments within the health system as well as justify expanding the staffing of this service. We will also describe the facilitators and barriers encountered while working with Health IT as this service is implemented into MyChart.

Integration of an Information Literacy Component into a Research Skills Training Program for Nurse Leaders: Development, Implementation, and Evaluation

Track(s): Education, Innovation & Research Practice

Aida Marissa Smith, Clinical Informationist, Ascension

Cyndi LaFond, Senior Director of Nursing Research, Ascension

Vallire Hooper, Senior Nurse Scientist, Ascension

Background:
Research skill development within the nursing and allied health workforce positively impacts high-quality patient care.1,2 However, barriers exist hindering nurse research. These barriers include, leadership support, gaps in education, weak resource infrastructures, and intimidation with the research process.1–5

The leadership team of a healthcare system’s national nursing research affinity group, including a medical librarian, addressed these barriers by offering research skills training to its members. The training was based on collaboratively developed learning objectives that included a substantial information literacy component. The training was conducted remotely, since the group’s 16 members were located across the country.

Description:
The research skills training included three one hour learning sessions addressing the phases of research, how to develop a research question, and navigating the medical literature. The last session, navigating the medical literature, incorporated content from the previous sessions and the voice of a nurse research scientist. The sessions were incorporated into the group’s virtual meetings, once a month for three months.

Research training participants were asked to participate in a pre and post survey, to ascertain the impact of the research skills training program. The survey measured whether the training enhanced the self-efficacy of the participants’ research skills. The rationale for this evaluation was to assist the implementation team with identifying any needed revisions or additional education. The survey incorporated questions from the “Individual” subscale of the Research Capacity in Context Tool,6 a standardized instrument. Three questions on the subscale addressed information literacy.

The survey data, captured via RedCap, will be analyzed using descriptive statistics, including average, median, and interquartile range. Differences in groups will be compared using ANOVA, t-tests, and Kruskal-Wallis for continuous variables, and Chi square and Fisher’s exact test for categorical variables. The results will be shared with participants.

Program Conclusions:
This nursing research skill training program offers an example. It shows how medical librarians can collaboratively work towards integrating information literacy instruction into professional development

opportunities for nurses. The pre and post survey results will be shared at the annual meeting and used by the implementation to team identify needed revisions or additional education.

It's Logical: Using a Logic Model to Plan and Evaluate a Campus-Wide Initiatives for Open Science

Track(s): Innovation & Research Practice

Sally Gore, Manager, Research & Scholarly Communications Services, Lamar Soutter Library, UMass Chan Medical School

Background:
In 2022, the White House Office of Science and Technology Policy (OSTP) updated its U.S. policy guidance to require that all results of taxpayer-supported research be made immediately available to the American public at no cost. This policy, known as the Nelson Memo, will significantly affect the majority of research conducted at UMass Chan Medical School, as well as acquisitions and subscriptions for the library. The Office of Sponsored Programs, Grants & Contracts Administration, looks to the library (Research & Scholarly Communication Services) to prepare investigators and staff for the implementation of these policies and mandates, including education and support for compliance. The goal of this program is to develop a successful campus-wide campaign that will both build upon existing services, as well as introduce the new policy.

Description:
A logic model is a visual representation of a theory of change. They are useful tools for thinking about, structuring, and visually communicating how a program will get results. Logic models are often used in grant proposals, but they are equally effective in program planning and evaluation at all levels. To plan and evaluate our campus-wide efforts to introduce, educate, train, and support the open science initiatives of the United States government affecting our large biomedical and clinical research community, the Research and Scholarly Communication Services Department is developing a logic model. Additionally, these open science initiatives will likely affect traditional journal publishing, prompting changes in subscription models, e.g., read and publish, transformative agreements. A successful campaign will allow more informed decision-making regarding these agreements and/or purchasing options. The model systematically looks at inputs/resources, activities, short-term outputs (1 year) that measure accountability, long-term outputs (1-4 years) that measure value to participants, and impact (3-5+ years) that measure the value of the programs and services to the university and the larger global community. It will provide us a roadmap to track our work, measure our success, identify our gaps, and plan future programming.

Program Conclusions:
We will provide the completed logic model and current status of our campaign during the program. These will include relationships and working groups established, outreach activities underway and/or completed, and first year outputs on education, consultation, outreach, and institutional support. We will also present the current status of our work addressing changes to scholarly publishing and our decisions regarding read and publish agreements.

Learning on the Job

Track(s): Professionalism & Leadership

Katie Pierce Farrier, Data Science Strategist, Network of the National Library of Medicine, Region 3

Sandra Desjardins, R & I Librarian, Texas Medical Center

Brandon Kennedy, Health Information Strategist, University of North Texas Health Science

Caraline Annichiarico, Academic Health Sciences Librarian, UAMS Library

Background:
A group of librarians noticed a lack of content specific to the challenges and concerns often encountered by early career librarians. In an effort to address this gap, the group partnered with a regional institution and hosted a three-part webinar series on job searching, setting professional goals, and navigating promotion and tenure. Group members will share their experiences about this process, what skills they learned, and how these experiences impacted their professional growth.

Description:
Members recruited expert speakers to address topics relevant to library students and early career librarians. Members volunteered to participate in soliciting speakers, developing templates, creating learning objectives, marketing the events, and hosting the webinar series. Members rotated duties to maximize the number of people involved. Overall, hosting the webinar series provided integral experience for LIS students and early career librarians. The experience also positively impacted the volunteers’ professional growth as they provided engaging content for their target audiences.

Program Conclusions:
Members gained experience with project management, utilized team collaboration skills, expanded professional networks, and developed marketing and communication skills. The three-part webinar series was offered the first half of 2023. Survey feedback and demographics were gathered from participants at the end of each webinar. So far, 122 people have attended, of which 97% of survey respondents thought the webinar would help advance their career, and 100% agreed or strongly agreed the webinars were engaging.

Lifelong Learning a Month at a Time: The Development and Ongoing Assessment of an Internal Professional Development Program

Track(s): Professionalism & Leadership

Anna Getselman, Associate Dean, Augustus C. Long Health Science Library

Rebecca Kennison, Digital Content Editor, Augustus C. Long Health Sciences Library, Columbia University Irving Medical Center

Background:
In June 2022 the health sciences library at a large urban academic medical center launched a continuing education program based on four categories of core competencies that had been internally identified: understanding user needs, effective communication, information and data literacy, and organizational orientation and strategic alignment. While previous efforts had been mostly informal and largely individual, this more formalized program emphasized group learning of new concepts and focused on developing interprofessional leadership and communication skills, with the stated goal to connect and learn from each other, thereby increasing overall understanding of the library and contributing to unit cohesiveness. After 15 months the program was evaluated, revised in response to staff feedback, and relaunched in October 2023.

Description:
From June 2022 through September 2023 staff met for an hour via Zoom on the second Monday of every month. Participation was required of all professional staff and optional for clerical staff. The program alternated between a journal club session led by two staff members and a formal presentation led by one staff member. Journal articles and presentation topics were chosen independently by each presenter. Journal articles were provided one week ahead of the meeting to enable everyone to read and prepare for discussion. The sessions in August and September 2023 were devoted to evaluation of the program. While the program was generally deemed a success by most staff, many expressed somewhat contradictory desires for both more flexibility and more direction. The monthly time commitment was also felt to be burdensome. A small working group was tasked with revising the program to address this feedback. A new bimonthly program was launched in October 2023, with the aim of introducing flexibility while still encouraging development of our core competencies. Two staff members lead each session. Format and content are determined by the pair in coordination with each other. Core competencies and learning objectives are clearly stated.

Program Conclusions:
The outcomes from the first year of the continuing education program — both positive and less positive — were summarized through the formal assessment held in August and September 2023. The revised program, launched in October 2023, will be evaluated ahead of this presentation; that assessment will be presented at the meeting. In particular, the outcomes from the first 6 months of the revised program will be compared and contrasted with that of the first year’s program, with special emphasis on how well the revised program has been received by staff during its first few months. Both the competencies development process and the structure of the program can be used as a model for internal professional development in academic health sciences libraries.

The Medical Library as a Connector to Campus Well-Being

Track(s): Professionalism & Leadership

Karin Saric, Librarian, Norris Medical Library

Annie Thompson, Head, Norris Medical Library, University of Southern California

Andrea Harrow, Clinical Librarian, University of Southern California

Background:
Following the recent intense experience with COVID-19, students, faculty, and clinicians are facing challenges with their well-being. Libraries have an impact on academic outcomes and support overall student success, and evidence shows that integrating wellness initiatives, the arts, and providing support for the whole student, researcher, and clinician, into the library setting can lead to a greater sense of belonging and well-being. Consideration of the whole person or whole student has become more important than ever and the library can be an integral part of creating “well” virtual and physical spaces as well as support other campus initiatives.

Description:
Well-being has been identified as a unifying value of our campus community leading to an increase in wellness activities across campus. However, library programming remains distinct in that it is open to all faculty, staff, and students. This paper will discuss library wellness programming and events and plans to continue increasing campus well-being. Since 2017, our programming has centered around mind, body, and soul. We have partnered with various groups and were instrumental in streamlining and centralizing event promotions across campus. Past events have included film screenings, therapy dog visits, and collaborations with university groups/divisions to host Pomodoro study sessions, topic specific presentations

from occupational therapy, physical therapy, and more. We will discuss our shift in focus since returning from the pandemic as well as highlight new initiatives and partnerships with campus communities. We will discuss best practices and offer potential ideas for relatively affordable projects as well as present data collected regarding campus interests. We will discuss how we utilized the data collected to convince administration to move forward with initiatives and space enhancements as well as discuss future plans to bring social innovation to our library.

Program Conclusions:
In 2024, we will continue to develop programming surrounding wellness and well-being and will launch a Reflection and Wellness Room in March. Feedback and data collected from the launch of the room, along with previous data collected, will be presented as well as lessons learned so far. Overall, the presentation will make a case for socially innovative initiatives in health sciences libraries.

Moving Out of the House (Librarian Program): Creating a New Medical Student Liaison Model

Track(s): Information Services

Gregg Stevens, Manager of Library Education and Clinical Services, University of Massachussetts Chan Medical School

Background:
Medical school X librarians had previously acted as liaisons to undergraduate medical students through a House Librarians program, aligning with the school’s six houses, or learning communities. Starting in 2022, however, two separate factors made this previous liaison model unsustainable. First, the school announced plans to increase class sizes substantially over four years, with a corresponding increase in the number of houses from six to ten. Secondly, the library saw heavy librarian turnover and family leave during the 2022-23 academic year, leaving most of the houses without a dedicated librarian for at least part of the year. A new medical curriculum, also introduced in 2022, provided potential opportunities for rethinking how liaisonship and outreach had been previously conducted.

Description:
In conjunction with faculty on the new curriculum’s leadership team, the library proposed to move away from the House Librarian model to the new Pathways Librarian program, which will instead support the seven thematic pathways of the new curriculum. These pathways, including Education, Health Systems Science, and Structural Inequity, Advocacy, and Justice allow students to explore aspects of medicine in depth. Students in these pathways will work on longitudinal group projects instead of the previous curriculum’s individual capstone projects; this will in turn change the nature of the types of research consultations handled by the librarians.

Librarians with interest and expertise in given areas chose their pathway affiliations. Due to the nature of some of the pathway specialties, librarians from other departments in the library chose to join the program. Because each librarian signed up for multiple areas, this created resiliency in the program’s staffing because each pathway had at least two librarians so there would never be a vacancy. The students benefit from having a choice of librarians with subject expertise. Early collaboration on the fall orientation sessions and pathways kick-off meetings for the new students allowed medical faculty and librarians to plan library instruction relevant to each pathway’s unique needs, instead of a big “one size fits all” session.

Program Conclusions:
More than 150 students were reached through introductory pathway sessions in October 2023, with eight librarians collaborating with medical faculty. It is hoped that further instructional opportunities and

consultations will result from this engagement during the current academic year. Communication and collaboration between the medical faculty and the librarians at all steps of the process has built trust and led to a successful rollout of the new program, ultimately allowing the House Librarian program to be sunsetted. We hope that, as the Pathways Librarian program matures, the level of embeddedness between the library and the new curriculum will increase even further.

New Ways to Engage Users and Provide Access to Primary Source Documents Arising From the Opioid Industry

Track(s): Information Management

Chris Shaffer, University Librarian, UCSF

Anne Seymour, Associate Dean and Director, Welch Medical Library, Johns Hopkins University

Kevin Hawkins, Program Director, Johns Hopkins University

Background:
This presentation examines a digital archives program that collects and makes available opioid industry documents for long-term public access and research. The program builds on a successful model to preserve tobacco industry documents, which since 2002 has supported over 1,000 publications about industry influence on health and led to groundbreaking change in national and global tobacco control laws and policies. The opioid industry and opioid epidemic are of widespread concern to health sciences librarians, as evidenced by education and public policy work by MLA, NLM, AAHSL, and other organizations.

Description:
The program brings together archivists, librarians, software developers, scientists, and historians at multiple institutions to develop a freely accessible digital archive of millions of opioid industry documents that can be used to learn from the opioid epidemic to improve and safeguard public health. The archive currently includes over 3 million opioid industry documents that can be cross-searched with other collections of previously confidential materials from the tobacco and other industries, including memos, reports, internal scientific studies, marketing and sales information, and public relations campaigns. These materials are used by scientists, journalists, lawyers, policymakers, and other stakeholders to investigate industry influence on public health. To reach a broader audience, the program includes a concerted effort to develop teaching materials drawing on the archive and to integrate use of the archive into curricula at universities around the country. The program has also contracted with a public relations firm to help engage journalists in reporting that draws on the archive, and the program is launching adjacent online resources meant to provide alternative paths to accessing the documents: a collection of powerful images found in the industry documents, and a toolbox of options for using computational methods to explore and analyze the archive documents.

Program Conclusions:
This presentation will describe current program goals, metadata and digital preservation considerations, outreach, evaluation methods, and collaboration opportunities. Outcomes include: 1) preservation of and public access to millions of opioid industry documents; 2) a growing number of scholarly and mass-media articles drawing on the archive; (3) growing user engagement through alternative paths to the archive.

No Bones About It: Developing Stronger Anatomical Model Collections Together

Track(s): Education, Information Management

Rachel Helbing, Head of Health Sciences Libraries, University of Houston Libraries

Stefanie Lapka, Interim Head of Health Sciences, University of Houston

Leonard Martin, Interim Head of Resource Management and Metadata, University of Houston Libraries

Background:
Like many academic health sciences libraries, we acquire anatomical models to support a range of health programs including but not limited to limited to kinesiology, medicine, nursing, and optometry. This paper will discuss how health sciences librarians collaborated with anatomy and physiology faculty to significantly expand the range of anatomical models at the institution's new allopathic medical school to support a multi-modal anatomy curriculum. An ongoing project between health sciences librarians and the head of resource management and metadata for the academic research library system to enhance discovery and speed circulation service of this nearly 200-item anatomical model collection is also a central focus of this paper.

Description:
Students, faculty, and librarians identified anatomical models as a collection need upon the opening of the new medical library. Librarians worked with anatomy and physiology faculty to identify the appropriate scope and makeup of the proposed collection. 67 models were selected for purchase, aligning with the integrated organ system-based biomedical sciences curriculum. Health sciences librarians connected medical school staff with the head of resource management and metadata to coordinate the ordering, shipping, receiving, cataloging, and processing of the collection.

The newly acquired models for the medical library augment a fairly robust legacy collection of 104 anatomical models located at the inclusive health sciences library; the collection now encompasses nearly 200 items across two library locations. For this complex collection, we wanted to improve discoverability and speed circulation service. Examining our goals, we concluded that providing enhanced catalog records and photographic previews could be beneficial for both library users and staff. We will discuss our project workflow from receiving to shelving the models. These cataloging and processing methods include creating a custom call number prefix and designing numeric ranges for the call numbers, linking to model keys, capturing and hosting photographic previews, and configuring physical locations.

Program Conclusions:
We aim to demonstrate that enhancing the catalog records for our anatomical model collection had a significant impact. By analyzing circulation statistics for both newly acquired and cataloged models as well as legacy models, we hope to show a measurable increase in usage for this collection compared to previous years. We also plan to collect feedback from catalog users and circulation staff to help determine the efficacy of including custom metadata, links to additional materials, and photographic previews for this collection. Several inherent challenges to this project will also be discussed, including the number of items on backorder, software limitations, time and staff constraints, and the nuances involved in processing unique formats in general.

No SLAC'ing Here: Reviving a Student Advisory Committee to Better Engage and Include Student Voices

Track(s): Education

Jenny Pierce, Temple University, Ginsburg Library

Courtney Eger, Learning and Engagement Librarian, Temple University

Natalie Tagge, Assistant Program Director, Research Advisory Services, University of California San Diego

Background:
Student advisory committees are an important tool in service assessments for health science libraries. They can also be used as an outreach tool, helping to get the word out about what the library has available for students. In 2021 librarians at an urban health science library decided to restart the library's student advisory committee. The library serves schools of dentistry, medicine, nursing, pharmacy, podiatry, and allied health at an R! institution. The previous iteration of the committee consisted of admin-nominated members that met once a semester for a boxed lunch. Meetings consisted mostly of library staff reporting out to students. It was difficult to get student members to attend and the committee ended due to lack of student interest.

Description:
Health science librarians decided to restart the committee in 2021. Reviewing the previous advisory board they decided to revise the entire process. The goals of the new committee structure were to increase student engagement by creating a new, more flexible student advisory group and create a sustainable group structure that wouldn’t dominate staff time. First the committee became a competitive volunteer opportunity. A marketing campaign was developed including social media, liaison librarian contacts, student affairs offices and orientations. Incentives for membership include a service letter from the director, swag bags, and an end of year gift. Secondly, meetings are held once a semester in a hybrid format, for months without a meeting engagement continues through a Canvas course. All library staff brainstorm feedback questions for the committee. Questions are used at both in-person meetings and for monthly online feedback. Involved library staff meet once a semester to assess how the committee process is going. Students complete an anonymous end of year survey allowing them to give feedback on the process. Student suggested action items are tracked and reported back to library administration.

Program Conclusions:
The program is a success. The committee received high scores from students in the end of the first year survey. In year two student application increased, including members wishing to remain on the committee, leading to an increase of representation.. Over the first two years student recommendations that were implemented included the creation of half hour library resources workshops by discipline, social media campaign on unique library resources, new board game and puzzle collections as part of an increased student wellness program, and development of new study spaces. Students gave feedback on a publishing support guide, developing a poster printing service and high stakes exam study tools. Staff time, while substantial, was lower than the previous iteration of the committee.

Onward & Upward: Highlighting the Contributions of Black Urologists through their Scholarship

Track(s): Education, Health Equity & Global Health, Innovation & Research Practice, Professionalism & Leadership

Bart Ragon, University of Virginia

Background:
The William P. Didusch Center for Urologic History curates an exhibit annually for the American Urological

Association's national meeting. The theme for this year's exhibit, 'Onward & Upward,' focused on the contributions of Black Urologists to medicine. The curatorial team, including a medical librarian, helped to create a publication network diagram illustrating the impact of Black Urologists on scholarship and medicine. The network diagram highlights not only the strong professional network created by Black Urologists, but their significant contributions of new medical knowledge, benefiting patients of all races and ethnicities.

Description:
The exhibit was designed to advance the dialogue surrounding the progression of Black Physicians in organized medicine and Urology, despite systemic racism in medicine: from exclusion to segregation to present day micro-aggressions and cultural insensitivity. The team consisted of prominent clinicians, educators, curators, historians, graphic artists, and librarians. The publication network was included in an exhibit that featured lectures, oral histories, panels, photos, and artifacts illustrating the impact of Black Urologists past and present. Initially, a list of 42 prominent Black Urologists was identified to seed the diagram, resulting in a visualization covering 8,800 coauthors across more than 4,700 publications. Publications were compiled using Web of Science's algorithmically generated author records, and names were disambiguated in Python using a combination of affiliation information and fuzzy string matching which estimates the similarity between non-identical words. VOSviewer generated the data visualization, which was presented at the AUA national conference in May 2024. The coauthor network illustrated an intricate web of interinstitutional collaborations that bridged disciplines and domains expertise. Additionally, a separate keyword network was generated, showcasing a wide range of healthcare topics spanning the spectrum of contemporary Urology.

Program Conclusions:
The goal of this project was to raise awareness about the invaluable contributions of Black Urologists that ultimately lead to improved outcomes for physicians and their patients. As a part of the overall exhibit, the interactive display effectively highlighted the many contributions of Black pioneers in the field of Urology while showcasing Black excellence in academia, workforce development, and leadership. Interest and awareness will be measured using an attendee scanner at the digital display.

Passing the Baton: Peer Mentorship and Succession Planning in the Retention and Cultivation of Professional Relationships

Track(s): Information Services, Professionalism & Leadership

Cindy Runnels, Supervisor, Library Services, Kaiser Permanente

Kristyn Gonnerman, Supervisor, Library Services, Kaiser Permanente

Background:
The departure of long-term librarians frequently means the loss of institutional knowledge and the emergence of service gaps as remaining librarians struggle to take on new responsibilities and form vital professional connections with key personnel/clinical departments in their new service lines. This paper provides an overview of a retiree-initiated peer-based succession-planning partnership intended to provide a 'warm hand off' and promote continuity of library services to the Regional Nursing Research Council of a large healthcare organization.

Description:
With a planned retirement date established for the 'primary' librarian, a peer-based succession and mentoring program was set into motion. A 'secondary' librarian stepped in as an associate library liaison to the Regional Nursing Research Council, partnering with the primary librarian and providing additional support for research projects, publications, posters, and a multi-day Evidence Based Practice Bootcamp.

The librarian team added monthly updates on current library/information topics to each Nursing Research Council meeting to highlight library and information services and administered periodic online surveys to gauge overall satisfaction with librarians/library services support, and to gather suggestions for improvement and additional topics of interest. This program provided opportunities for the librarians to raise their professional profiles with the nurse researchers within the organization, leading to increased opportunities to develop workshops, support region-wide MAGNET accreditation efforts, and gain authorship credit and experience with collaboration on integrative reviews.

Working in tandem, the ‘primary’ and ‘secondary’ librarians gradually reversed roles, allowing for a smooth transition of tasks and responsibilities, and the maintenance and continued cultivation of critical professional relationships.

Program Conclusions:
Forming a peer-based succession planning partnership has proven beneficial in the gradual handoff of responsibilities, the continuity of library services, and the maintenance of critical professional partnerships with this influential and active nursing research group. In this case, the prospective retiree initiated the succession plan since the retirement date was set well in advance. Where possible, transparent discussions involving peers and management should begin well before a retiree’s actual departure to allow for a well thought out plan including documentation, mentoring, and communication with other stakeholders to ensure the continuity of services and the retention of relationships cultivated over time.

Project Management Level Up: How The Right Tools and Training Can Improve Collaboration in Your Library

Track(s): Professionalism & Leadership

Tiffany Garrett, Director of Library Services, Roseman University of Health Sciences

Background:
The Roseman University of Health Sciences serves health professionals in pharmacy, nursing, and dental medicine. The university has an emerging College of Medicine with the first cohort of students expected in 2025. Roseman University has a current enrollment of around 1,300 students with an ambition of growing to 5,000 students in the next five years.

All this growth has required a very intentional approach to capacity building within the library so that library staff, resources, and systems are all prepared to serve the higher volume and variety of students. For these reasons, as the new Library Director in 2022 I began building a project management culture that would support the changes required to meet the needs of a growing institution.

Description:
The project management process utilized at Roseman University is based on project management principles such as Objectives and Key Results (OKRs), the RACI model, and the Kanban methodology. We use MS Planner and Teams heavily to organize the work and communicate it. I am currently in year 2 of 3 to train team leaders on the methods and tools.

I introduced the library staff to project management methodologies in 2022 by leading an RFID conversion and shift to an extended access model where the library would be accessible to students outside of staffed hours. This allowed me to show all library staff how to set meaningful objectives, track deliverables, communicate transparently throughout the process, and report on the results.

In 2023, we collaboratively set 12 team goals and I trained team leaders on how to lead a project. We also experimented with tracking deliverables in Excel and MS Planner. Additionally, I held project leads accountable for reporting progress in regular library planning meetings.

In 2024, I plan to train team leaders on how to manage their projects and track deliverables more directly in MS Planner. I will also provide more support and training based on feedback from 2023.

Program Conclusions:
The library’s project management methodology is assessed through progress on the projects themselves, as well as feedback from library staff. Project progress results will include the status of 2023 projects, key results, and informal feedback from projects that were not completed or had major changes to deadlines or deliverables. Library staff feedback will be collected through an anonymous end-of-year survey as well as one-on-one meetings with supervisors. In this presentation I will share how that feedback informed changes to the process in 2024, but the direct feedback and results of the survey are kept confidential to preserve the integrity of the results.

Providing Health Information to Patients: An Elective Experience for Fourth Year Medical Students

Track(s): Education, Information Services

Dana Ladd, Health and Wellness Librarian, VCU Libraries

Background:
Healthcare providers have a responsibility to provide patients with reliable and understandable information that patients can use to make informed decisions regarding their health. To enhance provider knowledge of health information dissemination to patients, the librarian created a non-clinical elective experience for fourth year medical students. Typically, the experience was via direct library observation, hands on experiences, and synchronous one-on-one learning sessions. In order to provide a more standard and consistent learning opportunity, we created seventeen health information learning modules and delivered the content via Canvas where students could work asynchronously. This paper explores the update of the elective through the creation of learning modules on Canvas, a web-based learning management platform.

Description:
The learning modules were developed to supplement the observational and hands-on experiences of the students. Through completion of the learning modules and activities in Canvas, students learn the importance of providing reliable and current health information to patients and their family members. Students will learn how to identify, evaluate, and access relevant consumer health information resources (print, audiovisual) on a variety of relevant health topics and formats, languages, and literacy levels. The learning modules will also cover topics such conducting the reference interview, information ethics, finding health information for varying patient demographics, health literacy basics, numeracy, and plain language concepts. Students will have the opportunity to complete assignments via Canvas to build skills and then use those skills during their hands-on observation experiences in the library, conducting information rounds, and participating in library programming and outreach activities.

Program Conclusions:
Two non-students reviewed the Canvas learning modules and provided feedback on ways to improve the

Canvas learning modules. Since the completion and implementation of the Canvas learning modules, two medical students have completed the elective experience. The students spent on average 35 hours completing the learning modules and activities and assignments in Canvas and spent about ten to twelve hours per week on site observing and participating in hands-on reference services and programming. Students provided verbal positive feedback regarding the content of the learning modules. Both students expressed some concern regarding the learning curve of using Canvas and one student expressed concern about making sure they understood their responsibilities and the assignments due dates.

Scoping Reviews in Library and Information Science

Track(s): Innovation & Research Practice

Alexia Sheck, Librarian, University of St. Augustine for Health Sciences

Julie Evener, Director for Content Management & Discovery, USF Health Libraries, University of South Florida

Matthew Chase, Instructional Services Librarian, Cuyamaca College

Jessica Cain, Librarian, Central Arkansas Veterans Healthcare System

Background:
First, we wanted to increase our familiarity and expertise regarding scoping reviews. We aimed to immerse ourselves in a better understanding of scoping reviews so that we could better relate to and support researchers at our respective institutions. We decided the best way to do that was to engage in a scoping review project of our own. Our second goal was to document and outline a scoping review process applicable to the library science field. While it would be best practice for information professionals to follow the same scoping review process and reporting guidelines established by other disciplines, there may be additional recommendations or considerations unique to library and information science.

Description:
Pursuant to gaining expertise to help us better support the researchers at our respective institutions, we followed scoping review methodology and reporting structures common in health sciences. The process we followed included six steps.

1. Identifying the research question.

2. Identifying relevant studies.

3. Study selection, which included a title and abstract review, full text scan, and article labeling.

4. Charting the data.

5. Collating, summarizing, and reporting the results.

6. Optional stage, consultation exercise, which we found to be not as relevant in the library science field as in medical and health sciences.

We also paid close credence to the JBI guidance for conducting scoping reviews and the PRISMA model for scoping review reporting, PRISMA-ScR.

Program Conclusions:
In all, we found the best practices for scoping reviews established for medical and health sciences to be applicable to library and information science scoping reviews as well, though considerations like document type (e.g., documents describing programs versus scientifically constructed research studies) and time

management (e.g., prioritizing the scoping review while balancing daily responsibilities in the library) have substantial implications in library and information science. We also felt that the experience of tackling a scoping review helped us better understand and support researchers coming to the library for help with these projects.

SEE-ing Results in LibGuide User Testing: Applying the SEE Protocol for LibGuide User Testing

Track(s): Education

Emily Espanol, Research & Learning Librarian, Roseman University of Health Sciences

Background:
When the expanded library team of a health sciences university approached its LibGuide redesign, they found various approaches to pedagogy and formatting across the 36 LibGuides created and inherited by a series of solo librarians. Usage numbers were up, but very little was known about actual use. The library team recruited students for a series of task-based usability tests as described in the literature but desired more explanation behind user decisions. The purpose of this round of LibGuide assessment was to develop a collaborative user testing room that welcomed user’s narratives. After further investigating UX research, the team discovered the Satisfaction, Effectiveness, and Efficiency (SEE) Protocol which allowed the user to reflect on two new criteria, Satisfaction and Efficiency.

Description:
A literature review revealed a simplistic model of user testing in libraries that uses a pass-fail rubric to assess a user’s ability to fulfill a common information need using a LibGuide. Of course, speak-aloud protocols were heavily used but post-test user reflection was seldom mentioned. The library team discovered that the time that was usually used to thank the participant was better spent venting, or asking about the user’s emotional highs during testing.A look into the UX literature revealed that the library team had stumbled into asking about user satisfaction and efficiency. After the LibGuide redesign, the team returned to test the new design and codified the “venting session” with three questions that ask users to rate and reflect on their satisfaction with the LibGuides and their perceived mental effort while searching for information. This way, not only does the library obtain more qualitative data but it also builds relationships between librarian and user.

Program Conclusions:
We expect the new criteria will support the efficiency data in our LibGuides usability tests. A rating scale with user input and narrative will help librarians dive deeper into their users’ information behaviors. While testing continues, we acknowledge that this may further help the librarians understand the information behaviors of their disciplines– pharmacy, nursing, and dental medicine– and make evidence-based design choices specifically for their users.

Six Years of CAIFL: Pushing the Limits of Librarianship Together

Track(s): Education, Professionalism & Leadership

Marie Ascher, Lillian Hetrick Huber Endowed Director, New York Medical College

Abraham Wheeler, Health Sciences Librarian, Michigan State University

Rachel Pinotti, Owner and Operator, Pinotti Information Services, LLC

Amy Blevins, Associate Director for Public Services, Indiana University School of Medicine

Background:
The overall goal of the Critical Institute for Librarians (CAIFL) is to enhance the abilities of health sciences librarians to take EBM training to the next level – to develop competencies in biomedical statistics, scientific methodology, and clinical decision making; and to enhance confidence and comfort of librarians in teaching the concepts of critical appraisal. To this end, in 2018 we were awarded an NN/LM grant to plan and execute training for librarians in critical appraisal. This paper will describe the course, outcomes, reflections, and future directions.

Description:
A cross-institutional team of librarians with knowledge of clinical epidemiology was formed as the program planners. We enlisted a physician EBM educator known to provide relevant internet videos and developed the curriculum. The completely online course is composed of two plenary sessions, five modules, peer presentations, participant led journal club presentations, IRATs, a pre-test and post-test, and an ongoing community of practice. We have conducted this course annually since January 2019 to a total of 202 medical librarians.

Program Conclusions:
While five years of the program has been completed, this paper will report through the sixth year of the program. We have pre-test and post test data and new initiatives in our community of practice that will be included in the final abstract. The feedback for this course is overwhelmingly positive but indicates the need for further and more in-depth instruction in this area.

Strategic Planning for Data Equity in a Department of Public Health: Challenges and Opportunities

Track(s): Health Equity & Global Health, Information Management

Pam Lough, Ontologist, California Department of Public Health / CDC Foundation

Abera Gelleta, Clinical Informatician, California Department of Public Health / CDC Foundation

Scott Fujimoto, Public Health Medical Officer, California Department of Public Health

Background:
Public health efforts to be more inclusive in monitoring data for race and/or ethnicity, sex orientation and gender identity, and at-risk communities have sparked a number of initiatives that may be of interest to librarians working with data or health equity. Disaggregation of data to better represent different health concerns of people with different origins, lifestyles, and/or identities is essential to support better planning for

public health. These initiatives are, in part, to comply with legislation mandating the collection of more detailed data for race or ethnicity than the data most recently collected by the US census. This talk describes some of the work being done in a department of public health to strategically plan to improve data equity.

Description:
Task forces and committees in public health are working to address challenges in disaggregation of data such as:

1) Sourcing detailed data for race and/or ethnicity,

2) identifying denominators for individuals of more than one race or ethnicity,

3) how to analyze data that has been collected over time with differing levels of granularity,

and

4) how to gather data for small populations without compromising privacy.

A scoping review of literature on demographic data collection and analysis for race, ethnicity, and nationality was conducted in 2023. The literature review explored definitions of race and ethnicity and methodologies for identification of race and/or ethnicity, methods for collecting data, and methods for analyzing data. The review was used to inform group objectives and to help find solutions to challenges faced by the groups. The groups have created subsections to work on some of the specific challenges.

Program Conclusions:
Outcomes the groups anticipate measuring include the development of techniques for determining denominators for individuals of more than one race or ethnicity, for analyzing data that has been collected over time with different levels of granularity, and for gathering data for small populations without compromising privacy. The ultimate outcome will be whether this information is enough to ensure that relevant legislation has been properly implemented.

Strength in Numbers: Using Data on Library Literature Searches to Measure Impact on Evidence-Based Patient Care and Hospital Policy/Procedure Changes

Track(s): Clinical Support

Lindsey Gillespie, Medical Librarian, Dignity Health

Michelle Lieggi, Clinical Education Librarian, Dignity Health

Background:
Hospital leadership is increasingly requesting metrics from its libraries to justify their value and continued existence in their organization. Although the value of hospital library services has been demonstrated, measuring their impact on direct patient care and other hospital-based outcomes is challenging. Yet, determining impact is critical to promoting the continued existence of library services. To better determine our impact on hospital-based activities, the Library examined 2 outcomes – direct patient care encounters and changes to hospital policies/procedures. We then reviewed data from past clinician literature search requests to determine whether the results contributed directly to either of these outcomes.

Description:
After reviewing the retrospective data, the Library determined that is was not sufficiently robust for tracking these outcomes and that a new method was needed for collecting data from clinician requests. In late 2023, the Library acquired a library tracking system and used it to create a custom literature search request form. The form includes a required field for users to indicate why they needed evidence-based literature on the topic, including whether it was for a direct patient care encounter or to update a hospital/system wide policy or procedure. The form will be piloted on the Library website in early 2024. Data entered on the form will be captured in the tracking software database. After an initial 3 month pilot, the Library will analyze the data to determine how many requests required evidence-based literature for a direct patient encounter or for a policy/procedure change.

Program Conclusions:
For those requests where a direct patient care encounter or policy/procedure change was indicated, the Library will follow-up by sending a brief survey to the users that asks whether the literature was actually used for those purposes. The confirmed data will then be summarized into a report for hospital leadership, and trended going forward. Long term, the Library hopes to fully implement the request form and use it to analyze other outcomes, such as use of library services to support continuing education, quality improvement, and organizational initiatives. These metrics will be used to demonstrate the value of retaining library services across the organization.

Strengthening Self-Directed Learning: Librarians and Faculty Collaborate on Curriculum

Track(s): Education

Megan von Isenburg, Associate Dean for Library Services & Archives, Duke University Medical Center Library

Samantha Kaplan, Liaison to Doctor of Medicine Program, Duke University Medical Center Library

Background:
Self-directed learning (SDL) experiences are required for institutional accreditation of medical schools by the Liaison Committee on Medical Education (LCME). To meet LCME standards, SDL experiences must include four components: identification of learning need; retrieval, analysis, and synthesis of information; appraisal of sources; and feedback from instructors. SDL is aligned with skills taught by librarians, including information literacy and evidence-based medicine, and those taught by basic and clinical science faculty, including critical thinking and constructing foundational medical knowledge. To leverage disciplinary expertise, librarians and faculty must collaborate. This paper will describe the collaborative development of a curricular thread for teaching and assessing medical students' SDL skills in the preclinical curriculum.

Description:
While the institution's curriculum already included several librarian-led sessions on information literacy and a required course on evidence-based medicine (EBM) co-directed by a librarian, there was not an explicit SDL assignment. To address this gap, librarians partnered with faculty to develop a deliberate, progressive, and clearly labeled set of SDL activities for first year medical students. Three learning activities were created across different domains: foundational medical knowledge, critical reflection, and clinical information needs. Each activity utilized an SDL activity as a pass/fail meta-cognitive assignment paired to an existing assignment. In each assignment, students were required to identify a learning need related to completing the original assignment, to select a relevant source, to evaluate the source, and to analyze the information retrieved. All students completed and received credit for the assignments. Assignments demonstrated student information seeking and evaluation skills at three distinct time points and in three different domains.

Librarians provided feedback directly to students for the critical reflection and clinical information assignments and provided a rubric for faculty feedback on the foundational medical knowledge assignment.

Program Conclusions:
Student responses highlighted information literacy skills that range beyond EBM (e.g., PICO, searching, and appraisal), including author voice, conflicts of interest, and the role of geography and power in research. Librarians gained a deeper understanding of student skills in information literacy and appraisal; and faculty gained a deeper understanding of source selection and evaluation. Librarians were also granted additional teaching time in busy curricula to ensure student preparation for these assignments. Healthcare providers increasingly must navigate misinformation and potentially biased information from artificial intelligence, flawed research, or faulty sources. Accreditation requirements for self-directed learning can enable librarians to expand existing information literacy teaching and evaluation to address these needs.

Strengthening the Workforce through the Health Sciences Librarianship Apprenticeship Program

Track(s): Professionalism & Leadership

Rachel Lerner, Research & Instruction Librarian, Quinnipiac University

Becky Morin, Associate Director Research & Instruction, Hirsh Health Sciences Library, Tufts University

Elena Schnerr, Southern Connecticut State University

Eugenia Opuda, Health and Human Services Librarian and Associate Professor, University of New Hampshire (UNH)

Sabrina Brown, Simmons University

Background:
Hiring qualified entry-level health sciences librarians is becoming increasingly difficult. Immediate postgraduate hires have received little training in health sciences librarianship. Despite 51 ALA-accredited LIS programs listing a health sciences course, these classes are offered infrequently. Four major but interrelated obstacles facing the field are: a lack of established school-to-career pipeline; a need for new practitioners to learn on the job, thereby decreasing entry-level readiness; a resource-intensive onboarding process for new practitioners; and a workforce that begins their career with a sense of uncertainty and imposter syndrome. Our project aims to address gaps in the library curriculum and prepare a select number of current students for success in health sciences and medical libraries.

Description:
The Health Sciences Library Apprenticeship Program (HSLAP) is a 4-month opportunity for library students entering their final year of study. The pilot year was fully funded, developed, and staffed by the MLA North Atlantic Health Sciences Libraries chapter. Project coordinators sent a call for applicants to area library schools and selected two mentees to participate. Over the course of 12 weeks, mentees completed four modules of online didactic instruction and a week-long on-site practical immersion experience at a local library with an assigned mentor. The online component consisted of NNLM and MLA training courses, journal club and other readings, activities, and presentations from local library directors with live discussion. Mentees received a per diem to cover gas, parking and lunches for the on-site immersion experience. During that time, mentees developed at least one presentable artifact to be used in the job interview process. Additionally, mentees developed a 15-minute PubMed class and presented it to the group for practice and constructive criticism, with the goal of simulating a job talk. Finally, mentees attended the NAHSL conference to network and present a poster about their experience.

Program Conclusions:
Post-program feedback sessions were conducted online with both mentees and mentors in two separate meetings. That information is currently being collated but the initial impressions are positive with constructive suggestions for the future. Plans for expanding the program are in formulation, including inviting other chapters to participate and looking for a more sustainable funding source. The project coordinators will present the specific feedback provided by mentees and mentors, highlight the benefits and success of the program, and address changes to the program based on participant and chapter responses. Coordinators will also present a curriculum and other supporting materials for those interested in adopting or expanding the HSLAP program in their chapters.

Stronger Together: Libraries, Their Governing Bodies, and Our Worth

Track(s): Professionalism & Leadership

Adela Justice, Sr. Librarian, UT MD Anderson Cancer Center

John Mokonyama, Medical Librarian, Penn Medicine, Chester County Hospital

Background:
Libraries are not in the business of making profits for their institutions and governing bodies, focusing instead on bringing value to their users. A health sciences librarian at a medium-sized hospital was tasked with determining and providing a monetary dollar amount for the value their library brings to the hospital. This request was brought by their new supervisor as part of a revised monthly and annual reporting system. Utilizing various methods, the librarian will demonstrate the proposed monetary evidence of value that their library brings to the hospital as well as share the process for how that figure was decided upon.

Description:
The librarian accomplished this value-finding task using a systematic approach. First, they took inventory of all the library's services and resources. Next, they gathered the statistics and metrics that are used to track library services, such as number of reference questions, literature searches, materials circulation numbers, etc. Qualitative tracking was used when applicable, including observations of users' learned research skills, case studies, and any user stories/testimonials. The librarian was also careful to note information about the library's continuous service improvements and how the improvements brought savings to the library's bottom line. The final step was to benchmark the library's services and resources with other libraries in order to present a comparative analysis of the library. The librarian also queried other health sciences librarians via listserv to see if other librarians had been asked to provide monetary evidence of their library's value, and if so, how they arrived at the figure. The librarian's team also conducted a literature search to see if any other types of libraries—including public, school, academic, and special—had received such a query.

Program Conclusions:
The question the supervisor asked about the library placing a dollar amount on their value, and therefore, return on investment from the hospital is a fascinating one. This librarian was able to come to an estimate and present it to their supervisor, showing that the cost of keeping and maintaining a library is far under the overall value a library brings to its governing body, even when you translate those values to dollar amounts. Other library workers may find this process useful for determining a dollar value of their own library, either as an item of interest on an annual report or, more seriously, as preemptive data to have on hand during times of budget cuts.

Stronger Together: Supporting Student Success with Cross-Campus Collaboration

Track(s): Education, Information Services

Elizabeth Coghill, Executive Director, Student Academic Success Services, East Carolina University, East Carolina University

Jeffrey Coghill, Outreach Librarian, East Carolina University

Background:
Within the backdrop of Institutional strategic goals emphasizing student success, the retention and degree attainment of our students in post-secondary learning environments becomes the mission of all campus partners. Recognizing the need for new connections between campus partners, session presenters address how cross-campus expertise and collaboration is applied to student success and initiatives within the Provost Office and Academic Affairs. The session will highlight LibGuide utilization in establishing a theoretical foundation and framework to share with both on campus and external stakeholders.

Description:
Moving beyond the traditional use of research guides to support course instruction, this session introduces an innovative utilization of LibGuides to foster cross-campus collaborations in support of university strategic goals and student success initiatives. Redefining the traditional campus roles of librarian and student success administrator, session presenters will highlight the use of collaborative research to establish LibGuides as a theoretical framework within student success programs and services. Presenters will explore the use of LibGuides in cross-disciplinary processes within four student success initiatives and will identify ways other campus librarians can leverage library resources in support of campus strategic initiatives outside of classroom instruction. Finally, the session will introduce new techniques in building LibGuide formats and data collection processes for partners outside of the library learning environment.

Program Conclusions:
Session attendees will: 1) explore the use of LibGuides in cross-disciplinary processes, 2) examine the relationship between campus partners and student success initiatives, 3) identify ways to leverage library resources in support of campus strategic initiatives outside of classroom instruction, 4) learn new techniques in LibGuide formats and data collection processes.

Supporting Arts in Medicine and Health Humanities Through Collaboration

Track(s): Education, Information Services

Chloe Hough, Reference and Instruction Librarian, Nova Southeastern University

Prasanna Karur, Nova Southeastern University

Sherilyn Nguyen, Nova Southeastern University

T. Brandon Hall, Nova Southeastern University

Tariq Rahaman, Reference and Instruction Librarian, Nova Southeastern University

Background:
The [library] launched with the newly built campus in 2019 and serves several programs including medical, dental, nursing and health sciences. Since reopening the campus post-pandemic and hiring new staff, the relatively small team is in the process of creating relationships among faculty and students in order to maximize the utility and impact of the library. One of the library’s goals is to support emerging health humanities initiatives on campus in order to foster the development of social-emotional skills and promote creativity and stress relief. To meet this goal, the librarians have worked towards launching a graphic medicine collection, book discussion, and two campus-wide events in collaboration with student interest groups.

Description:
The team at [library] have worked toward launching a graphic medicine collection to supplement the small reserve print collection and promote health humanities. Additional books will be added, including suggestions from the College of Medicine’s Medical Humanities student interest group. Alongside the launch of the collection, the library plans to implement accompanying programming such as a book display, book discussion, and health humanities workshop.

Also in collaboration with the student interest group, the library is currently planning an artwork showcase event in Spring 2024. Students from any program on campus will be invited to submit artwork, writing and photography of various kinds to be printed and displayed for a month-long exhibit. The exhibit will have a kick-off event co-sponsored by the library and student affairs. The library has also collaborated with the medical school’s Photography Club to host their project, Humans of [Institution], featuring photos and brief stories in the style of Humans of New York. The collected pictures and quotes are a showcase of the unique stories of the interviewed students and staff members. The collection has been framed and mounted on campus and is archived in [the University archive]. Future events will continue to be archived.

Program Conclusions:
While the Humans of [Institution] exhibit was a passive, walk-up experience, the library hopes to evaluate the upcoming medical humanities showcase via number of art submissions and number of attendees at the opening night event. Participation in the future graphic medicine book discussion, as well as circulation metrics on the graphic medicine books, will also help in evaluating student and faculty interest in this kind of programming and collection.

Take a Walk on the Wild Side: Walking Guides for Self-Directed Nature-Based Therapy

Track(s): Education, Information Services

Ariel Pomputius, Health Sciences Liaison Librarian, University of Florida

Nina Stoyan-Rosenzweig, Senior Associate in Libraries, University of Florida

Background:
Data supports the effectiveness of nature-based therapy to promote wellness, ease anxiety, reduce physiological stress, and enhance the immune system. However, interacting with nature or understanding the natural world fully can be difficult when one lacks experience with local flora and fauna. While nature walks with a guide can be helpful to share knowledge, finding the time to schedule a formal event can be limiting. As a neutral space that serves several health science colleges and is neither classroom nor clinic,

the Library is uniquely suited to support visitor wellbeing. The Library’s Wellness Team created nature guides as a passive program to encourage visitors to take a break in the natural spaces around the library at their convenience.

Description:
In order to streamline the process of editing and printing the guides regularly, the team created a template of four photos and descriptor spaces on the front of the half-sheet guide, with a suggested walking route starting at the library entrance on the back. Guides included descriptions of unique behavior and locations where these animals and plants could be sighted on campus along the recommended route and were updated regularly to reflect the changing seasons. Photographs and descriptions were supplied by a library employee with experience in environmental education and a passion for exploring local wildlife.

Guides were printed in-house and made available near the library entrance and in a clinical space near the recommended walking path. Instructors also distributed the guides when their courses intersected with nature-based therapy, including health humanities courses, discussion seminars, and a unique herbology elective. In addition, guides were made available online through the Library’s LibGuide and were featured in the Campus Nature Rx newsletter.

Now that the format of the guide is developed and word is spreading, the team looks to advertise the guides more heavily on the Library’s social media.

Program Conclusions:
Evaluation has been discussed, but no formal assessment has been carried out beyond tracking the number of guides taken from various locations and for different activities. The guides have been anecdotally well-received by students, staff working in wellness, and instructors for courses related to nature-based therapy. The team is curious to see if promoting the virtual guides available on the Library’s LibGuide will increase engagement, in addition to the response to the physical guides.

Teaching Librarians About NLP Tools for Clinical Text De-Identification

Track(s): Clinical Support, Education, Innovation & Research Practice

Katie Pierce Farrier, Data Science Strategist, Network of the National Library of Medicine, Region 3

Christine Nieman Hislop, Data Education Librarian, University of Maryland, Baltimore

Background:
Before sharing clinical data, personal and sensitive information must be removed or de-identified, which is often a repetitive, time intensive process. There are freely available tools that use natural language processing (NLP) to de-identify clinical text. These tools can be used to produce HIPAA compliant data that can be more safely shared and help researchers comply with data sharing policies.

Description:
Librarians created a one hour webinar intended to introduce information professionals to artificial intelligence (AI), NLP, and provide fundamental context about how they work. The class explored openly available tools that use NLP to find and redact personally identifying information, and discussed the distinctions between privacy, de-identification, anonymization, and HIPAA compliance. Learners were provided with outreach examples and encouraged to brainstorm ways to promote these tools to their researchers.

Program Conclusions:
AI tools continue to grow in use and popularity. Librarians need to better understand available tools and ways to incorporate them into their outreach and instruction. We plan to pilot the course in the spring. Preliminary data and feedback about the class will be shared.

Turn Down the Volume, Turn Up the Skills: Addressing Consult Volume through Co-curricular Graduate Student Programming

Track(s): Education, Information Services

Carrie Baldwin-SoRelle, Health Sciences Librarian, UNC Chapel Hill

Karen Grigg, Health Sciences Librarian for Collections and Instruction, UNC Chapel Hill Health Science Library

Background:
The public health liaison team comprised of a dedicated liaison librarian and one to three additional health sciences librarians has been working for several years to address high individual consultation volume through curriculum-integrated instruction in a large school of public health. The librarians currently reach all Master of Public Health (MPH) students in at least one required class each spring, plus additional concentration-specific and elective classes. While evaluating curriculum-integrated instruction, we identified a few reoccurring periods with a high volume of non-course-specific individual consultations. Students often sought assistance with skills not covered in depth by existing library instruction in core classes: locating grey literature, public and practitioner-focused training resources, and systematic reviews.

Description:
While course-based integration has been successful, librarian capacity has been overwhelmed by the high volume of consultations. To meet these students' needs, we identified three opportunities for intervention: 1) MPH student orientation; 2) MPH summer practicum assignments; and 3) master’s papers with a systematized review requirement. Next, we identified and approached key administrative and faculty stakeholders.

With stakeholder buy-in, we designed programs based on documentation they provided and our perception of student needs. The interventions varied in both format and content and included course management system-integrated digital learning objects, workshops, and documentation.

We advertised the sessions through academic advisors and program leads, allowing us to reach a targeted student audience. The "Public Health Practicum Bootcamp" workshop uptake was particularly popular, offering students literature search and grey literature identification skills when needed most. The workshop was offered to both residential and online students, and held online so students who were potentially on-site at their practicum assignments could attend. The orientation modifications unfortunately proved to be the least effective, as the prepared content was excluded from the required orientation modules. However, the content was adapted for other health affairs schools and is

Program Conclusions:
The co-curricular programs offer an opportunity to provide library support, while introducing students to skills for use beyond their coursework. We will evaluate all programs over the next two years. Outcomes of interest are student skills and feedback, collected through attendance, post-workshop surveys, and from program coordinators; annual evaluation of librarian consultation load; and relationship building with school of public health leadership.

The first set of practicum workshops was highly effective. An informal comparison of statistics between summer 2022 and summer 2023 shows that one-on-one meetings were more evenly distributed across months, rather than a heavy load of consults in May, when practica begin. The systematized review support session is currently in progress and will be evaluated during spring 2024.

Using a General Survey to Determine Value: One Health Sciences Library’s Experience Customizing the MISO Survey

Track(s): Professionalism & Leadership

Robyn Gleasner, Resource Management Librarian, University of New Mexico

Sally Bowler-Hill, Manager, Administrative Operations, UNM Health Sciences Library and Informatics Center

Ingrid Hendrix, Division Head, Research, Education and Clinical Information Services, University of New Mexico

Kristin Proctor, Resource Sharing Coordinator, UNM Health Sciences Library and Informatics Center

Laura Hall, Division Head, Resources, Archives & Discovery, University of New Mexico

Lori Sloane, Data Manager, University of New Mexico Health Sciences Library & Informatics Center

Melissa Rethlefsen, University of New Mexico, University of New Mexico

Background:
The purpose of this study was to obtain reliable data about satisfaction with and value of services and resources to users at a health sciences library using the Measuring Information Service Outcomes (MISO) Survey. The study team evaluated multiple survey options including MISO, LibQual, LibStat, and creating an in-house survey. The ITHAKA survey was not considered due to its length and complexity. We selected MISO based on its flexibility/customizability, professional administration, ease of use, previous use of the tool, and historically high response rates. The team significantly customized the survey tool, adding 40 customized or local questions. The survey was distributed in February 2023 and sent to a sample of 4,223 faculty, staff, and students.

Description:
The survey received a 43% response rate, with a 38% completion rate. Custom questions that had been used before, thus providing longitudinal data, and custom questions with free text responses provided the most useful data. Data from the survey is being used to inform instructional offerings and plan furniture for a large remodeling project. However, some custom quantitative questions and several of the questions used from MISO’s question bank provided incomplete or uninterpretable data. Some respondents expressed dismay at MISO’s mandatory race/ethnicity and gender questions, data the study team did not need for our intended use. As with previous administrations, the amount of email sent to the campus survey administrator by respondents was unmanageable due to the highly personable nature of the survey invitation templates. Additionally, each administration of the MISO survey has been progressively more difficult to run though a biomedical IRB, showing gaps between IRB’s at the undergraduate institutions MISO predominantly serves and the IRB this library uses.

Program Conclusions:
Despite the obstacles, health sciences libraries should seriously consider using general library customer satisfaction surveys. The professional vetting and administration that goes into a fee-for-use survey like MISO provides significant value in terms of its usability, both for the survey team and potential survey respondents. The tools provided to help administer and monitor the survey, as well as the rigor of

participating in a cohort minimize distractions and keep the survey project on track. The data can also be used as a baseline for more targeted in-house assessments that dig deeper into specific areas.

Using iCite to Generate Bibliometric Reports to Support Evidence-Based Decision Making

Track(s): Information Services, Innovation & Research Practice

Paul Albert, Associate Librarian, Weill Cornell Medicine

Terrie Wheeler, Library Director, Weill Cornell Medicine

Background:
This project aimed to develop an open-source tool that leveraged publication data and metrics from NIH's iCite to generate bibliometric reports. These reports provided valid, field-normalized metrics to support data-driven decisions about faculty scholarly output at academic institutions.

Description:
We developed scripts to retrieve data from iCite and calculate metrics for a researcher's publication set. We integrated this into our institutional publication manager, ReCiter, to allow on-demand bibliometric report generation. After prototyping, we released the open-source code and documentation to enable adoption at other institutions. The evaluation of this service is in progress.

Program Conclusions:
This tool empowered administrators with instant, authoritative bibliometric reports to inform high-stakes decisions about faculty. The open-source availability also advanced transparency and equity in faculty evaluation. Our evaluation plan is to assemble and solicit critical feedback from a panel of end users and stakeholders (including representatives from Faculty Development, Institutional Reporting, and faculty members) to evaluate for utility, appropriateness, ease of use, and correctness.

Using Principle-based Instruction to Overcome Challenges in Teaching Systematic Review Methods Across Disciplines: A Workshop Case Study

Track(s): Education, Information Management

Jackson Hoch, Evidence Synthesis Librarian and Library Liaison, Newman Library, Virginia Tech

Cozette Comer, Evidence Synthesis Services Coordinator, Virginia Tech

Background:
Systematic reviews (SR), abundant in health and medical sciences, are proliferating across other disciplines at an exponential pace. As a result, library employees who have traditionally supported SRs in health and medicine are now being asked to translate these methods to other subject areas. In our library, within a public research institution, we established a principle-based, scaffolded educational program designed to support the application of SR methods across disciplines. In this presentation we describe our approach, specifically highlighting the benefits of focusing on SR principles. Although this approach is threaded throughout our services, we highlight our recurring multi-day, activity-based workshop as a case study.

Description:
We characterize our “principles-based” approach as instruction that emphasizes the underlying characteristics, values, and expectations of the method, why those characteristics are important, and how the stages of a systematic review work toward those values and help review teams to meet the expectations of their intended audience. Our approach is not an uncommon pedagogy. For example, it is reminiscent of the ALA Framework for Information Literacy for Higher Education which focuses upon “interconnected core concepts…rather than…enumeration of skills.” However, there is little discussion of similar framing for SR instruction. Through a combination of principle-based instruction pedagogies, hands-on activities, and self-explanation exercises, we aim to empower learners’ to make methodologically sound decisions during their reviews, rather than simply teaching the required steps or guidelines. In particular, our four-day, activity-based workshop was born out of the need to provide educational opportunities for students, faculty, and staff with a variety of experiences with ES methods and from many disciplinary backgrounds. We’ve revised this workshop, over four iterations, with feedback collected through daily forms and an overall survey. Presentation attendees will be able to consider ways to apply a principle-based approach for teaching the SR methods at their own institutions.

Program Conclusions:
Since its launch in 2021, we have collected a great deal of feedback from participants. Across all iterations, the workshop is rated as exceeding or meeting expectations by the majority of participants. On average, participants self-reported confidence in applying SR methods increased by approximately two points on a five-point likert scale. We plan to begin IRB approved data collection in January 2024 in an effort to more formally evaluate effectiveness of this approach. Through formal data collection, we will begin testing basic competencies regarding participants’ understanding of the principles of the systematic review method and continue collecting self-reported confidence. For this presentation and any resultant manuscripts we will also include a review of existing pedagogical approaches and literature related to our approach.

Utilization and Uptake of the UpToDate Clinical Decision Support Tool in Selected Medical Schools in Uganda (August 2022-August 2023): a partnership w

Track(s): Clinical Support

Alison Kinengyere, Associate Library Professor, Makerere University-Albert Cook Medical Library

Glorias Asiimwe, Busitema University

Adrine Nyamwiza, Medical Librarian, Makerere University-Albert Cook Library

Arthur Karemani, Gulu University

Julie Rosenberg, Ariadne Labs

Nelson Twinamasiko, Mbarara University of Science and Technology

Wilson Adriko, Mbarara University of Science and Technology

Background:
The use of point-of-care, evidence-based tools in clinical practice has become increasingly popular in many parts of the world. Evidence-based tools can provide easy-to-use, high-quality, regularly updated information and have been shown to improve clinical outcomes. Integrating such tools into clinical practice is an important component of improving the quality of health care. However, such tools are rarely used in resource-limited settings like Uganda. Hence, there is limited research on uptake, especially among medical students.

This paper explores the utilization and uptake of the UpToDate tool that was provided free of cost at selected medical schools in Uganda.

Description:
Through Better Evidence at Ariadne Labs, UpToDate granted free access to five medical schools in Uganda. On-site librarians facilitated training sessions and spread awareness of the tool. Usage data over the course of one year was aggregated, based on log ins and content views, and presented and analyzed using Excel tables, charts, and graphs.

Program Conclusions:
Because UpToDate is a clinical resource, the outcome of the program is to provide credible, evidence-based, up-to-date and reliable information that can be used by clinicians at the point of care of patients. This is being measured using the usage data as well as survey responses from healthcare providers and researchers who have used UpToDate at the point of care and research.

The sample data analyzed shows evidence of meaningful usage during the period August 2022 - August 2023. However, usage varied by user categories, showing some inconsistent uptake and low usage.

Librarians can draw upon these results to encourage stakeholders to support uptake of point-of-care tools in clinical practice.

LIGHTNING TALK PRESENTATIONS: RESEARCH ABSTRACTS

We have sorted lightning talk presentation research abstracts in this section by title in alphabetical order.

The Effectiveness of Library Space on Student Learning and Success: Perceptions and Realities

Track(s): Education, Professionalism & Leadership

Samantha Loster, Electronic Resource Management Librarian, Rosalind Franklin University of Medicine and Science

Objectives:
We are a health sciences library serving graduate students across 6 colleges focused on the health professions. Our university has embarked on a massive space planning endeavor to make better use of our spaces. The last big change to our library space occurred in 2015, when our open stacks were converted to student study areas. Despite renovations being years out, we started our own planning to better assist the university in making the best choices for our students. To do this, we wanted to better understand the perceived effect of library space on student learning and success.

Methods:
Our library has 3 main spaces; the learning commons where speech is allowed and the quiet study area and lower level which are low speech or silent study areas. We focused on five main components within learning commons; collaborative study rooms, group collaboration tables, small study rooms, individual study spaces, and individual computer stations. The five main components from the quiet study area and lower level that we focused on were open study tables, group study rooms, individual study carrels, individual study cubicles, and the silent study room. Student perceptions were amassed in three separate ways. The first and second were quantitatively collected via an anonymous survey left up in the library for the two weeks before winter break. The third was collected qualitatively via two, 1-hour focus group interviews. The anonymous survey and the focus groups included images of each component to ensure everyone had the same vocabulary for our inquiries. We then compared these to a fourth dataset, a library space count, collating data on what parts of the library are in most use during specific parts of the day, week and year. This data has been amassing continually since 2021 and sporadically as far back as 2013.

Results:
Space count data shows that our quiet section is the most occupied area of the library. The anonymous surveys showed that our individual study cubicles were ranked the highest. More areas of the learning commons were ranked higher in terms of benefitting student success than areas of the quiet section. Our computer area consistently ranked as least beneficial. Focus groups revealed one student found the computer areas useful for the CD drive. Students also complained that the lower level lacked natural light and the collaborative study rooms were inaccessible due to personal items being used to “hold” rooms.

Conclusions:
While the other complaints regarding lights and “dibs” have been known issues, the dislike of the computer area is a new concern. We have removed some of the computers, converting some to docking stations for student laptops and others to open study space. We are acquiring disc drives for students to check out. We plan to create room calendars and rules for the collaborative study rooms that prioritize group study and equitable access. We are exploring ways to increase natural light to areas of the lower level as well as how to best reconfigure parts of the lower level and quiet sections. We hope that the changes we are able to make will increase student learning and, ultimately, student success.

Entrustable Professional Activities (EPAs) Assessment in US Allopathic and Osteopathic Undergraduate Medical Education: Initial results from a scoping

Track(s): Education

Katie Hoskins, Health Sciences Librarian, Assistant Professor, Touro University Nevada

Heidi Reis, Liaison Librarian to the Brody School of Medicine, East Carolina University

Laura Zeigen, Health Sci Ed/Research Lib, OHSU

Margaret Hoogland, Clinical Medical Librarian, Mulford Library, University of Toledo

Objectives:
The Association of American Medical Colleges announced 13 Entrustable Professional Activities for Entering Residency (EPAs) in 2014, which osteopathic and allopathic medical schools adopted. Linking information literacy to EPAs and determining where librarians can assist with EPA knowledge acquisition and competency provides a pathway for librarian integration with curriculum. This review sought to understand and map assessment methods for EPAs in medical education by answering four questions.

1. Which EPAs do US medical schools assess?
2. To what extent do they assess EPA 7?
3. How do clerkships assess different EPAs?
4. Do allopathic and osteopathic schools assess EPAs differently?

Methods:
Researchers developed a search strategy in PubMed (Medline) to find all articles examining EPA assessment in undergraduate medical education, which two colleagues peer reviewed using a modified PRESS (Citation?). Then, researchers translated the search strategy into other databases and run from 2014 until January 2023 in PubMed (https://pubmed.ncbi.nlm.nih.gov/), Scopus (Elsevier), Web of Science (Clarivate), ERIC (EBSCO), Embase (Elsevier), MedEdPortal and Google Scholar. The results were uploaded into Covidence and automatically deduplicated, resulting in 1404 articles to screen. Articles were double screened against the inclusion criteria in two stages, first the title/abstract and then full text by the 4 authors. Inclusion criteria were: US based osteopathic/allopathic medicals schools, reported on the EPA(s) assessed and how they were assessed, and shared results of the assessment. In Covidence, researchers developed a data extraction form. The data extraction form gathered citation details, school type (allopathic/osteopathic), phase in MD program (preclinical/clinical), specific EPA assessed and how it was assessed (e.g. pre/post assessment, workplace-based assessment, OSCE/Standard patient, direct observation, simulation etc). Researchers piloted the data extraction form with 12 articles. After pilot test results, researchers adjusted aspects of the form for clarity.

Results:
Of 3031 retrieved citations, 1404 results remained after de-duplication. Following title and abstract screening, 369 abstracts moved to full-text screening. Currently, ongoing data abstraction of the 108 included full-text articles and conference proceedings indicate that medical schools most frequently assess EPA 10 and EPA 1. Although EPA 7 is of greatest interest to librarians, it is not among the top five assessed EPAs extracted thus far. The predominant EPA assessment method overall, at this stage of data abstraction, is medical student self-assessment (64% of currently abstracted articles). These results could change following completion of data abstraction.

Conclusions:
At this point in the study, US medical schools most frequently assess EPA 10 and EPA 1. The assessment of EPA 7, which could provide the greatest opportunity for librarian involvement, appears infrequently in the published literature. This could present an opportunity for inclusion of librarians in assessment of EPAs beyond EPA 7. Assessment of EPAs by clerkship varies. It is challenging to ascertain if allopathic and osteopathic schools assess EPAs differently, as few osteopathic schools have published research on this topic. A difference could exist between what takes place in practice versus what is published around assessment of EPAs, and librarian involvement in EPA assessment, which might present opportunities for further research on this topic.

An Evaluation of Connecting Library and Information Science Graduate Students with Consumer Health Information Specializations (CHIS)

Track(s): Education, Information Services

Samantha Nunn, Program Manager, NNLM Training Office

Objectives:
Through a unique partnership with a national association, a process was developed to connect professors teaching consumer health-related courses at LIS graduate programs to get their courses approved to meet the core competencies required for a CHIS certificate. After a few years of launching the program, the author conducted an evaluation to determine if the program is empowering students to get their CHIS certification while attending their LIS program. To determine the program’s impact, the author is gathering information on the number of students who received their certification and receive feedback from professors and students participating in the program.

Methods:
Quantitative data was collected to compare the number of students who received their CHIS certificate to the total number of students who completed the CHIS-certified course. The number of CHIS recipients was retrieved through the partner association that grants the CHIS certifications to recipients, and the total number of students who completed the course was collected through the participating LIS programs. Qualitative data was also collected by emailing instructors teaching a CHIS-certified course to request feedback on ways the program can be improved. Some of the questions the author asked were if the application process to get their course CHIS-certified went smoothly, if the procedure for offering CHIS certificates worked well, if any students experienced any challenges applying for their certificate that they’re aware of, and more. Additionally, interviews were conducted with a sample of LIS students who have received their CHIS certificate to assess if the process for applying for their certification was easy or challenging and if the certification was useful for their career development and to share examples, if any. This data is critical in evaluating if students are interested in receiving a CHIS certificate and identifying challenges professors and students may be experiencing in the program.

Results:
The preliminary data illustrated that more than half of the students who completed the course applied to receive their CHIS certification. Professors who provided feedback thus far expressed the application process was straightforward and appreciated the support from the organization.   Furthermore, professors described the certification as a nice way to introduce students to the health sciences librarianship field. Additionally, students appreciate the opportunity to add this valuable certification to their resume or curriculum vitae. More data is being gathered at this time and will be shared at the conference.

Conclusions:
The program continues to provide students with an opportunity to be recognized as having a specialized skill set in providing accurate and fact-based consumer health information on top of the course credits they earn through their LIS program. Currently, twelve classes have been approved to offer CHIS certificates from eight universities nationwide. The preliminary data has given insight into students' interest in obtaining a CHIS certification. The final results will guide the author in taking the next steps to improve the procedures involved in the program and provide helpful resources for instructors to use to help share the benefits of earning a CHIS certification with their students.

Experiences of Librarians Working with Physician Associate (Assistant) Programs: Preliminary Interview Results

Track(s): Education, Information Services

Karina Kletscher, Health Sciences Librarian, Creighton University

Megan Jaskowiak, Health and Social Sciences LIbrarian, Miami University

Michelle Nielsen Ott, Assistant Professor, Science/Health Science Librarian, Bradley University

Objectives:
According to the United States Bureau of Statistics, there will be approximately 12,000 openings for physician associates each year for the next decade (https://www.bls.gov/ooh/healthcare/physician-assistants.htm). To meet this demand, colleges and universities have been adding to the number of accredited programs. The three authors work for such institutions and when preparing for their programs noticed a lack of research on how this population uses the library and interacts with librarians. The purpose of this mixed methods exploratory study is to examine the practices and perceptions of librarians/information specialists who are or will actively support PA programs.

Methods:
This study is now in the second phase. In the first phase, a survey was sent in early 2022 to multiple listservs and librarians/information specialists listed as subject liaisons for the PA program. We collected 71 completed surveys using Qualtrics and performed quantitative analyses. Sixty-eight of these participants were willing to participate in semi-structured interviews. Zoom was used to conduct 15 interviews. Corrected transcripts are being coded and thematically analyzed with Dedoose.

Results:
Upon acceptance of this abstract for a lightning talk, this section will be updated with our preliminary interview results.

Conclusions:
This study’s results will expand the knowledge of what resources and services PA programs utilize and how libraries can best collaborate with them. These findings will assist both librarians/information specialists already working with PA programs and those at institutions developing new PA programs.

Health Professions Students’ Use of Generative AI Applications

Track(s): Education

Edwin Sperr, Clinical Information Librarian, AU/UGA Medical Partnership

Emily Harris, Dental Medicine & Cancer Librarian, Augusta University

Objectives:
While there has been much discussion about how (or whether) health sciences librarians should support student use of generative AI technologies such as ChatGPT, little is currently known about the actual extent of student use of such technologies. This study is designed to survey students about how, why and how often they are using these applications.

Methods:
The investigators have created an anonymous survey that will be distributed to medical and other health professions students at an academic institution in the United States. This survey is designed to elicit information about which individual generative AI applications they are using and for what purposes. Basic demographic information will be collected for each respondent as well as which course of study they are pursuing.

We are currently in the process of obtaining approval for this project from our local Institutional Review Board. The survey has already been pre-approved for distribution in March 2024 by the campus office of institutional effectiveness.

Results:
We are looking forward to beginning the process of gathering and analyzing data in March.

Conclusions:
We anticipate that the results of this project will help librarians, teaching faculty and administrators make better decisions about how to approach these technologies in health professions education going forward.

Historical Cadaver Ledgers as Valuable Chronicles of Cadaver Use in Early Medical Education

Track(s): Education

Heather Harrison, Special Collections Coordinator, Kansas City University

Lori Fitterling, University Library Director, Kansas City University

Objectives:
Our objective was to first document and research these archival records and then apply library preservation policies to ensure the future of these historical artifacts.

Methods:
We began by extracting detailed data from the ledgers, digitizing content, and cataloging the documents for our historical archives collection. Library preservation policies were followed. Librarians performed a robust literature search in PubMed, Google Scholar, and other relevant databases. The search retrieved information on the historical use of cadavers in medical education. We also researched specific funeral homes and mortuaries that were cited in the ledgers and contacted the State Historical Society about the cadaver records. We also contacted older osteopathic university libraries or archives inquiring if their historical archives included cadaver-receiving records. Finally, we documented our processes and results.

Results:
The cadaver ledgers provided a chronological record of the need for a human body dissection experience in medical education and the importance of hands-on anatomy education for the years 1911-1994. From our literature search, we focused on the ethics of how cadavers were obtained and used in the past, the legal and regulatory frameworks surrounding the use of cadavers, and how this has contributed to current body donation programs.

Conclusions:
Preserving and studying cadaver records is valuable in understanding the history of medical education and its implications for present-day practices. Highlighting cadaver characteristics helps to acknowledge the contributions and experiences of those whose bodies were used for the training of osteopathic physicians.

By looking back at historical practices, we can inform ongoing discussions about the best ways to teach anatomy and medicine. Overall, this project not only contributes to the preservation of historical records but also provides insights into the development of anatomy education and the contributions of those who donated their bodies for the advancement of medical knowledge.

Inclusive Information Literacy Education Enhances Digital Freedom in Black Diasporic Communities

Track(s): Education, Health Equity & Global Health, Information Services

Bethany Mcgowan, Associate Professor, Libraries and School of Information Studies, Purdue University

Objectives:
In this study, the critical nexus between digital freedom and proficiency in navigating online health information is explored within Black diasporic communities. Barriers to accessing reliable health resources in these communities often result in the spread of misinformation, compounding existing health disparities. This study advocates for the implementation of inclusive health literacy education, tailored to the specific needs of Black Americans, to address this issue and foster transformative change.

The research seeks answer: How does inclusive health literacy education impact the cultivation of digital freedom and proficiency in navigating online health information within Black diasporic communities?

Methods:
The research design employs a qualitative approach, utilizing interviews with 15 librarians and information scholars across academic, public, and government library settings from the U.S., Canada, and Finland. Transcribed interview content was thematically analyzed using NVivo.

Results:
Three key themes were revealed. Firstly, inclusive health literacy education cultivates empowerment and autonomy. Secondly, beyond digital skills, inclusive health literacy education highlights the importance of cultural competence. Lastly, the ripple effect of inclusive health literacy education extends to community-driven initiatives.

Conclusions:
In conclusion, this research underscores the significance of inclusive health literacy education in enhancing digital freedom and health information-seeking skills within Black diasporic communities. The thematic analysis reveals the multifaceted benefits of such education, portraying it as a pivotal instrument in dismantling health disparities and contributing to the cultivation of a healthier, more informed society.

Inclusivity Initiatives for Health and Medical Library Workers: Recommendations for Practice based on a Scoping Review

Track(s): Professionalism & Leadership

Amy Taylor, Medical Librarian, Houston Methodist Hospital

Xan Goodman, Health Sciences Librarian/Associate Professor, University of Nevada, Las Vegas

Jane Morgan-Daniel, University of Florida, University of Florida

Chloe Hough, Reference and Instruction Librarian, Nova Southeastern University

Objectives:
Four librarians conducted a scoping review on diversity, equity, and inclusion (DEI) initiatives for workers in health and medical libraries. The review’s objective was to map out how these DEI initiatives vary in terms of 1) how DEI is defined; 2) their focus, such as policies, programming, recruitment/retention, services, or space; 3) their stated impact; 4) how libraries advocate for involvement in DEI and the associated challenges they face; and 5) recommendations for practice or research gaps. The review encompasses the years 2014 onwards due to the globally emerging Black Lives Matter movement, which catalyzed awareness around DEI.

Methods:
A protocol was registered in the Open Science Framework and the literature search took place in August 2023. Eight databases were searched for a total of 8,911 results. De-duplication and screening took place in Covidence. After pilot screening, four reviewers independently carried out title and abstract screening of the 6,899 results, with two reviewers per article. Full-text screening used the same process and screening disagreements were resolved through discussion. Twenty-four articles progressed to the data extraction stage, where the pre-populated data extraction forms were filled out by two team members independently. The data extraction results were analyzed thematically and summarized narratively.

Results:
The results encompass a map of types of DEI initiatives conducted for workers in health science libraries over the past seven years in different countries, as well as levels of impact, the challenges and successes involved, and strategies for justifying and advocating for library involvement in DEI.

Conclusions:
Evidence-based recommendations for other libraries considering inclusivity initiatives include: acknowledging emotional labor, establishing DEI committees, implementing ongoing training, and increasing awareness of structural barriers. Research gaps identified were strategies for training, recruitment, mentoring, and evaluation methods.

Liaison Diaries: Two First-Year Health Science Librarians Reflect

Track(s): Professionalism & Leadership

Charlotte Vandervoort, Research and Education Librarian, UNT Health Science Center

Melissa Brand, Research and Education Librarian, UNT Health Science Center

Objectives:
Seldom do two librarians begin their journey at a library simultaneously, providing a rare opportunity to compare and contrast the first-year experiences of health science liaison librarianship. This comparative exploration is presented through the dual perspectives of a librarian new to the profession and another transitioning from general librarianship to the health sciences. By sharing insights into their challenges, successes, and adaptation strategies, the objective is to offer a comprehensive view of the unique journey undertaken by these librarians.

Methods:
Embarking on the exploration of our first-year experiences in health science liaison librarianship, we chose a qualitative research approach, leveraging in-depth interviews and reflective narratives to delve into the nuances of our journeys. As the two subjects, we represent distinct perspectives—one of us being a newcomer to the library profession, while the other brings prior experience in general librarianship but is new to the health sciences.Throughout our inaugural year, we engaged in periodic interviews that provided an evolving snapshot of our roles, challenges, and adaptive strategies. These conversations allowed us to openly share our insights, reflections, and the evolving dynamics of our immersion into the realm of health science liaison librarianship.Our data analysis followed a thematic approach, identifying recurrent themes, shared challenges, and individual successes from the interviews. This comparative analysis aimed to spotlight commonalities and distinctions, and offered a nuanced portrayal of health science liaison librarianship through the lenses of a novice and an experienced librarian in transition.

Results:
The initial journey of the health science librarian illuminated the inherent challenges and complexities involved in this specialized role. Navigating the intricate landscapes of medical, research, and academic units presented a unique learning curve, demanding a deep understanding of medical terminology, familiarity with health databases, and the ability to engage with healthcare professionals and researchers effectively. Our experience underscored the importance of not only possessing specialized knowledge but also cultivating meaningful connections within these specialized areas.

Conclusions:
Our parallel journeys highlight the pivotal role of specialized knowledge in navigating the intricacies of this field. Through these shared experiences and insights, the aim is to inspire a collaborative dialogue among professionals, fostering a deeper understanding of the multifaceted nature of liaison librarianship in the ever-evolving landscape of health sciences.

Our Stories, Our Queries, Our Health: Health Information Seeking Behavior in the Context of Blackness

Track(s): Education, Health Equity & Global Health, Information Services

Dev Wilder, PhD Student, University of Illinois Urbana-Champaign

Objectives:
1. Analyzing how Black users engage with search engines to obtain health information

2. Discovering critical spaces Black users go to for their health information need specifically in a digital capacity

3. Black users assess the quality of health content that is available in these online spaces?

4. The support of medical and health libraries in health information seeking behaviors for Black users

5. Documenting individual stories in health information seeking behavior

6. Investigating how cultural contexts (ambiguity in Blackness) inform the health information search process

Methods:
The developing research plan is the following:

Setting:

1. United States

2. Areas with predominantly African American/Black/African Diasporic Individuals in US

Population:

1. African American/Black/African Diasporic Individuals in US

2. Participant ages 18<

3. Access to Internet

Design:

1. Using interactive information retrieval (IIR) to understand technological engagement in health information search from Black users (Qualitative and Quantitative)

2. Conduct oral histories on accessing health related information over time and documenting current experiences in searching for health information (Qualitative)

3. Conduct focus groups to gather data on preferred sources, trusted/least trusted platforms (Qualitative and Quantitative)

4. Health information literacy assessment (Quantitative)

5. Participatory design to build a community search guide with resources for Black people (Qualitative)

6. Participatory design to create programming for health information literacy and support (Qualitative)

Results:
Ideal Outcomes:

1. Promote agency and collaboration to Black patients and health care professionals

2. Build a community search guide with resources for Black people

3. Create programming and information pipeline between medical/health libraries and general public

4. Explore and highlight the social, economic, cultural, and technological factors that impact the health information search process

Conclusions:
There is little research and investigation specifically around cultural factors impacting the health information search process and that acknowledge the social, economic, and technological factors unique to the Black community. In relation to medical library and information science professionals, there can be more programming that goes beyond patient education and geared towards general education as well as increasing health information literacy. Current studies tend to focus on the likelihood of searching or search by individuals with preexisting conditions. In addition to that, more research continues to be conducted on health information seeking behaviors compared across races but limited exploration explicitly observing a community at large and understanding intercommunal nuances that occur.

Potential Pitfalls of Using Abbreviations in PubMed Searches

Track(s): Information Services

Edwin Sperr, Clinical Information Librarian, AU/UGA Medical Partnership

Objectives:
Less experienced searchers often rely on automatic term mapping (ATM) to convert simple keyword searches in PubMed to more sophisticated strategies. Healthcare workers often use abbreviations when discussing or documenting patients and so may be tempted to use abbreviations when searching PubMed as well. This project is designed to ascertain how well ATM handles these types of abbreviations and the impact it has on retrieval for users.

Methods:
Lists of medical abbreviations previously identified by other investigators were obtained by the author. These abbreviations cover a broad range of acronyms, disease states and biological readouts, ranging from the common (‘CPR’ for ‘cardiopulmonary resuscitation’) to the relatively obscure (’msud’ for ‘maple syrup urine disease’). These abbreviations were searched programmatically in PubMed using the eutils API, and results from each search were checked to see if the abbreviation was mapped using automated term mapping. For those abbreviations that were mapped, hand-checks were performed to see whether those mappings were correct.

Results:
In one set of 2666 medical abbreviations derived from Wikipedia, only 35.11% (936) were automatically mapped in PubMed searches using ATM. Of those that were mapped, about half were incorrectly mapped to journal titles, with much of the rest of the abbreviations being assigned to authors or MeSH subheadings. Indeed, only 7.09% (189) abbreviations were correctly mapped to a common clinical meaning by ATM. Further investigations are being conducted to assess the full impact of these gaps on retrieval effectiveness.

Conclusions:
Very few medical abbreviations are correctly mapped by PubMed’s automated term mapping. PubMed users should be cautioned not to use abbreviations instead of full words in a simple search as they are likely to miss a large portion of the items that they are looking for.

A Scholarly Inquiry into the Implications of Terror Management Theory for Health Information Services

Track(s): Education

Dasha Maye, Technology and Communications Strategist & Asst. Professor, NNLM Region 2 MUSC

Objectives:
The overarching objective of the lightning talk is to offer perspective on the intricate correlations between social psychology, human information behavior, and potential health implications within terror management theory.

Methods:
This presentation centers on a meticulous analysis and curated annotated bibliography of the article "Potential Implications and Applications of Terror Management Theory for Library and Information Science."

The annotated bibliography not only situates itself within the broader academic discourse, elucidating social theoretical underpinnings, but also distills practical insights for librarians and health workers interested in information services and factors influencing health-related decision-making.

Results:
Aiming to transcend conventional disciplinary boundaries, the presentation delves into the nuanced interplay between terror management theory and health information services, and interprets how psychological phenomena manifest in human information behavior with direct implications for public health outcomes.

Conclusions:
The annotated bibliography not only situates itself within the broader academic discourse, elucidating social theoretical underpinnings, but also distills practical insights for librarians and health workers interested in information services and factors influencing health-related decision-making.This represents a unique opportunity for the field to engage in a dialogue transcending disciplinary silos. By presenting a synthesized exploration of terror management theory, information behavior, and potential health implications, the lightning talk seeks to stimulate intellectual discourse and foster collaborative initiatives bridging academia and healthcare. Join me on this journey of discovery, where minds converge, practices evolve, and interdisciplinary collaboration thrives.

Spurious Copyrights, Paywalls, and Public Domain Works by United States Government Officials

Track(s): Information Management, Information Services, Innovation & Research Practice

Kate Nyhan, Research and Education Librarian for Public Health, Cushing/Whitney Medical Library, Yale University

Objectives:
Documents created by "by an officer or employee of the United States Government as part of that person's official duties" are in the public domain and not subject to copyright. But in practice, journal articles written by United States Government employees are sometimes published behind paywalls, or made available to readers for free but encumbered by copyright statements that prevent reuse. In this observational pilot study, I will investigate publications by United States Government employees in health sciences journals -- publications which should be, but may not actually be, in the public domain.

Methods:
In this observational pilot study, I will use publication metadata about author affiliations, open access status, and copyright status from sources such as PubMed, Medline (Ovid), Lens, PubMed Central, and/or Open Alex. After identifying articles by employees of different United States Government agencies, I will characterize the articles' open access availability and their licensing status. I will account for the effect of the "embargoes" which in some cases delay public access/open access. I will explore the prevalence of not-public-domain status articles for multiple federal agencies, journals, and publishers.

Results:
I will identify positive or negative outliers -- agencies, journals, or publishers which are handling public domain articles much better or worse than their peers. I will also make recommendations about the relative value of different bibliographic data sources and analysis pipelines to colleagues who desire to monitor open access or licensing status of articles at scale. I will deposit on the Open Science Framework any datasets

generated during the project -- potentially including lists of putatively public domain articles by agency, journal, and publisher.

Conclusions:
Articles that are putatively, but not actually, in the public domain present an opportunity for authors, journals, and publishers to collaboratively increase access to information. This analysis will establish the scale of the problem -- and also the potential upside of interventions to improve the identification by publishers of works that should be in the public domain.

Standing Strong in Electronic Resources: Strengthen Medical Libraries through a Consortium

Track(s): Information Management

Folasade Kolawole, Lead City University

Modupe Oluwabiyi, Standing strong in Electronic resources: Strengthen Medical libraries through a Consortium, E. Latunde Odeku Medical Library, College of Medicine, UI, Nigeria

Objectives:
The study aims to examine the state of electronic resources in Medical libraries in Nigeria. The study also explores the concept of building a consortium among medical libraries to pool resources together, expertise, and bargaining power to be able to stand strong.

Methods:
Qualitative and quantitative research designs were adopted for the study. To collect data, a questionnaire was designed with questions like demographic characteristics, need for electronic resources, frequency of use, and performance of the medical library in satisfying the needs of the students, faculty members and research fellows. The questionnaire method and interview methods were adopted. The target population of this study is librarians from all the medical libraries across the nation. The questionnaire was an online survey because the libraries are all over the nation.

Results:
The findings from the study revealed that the majority of the libraries do not have subscription-based electronic resources due to financial challenges. The study gave recommendations that medical libraries should collaborate to form a consortium to tackle the challenges facing medical libraries in Nigeria. The framework for the development of the consortium too was discussed.

Conclusions:
Clinicians are always in need of quality information for their day-to-day activities but lack of quality electronic resources is a challenge. The mission of medical libraries is to enhance health and research through robust information resources. The way out to fulfill this mission in medical libraries is to come together to build a consortium. We are stronger standing together but we are weaker staying apart. Collective subscription to electronic resources will help to cope with the rising cost of information resources which will lead to resource sharing among member libraries. Resource sharing is very crucial to medical librarianship. Resource sharing will bridge the gap between stronger libraries and weaker libraries and eventually, we all become stronger.

Stronger Together: Comparing Overviews in DynaMed Plus, ClinicalKey, and UpToDate

Track(s): Clinical Support, Information Services

Dana Gerberi, Mayo Clinic

Ellen Rothbaum, Medical Librarian, St. Francis Hospital & Heart Center

Helen-Ann Brown Epstein, Informationist, Virtua Health

Kaitryn Campbell, Medical Librarian, St Joseph's Hospital

Objectives:
Today's clinicians often seek information on their mobile devices. With the use of an evaluation rubric, a team of medical librarians from different healthcare institutions will test popular point-of-care apps to explore how they measure up in answering real-life clinical questions.

Methods:
Apps from Clinical Key, DynaMed and UpToDate will each be rated based on ease of navigation, currency of the information, availability of graphics, timeliness of references, and other standard criteria. A set of wide-ranging sample questions, derived from interprofessional clinical rounding on medical-surgical units and intensive care units, will be used for testing. Overall impressions will be offered on the informational value of these tools in clinical settings.

Results:
This lightning talk will present a comparison of how the apps measure up for patient care.

Conclusions:
The team of librarians share a fair analysis of the value of apps from Clinical Key, DynaMed and UpToDate in addressing clinical questions and make recommendations for providers and other health information professionals.

Stronger Together: Librarian Utilization in Systematic Reviews And Meta-Analyses at a Multi-Site Academic Medical Center.

Track(s): Information Services

Dana Gerberi, Mayo Clinic

Diana Almader-Douglas, Librarian III, Mayo Clinic (Arizona)

Ellen Aaronson, Mayo Clinic

Heather Jett, Mayo Clinic

Tara Brigham, Supervising Librarian in Florida, Mayo Clinic

Objectives:
Systematic reviews and meta-analyses published by authors from a multi-site academic medical center published between 2018-2023 were quantified to determine librarian involvement. In 2020, a process analysis team found that requests for systematic reviews and meta-analyses were from inexperienced staff

and did not utilize librarians in their projects. In 2020, 500 systematic review and meta-analysis requests were received, many of which did not register protocols, had poorly defined clinical questions, and did not comprehend methodologies.

Methods:
Searches in our home-grown institutional authorship database, PubMed and Scopus were conducted to learn more about published systematic reviews and meta-analyses by authors in our organization. Keywords included “information professional*,” “librar*,” “systematic review*,” “meta-analys*.” Librarians conducting literature searches in the organization were also searched individually in each database. Publications were limited to 2018-2023. Over 10,000 citations were retrieved. After de-duplication in EndNote, there were about 5000 remaining citations.

Next, title and abstract reviews were conducted in Covidence to exclude papers by investigators outside our organization as first author. Exclusion criteria also included published manuscripts that were not systematic review or meta-analysis publication types. Around forty-nine percent met inclusion criteria for review for librarians as co-authors or given formal written acknowledgement in the remaining published papers. The remaining results were then categorized as having librarian involvement or no librarian involvement.

Results:
Preliminary findings of the data indicate that a substantial portion of systematic reviews and meta-analyses by authors in our organization are carried out without assistance from librarians as evidenced by librarian co-authorship or written acknowledgment in publications.

Conclusions:
Librarians in our organization receive systematic review and meta-analysis requests from students or short-term staff who have been given assignments to complete these projects without adequate support or education. Librarians also encounter staff who have poor understanding of a systematic review as a project versus the concept of “searching systematically.” Next steps include outreach to staff with research activity. Interventions include announcements on digital signage for employees, sessions to help with systematic reviews such as clinical question development, protocols, and associated software. Reaching out to departments is also feasible as departmental presentations during meetings are already taking place.

LIGHTNING TALK PRESENTATIONS: PROGRAM DESCRIPTION ABSTRACTS

We have sorted lightning talk presentation program description abstracts in this section by title in alphabetical order.

The Accidental Archivist: Interdepartmental Collaboration to Preserve Historical Records in a Community Hospital

Track(s): Information Management

Margaret Easbey, Librarian, Cottage Health

Background:
To describe, arrange, and make accessible a large collection of historical documents,

photographs/multimedia, and objects pertaining to the history of a community Hospital and to the history of medical practice.

Description:
Upon discovering the collection of roughly 100 containers (mostly cardboard file boxes), the Librarian (who has past experience in archival work) coordinated with the department in possession of the collection to implement a plan for its permanent preservation, organization, and housing. A review of literature pertaining to this unique scenario was conducted although few resources exist. The Librarian then contacted several local archivist colleagues and sought advice from MLA colleagues. Next, founding documents for the project were created, including a step-by-step plan, accession documents and inclusion criteria in the event that donors come forward in the future, and an initial inventory spreadsheet. The Librarian and Collaborator then began labeling containers, identifying material type, and making general notes as to contents of the containers especially noting anything fragile, dangerous, or noteworthy. With that step done, an arrangement plan is under way, and then the long phase of preservation and storage will begin. Ultimately our aim is to host a discoverable catalog online, and to digitize documents and photographs for ease of access and to reduce contact with the physical materials.

Conclusion:
Our priority is to preserve the rich history of this community Hospital. We hope that in doing so materials will be easier to access so that continued collaborations can occur between Hospital Administration and the Library to create exhibits and other publicity materials that highlight our collection. We also expect that this project will serve as a valuable opportunity for recognition of the Medical Library during a time when the value of libraries is increasingly called into question.

Adapting a Systematic Review Service for a Two-Person Reference Department

Track(s): Information Services

Elizabeth Huggins, Research and Education Librarian, Loyola University Chicago Health Sciences Library

Jonna Peterson, Director, Loyola University Chicago

Background:
Many health sciences libraries offering services supporting systematic reviews and evidence syntheses (ES) employ multiple librarians to cope with the exhaustive searches and time commitment. A small midwestern health sciences library joined its larger cousins by launching a formalized ES service in 2022, but with reduced staff. Only two librarians (the only reference librarian and the interim director) could provide needed services, so clear project expectations, time-saving techniques, and automated tools were essential to the success of the new program.

Description:
In July 2022, the interim director and research librarian formalized the library’s Systematic Review and Evidence Synthesis (SRES) service with a dedicated webpage and inclusion in library services (resource selection, exhaustive searches, documentation, screening tools, etc.). We implemented changes to the workflow to manage the projects: mandatory meetings with the costumer, compulsory protocols for all reviews, literature retrieval methods and timeline, and utilizing automated tools for full-text retrieval, PRISMA diagram, and screening/evaluation.

Conclusion:
Since the formalization of the service in July 2022, the number of systematic review and scoping review projects has increased tenfold. The approaches we implemented have allowed us to keep up with demand without sacrificing other services. The presentation will illustrate this change by comparing statistics from both the 2019 and 2023 AAHSL surveys.

Artificial Intelligence: Your Library Event Planning Assistant

Track(s): Information Services

Colleen Cuddy, Director, Research and Academic Collaboration, Stanford University, School of Medicine, Lane Libary

Boglarka Huddleston, Manager, Research and Instruction, Stanford School of Medicine

Samantha Willairat, Research Communications Librarian, Lane Library, Stanford University School of Medicine

Background:
There are multiple areas in academic medical libraries where AI is making an impact on service delivery and productivity. One area in particular is event planning. AI tools can benefit event planning from start to finish. A small academic medical library has used AI to help plan and run several successful events using generative AI tools to identify topics and speakers, develop program content, create marketing and event materials, and design evaluation tools. AI has become integral to running these events, making it a good but not a consistently outstanding assistant. A case study of using AI for library event planning will be presented.

Description:
We took an exploratory approach to using generative AI tools such as Rabbit, Elicit, and ChatGPT for library events and found several areas along the event planning timeline where AI was helpful. Identifying potential speakers and topics for events is one area where AI lends a hand. We also developed a workflow to create discussion and panel questions, create speaker bios, write speaker introductions, and develop the agenda and show flow for event pacing. AI has also been helpful in developing slides, marketing, and event materials and promoting our events on social media. We also learned valuable lessons about the accuracy of AI-generated content. Evaluation of the use of AI in event planning has been largely informal. It includes sharing tips, tricks, and best tools with each other, sharing prompts in a prompt library, and disseminating what we have learned through the Library’s AI Community of Practice. Overall, using various generative AI tools has resulted in several successful events and made the planning process more efficient.

Conclusion:
Using AI tools for library event planning and management has allowed us to optimize logistics and run engaging events. It freed up staff time spent on planning and enabled us to host additional events and use our creative selves at work. Learning how to use AI tools for events has translated to the use of AI tools in other areas of our work. In a distributed, team-based environment, the success of using AI in this context has encouraged staff in other library areas to use AI and to explore and employ generative AI tools more generally. Staff who have participated in event planning have confidence in using AI in their day-to-day work and have become regular AI users.

Assessing the Quality of Biomedical Boolean Search Strings Generated by Prompted and Unprompted Models

Track(s): Innovation & Research Practice

Robyn Reed, Biomedical Informatics and Emerging Technologies Librarian, Penn State College of Medicine

Derek Barnett, MD Candidate - MS2 Class of 2026, Penn State College of Medicine - University Park Program

Background:
The application of Large Language Models (LLMs) in medical librarianship, specifically in the development of Boolean search strings for literature review, is an emerging area of study. Despite known limitations in generating accurate biomedical citations, the potential of LLMs in formulating effective search strategies warrants investigation. This study aims to assess the efficacy of LLM-generated Boolean search strings in navigating the extensive medical literature, specifically within PubMed. The focus will be on evaluation of AI-generated search strings, providing a comparative analysis with expert-generated Boolean search queries. Such an assessment is essential for understanding the utility of LLMs in specialized academic and clinical research contexts, and may offer insights for future AI applications in literature searches.

Description:
Our initiative investigates the feasibility of using ChatGPT and a prompted GPT model to create Boolean search strings for literature searches. We explored the output of an unprompted ChatGPT to determine its inherent ability to generate Boolean search strings from keyword prompts. Additionally, with the recent release of OpenAI’s GPT models, we developed a prompted GPT model for producing Boolean search queries. This research also aims to assess the significance of prompts in generating optimal search strings. To evaluate the quality of the unprompted and prompted GPT models' search string outputs, we compared them with search queries published in academic papers. We extracted keywords and main ideas from the background sections of these papers and used them as inputs for the models. Our analysis includes both published and unpublished search strings, along with manipulation of the number of variables in the model for a comprehensive assessment. Furthermore, we compared the publications retrieved using the AI-generated search strings to those found using traditional search methods.

Conclusion:
This study is ongoing. Preliminary results indicate the prompted model is able to reliably produce Boolean search queries that are comparable to simple strings. More advanced strings are yet to be tested. Additionally, non-prompted search queries are yet to be examined. Results and limitations will be shared in this lightning talk, highlighting the key outcomes.

Bridging the Gap Between the Spanish-Speaking Roanoke Community and Medical Libraries

Track(s): Professionalism & Leadership

Roberto Silva, Head, Fralin Biomedical Research Institute Health Sciences and Technology Library, Virginia Tech

Background:
Medical libraries are not usually the first place for information gathering that Latinos, or Spanish-speaking communities, think about when dealing with medicine or healthcare in general. What started as a mutual collaboration during COVID-19 in late 2020, between an academic institution and a non-profit organization in Roanoke, has now transformed into two book clubs in Spanish for local members of the community that give them access to medical libraries and research facilities. Our objective is to bridge the gap between academic institutions and the public, in a medical setting, with the end goal of eventually offering health literacy services in Spanish to those that do not have access to it.

Description:
Our relationship with Casa Latina, a non-profit organization in Roanoke that addresses the needs of the local Spanish-speaking community in the Roanoke Valley of Virginia, started back in 2020 as part of the Latino Oral History Project: Voces of a Pandemic Project at Special Collections and University Archives at Virginia Tech. This project was in partnership with the Voces Oral History Center at the University of Texas at Austin. It ended in 2021 with a total of 16 interviews in both English and Spanish. Ever since, we have been in constant communication about finding ways to make our academic resources available to the public and the people they serve. Since I had been running a book club in Spanish since 2021, we decided to give the book club a try, given that we had several titles and copies of books that had already been used. In June 2023, we launched the first book club in Spanish in the city of Roanoke sponsored by an academic institution and a non-profit organization. Evidence that demonstrates the success we are having is that a second book club was created three months later, and they have been part of our academic events.

Conclusion:
We have taken a long-term approach in intentionally developing this relationship with Casa Latina. It has been three years in the making and involves a community group that has grown over time. The whole point is to have mutual benefit by having mutual interests; and the book clubs are the perfect mutual interest to bring us together. Opening the doors of our medical library and academic institution to the public, not only benefits the Spanish speaking community of Roanoke but it also benefits us in academia. It brings cultural competency for health professionals. Our hope is to develop health literacy programs for Casa Latina in the next two years that can be used across the city.

Building an International Partnership Between Academic Health Sciences Librarians: From Informal Exchange to Research Collaboration

Track(s): Health Equity & Global Health

Alice Stokes, Clinical and Education Librarian, University of Vermont

Maria Sobrido-Prieto, Contract Doctoral Professor, Universidad de A Coruña, Facultad de Ciencias de la Salud

Background:
Health sciences librarians around the world share many common goals and challenges. While national cultural environments, healthcare systems, and educational systems create conditions unique to each country, our shared professional values and mission connect us. Learning about medical librarianship in other countries helps:

a) understand barriers and solutions in different environments;

b) share resources and knowledge;

c) and build a network of experts worldwide, one element of MLA’s mission (https://www.mlanet.org/page/vision).

During an institution-supported sabbatical, an American health sciences librarian had the opportunity to pursue research on medical libraries and medical librarianship in Spain. This included a three-month research period in the country.

Description:
During her stay, the American librarian established networks with a representative from the regional virtual medical library. As a result of this connection, she was able to learn about the functioning of the virtual library (structure, organization, operations and staffing). This relationship created opportunities for further networking, including connecting with a librarian at the local academic health sciences library, which is responsible for the training of health sciences degree students. This resulted in an ongoing informal exchange.

During the exchange, the American librarian learned about the integration of the Spanish librarian into the health sciences curriculum and their teaching methodologies. The pair met in-person periodically over a three-month period and identified research topics of mutual interest. In the year following the visit, the pair continued to collaborate via email and remote meetings.

As a result of this partnership, the team collaborated on a study about the educational activities of hospital libraries in Spain. They conducted a survey of hospital libraries in Spain, analyzed the data and presented the results at the National Congress of Information and Documentation in Health Sciences (October 2023).

Conclusion:
The outcomes of this project to date include the completion of research on several topics (history of libraries in Spain, authorship of nursing publications, educational activities of hospital libraries in Spain.) Future plans include a visit by the Spanish librarian to the American librarian's home institution. The team is preparing several articles for submission to relevant journals.

The team has set a goal to increase current literature on Spanish medical libraries published in English, which is scarce. The dyad is involved in ongoing joint research projects along with other colleagues in Spain.

The presenters will offer suggestions and strategies for others interested in pursuing international partnerships. The experience has been enriching and professionally revitalizing for both parties.

Calculating Cost per Use Statistics for ClinicalKey Packages

Track(s): Information Management, Innovation & Research Practice

Liz Kellermeyer, Library Director, National Jewish Health

Background:
Objective: Customize a report that will calculate cost per use for ClinicalKey packages.

ClinicalKey (Elsevier) is a product that provides access to ebooks and journals, purchasable through subscription packages. Libraries have the option of subscribing to all content or to individual packages that contain journal and ebook titles bundled by specialty. While it is possible to pull usage statistics for individual journals, the vendor does not supply a way for librarians to pull usage statistics by package, even though that is how the content is sold. This talk describes a method using Microsoft Excel to calculate cost per use for these packages.

Description:
Methods: ClinicalKey supplies access to an updated master content list on their website, which lists details about all current journal and ebook titles in their subscriptions, including which package each title falls within. Via institutional login, account administrators are able to download on-demand standard, master and custom usage reports as per COUNTER Code of Practice Release 5. To calculate package cost per use, librarians can merge key parts of these two documents, then use INDEX and MATCH functions to attach the appropriate package to each title. It is then possible to sort by package, tally usage numbers, and calculate total usage per package. From there, standard cost per use calculations can be used.

Conclusion:
This report enabled our library to halve our CK package subscriptions, freeing up funds for other resources. Understanding individual package usage is crucial for gauging return on investment. Having these statistics allows librarian administrators to better understand how their resources are being used and whether budgetary funds are being properly allocated. Some examples of information this report can provide that is otherwise undiscoverable include: underuse of specific packages, packages that are offering the best value, and relative spread of journal use within a package (e.g. if a majority of the package use can be attributed to a single journal). Such information aids in strategic subscription decisions and precision cuts, maximizing budge efficiency.

Case-Based Learning Enhances Engagement, Critical Thinking and Motivation

Track(s): Education

Anna Liss Jacobsen, Health Sciences Librarian, Indiana University Indianapolis

Background:
Health science students need information literacy skills to competently complete class, research, clinical and other work. Students in a large university's undergraduate Nursing Research Methods and Evidence Base Practice (EBP) classes struggled with information literacy skills when one-shot library sessions were taught. Many were unable to find quality, relevant resources for their class work. Therefore, a library instruction session, incorporating Case-Based Learning (CBL), was developed and implemented to increase student motivation and engagement with material and create an environment for students to teach and learn from each other. Additional goals were to strengthen students critical thinking skills, consolidate information literacy skills and increase the likelihood that students apply skills learned for future information needs.

Description:
A library instruction/CBL session was recommended to nursing faculty teaching three EBP courses to 150 undergraduate students during the spring 2024 semester to strengthen students' information literacy skills. Faculty responded positively and approved it. The revised library session included a 30 minute library instruction session and 50 minute CBL. The library session was developed using Bloom's Taxonomy and backward design. Student learning outcomes included, students would be able to:

1. Identify PICO question keywords
2. Explain basic database search skills
3. Construct PubMed searches

4. Apply PubMed tools

5. Locate full-text and citations

A CBL was developed and finalized with faculty input. Finally, the library lesson was developed. The CBL required students to work as a team teaching a fictional student to find qualitative articles answering a PICO question using PubMed. The CBL included detailed instructions and hints. Additional questions invited students to self-evaluate their search processes. The librarian and faculty provided guidance to students as they completed the CBL.

One professor included the CBL as a graded assignment. The librarian reviewed the submissions and provided feedback to these students. Faculty provided informal feedback and students self-evaluated. A peer observer provided summative feedback of one instruction session.

Conclusion:
The CBL goal of enhancing student engagement with material, teaching and learning from each other and problem-solving to develop critical thinking skills was achieved. Overall, the students enjoyed themselves and were motivated and actively engaged in the CBL. Students taught and learned from each other, especially with librarian/faculty encouragement. Students further learned from their mistakes. Two EBP classes included students with less previous nursing and information literacy experience. One class was advanced; students had more previous nursing and information literacy experience. Outcomes differed between the more and less experienced classes. Themes students noted when self-evaluating their work were: importance of using skills gained to broaden and narrow search results to identify relevant resources, PubMed functions and more.

Collaborative Networks Among Different Schools to Enhance Access to Health Information in a Large-Scale University: Challenges and Opportunities

Track(s): Professionalism & Leadership

Alejandra Martinez del Prado, Collaborative networks among different schools to enhance access to health information in a large-scale university: challenges and opportunities., UNAM School of Medicine Library

David Flores Macias, UNAM School of Medicine Library

Background:
The COVID-19 pandemic forced libraries to rethink the ways of serving our final users, due to the confinement. Although the university already had a remote access service to digital collections, in 2020, there was not access to all the basic materials in electronic format needed by our first and second year students, and this became a priority challenge to address. It was identified that our Rector's Development Plan included the creation of working groups between related libraries to continue with the joint acquisition of electronic collections that benefit the university community, so the creation of the Health Libraries Group was proposed to meet this goal, which was approved and the group was created in March 2021 and it was made up of 7 schools related to the health science area.

Description:
There is a General Direction of Libraries (GDL), responsible to manage all the university libraries, and the medicine school library requested the GDL to coordinate the health library group, which was approved. Since it was created, direct communication was established with the members of the group to identify the

information needs, and it was discovered that there were many concerns and problems in common, therefore a global perspective work was started. A work plan has been established at the beginning of each year, in which the needs of each member library are considered, to determine the priorities of electronic resources and platforms on which to work, whether to be acquired or subscribed for the first time, or to rethink its renewal as a group of health libraries, avoiding isolated efforts. Constant communication is maintained with the GDL to support the group achieving better negotiations. Also, a collaborative work with publishers have been made, with the purpose to obtain better, more comprehensive resources that can serve all the communities of the health group libraries and not limit them to only a very specific area. It has been achieved that the entire university community can have access to the information purchased or subscribed, and is not limited to users of the group's libraries.

Conclusion:
Through these almost 3 years, the university's electronic collections have been enhanced, resulting in optimizing the budget, acquiring electronic resources of common interest in the area of health sciences, achieving better negotiations with suppliers, since with larger purchases, larger discounts have been obtained. It has been possible to subscribe two platforms as a group and two global subscription of ebooks from a publisher. The above has benefited mainly a community of approximately 60,000 end users of this group of health libraries. Communication between libraries has been enriched and the exchange of experiences on these topics has allowed libraries to adopt best practices in their communities. Other academic activities have been encouraged, such as greater participation in book fairs. There is more work to be done in the group.

A Collective Approach to Integrating Health Equity Considerations in Guideline Development

Track(s): Health Equity & Global Health

Carol Colasacco, Medical Librarian Specialist, College of American Pathologists

Marisol Hernandez, Medical Librarian Specialist, College of American Pathologists

Background:
As part of an organization committed to equity and inclusion, our team was inspired to take a closer look at our guideline development processes with the same intentionality. Clinical practice guidelines can promote health equity, but they may also unintentionally create or exacerbate existing health inequities. While guidance for the integration of health equity considerations into clinical practice guideline development is limited, we surmised that a review of available procedures and an investigation into what other guideline development groups have done to address health inequities could inform our evaluation. We examined our guideline development work breakdown structure in detail to develop a focused strategy to incorporate health equity considerations into our processes.

Description:
We reviewed published literature and guideline development resources (e.g., PRISMA-Equity, STROBE-Equity, GRADE, WHO-INTEGRATE framework) to better understand the complexity of health equity considerations in clinical practice guideline development. We then performed a detailed review of the individual steps of our work breakdown structure and brainstormed ways that health equity discussions could be integrated throughout the entire process: from the time a guideline topic is selected all the way to when guideline impact is evaluated. We presented our findings and recommendations to our guideline oversight committee. Upon consensus, we integrated health equity considerations into three specific processes and revised our standing operating procedures to reflect these changes. We will evaluate our process improvements by presenting specific health equity factors for individual projects during tri-annual

oversight committee meetings, reviewing standard operating procedures annually, and polling guideline panel members at predetermined intervals. Because traditional evidence-based guidelines may take several years to produce, changes to facilitate health equity considerations will take time to fully implement and evaluate.

Conclusion:
In an effort to drive heath equity, our team researched published literature, examined current processes, identified areas of improvement, revised procedures, and developed a plan to evaluate the changes. With input and feedback from guideline staff, guideline panel members, our guideline oversight committee and by surveying the literature, we were able to develop a collaborative approach to integrating heath equity into our clinical guideline practices.

"Connecting in Context: Situating Online Library Instruction within the Research Lifecycle"

Track(s): Education

Talicia Tarver, Research and Education Librarian, Virginia Commonwealth University

Hope Kelly, Online Learning Librarian, Virginia Commonwealth University

Background:
The health sciences library’s instruction is integrated across multiple health sciences schools within a major research institution. While online learning objects were routinely created and managed by department work groups, the temporary shift to strictly online learning left a lasting impact on how the department thinks about the sustainability and usability of online library instruction. The collection of objects can now be examined comprehensively, organized, and gaps identified.

Description:
This lightning talk summarizes an independent project whereby a health sciences library’s research and education department contextualizes its online instruction within the research lifecycle (as defined by Harvard University: Research Support at Harvard). The goal of this project is to compile and curate the current online learning objects as an index of open educational resources available to health sciences and library faculty. The following steps will be taken to create this index: Update the record of current objects and categorize them based on the topic covered and which research lifecycle heading it most addresses; Consult with an open educational resources librarian to locate an existing platform on which to host this index to maximize access; Promote the index as a potential part of an open educational repository.

Conclusion:
The project is still under development, but objects will be selected based on criteria including date of creation, frequency of use, and relevance to research methods courses. Because health sciences faculty request librarian-created objects for individual courses on research methods, the hope is that this index will streamline the process, including tracking the objects’ usage based on number of views and points of access.

The Cura Corner: Embodying Whole Person Care in the Library

Track(s): Information Services

Karina Kletscher, Health Sciences Librarian, Creighton University

Background:
“Belonging” has resurged within university campus climate discussions. A sense of belonging can help students feel supported and valued, which can lead to better academic outcomes and a more positive climate. Health sciences education, especially, faces pressure to address belonging’s complexities as their learners face high levels of stress, depression, depersonalization, and yet demonstrate feelings of social or self-stigma in seeking help – before they even emerge into high-stress roles with high rates of burnout and significant mental health challenges. In response to these wicked problems, and the myriad requests from learners who lacked access to personal care and wellness resources on a small urban campus, the Health Sciences Library and Student Life co-created a new wellness initiative: the Cura Corner.

Description:
This talk describes the planning process, implementation, and management of a dedicated wellness corner in an academic library. Designed with the Jesuit value cura personalis, “care for the whole person” and the Eight Dimensions of Wellness in mind, the permanent resource includes health and wellness information from university units, local organizations, and authoritative agencies, as well as take-what-you-need items for study, personal care, or grounding. The corner also facilitates community information-sharing and uplifting messaging between learners. It has even inspired a popular extra-credit practicum assignment, now in its third year. Despite a physically small footprint, the Cura Corner was not without its challenges; these institutional, financial, and professional challenges will be outlined as well as how interdisciplinary approaches and collaborations – and moxie – solved them.

Conclusion:
This talk will demonstrate how health sciences libraries can creatively and meaningfully engage in interprofessional initiatives, furthering conversations of libraries’ roles in consumer health and campus climate. Not only has the Cura Corner surpassed our initial hopes moving into its third year, but the project has also strengthened the collaboration between the Library and our collaborators, as well as increased the visibility of the library as a health and wellness resource and refuge for our campus community. Highlights of our current works-in-progress include inclusion in the university’s Giving Day campaign, collaboration with visiting capstone students on similar initiatives, and forthcoming research on health sciences students’ approaches to holistic wellbeing.

The Data Lifecycle Isn’t for Me! Showing the Benefits of Interoperable RDM Training

Track(s): Education, Information Services

Julie Goldman, Research Data Services Librarian, Harvard Library

Background:
A considerable challenge for institutions is segmentation of groups, creating barriers to communication and support for data services between disparate communities. To combat this issue, communication and collaboration between data professionals in the library, research labs, and information technology departments is imperative to help reshape institutional data services. Our Research Data Management Working Group made significant strides towards overcoming siloed environments by integrating resources and training from multiple data support groups, with emphasis on broader community engagement. Our goal was to streamline the dissemination of information to researchers and staff.

Description:
Through a group partnership, we have provided a dedicated space for locating training and events and identifying how they each align with the research data lifecycle, to build knowledge capacity about how data related topics intertwine. The group consolidated multiple training calendars, previously maintained by separate groups, to provide one landing space where researchers can view training and events focused on the research data lifecycle. This interoperability provides researchers a clear view of what resources and support is available to them. To ensure training remains updated, trainers from across the institution meet on a quarterly basis to discuss trends, incentivizing one another to improve and adapt their training. Additionally, we formalized a ‘data manager’ role at the institution. By encouraging labs to identify a dedicated individual responsible for research data, we are setting labs up for success with data oversight and aligning with goals of funders like the NIH.

Conclusion:
As an example, this past summer, we brought various experts from across the university library and information technology departments together to offer targeted training on Data Visualization. The training was hugely successful, with high turnout and interest from the research community. We will also be monitoring session attendance before and after the collaborative and consolidation efforts to understand the impact of our efforts. Through our examples, attendees will gain the strategies and examples to apply in their own research communities.

Defining Our Scope: Eliminating “Service Creep” in Library Systematic Review Support Services

Track(s): Information Services

Jenessa McElfresh, Interim Associate Director, University of Tennessee Health Science Center

Background:
As patron demand increases for library systematic review services, so does the potential for the scope of these services to outstrip the ability of the library to offer expert support throughout the review process - a phenomenon referred to here as “service creep.”

This lightning talk outlines strategies used to identify and mitigate instances of service creep in the established systematic review support service at an academic health sciences center library. This includes an overview of the indicators of service creep, interventions employed to reduce or eliminate service creep, and considerations for other libraries as their systematic review services grow.

Description:
Increased patron demand coupled with limited librarian capacity led an academic health sciences library to critically examine their systematic review service to identify areas in which the library was supporting systematic reviews outside of the intended scope of the service. Service metrics and observation were used to identify instances of service creep in the library’s systematic review services in both policy and practice. Interventions utilized to reduce systematic review service creep include: changes to the library’s systematic review service policy, updates to procedures for requesting and fulfilling systematic review services, and communication enhancements to clarify the scope of service internally and externally.

The identification of service creep and subsequent implementation of interventions resulted in a redefined understanding of the library’s systematic review service for both librarians and patrons, including defining what is not within the scope of the library’s systematic review services. Because the depth and breadth of the library’s systematic review service is better articulated and understood, the library is able to take on

more systematic review projects, capitalize upon librarian expertise and comfort in search development, and reduce librarian stress and workload per review project.

Conclusion:
Service creep is endemic in the growth of library systematic review services as patron demands can lead to librarian involvement throughout all steps in a systematic review project. At one academic health sciences library, unofficially supporting researchers throughout the entire review process led to inconsistent and unsustainable systematic review service implementation. The identification of this service creep indicated a need to re-examine the scope of the library’s systematic review services. Modifications to the library’s systematic review service policy, service procedures, and communication methods helped eliminate service creep in the systematic review service, allowing the library to continue to offer high quality systematic review services in areas of librarian expertise.

Developing Partnerships for Outreach to Indigenous Communities

Track(s): Information Services

Kiara Comfort, Community Outreach and Health Systems Librarian, McGoogan Health Sciences Library- University of Nebraska Medical Center

Background:
This presentation will focus on how an academic health sciences Community Outreach Librarian partnered with its College of Public Health’s Director of American Indian Health to promote health education to Indigenous communities in [state]. Libraries, with appropriate support, can develop partnerships with Indigenous communities to promote health education. There are four unique tribal communities in [state]. One mission of the university is to support tribal communities to promote healthy outcomes. With the appointment of the College of Public Health’s new Director of American Indian Health, the library sought to connect the Community Outreach Librarian to forthcoming work with Indigenous communities and with students on campus to promote Indigenous health education.

Description:
Part of [university name]’s mission is to support health in tribal communities by promoting of health education initiatives. The library supports this effort by exhibiting at tribal health fairs and supporting middle and high-school students and their teachers through educational initiatives such as the Science Education Partnership Award and the Youth Enjoy Science program. Engaging with Indigenous and tribal communities is possible only due to partnerships built overtime and with a commitment to trustworthiness. For the librarian, successful relationships require being aware of the different political and social structures that surround tribal communities and acknowledging one’s position as a non-member of communities. To implement programming for these communities the Director of American Indian Health met monthly with tribes to build trust and to understand their health needs. Once trust was established, the librarian was brought in to consult on health information needs.

Conclusion:
Through this partnership, the library has promoted health information via the tribe's health fairs, a community night focused on Indigenous health and wellness, and by supporting middle and high school students in their research endeavors. This partnership extends the library’s work to support the health of [state] and recruit pre-health sciences students. By maintaining these trusted partnerships, the library can continue to grow outreach within the Indigenous communities and support future health information needs.

Evaluating a Hospital Library’s Customized Search Alert Service

Track(s): Clinical Support, Information Services

Kathryn Vela, Medical Librarian, St. Luke's Health System

Background:
The St. Luke’s Library serves a distributed health system and supports the information needs of healthcare providers and administrators. In 2021, the library launched an expansion on the popular librarian-mediated literature search service that gave users the option to request that their search request be re-run each month and the newly published articles be sent to them in a curated list. The purpose of the customized monthly search alert service is to provide hospital staff with regular updates on the latest literature on topics of interest to support patient care and hospital policy.

Description:
The St. Luke's Library added a question to the existing literature search request form that gave users the option to request an ongoing monthly search alert for their topic. The initial search is completed through EBSCO databases that can include MEDLINE, CINAHL, PsycINFO, and/or Health Business Elite. The final search string from this initial search is saved to a search alert tracking sheet, along with user information like email, patron location, staffing group, and the health system goal served by the search. Each month, the searches saved in the tracking sheet are re-run, and the results are manually formatted to include the citation, abstract, and proxied link for direct access; these are then sent to the corresponding users. Users have the option to cancel or update their search alert at any time. In September 2023, a nine-question survey was distributed to 115 past and current users of the search alert service to evaluate its effectiveness and potential improvements. After a two-week period with one reminder email, the survey was closed, and the results were analyzed using descriptive statistics and thematic coding.

Conclusion:
Since the search alert service was implemented, 40% of search requests submitted via the library's request form ask for a monthly search alert. On average, 55-60 search alert results are distributed each month. The evaluation survey had a 42% response rate and 94% of respondents reported being somewhat or very satisfied with the service. Ninety-four percent of respondents also found that the search results were somewhat or very relevant. Almost a third of respondents reported accessing full-text articles via the library based on their search alert results. Based on respondent feedback, an option for quarterly search alert updates was added to the service, as well as a more user-friendly cancelation feature.

Evolution of a Virtual Medical Library Supporting a Top Pediatric Research Hospital

Track(s): Information Services, Innovation & Research Practice, Professionalism & Leadership

Kristen Burgess, Manager, Cincinnati Children's Hospital Medical Center

Karen Whyte, Librarian, Cincinnati Children's Hospital and Medical Center

Background:
This presentation explores the journey of a small research-focused medical library that has transitioned from a traditional in-person facility with a physical collection to a primarily digital existence. The library serves as a vital resource for a top ranked pediatric research hospital, adapting to the evolving landscape of healthcare information access.

Description:
The session will delve into the library's historical progression, highlighting its origins as a physical “space” and the pivotal steps taken to establish a virtual "space”. Attendees will gain insights into the challenges faced and lessons learned during this transition, emphasizing the library's resilience in maintaining its research and customer-oriented focus over time and through the transition from physical to virtual.

Key positions integral to the library's virtual success will be outlined, showcasing the roles and responsibilities essential for effective operation. Emphasis will be placed on the dynamic web design and communication tools that are instrumental in facilitating a seamless transition to a digital platform. Attendees will have the opportunity to explore the technological infrastructure that enables the library to provide comprehensive support to researchers within the hospital.

Additionally, the presentation will outline the library's future plans, emphasizing ongoing developments and partnerships with other groups dedicated to evidence-based medicine in the healthcare system. The collaborative initiatives with external entities will be discussed, underlining the library's commitment to fostering a network focused on advancing medical research and patient care through evidence-based practices.

Conclusion:
This session offers a comprehensive exploration of the evolution of a small research-focused medical library into a primarily virtual entity, supporting the top ranked pediatric research hospital. Attendees will leave with a deeper understanding of the key positions, technological tools, and strategic partnerships that contribute to the library's success in the ever-evolving landscape of healthcare information management.

Examining Institutional Evidence Synthesis Publications to Inform Outreach & Provision of Service for an Academic Library’s Evidence Synthesis Service

Track(s): Information Services

Philip Espinola Coombs, Health Sciences Librarian, Northeastern University

Background:
Our institution–like most institutions–has seen the number of evidence syntheses published by institutional affiliates increase substantially over the last decade. The increased number of publications corresponded with an increased need for librarian support. This prompted the development, in 2018, of dedicated library support for evidence synthesis at our institution, which is a multi-campus R1 university. The scope and formality of Library support has changed and grown significantly over the last six years. As a step towards further advancement of our support services, we wished to systematically examine our institutional affiliates’ evidence synthesis publications over the period 2018 to the present.

Description:
The purpose of the project was to answer three related questions: 1) Are there differences in the patterns of evidence synthesis production among disciplines represented at our institution?, 2) Are there differences in librarian involvement among disciplines represented at our institution?, and 3) How might these findings influence our service provision, education, and outreach with regards to the Library’s Evidence Synthesis Service?

Searches for evidence syntheses with institutional affiliates as authors were conducted in PubMed, Web of Science, Academic Search Complete, and Education Research Complete covering the dates of 1/1/2018 to

11/8/2023. Included papers were categorized using the typology in Sutton, et al, 2019. Each manuscript was reviewed to identify 1) the degree of librarian involvement and 2) the librarian’s institutional affiliation.

Spreadsheets were produced with summary data examining authors, disciplines, evidence synthesis types, and data on librarian involvement. For internal outreach purposes, separate spreadsheets were created for each liaison librarian. These liaison-specific spreadsheets included author and publication data relevant to those disciplines the liaison supports. Liaison-specific spreadsheets were shared with individual liaisons prior to a departmental meeting during which the project was presented.

Conclusion:
This systematic examination gave us a clearer picture of evidence synthesis production at our institution. Evaluating the data allowed us to identify the most ‘productive’ authors and disciplines, illustrated patterns in affiliate status and discipline, and suggested changes to be made to our LibGuide and website presence, such as the inclusion of more language, examples, and relevant guidance from psychology, our top disciplinary producer of evidence syntheses. We also discovered departments which could be ripe for targeted outreach due to their frequency of working with librarians from other institutions.

The project proved its value by giving us the data for productive internal outreach to liaison librarians, as well as presenting us with possible avenues for improved service provision and growth.

Exploring Healthcare Data with Novice Medical Marketers: A Collaboration Between a Health Sciences and Business Librarian on a Marketing Class

Track(s): Education, Information Services

Abigail Morgan, Social Sciences Librarian, Miami University

Megan Jaskowiak, Health and Social Sciences LIbrarian, Miami University

Background:
At a mid-sized public university, a capstone marketing class in which students gain valuable hands-on experience with real world clients worked with a medical device manufacturer as their semester-long project. Development of a marketing plan for a product requires extensive research with demographic and industry data and an understanding of how the product will be used. The business librarian is embedded into the course to support students with finding data and industry research. However, marketing medical devices requires knowledge of health resources not typically used by business librarians. Students were business majors, not pre-med or life sciences, which meant they had little context for medical research databases or even know where to begin searching.

Description:
To gain familiarity with the products and services being marketed and then pass that knowledge on to students, the business librarian met with the health sciences librarian prior to the start of the semester to discuss the library’s resources, the parameters of the project, develop keywords, and to look for data availability. Medical resources like AccessMedicine, UpToDate, and the Elsevier EBS e-book collection which were used to answer questions about terminology and surgical procedures. The two librarians also met throughout the semester to collaborate on answering more specific student questions as they arose. Students wanted to know things about patient journeys for particular surgical procedures, readmittance rates, and how many doctors, nurses, and other staff are usually in the operating room during surgery. The library doesn’t have access to academic versions of American Hospital Association data or medical marketing data like GlobalData Medical so we were very reliant on free federal data to address these

questions. However, federal medical databases are not generally built for marketing research purposes. After discussing options with the health sciences librarian, the business librarian used PubMed, HCUPnet (from Agency for Healthcare Research and Quality), Medicare and Medicaid data to find information for the class.

Conclusion:
Each student group was expected to prepare and present a 20 minute presentation of their final marketing plan supported by research to the client. This served as their final grade for the semester. The business librarian attended the final presentations and observed which resources students cited in their finals. Articles that were found on PubMed were used the most often, especially ones that covered specific kinds of patients, how many there are, and if the number is increasing. One surprising discovery made during this project was how such granular data was often found only in articles found during searches in PubMed. If marketing students work with a medical manufacturing client again in the future, we will recommend PubMed more often.

Finding the Bottlenecks in Research Data Management

Track(s): Information Management, Information Services, Innovation & Research Practice

Jacqueline Gunther, Research Engagement and Data Curator, Cold Spring Harbor Laboratory

Background:
In response to the NIH data management and sharing (DMS) mandate, the institute formed a committee which included OSP, the Office of Research, scientists, and the research library. The Library was asked to lead the effort for educating the community on the new policy and reviewing DMS plans prior to submission. As one of the Science Informationalist, an expert in data and a PhD in a scientific field, I was tasked to consult with applicants on the their DMS plans and to approve them. Throughout this process, I saw the need to give researchers better tools to help them quickly and efficiently curate data. Therefore, I seek to determine what the bottlenecks are for data management at the ground level.

Description:
Data Management is important for scientific research, but is often overlooked during the day-to-day routine in the laboratory. I invited the labs in five major research fields to participate in an assessment. The goal was to understand what type of data each lab produces. A spreadsheet of the experiment, type of data acquired, how the data was acquired, whether there is associated metadata, etc. is filled out to better understand the workflow of a specific lab. The spreadsheet was developed based on the NIH DMS policy requirements. During this interview process data management challenges are identified. Then a report is generated and confirmed by members of the lab. Then immediate and/or long-term solutions are generated. For example, a lab may need to comply with certain data standards in order to share data on a specific repository. I can help create templates that will comply with the data standard. Another issue may be creating metadata from a pdf that was acquired from an outside lab. In that case, tools can be created through Python to expedite metadata creation. A resource pack will be created based on each lab’s needs. These resources can then be disseminated for the benefit of other organizations.

Conclusion:
We will evaluate the success of the program by following up with the researchers and seeing how often they use their resources. This can be done as a simple survey or follow-up call. Additionally, the library helps researchers share their data and will give us another marker of how well the data is being managed. The goal would be the researchers using these tools to make their data management faster, more efficient, and accessible. We expect to encourage researcher towards better data management practices by making the

process as easy and smooth as possible. Once we can manage this at our own institutional level, we plan to disseminate our materials so that other libraries can benefit from our program.

Fostering Future Librarians: Using Moodle to Transform Internships

Track(s): Education

Erica Lake, Health Sciences Librarians Outreach Specialist, Network of the National Library of Medicine, Region 6

Abby Dowd, National Training Office, NNLM

Annisija Hunter, MLIS Student, University of Missouri

Darlene Kaskie, Associate Director, Region 6, Network of the National Library of Medicine

Background:
Championing health sciences librarianship to Library and Information Science (LIS) and other graduate programs is critical to growing the field, and our organization is committed to offering engagement opportunities for these students. Having hosted an LIS student for a week-long Alternative Spring Break experience in 2023, we wanted to expand our offering into a 12-week virtual internship. We knew we had a meaningful and important project to offer students; however, our staff lacked the capacity to dedicate weeks to education and training, essential for developing the required knowledge and skills for the work. The solution lay in leveraging the online learning platform, Moodle, to build an internship hub.

Description:
Two staff members agreed to serve as instructors for the internship and invited an in-house instructional designer to build out the Moodle shell. The team organized the project work into three phases – Learning, Building, and Sharing – with each phase representing four weeks of the internship. Readings, videos, and tutorials were loaded into the Learning phase to support successful independent work. These were created using Moodle pages and Moodle assignments. The Building phase included software and resources needed for the project, as well as areas to share and store work in progress. The internship culminated in the Sharing phase, with the student presenting on the completed project work and overall internship experience. The instructional designer enabled a Wiki journal on Moodle for the students to share weekly reflections on their training, project work, and related observations. Other communication channels included weekly overviews created using Moodle pages that provide learners with important information and links for the weekly check-in meetings with the instructors. The Moodle class also included specific areas where the instructors could provide announcements to all participants. The result was a virtual office for the student, housing everything related to the project and the internship in one spot.

Conclusion:
Several strategies will be used to evaluate the effectiveness of using Moodle to host internships: gathering regular student feedback through Wiki journal entries will allow adjustments to be made during the internship; two student self-assessments will help determine if the experience matched their expectations; and a final survey related to the Moodle platform will be administered to the student and instructors to gather feedback to improve the internship experience. Although an initial investment of time was required to build out the Moodle, staff now has a tool that is ready to go whenever an opportunity arises to host students. Creatively using online learning platforms like Moodle can enable other libraries to provide engaging, virtual internships despite limited staff capacity.

A Framework for Auditing Resource Access

Track(s): Information Management

Linda Van Keuren, Asst Dean Resources & Access, Georgetown University Medical Center

Background:
The newly developed Audit Toolkit enables libraries to review their resource access in four areas usability, privacy, reliability, and security. The objective in using the toolkit is to identify and address potential access-related issues, assess potential ethical or legal exposure, and identify best practices and recommended next steps for moving forward if problems are identified.

Description:
This presentation will focus on one health science library's experience using the toolkit to identify and address potential access-related issues, assess potential ethical or legal exposure, and identify best practices and recommended next steps for moving forward. Multiple major resource platforms will be examined using the toolkit and the experience of using it will be shared in the presentation.

Conclusion:
The expected outcome is an understanding of the toolkit and the benefits that can be gained by utilizing an access audit. Some expected outcomes of using the toolkit are 1. information collected in the audit can be leveraged to make suggestions and negotiate with vendors; 2. librarians can use the information to advocate for the privacy of user data; 3. "pain points" in the access and authentication process can be identified and resolved and 4. information gathered can serve as a valuable guide for teaching and training users understand the value of their online identity.

From Gowns to Towns: Partnering with Community Organizations to Teach Health Literacy Skills

Track(s): Education, Information Services

Karen Grigg, Health Sciences Librarian for Collections and Instruction, UNC Chapel Hill Health Science Library

Terri Ottosen, Community Engagement and Health Literacy Librarian, University of North Carolina at Chapel Hill Health Sciences Library

Background:
Locating reliable health information and managing health care are important to older adults. Additionally, millions of Americans serve as caregivers for someone with a serious health condition. The Health Sciences Library (HSL) at the University of XXX serves the citizens of XX as part of its mandate to give back to the community. The University’s mission statement includes the provision that we share knowledge-based services to the citizens of XX. The instruction team prioritized rural and small town communities, as these citizens are often underserved. The Health Sciences Library recently partnered with two offices of the Area Agency on Aging to bring workshops to older adults and caregivers in two small towns.

Description:
After the Health Sciences Librarians reached out to several organizations, they identified several potential collaborators and conducted a thorough needs assessment to determine the specific information needs and services of underserved populations in rural XX counties. Both contacts at Area Agencies on Aging

mentioned having a shortage of instructors and were happy to plug the team into their workshop schedules. One of the collaborators thought the seniors in the community would be interested in the proposed instruction session finding reliable health information on the Web, talking with doctors, avoiding health care-related fraud, and some helpful mobile apps to manage wellness and health. The collaborator for the other community suggested that she add a similar session to a previously scheduled all-day conference for caregivers. The librarians provided two workshops in two small towns in XX. During the workshops, the instructors emphasized better starting places for older adults and caregivers to find quality health information, discussed tips for talking to doctors and avoiding health care fraud, and provided attendees with a handout for further information and websites to explore, as well as contacts for further assistance.

Conclusion:
Both workshops were well-received. The Health Sciences Librarians followed the workshops with several discussions on lessons learned and future plans. One lesson learned was that attendance is increased when workshops are part of a larger event. This presentation will discuss other lessons learned and future plans and collaborations, as the HSL has plans to form other partnerships and to continue providing outreach throughout the state, including underserved rural and urban areas, as well as with indigenous communities.

From Query to Answer: A Health Sciences FAQ at a University Library

Track(s): Information Management, Information Services

Eleni Philippopoulos, Liaison Librarian, McGill University

Sabine Calleja, Liaison Librarian, McGill University

Background:
Despite best efforts in teaching the basics of health sciences research, librarians receive a high volume of questions on the same topics from their students. To mitigate the repetition in answering these questions, which can take up valuable librarian and student time, a team of health sciences librarians at an academic institution put together a health sciences FAQ hosted on the SpringShare LibApps software.

Description:
Consisting of 22 initial questions organized among eight categories, including Knowledge Syntheses, Searching, Databases, Citations, Evidence-Based Practice and others, these FAQs transcend the specific health sciences disciplines. Questions were selected based on consultation with the team of health sciences librarians, and common themes were extracted and consolidated based on these conversations and the authors' own expertise and experience teaching and interacting with students and faculty in the health sciences. Draft answers were compiled by the authors of the FAQ page and verified by all team members to ensure accuracy and compliance with library service guidelines. The guide was added to an existing page that houses FAQs for other subjects and presented to the library team at the university as a new tool.

Conclusion:
We will be measuring user interactions with the individual questions on the FAQ, including page visits, page likes/dislikes, comments on questions and requests for additional support. We are hoping that our fellow liaison librarians, who are sometimes asked reference questions relating to health sciences, will also provide feedback on its use and content once they become more familiar with it.

Implementing Zscaler as a Remote Access Solution – What the Library Needs to Know

Track(s): Clinical Support, Information Management, Information Services

Liz Suelzer, Application Support Analyst, Sr, Advocate Health - Midwest Library

Willie Lasticly, Cybersecurity Engineer Sr, Advocate Health - Midwest Region

Background:
A large healthcare system supporting remote access to internal resources and IP-authenticated websites recently switched from Citrix AnyConnect to Zscaler as their remote access solution. This change caused problems with access to library resources and required library and health information and technology (HIT) staff to collaborate to restore access. Zscaler is a cloud-based ‘zero-trust’ remote access solution. By default, it trusts no one inside or outside the network; verification is required from everyone trying to gain access to corporate assets. Zscaler provides more secure access to the internet and internal resources than a traditional VPN. Because of the enhanced security, more healthcare systems are implementing Zscaler. Hospital libraries should be aware of this product in case their institution implements it.

Description:
This presentation will provide a brief overview of how Zscaler differs from other VPNs, it will address how the problem with access to library resources was resolved at our organization, and it will provide tips for those who are transitioning to Zscaler. Cooperation between the library and HIT professionals was needed to ensure that access to library resources worked in the new environment. Library staff maintain subscription resources and websites that needed to be cleared with Zscaler. Additionally, the library staff were able to recognize when problems surfaced with the resources. They also provided information to HIT about library access and what a typical experience should look like. HIT staff were able to safelist the necessary URLs within Zscaler and interfaced with Zscaler support when questions arose. Our organization uses Zscaler Private Access (ZPA) as an alternative to a VPN, and Zscaler Internet Access (ZIA) to provide secure access to public websites. Because Zscaler is a cloud-based solution, the websites for library resources needed to be redirected from ZIA to ZPA to mimic the behavior of a traditional VPN and make it appear as if the communication is initiated from inside the organization’s network.

Conclusion:
Zscaler was rolled out at our institution over a few months and the library was made aware of access issues before too many people were relying on Zscaler for remote access. Conversations between HIT, library patrons and library staff were necessary to determine why the access issues were happening. Working with a dedicated team of HIT and library staff allowed the small group to focus on resolving access issues and setting up website URLs in Zscaler. Maintaining a current list of library subscriptions and URLs for library websites that use IP authentication was vital. As new library resources are purchased and website URLs change, communicating this to HIT so that Zscaler can be updated is now part of our workflow.

Integrating Journal Impact Factor Metrics into Citation Reports

Track(s): Information Management, Innovation & Research Practice

Liz Kellermeyer, Library Director, National Jewish Health

Ruby Nugent MA LIS, Biomedical Research Librarian, National Jewish Health

Background:
Objective: Add journal metrics, including JIF and journal quartile ranking, to an existing WoS citation report. Although the Web of Science (Clarivate) database offers a large amount of statistical information with their existing citation reports, metrics about individual journals are not included in the output. Our institution's promotion and review committee evaluates the quality of journals where faculty are publishing, and asked for this information to be integrated into citation reports. Librarians developed a process using Microsoft Excel to incorporate these JIF metrics into the reports. The supplementary data included by incorporating these formulas provides a broader context for an individual's scholarly impact and standardizes the content for each report that is created.

Description:
Methods: At the annual release of Clarivate's Journal Citation Reports, librarians can create an updated list of journals where faculty at their institution are publishing. This list can be customized to include individual JIFs and JIF quartile rankings. After running a WoS citation report and exporting it to Excel, librarians can then query this master journal list using INDEX and MATCH functions to populate JIF and JIF quartile rankings for each journal title listed in the citation report.

Conclusion:
Results: The result of this method is that these citation reports provide a simplified way for the review and promotion committee to evaluate the caliber of journals where faculty are publishing. While not a definitive measure of quality, a journal's impact factor and its JIF quartile ranking offer standardized metrics that can be used as a comparison standard. Along with data already provided in the WoS citation reports, librarians are able to create a customized report that includes additional standardized fields recognized as valuable benchmarks of scholarly impact by researchers and practitioners. The librarians have been recognized as the experts for these reports and are regularly invited to participate in departmental and research meetings to talk about the process of creating these important documents.

Integration of a Medical Librarian into a Clinical Teaching Certificate Program Enhances Teaching of Evidence-Based Medical Education

Track(s): Clinical Support, Education, Information Services

Anna Liss Jacobsen, Health Sciences Librarian, Indiana University Indianapolis

Patricia Kritek, Professor of Pulmonary, Critical Care and Sleep Medicine, Vice Dean for Faculty Affairs, University of Washington School of Medicine

Somnath Mookherjee, Professor of Medicine, Division of General Internal Medicine, Director of Center for Learning & Innovation in Medical Education, School of Medicine, University of Washington

Background:
Health sciences faculty are expected to be effective educators, but few have had training on how to teach effectively. To address this gap, we created a Clinical Teaching Certificate (CTC) program to support teaching excellence at a large medical school employing nearly 9,000 teaching faculty. The CTC goals were to strengthen the clinical teaching skills of faculty interacting with learners at all levels, foster a shared mental model of teaching excellence, and offer an institution-wide, standardized, durable health sciences teaching curriculum. The CTC was launched in 2021-2022 and included six synchronous sessions with accompanying learning materials. A medical librarian embedded in the CTC team provided in-depth, evidence-based education theory and practice support strengthening the CTC program content, communication and quality.

Description:
The program director identified CTC goals by reviewing the literature and existing programs. Next, the director created a curriculum map and, with input from diverse stakeholders, finalized program goals. These included maximizing learning climate, optimizing learning content delivery, and assessing and providing feedback to learners. The final curriculum included six learning outcomes presented as six online synchronous sessions with corresponding asynchronous learning modules. Participants were required to complete all six sessions and modules to earn the certificate.

Core faculty were selected to develop and lead the six sessions via a competitive application process. The medical librarian applied to serve as core faculty and was recruited to serve as an embedded librarian. The librarian's goals were to: (1) provide evidence-based education literature supporting session development, and (2) attend sessions, document questions and provide resources answering questions.

Program evaluation included post-session survey of participants and evaluation by core faculty of librarian support. Core faculty were asked:

1. How did the librarian improve your session?
2. How could you have better used a librarian's support?
3. Other thoughts?

Conclusion:
The first CTC cohort had 218 registrants. Sessions averaged 129 participants; 51 earned the certificate. Evaluations were consistently high. Suggested improvements included teaching to a broader audience, revising active learning techniques and more.

Librarian support extended beyond the original goals and was tailored to core faculty needs and requests for support. The librarian engaged with all core faculty and contributed to most sessions, including developing content, slides and active learning opportunities for some sessions. She attended and provided follow-up support for three sessions. Themes from the core faculty survey around the role of the librarian included: her support enhanced the evidence base and quality of the sessions, sessions were developed more efficiently, and opportunities for further engagement in the future.

Interactive, Inclusive, and Impactful—Engaging Stakeholders in Collection Development Policy Revision

Track(s): Information Management, Information Services

Li Ma, Duke University Medical Center Library

Megan von Isenburg, Associate Dean for Library Services & Archives, Duke University Medical Center Library

Background:
Collection development policies have not always reflected the rapid changes in health sciences resources, institutional budget realities, and current practices. At this institution, there were separate policies for serials, books, e-resources, and manuscript collections, all of which were eight years old. While policies tend to be mired in library jargons and technical processes, we sought to foster understanding and trust by engaging non-collection colleagues and non-library stakeholders in the policy revision process.

Description:
Library staff developed specific use cases based on recent collection requests, such as requests for new journals, new clinical reference tools, curricular teaching materials, and novel serial subscriptions based on

alternative pricing. Use cases were presented at a Library Advisory Committee to solicit perspectives from a broad array of external stakeholders, including students, faculty in multiple programs, research administrators, and clinicians. Feedback and perspectives were captured and analyzed against current collection practices. Many external stakeholders indicated interest in new approaches to funding requests, such as collaborations with requesting departments, and support for the library to deny collection requests. Proposed changes were reviewed and discussed by all library staff, including those outside the collections department. Several changes resulted, including merging all previous policies into one and framing decision-making around criteria librarians should use rather than prescribing specific conditions. The policy review also resulted in changes to the faculty authors collection: while it had previously been a closed collection focused on preservation, it is now a circulating collection with an emphasis on access.

Conclusion:
The revision process enabled collections librarians to assess priorities and perspectives of internal and external stakeholders. The culmination of these efforts not only emphasizes diversity, equity, and inclusion (DEI) principles but also results in increased access to previously restricted collections, notably the faculty author works. Stakeholders in the Advisory Committee developed knowledge of collection practices extending beyond the routine challenges librarians face, delving into budgetary considerations and the complexities of collection management. Highlights of the resulting policy are now shared on the library's website for all patrons to review. We believe the transparency used in this process has built greater trust in the library's budgetary discretion and decision-making.

Interprofessional Collaborative Learning: Evidence Synthesis in Nursing Science

Track(s): Information Services

Gregory Laynor, Systematic Review Librarian, NYU Health Sciences Library, NYU Grossman School of Medicine

Benjamin Bass, Research Coordinator, Center for Innovations in the Advancement of Care, NYU Langone Health

Emma Millon, Associate Director of Research, Department of Integrative Health, NYU Langone Health

Jennifer Withall, Nurse Researcher, Center for Innovations in the Advancement of Care, NYU Langone Health

Kathleen Zavotsky, System Senior Director Nursing Research and Program Evaluation, NYU Langone Health

Carlita Anglin, Lead, Programming and Clinical Support, NYU Health Sciences Library, NYU Langone Health

Fariza Alendy, Program Director, Health Promotion, Department of Nursing & Patient Care Services, NYU Langone Health

Kathleen DeMarco, System Senior Director of Nursing Wellness and Resilience, NYU Langone Health

Background:
Librarians, nurse researchers, a data analyst, and an integrative health researcher established an interprofessional collaboration to develop capacity for using evidence synthesis methods in a large, urban academic medical center. Comprised of five campus locations and 50,000 employees, this academic healthcare system has approximately 10,000 nurses and 20 health sciences librarians. The collaboration was sparked by a conversation between an academic nurse scholar and a health system-based nurse

researcher about the need to systematically characterize the state of the science on interventions for nurse wellness. The interprofessional team came together to turn this specific need for evidence synthesis into an opportunity to learn together and begin scaling up capacity for evidence synthesis in nursing science across the health system.

Description:
The interprofessional team developed a novel evidence synthesis collaboration for this academic health system. A scoping review was designed to characterize how wellness interventions for nurses have been implemented and evaluated at a health system level. The scoping review methodology enables us to study nurse wellness interventions at a macro level, beyond one-off interventions, and thus make informed recommendations to executive leadership.

This project fosters institutional capacity for high-quality evidence synthesis in an academic health center setting. Advanced researchers in nursing schools often undertake evidence-synthesis projects rather than those working in an academic hospital setting. Although clinical nurses are frequent consumers of synthesized evidence, they are less involved in its production, and this is something we want to change.

The team brought together existing efforts at the institution, including a robust integrative health research initiative, embedded nursing librarianship, library systematic review instruction, and nursing science scholarship. Findings from this scoping review will inform how the institution directs resources for nurse wellness. Additionally, the collaborative interprofessional learning experience of conducting the review will build capacity for future evidence synthesis projects in nursing science.

Conclusion:
Evidence synthesis projects are resource-intensive work. We capitalized on the need for this review as an opportunity for team members to build competence and efficiency using techniques of evidence synthesis. A critical outcome of this project will be an expanded institutional capacity to address future evidence needs and to serve as an exemplar for other nursing synthesis projects. The collaboration bridges the clinical-academic environments and represents important opportunities for scholarship within academic health centers. There is potential for further nurse engagement in producing evidence syntheses by connecting into existing institutional structures such as our nursing fellowship program and specialized nursing councils. The project empowers participants to understand, apply and communicate evidence synthesis more deeply.

Investigating Machine-Actionable Data Management Plans and Shedding Light on Research Data Management Infrastructure at an Academic Medical Center

Track(s): Information Management, Innovation & Research Practice

Michelle Yee, Research Data and Metadata Management Librarian, NYU Langone Health

Nicole Contaxis, Head of Data Sharing and Metadata Management, NYU Langone Health

Background:
As part of an enterprise-wide effort to enhance data governance at an academic medical center, a team of librarians developed a project to assess the tools and features of data management infrastructure that presently support funded research at the institution. The primary objectives are to (1) investigate how data management information is currently communicated across the institution, and (2) how to utilize the information reported in data management plans to ease research operations. This undertaking would bolster ongoing work in the library science community to design and implement maDMSPs.

Description:
This effort leverages qualitative data collected from focus group discussions and interviews, as well as the content of data management and sharing plans (DMSPs) submitted to the NIH following the implementation of their new policy. Focus groups and structured interviews with stakeholders involved in research (i.e., scientific cores, institutional review board, information technology, investigators) will identify what information is communicated for standard operations and compliance with internal and external policies. Questions and prompts will solicit information such as participants’ interactions within and across departments, types of messages exchanged, and reporting needs. The librarians also plan to assess the content of submitted NIH DMS plans to examine investigator-reported data management and sharing resource needs. Project partners across the institution will assist with the recruitment of participants for the interviews and focus groups, as well as provide access to research tracking systems. Findings from these efforts will be mapped to metadata for a prototype maDMSP and charted in diagrams that illustrate the flow of information between researchers and research support offices.

Conclusion:
The project is still underway. Outcomes will include identifying key roles of the network of research offices across the institution, resource acquisition pipelines, type and scope of information communicated between parties, and routes for monitoring and reporting research for compliance with regulations. These findings will enable the authors to identify pain points and research operations that maDMSPs can facilitate. They will be shared to guide future best practices for the communication of research data management information with maDMSPs.

Libraries and Patient Portal Instruction: Stronger Together

Track(s): Health Equity & Global Health, Information Services

Sydni Abrahamsen, Librarian III, Mayo Clinic

Melissa Wagner, Librarian, Mayo Clinic

Background:
Patient health portals have been shown to create greater patient engagement with the health and medical process. However, there are barriers to entry for some users as they require high levels of digital literacy, health literacy, and comfort with technology. Librarians are skilled at providing technology help and instruction. Offering one-on-one portal help in the library fills an unmet need. Patient portal assistance is another service the library can offer to add value for providers and other library stakeholders.

Description:
We identified a gap in service at our institution: patients needed hands-on help setting up and using their patient portals, but the only assistance available was through a helpline. Patients were frequently referred to the library with portal issues because we have computer stations, however, staff had no formal training in offering assistance. We felt that offering in-depth, hands-on help with the portal could increase library visibility and relevance as well as increase satisfaction with the patient portal.

Our first step was to design and distribute a survey asking patients about their portal use and interest in assistance. Many survey takers indicated a desire to learn more about using the portal. We then contacted the institution’s customer helpline staff about the possibility of collaboration. They were excited to partner with us to hear our feedback and to have boots-on-the-ground support. A two-day training session was held to teach library staff the basics of troubleshooting and remedying patient portal issues. After this training, we began offering one-on-one portal assistance and instruction in the patient libraries on both PCs and mobile devices.

Conclusion:
Patients are asked to complete a short evaluation form following instruction. Feedback has been overwhelmingly positive. Awareness of the service has spread through word-of-mouth, and we now receive referrals for portal help from information desks, check-in desks, and providers. Our next step will be to work with customer assistance staff to design course materials for a group class that will be offered later this year. The class will be evaluated with pre- and post- surveys to determine impact of the instruction.

Library as the Catalyst for Publisher and Professional Society Collaboration

Track(s): Education, Professionalism & Leadership

Bryan Hull, Head, Digital Publishing, Spencer S. Eccles Health Sciences Library, University of Utah

Background:
The project began in 2003 as a partnership between a professional medical society and an academic health sciences library. The project developed an open-access repository of educational materials specific to the medical society's discipline. The repository continues to be used for educational and research purposes by health care professionals, educators, patients, and students. Funding for the project was initially supported through grants but eventually became solely part of the medical society’s annual budget.

Description:
The executive board of the professional medical society encouraged the project’s steering committee to seek a revenue source to support the project. The project committee developed a business model based on licensing products through a publisher in order to generate royalty revenue. A curriculum was targeted as the initial product to develop for licensing to provide institutional access beyond members of the professional medical society and develop a revenue source. The steering committee met with representatives from a publisher that had a platform appropriate for the curriculum. A contract was negotiated between the professional society and publisher in order to give this nontraditional publication a chance. Success of this endeavor was predicated on steady revenue being generated for the sustainment of the project.

Conclusion:
The curriculum project went live on the publisher's platform in 2017 and received a major update in 2020. The curriculum contains 2,234 files of learning materials organized by the curriculum outline and is updated quarterly with new submissions to the repository. A second product was developed and launched in 2019 based off a survey of general health practitioners for a basic techniques curriculum in order to meet wider audience needs beyond those within the medical society's discipline. The collaboration continues to be a fruitful endeavor for the publisher, professional medical society, and academic health sciences library.

Marketing with AI: Creating a Marketing Team Prompt Library

Track(s): Information Services

Amanda Woodward, Web Services Librarian, Stanford Lane Medical Library

Sonam Soni, Resource Management Librarian, Stanford Medicine

Background:
Academic medical libraries need to create engaging marketing content to build awareness for and drive the use of library resources and services. This can be a challenging task given the variety of audiences that medical libraries serve, including students, residents, fellows, clinicians, faculty, researchers, staff, and others. In addition, marketing content needs to be customized for different communication channels, including email, social media, and blogs. Executing an effective marketing strategy with tailored content for distinct audiences and platforms is difficult, especially if a library does not have a dedicated marketing specialist. The three staff members composing our Marketing and Communications Team (MCT) at our academic medical library are experimenting with generative AI tools to create marketing content efficiently.

Description:
We are taking an exploratory approach to using generative AI tools in our marketing tasks. Each team member is trying a variety of tools, prompts, and iterative prompting to assist in their typical marketing content creation. As we discover successful methods for generating content, we are building a prompt library in Google Sheets. We are capturing the category, task, chatbot, prompt text, variables, and recommended iterations in our sheet. Our goal is to build a reusable library of prompts for marketing content creation to use collaboratively. For example, we have a regular Resource of the Month campaign that includes an instructional blog post typically written by a liaison librarian. We created an AI prompt to help draft these posts and then shared it with the liaisons as a way to save writing time. We also experimented with using AI tools to reformat content for particular platforms. For instance, we used ChatGPT to transform written instructions into a tweetorial for X/Twitter with a set number of posts that fit the platforms’ character limits. We plan on evaluating which marketing content tasks are more efficient with AI tools and when it’s best to avoid using AI tools for our marketing efforts.

Conclusion:
Through this experimentation phase, our goal is to build a core set of AI prompts for our regular marketing tasks throughout the year. We plan to evaluate the effectiveness of the prompt library by tracking how often we use the prompts while creating marketing materials. We will also survey our MCT staff and liaisons who help with marketing content to see if they used the shared AI prompts and how effective the prompts were at saving time while creating content. We plan to present the survey findings and share how well the prompt library helped with achieving our primary desired outcome of time savings.

Medical Library Bioinformatics Research Reproducibility Service

Track(s): Innovation & Research Practice

Mengrui Zhang, Informationist for Bioinformatics and Data Integrity, Weill Cornell Medicine

Sarah Ben Maamar, Associate Director for Research Services, Weill Cornell Medicine

Terrie Wheeler, Library Director, Weill Cornell Medicine

Background:
In the rapidly growing fields of academic research, the aim of research are not only groundbreaking discoveries but also a commitment to the transparency and reproducibility of scientific investigations. One of the objectives in our medical library is to redefine scientific integrity and collaboration through its Research Reproducibility Service. The purpose of this service in the library aims at fostering best practices in research reproducibility, especially in bioinformatics, and responds to the growing call from NIH for more transparent and reproducible funded research.

Description:
To enhance the Research Reproducibility Service in bioinformatics, we have built comprehensive, end-to-

end bioinformatics reproducibility pipelines including consistent analysis results, code version control and sharing, compute environment control, and a documentation guide. Currently, the process is to gather complex methodologies employed in bioinformatics analysis into reproducible pipelines for a single research study. For example, in single cell analysis, We stored the code in GitHub with versions controlled for the parameters used in analysis of normalization, cell clustering or finding marker genes. We packed the computing environments into a Docker container and backed up the raw/processed data in Cloud Storage (AWS). The pipeline will automatically run and connect them all together. This approach guarantees that the entire analytical pipeline produces the same results reliably each time it is executed. To evaluate the service we will conduct a survey of customers and present preliminary results to assess how well our service is improving the reproducibility in a given research study. Additionally, we plan to offer more online educational material on how to perform reproducible bioinformatics analyses as well as expand our support to various bioinformatics analyses such as single cell analyses or analyses automation.

Conclusion:
We have constructed pipelines in RNA-Seq, single cell and Metagenomics analysis to reproduce consistent results for research and teaching purposes. To measure the outcomes of this service, we expect the survey to include but not be limited to reproducibility success, time and resource efficiency, and training effectiveness. Unlike other bioinformatics cores, the medical library has unique inclusive, educational, and collaborative mission. Therefore, we think the medical library is an ideal place for making bioinformatics reproducibility pipelines available to a wide range of disciplines and a diverse range of researchers. The future of this service will contribute to the bioinformatics research landscape in an effort to support transparency, reliability, and integrity of research in the medical field.

Multi-Stakeholder Collaboration to Develop Bioinformatics Information Resources for Novice Learners

Track(s): Education, Innovation & Research Practice

Aida Miro-Herrans, University of Florida

Ashley Beard, Graduate student, University of Florida

Background:
Bioinformatics is the use of computational tools and methods to solve biological problems. Given the large amounts of data and tools that are continuously generated for genome sequence studies, novice bioinformatics learners find it challenging to identify the appropriate tools and methods to analyze their data and begin learning computational skills. The bioinformatics librarian at a research and health library partnered with the director of the bioinformatics core at the research institution and a novice bioinformatics graduate student to develop learning resources for novice bioinformaticians from a range of users, including students, faculty, and clinicians.

Description:
While the bioinformatics librarian directed the project, the content in the resources was driven by the learning needs of the graduate student and informed by the practical experience of the core director. The first resource is a glossary of vocabulary related to bioinformatics and genomics, with definitions, synonyms, and related terms. It was developed for both novice bioinformaticians and librarians searching for bioinformatics and genomics terms. The second resource provides best-practices in bioinformatics methods for genomic sequence processing. Both resources are available online as open educational resources.

Conclusion:
Usage statistics show that the resources have been accessed through the library website over 250 times

since they became available. They are also being included as learning resources in genomics courses at the bioinformatic librarian’s academic institution.

Online Instruction in Publishing Academic Research for an Invested Student Population

Track(s): Information Services

Heather Brown, Scholarly Communication Librarian | Head of Access Services, University of Nebraska Medical Center

Background:
Publishing scholarly academic articles is central to the reporting of research in the health sciences. Students across disciplines do not generally receive formal education in navigating the publishing environment within the curriculum but may receive from mentors or through experience. The 2023 Student Satisfaction Survey, administered by Division of Student Success and Office of Accreditation and Assessment, reported that 19% (n=374) of respondents indicated that they want additional information, training, or tools in publishing academic research.

Description:
Seeing the survey results, the library made providing instruction on the publishing process part of its strategic plan. Initial steps involved reviewing the literature on such endeavors and seeking input on topics and format from stakeholders. Based on the timeline and variations of academic program schedules, seven online accessible videos, faculty interviews, and resource lists will be developed and placed on a research guide and publicized. Objectives will be mapped to institutional learning objectives and the ACRL information literacy framework. Use of the publishing instruction materials will be piloted in the summer of 2024 with various student programs before being launched in August 2024.

Conclusion:
The instructional videos will be eligible for the Graduate Studies Service-Learning and Professional Development Badge Program. Self-assessment questions and feedback forms will be available for each video and will inform future instruction.

Oops! They Did IT Again…The Finale

Track(s): Information Services

Janice Thompson, Team Lead, William Osler Health System

Background:
Most community hospitals rely on Internet Protocol (IP) authentication for their resources. Hospital Information Technology (IT) departments don’t always comprehend the way publishers authenticate library resources and can be loathe to move to proxy servers. At our organization, we dealt with several IP address changes and often had to contact publishers after the changes had been implemented. The tipping point was our move to Zscaler IP addresses – cloud based, random and shared IP addresses. All of a sudden, our IP addresses were no longer acceptable and access to our resources became endangered.

Description:
To provide access to our library resources we became adept at devising work arounds – whitelisting static IP addresses, embedding the username & password in a custom URL on our Intranet, token access - none of which were ideal solutions and still left remote access to mostly usernames and passwords. During a conversation with a colleague, it was mentioned that they were using a product called LibLynx for remote authentication with single sign-on. The light bulb went on! If LibLynx provided IP authentication, then perhaps we could use their IP addresses to authenticate our resources with the bonus of seamless staff authentication with remote & mobile access.

Conclusion:
We are now using LibLynx as our Intranet webpage and all our resources are now easily accessible. Our I.T. department assisted with the single sign-on integration so that users have seamless access on or off site. LibLynx offers customization and we’ve made use of this by displaying relevant resources to certain groups in our hospital, the addition of request forms, subject guides, the library newsletter, and email banners for promotion. The reporting component of LibLynx also allows us to see which departments or roles are using our resources. This along with resource usage statistics will provide a true picture of how our resources are being used.

For the library team, LibLynx has taken the pain out of our authentication woes. LibLynx offers a reliable, seamless, and customizable way to access our resources on and off site.

The Organization Is Stronger When We Build It Together: A Collaborative, Librarian-Focused Organizational Redesign

Track(s): Professionalism & Leadership

Erika Sevetson, Director of Health and Biomedical Library Services, Brown University Library

Background:
At a medium-sized private university library, support for the health sciences historically was organizationally a part of a large interdisciplinary department of subject librarians. Although health sciences library services became a distinct unit in the organization, job descriptions retained the legacy structure of “one librarian to one department.” In other words, each description and title was unique, with little overlap between librarian roles. This resulted in complicated titles, a lack of flexibility to respond to existing needs, siloed work, and burnout. A vacancy in one position, in addition to the creation of a new FTE line, created an opportunity to create a structure that will clarify roles, provide individual growth and leadership development, and emphasize collaboration and shared responsibility.

Description:
After an informal review of job descriptions and organizational structures at other health sciences libraries, the Director created three potential organizational structures, focusing on job descriptions and the need to assign work in functional areas as well as on a subject basis. The intention was to create a structure that would be responsive to evolving needs, and no reporting lines would be changed. The structures were presented, with pros/cons of each, to the full department of health sciences librarians, and after feedback we transitioned to a model where each librarian has a core job title (Health Sciences Librarian for [subject]), along with a shared core job description. Each description contains 15% time for “Collaborative program development and leadership” which may be redefined and reassigned at annual review periods. Areas of collaborative development and leadership are still being defined and assigned but will include coordination of: the literature review service (had previously been included in one librarian’s job description), education/instruction, collection development, and communications. The work to define the areas of need is being shared across the department.

Conclusion:
The structure is still in development, so ongoing assessment will be crucial, along with regular review of assigned and evolving leadership areas. Librarians in the department are fully engaged in the process and welcome the opportunity to build skills in a variety of realms, and the structure has been a recruiting asset. In addition, the university libraries are currently redesigning the promotional program, and the new health sciences job descriptions are being used as a template for other departments in the system. The opportunities created by “collaborative program development and leadership” areas will be critically important for the promotional process.

Partnering to Teach Evidence-Based Practice Skills at a Healthcare University

Track(s): Clinical Support, Education

Amy Ferguson, Health Science Librarian, Parker University

Background:
Like other healthcare universities, the university discussed in this talk promotes Evidence-Based Practice (EBP). However, EBP involves complex skills that cannot be developed in a single course. To ensure students receive repeated exposure to EBP and in compliance with our regional accreditation agency, the university developed a Quality Enhancement Plan with the topic of Enhancing the Use of Evidence. The goals of the five-year plan are to “Improve students’ abilities to obtain and apply literature to a question or topic” and to “improve faculty members’ abilities to provide appropriate opportunities for students to demonstrate their ability to obtain and apply literature to a question or topic.”

Description:
As a university-wide effort, the QEP involves many departments and individuals. The QEP manager recognized the need to involve the library. Many of the skills required for EBP align with the library’s mission and expertise: developing an appropriate research question, awareness of health science databases and search tools, search strategy, and critical evaluation.

One of the ways the library participates in the QEP is by co-teaching workshops and webinars with the QEP manager. By co-teaching, the QEP manager and the librarian are able to focus on their own areas of expertise. The QEP manager provides expertise in the QEP and EBP in the real world. The librarian provides expertise in research tools and search skills. Collaborative planning results in more engaging workshops. The librarian and QEP manager also benefit by learning from each other.

Conclusion:
Student learning outcomes will be accessed through qualifying QEP assignments across disciplines. Student Learning Outcomes:-Students will demonstrate an improvement in their ability to identify a question or topic for a literature search. (ASK)-Students will demonstrate an improvement in their ability to search for literature. (ACQUIRE)-Students will demonstrate an improvement in their ability to reduce bias in available literature. (APPRAISE) -Students will demonstrate an improvement in their ability to apply available literature to a question or topic. (APPLY) -Students will demonstrate an improvement in their ability to formulate a recommendation or hypothesis based on the application of available research to a question or topic. (ASSESS)

Reaching More Users by Repurposing Instructional Session Content Through LibGuides

Track(s): Education

Rob Wright, Basic Science Informationist, Johns Hopkins University

Background:
A long-standing educational program of hands-on, synchronous instruction sessions teaches effective strategies for finding biological data to clinical, public health, and basic science users across medicine, nursing, and public health schools. Instructional sessions cover searching skills in the web interfaces of gateways to genomics information, including BioCyc, the Ensembl Genome Browser, and NCBI. The NCBI sessions include a stand-alone session on the Gene Expression Omnibus and another session on finding gene-based information in the Nucleotide, Gene, and dbSNP databases. While these sessions are offered multiple times during the year and are well-attended, their format limits their potential impact. As a way of reaching more users, an easily accessible, asynchronous format for session content has been developed using LibGuides.

Description:
The instruction session on NCBI Nucleotide, Gene, and dbSNP, whose content is in a prezi, was the first of the synchronous sessions to be translated into a LibGuide. The resulting guide, "Finding Sequence Using NCBI Nucleotide," contains only the Nucleotide-based content from the instruction session. Separate, individual guides on Gene and dbSNP are planned. The translation process was a fairly straightforward mapping process, with top-level prezi sections or slides forming the structure of the pages in the LibGuide and sub-level parts of the prezi corresponding to guide boxes. A handout of the hands-on searching activity worked through in class was translated into its own page in the guide, with the individual steps in the activity forming the boxes within the page. The resulting guide has pages that provide learning objectives, background information on NCBI and Nucleotide, background on the process and components of complex searching, and a worked search example in five steps. The completed guide was advertised to users via listservs and promoted when teaching. To compare the impacts of the instruction session and the guide, attendance statistics and usage statistics will be gathered, respectively.

Conclusion:
To measure the extent to which the translated LibGuide reached more users than the instruction session, guide usage and session attendance over a common time period will be compared. The limitations of this comparison and alternative future means of assessment will be discussed once the data is in hand.

Reduce, Reuse, and Recycle Your Hospital Library Collections

Track(s): Clinical Support, Information Services

Kellee Selden, Manager of Library Services - Nationwide, Ascension

Lucinda Bennett, Medical Librarian, Ascension Saint Agnes

Background:
The objective of the Ascension print recycling program is to redirect specific resources to electronic format and reassess the needs of our medical communities. In considering how to right-size each collection, the presence of older print materials versus online accessibility took precedence. By eliminating unnecessary

print materials, each librarian has had the opportunity to discover overlap in print vs electronic access, alleviate their spaces of underutilized resources and consider modernization of their physical spaces.

Description:
First, the Ascension librarians reviewed their print book and journal collections. Titles that are available in e-book format, journals that overlapped multiple years via an online platform/database, or are recently updated with new editions, were singled out to be replaced. Next, those volumes designated for recycling were appropriately marked and deleted from the electronic catalog. For some librarians, the de-accessed books have been made available for sale. During this time, librarians, or other leadership members, send updates and plans for future use of the space in the form of proposals. By recycling the leftover print materials, either by pulping for new paper products or using the energy to create electricity, the program supports Ascension’s environmental sustainability program. Evaluation and success are measured in funds from book sales, tonnage from recycling and the innovations requested for future renovations and/or improvements to online access managed by a remote librarian.

Conclusion:
To date, the national project is ongoing. Currently we have recycled 42 tons of books and transitioned 4 libraries to be completely online with librarians working remotely, and 3 now operating in hybrid form. There are still 4 Ascension libraries to go through this project.

A Remote Approach to Rebuilding Relationships with the College of Pharmacy

Track(s): Professionalism & Leadership

Dana Thimons, Health Sciences Librarian, Xavier University of Louisiana

Background:
An academic library facing numerous challenges, including recruiting during the COVID-19 pandemic, launched a program two years ago employing a group of part-time, remote librarians to provide library services and improve processes. The remote librarians were assigned liaison roles based upon their expertise in the university’s curriculum subject areas. They work collaboratively with each other and the on-site, full-time librarians and staff to provide services such as reference, instruction, outreach, and collection development. Since the inception of the program, the liaison to the College of Pharmacy (COP) has been a remote librarian working 10-15 hours per week. This librarian was hired at the start of the spring 2024 semester in a full-time, remote capacity.

Description:
There were two key factors leading to the creation of this initiative:

1. Because the COP did not have a dedicated library liaison for several years, they had limited engagement with the library.
2. While many academic libraries are offering virtual services and hybrid positions, full-time liaison support appears to remain novel.

The goal of the initiative is to re-establish relationships and promote the library within the College of Pharmacy.

While leveraging previously established relationships to cultivate champions for the library, the librarian began the initiative by communicating with faculty to spread awareness of library services.

The librarian is creating new guides and tutorials and planning pharmacy-specific virtual workshops. They will attend the August 2024 P1 student orientation in-person.

To evaluate the initiative, the librarian gathered baseline data of how the COP is utilizing the library. This included the number of instruction sessions provided to COP classes and purchase requests submitted by COP faculty during the 2022-2023 academic year. Reference analytics were analyzed for transactions involving the COP. The librarian also created a qualitative survey to measure COP faculty perceptions of the library. The survey will be distributed in the spring 2024 semester and again in spring 2025.

Conclusion:
University/hospital mergers, declining budgets, and recruitment challenges are issues faced by many health science libraries that can lead to limited engagement with users. Lessons learned from this initiative are relevant to health sciences libraries working to reengage and rebuild those relationships.

The librarian will share successes and roadblocks faced. Results of the first qualitative survey measuring COP faculty awareness and perceptions of library services will be provided.

Rethinking Reference Statistics: Optimizing Inputs for Better Outputs

Track(s): Information Management, Information Services, Professionalism & Leadership

Jessica Petrey, Associate Director, Clinical Services, University of Louisville

David King, Web & Technology Librarian, University of Louisville Kornhauser Health Sciences Library

Background:
Libraries rely on reference statistics to assess their services, track progress towards strategic initiatives, better plan to meet the evolving needs of users, and provide quantitative evidence of value when communicating with administration and stakeholders.

Meaningful interpretation of statistics for any of these purposes is dependent on complete, consistent, and accurate data entry by library personnel; recognizing the integrity of our own reference statistics had been compromised in all three areas, our library sought to improve our data collection process. We present here the process by which we assessed our activity tracking and reporting needs, changes we implemented as a result, and lessons learned that may benefit other libraries interested in improving collection and stewardship of their own data.

Description:
Because incomplete or delayed entry of librarians’ activities and lack of consistency within the entered data were known issues, feedback was collected from personnel who provide reference or instruction services to identify specific pain points hindering their ability to enter transactions and to understand variations in form usage. We manually reviewed the collection form and conducted a thorough analysis of the existing dataset to identify areas of concern for potential data entry errors and improve alignment with reporting needs.

Obsolete data points were removed from menus. Fields and field content were carefully reviewed for redundancy and were merged or renamed for clarity as appropriate. Settings were adjusted to ensure fields

relevant to all activities were required while optional fields were used sparingly to collect information applicable only for certain transactions. Text or numeric entry fields were replaced with selectable menus to minimize risk of typographical errors. Categorization was updated to align with reporting requirements of administration, external organizations, and other stakeholders. A comprehensive guidance document was developed to facilitate training and standardize understanding of data fields and input options, and a group session was scheduled to orient the team to the updated process.

Conclusion:
Preliminary results indicate reduced data entry errors and more accurate generated reports. Improved usability reduced time required to track activities, and aligning with reporting needs facilitated sharing of exported data with minimal cleanup. Librarians and staff reported increased satisfaction with usability, specifically citing the elimination of redundant or unused variables, decreased overall length, less scrolling with streamlined menus, more logical field organization and wording, and less ambiguity between input options.

Further day-to-day use of the form will allow us to further assess and adjust the form, providing a more accurate overall appraisal. We anticipate that the improved form will facilitate higher quality data collection while requiring less time away from our librarians’ primary responsibilities.

Searching Like a Boss: #PubMed and TikTok

Track(s): Education, Information Management

Pam Pierce, Digital Scholarship and Repository Librarian, OHSU Library

Background:
In fall 2023, the XX University Library conducted the first Pizza and PubMed session. Using #PubMed on TikTok, this highly participatory workshop/pizza party explored how the database is used and misused, sharing tips to save time, as well as support patient communication. This lightning talk will explain how the workshop was created and give others the chance to adapt it for their own institutions. Workshops such as this make PubMed approachable and may possibly lead to it being used more often within the clinical setting (Nicholson J, Kalet A, van der Vleuten C, de Bruin A. Understanding medical student evidence-based medicine information seeking in an authentic clinical simulation. Journal of the Medical Library Association 2020 Apr;108(2):219-28. DOI: 10.5195/jmla.2020.875.)

Description:

1. Select date for Pizza and PubMed workshop when students will most likely attend
2. Market workshop in conjunction with student groups
3. Select and screen TikTok videos, with an emphasis on PubMed techniques and how patients communicate
4. Connect videos to concrete PubMed techniques that will save students time

Conclusion:

1. Build a collective understanding of how TikTok videos can improve PubMed learning outcomes

2. Consider the importance of approachability when teaching PubMed

Self-Reflections on Our Literature Search: Creation and Utility of a Literature Search Best Practices Guide for librarians

Track(s): Education, Information Services

Heidi Reis, Liaison Librarian to the Brody School of Medicine, East Carolina University

Soph Myers-Kelley, Laupus Library, East Carolina University

Amanda Haberstroh, Liaison Librarian to the School of Nursing, East Carolina University

Background:
Literature search best practices and expectations are often unspoken or assumed by librarians. Between individual librarians, the rigor, preferred approach, and skillsets used for literature searching vary widely. Colleagues at [Library Name] worked together to create universal expectations for our department when working with literature search projects via group discussions and writing sessions. The result was an internal document that can now be used to train librarians and ensure consistency in literature searching for patrons. During this lightning talk, we (contributors to the guideline) reflect on the potential impact the creation of this guide has on our practice through an internal Qualtrics survey and self-reflection. Furthermore, we hope the description of our process to create the guide inspires other librarians.

Description:
The creation and evaluation of the Literature Search Best Practices Guide occurred in three main stages. In stage one, members of the department agreed that a unified understanding of how best to conduct literature searches for patrons was needed. Then, librarians sought consensus on nuanced issues that impact literature search services, like technical, procedural, relational, and ethical considerations for literature searches. The department resolved these issues in a series of group discussions over a period of five months. In addition, a survey of the departmental librarians to identify common logistical and clarifying questions when replying to lit search requests was used to compile a list of useful key questions. As part of stage two, members of the department volunteered to write sections of the best practices guide, with the sections arising from the discussion series, list of key questions, and justification for developing the guide. For stage three, an evaluation of the utility of the guide was conducted. The authors sent out a Qualtrics survey with self-reflection questions aimed at seeing if/how the creation of the guide impacted literature search practices of colleagues.

Conclusion:
We collected five unique responses to the Qualtrics survey. The responses primarily revealed that contributors to the guide felt reflecting upon their own practices was both professionally beneficial and insulated against feelings of inadequacy or imposter syndrome. Creating the guide had an added benefit of revealing colleagues' practices, allowing librarians of all levels to hone their skills. Developing a best practices guide has the potential to provide a starting point and guardrails for new librarians as they are onboarded into their roles. We have completed the initial development of this guide and shared it on Open Science Framework (https://osf.io/be29q/). Future steps will include an evaluation of the usefulness of the guide in practice, which will be undertaken in June 2024.

Spotlighting PUMP Act Resources & Breast Practices for Incorporating Lactation Support into a Medical Library Workplace Culture

Track(s): Professionalism & Leadership

Tiffany Follin, Medical Liaison & Outreach Librarian, Florida Atlantic University

Background:
According to ABetterBalance.org, 60% of women stop breastfeeding before they plan to, 50% had employment plans impact their baby-feeding decisions, and only 40% had access to both sufficient time and private space to express milk while at work. The presenter found a lack of information provided by her academic institution when preparing to go on FMLA leave and upon her return. With the encouragement of her department head and a determination to make it to one year, she gathered resources to plot the best work plan to productively pump and meet work duties. The objective of this talk is to share with the medical library community the resources learned along the way and implemented into the department’s culture.

Description:
The department’s small size and existing collaborative culture organically enabled a supportive approach to department activities and goals. Two national goals of the United States’ Healthy People 2030 initiative to increase the proportion of infants that are breastfed exclusively through age 6 months, and the proportion of infants breastfed at 1 year of age. With no local guidance from the academic institution to meet this national goal, other than a labor law poster on the HR website, implementation and follow-through was a very individualized process of trial and error. The presenter utilized her private office and a mini fridge to meet basic pumping needs. Informal evaluation was then held daily at the employee level and sought to observe and react to such performance measurements as milk output from sessions vs daily meeting and project deadlines. There were also daily evaluations that uncovered gaps in implementation of pumping accommodations, such as the office’s side window privacy screen not being long enough to prevent someone peeking under. Management-level informal evaluation evolved to become largely support-based and used a reactive model based on weekly department meetings, where time conflicts and project timelines were adjusted to meet employee pump break requirements.

Conclusion:
The author’s immediate initiative was to meet a facet of working parenthood not acknowledged and talked about at the institution. Secondary goals were to gather resources that could later be shared with colleagues on campus as well as in the health sciences librarian field (which resulted in an accepted talk at a regional medical library conference). Future plans include creating a formal checklist and reaching out to the institution’s HR department advocating inclusion with all FMLA request approvals related to the birth of a child. Although this is a larger social issue spotlighted within a medical library setting, policies and interventions ultimately play out locally and to succeed nationally, we need to succeed within our local spheres of influence.

Strategic Planning for Hospital Libraries: A Case for Investing in Planning Efforts

Track(s): Clinical Support, Information Management, Professionalism & Leadership

Catherine Soehner, Executive Director, University of Utah

David Coleman, Medical Librarian/Informationist, Hawai‘i Pacific Health

Background:
According to the Medical Library Association Hospital Libraries Caucus Standards Task Force (2022), standards for hospital library practice includes strategic planning1. A strategic plan generally serves two aims: 1) an external purpose that positions the library in its overall environment, and 2) an internal purpose that gives focus to the work and resonates with members of the library. Additionally, areas of weakness can be identified instead of ignored and areas of strength can be built upon. Since hospital libraries are often staffed by just one or two people, making time for strategic planning may seem impossible and unproductive. However, many people2 have indicated that the benefits significantly outweigh the challenges particularly when it comes to demonstrating the value of libraries.

Description:
One hospital librarian consistently conducted strategic planning, creating a new plan each year. The plan consisted of a set of documents encompassing a mission statement, vision statement, and strategic directions broken down into goals, with each goal linked to the organizational value that it supported. Within those goals are objectives that are the focus of each fiscal year for three years. Frequent review of these documents, collection of supporting metrics, and regular communication with administration kept library services relevant to and in alignment with organizational values. An annual report with a cost-benefit analysis based on costs of major databases and subscriptions compared to usage statistics became a pivotal communication tool. This analysis provided a clear graphic for the cost of library operations in the context of service provided to the organization. As a result, during the hospital budgeting process, the library became a “need to have” item as opposed to a “nice to have” line item. In addition, this analysis became a useful tool for subscription renewal negotiations and provided the basis for negotiation with vendors about possible multi-year subscription renewal discounts. 1. J Med Libr Assoc. 2022 Oct; 110(4):399 2. J Med Libr Assoc. 2002 Jan;90(1):86-92.

Conclusion:
Strategic planning is an important leadership tool and one that is easy to put off or ignore, especially when staffing and time are limited. Unexpected and powerful benefits of developing a strategic plan are often found in the process itself, which can lead to new ideas and unexplored strengths. With a focus on serving the overall institution, the library can position itself to contribute significantly to the quality of healthcare delivered and to the improved patient experience. A robust strategic plan, developed in partnership with key stakeholders assures the library’s continued ability to expand access, enhance services, and strengthen capacity in ways that keep it relevant and essential to organizational success.

Strategies for Successful Onboarding in Health Sciences Librarianship: A New Librarian’s Perspective

Track(s): Professionalism & Leadership

Loida Pan, Research Impact Librarian, NYU Langone Health

Background:
The onboarding process poses unique challenges for new librarians, particularly those starting their first position. This talk aims to provide insights from the perspective of a new librarian, offering practical suggestions for librarians going through the onboarding process.

Description:
In this presentation, I will discuss key insights and strategies garnered from my onboarding experience as a new librarian to the health sciences field. These revolve around cultivating relationships with colleagues in

and outside your institution, familiarizing yourself with trends and tools relevant to your field, as well as pursuing professional development opportunities. Providing new librarians with strategies for a successful onboarding experience can help promote stronger collaboration between peers, assist in defining expectations, and help new librarians effectively meet patron needs.

Conclusion:
These insights can be used to enhance the onboarding process for new librarians in health sciences. Ensuring a successful onboarding experience for new librarians empowers them to be able to best serve the needs of patrons and further the goals of the institution.

Stronger When Supported by Data: Clinical Impact of a Librarian Mediated Literature Review Service

Track(s): Clinical Support

Heather Martin, Director of Medical Libraries, Providence

Carrie Grinstead, Providence, Providence Library Services

Danielle Linden, Program Manager, Providence St. Joseph Hospital

Background:
Our library team has not had a systematic way to gather feedback from our users regarding our literature review service. Previous studies have documented the importance of library services in hospitals, and the impact librarians make on quality patient care. As a small team serving a large health system, we need to carefully evaluate the effects of our work, share successes, and gather information that will help us improve. We conducted this quality improvement project to understand whether a professional literature review service impacts patient care and saves clinicians’ time in a large integrated health system.

Description:
We adapted, with permission, the survey instrument and methods of a recent quality improvement project authored by Siemensma, et al (2021) in the Journal of Hospital Librarianship.

Our quality improvement project received IRB approval in late 2022. Throughout 2023, we sent the adapted survey to all employees who requested literature searches. When a librarian completed a search and sent results to the requestor by email, they noted the requestor’s email address in a REDCap form. Each Friday we ran a report of all email addresses that received literature searches two weeks prior. Using a generic email address associated with our private SharePoint site we sent an email with instructions and the survey link to requestors. All requestors’ email addresses were in the BCC field.

Because the purpose and results of each search were different, employees received an email with the survey link each time they requested a new search. We used Excel to compile summary statistics, documenting: survey response rate, respondents’ primary roles, impact on practice, perceived quality of the literature review, and time saved.

Conclusion:
): Data collection closed in January, and we will begin analyzing results in February 2024. We hope to learn how often our search services lead to modification or confirmation of current clinical practice. We’ll also be able to estimate the amount of time our users save when they ask us for literature searches, rather than attempting to find the information themselves. Space for free text in our survey allowed users to provide anonymous feedback on our services. Direct feedback from Library patrons will be used to inform leadership and make a case for growing our services.

Supporting Health Equity Initiatives: Creating an Interactive eResource to Support Health Disparities Competency

Track(s): Education, Information Services, Innovation & Research Practice

Beth Reeder, Assistant Director of Operations & Public Services, University of Kentucky

Cayla Robinson, University of Kentucky, University of Kentucky

Lauren Robinson, Associate Director of Research Services, University of Kentucky Medical Center Library

Rebecca Morgan, Health Sciences Library, University of Kentucky

Stephanie Henderson, Association Director of Outreach and Engagement, University of Kentucky

Background:
Purpose: This lightning talk describes a novel method for teaching users how to better search, find, and interact with literature on health disparities while highlighting resources from the library collection.

Setting: A R1 health sciences library serving medicine, nursing, pharmacy, health sciences, public health, dentistry, agriculture, food, and environment.

Description:
In 2021, our library purchased a collection of Equity, Diversity, and Inclusion (EDI) eBooks. Instead of folding them into our collection, we built a resource that highlights those eBooks alongside newly created tutorials. The purpose of this resource is to provide in-depth, asynchronous education on vulnerable population and health disparity searching to promote an environment of inclusivity and equity. The three tutorials cover topics such as searching nuanced literature, defining vulnerable populations, and researching marginalized peoples. In addition, we developed three interactive modules embedded within the resource to teach users how to be more inclusive searchers. The modules highlight: (1) Appalachian Populations, (2) LGBTQIA+ Populations, and (3) Racial and Ethnic Minorities. These modules have specific learning objectives that help users better understand the problems associated with searching population-based literature and provide them tools to become better and more inclusive searchers. In this presentation the authors will discuss the resource itself, how we conducted sensitivity reading, our plans for future assessment/expansion, and creating open access materials for reuse.

Conclusion:
Development of the project is complete. Assessment and expansion are the next steps. The authors will discuss how they conducted sensitivity reading, the progress of assessment, and future directions. Current outreach includes working with EDI (DEI) committees within various medical campus Colleges and departments, presenting the resource at curriculum committee meetings in order to integrate the tool into instruction, and showcasing the resource across the University campus.

A Systematic Approach to Review Services

Track(s): Information Services

Emme Lopez, Assistant University Librarian, Texas A&M University

Margaret Foster, Evidence Review & Scholarly Dissemination Librarian, Texas A&M University

Background:
The Medical Sciences Library at Texas A&M University has a long history of providing support for all types of evidence reviews. However, that support had never been codified into a defined service. We plan to discuss the process we used to structure and launch a review service. Topics include the backend and reporting processes, statistics and storytelling, and how we approached the unexpected and unknown.

Description:
What seemed like a straightforward project became more complex due to the shared nature of the service across a large library system and staffing changes that increased scope and capacity more quickly than expected. Our first goal was to clearly delineate the difference between our fee-based academic center dedicated to evidence review and the review service freely available to our client groups. We also created an organizational structure with a dedicated librarian and library assistant to improve efficiency. We developed a process and workflow map identifying areas for automation, places for built-in templates, and checkpoints in the process. This effectively turned the backend process into a teaching tool for newer reviews librarians. Operational considerations around staffing and web presence led to a staged implementation. We used the software we had available through institutional subscription, including Teams for workspaces and archives, LibAnswers for ticketing, and Covidence for production. We gather statistics on time spent, client types, review types, and how many requests we refer or turn away. We also gather open-ended feedback from users of the service. Since this is our first year, it is likely that the focus of evaluation will shift as we become more established.

Conclusion:
We look to see if we are getting repeat customers and how many of our reviews go all the way to publication.

Topics in Health Sciences Information: Developing an Undergraduate Curriculum Using Health Literacy Frameworks

Track(s): Education

Colleen Foy, Research & Instruction Librarian for the Sciences, Wake Forest University

Background:
1. At the end of this talk, attendees will have been exposed to theoretical frameworks that can support health literacy-based course development.

2. Attendees will have access to an outline for a course that will cultivate a foundation for lifelong learning in the health sciences for their student or patron populations.

3. Attendees can take first steps in designing an activity or assignment that will provoke student reflection and acknowledgment of historical or current health science topics.

Description:
This health literacy (HL) course was tangentially developed with an MLA RTI project. During this research, Don Nutbeam’s HL Framework and the Research Triangle Institute’s HL Conceptual Framework were utilized to establish educational and instructional techniques. The combination of these theories identify the primary hierarchical constructs of HL development as well as acknowledge external factors that influence

the refinement and utilization of HL skills. What became evident was the lack of undergraduate course offerings at the principal investigator’s academic institution that cover these concepts from an information literacy standpoint. How are students processing health related messages to inform decisions critical to their personal and professional development? How can health sciences librarians engage students to simultaneously embrace and combat the current information landscape? What transpired was the Topics in Health Science Information course curriculum which contains modules that include but are not limited to: the history of health sciences, evidence based medicine and academic practices, mis-, dis-, and malinformation, health equity, and health insurance 101. Pre/post course surveys collect student confidence, understanding, and awareness responses relative to HL skills, attitudes, and behaviors; outcome information can be found in the Program Conclusion entry.

Conclusion:
Topics in Health Science Information is a 1.5-hour credit bearing course and was offered in the Fall of 2023; 17 students attended and were invited to take pre- and post-course surveys. One additional set of assessment data will be collected during the Spring 2024 section and by the time of this presentation. With three Likert-style questions and a 76.5% response rate, surveys reported a 64% increase in confidence, a 72% increase in understanding, and a 65% increase in awareness of issues related to health science information handling behaviors and attitudes among students. Additionally, post-course surveys included an open-ended text-based question inquiring what topics respondents wished were included in discussions to allow for continued course development.

Unionization at a Health Sciences Library

Track(s): Professionalism & Leadership

Caitlin Maloy, University of Washington

Leah DeSantis, Public Health and Research Services Librarian, University of Washington Health Sciences Library

Leslie Gascon, Collection & Research Services Librarian, University of Washington Health Sciences Library

Background:
Library staff across a large academic institution recognized the need to increase diversity in hiring and retention within the libraries by disrupting stagnated wages and fostering a healthy work environment across the institution's libraries. This lightning talk briefly details the process and timeline of librarians and professional staff successfully unionizing to achieve these goals, including lessons learned and the impact of unionizing on the health sciences library.

Description:
An analysis of wages and benefits revealed that our institution was one of the lowest paying among peer institutions when adjusted for cost of living and inflation. Staff investigated union organizations and quietly organized for a year before publicly announcing the intent to unionize in 2020. The year that followed consisted of collective actions and negotiations to demonstrate a united and shared goal for a better workplace, and resulted in the institution recognizing organizing staff as a bargaining unit in 2021.

After an overnight bargaining session before a planned indefinite strike to start that morning, the union and employer agreed to a contract on January 25th, 2023. Sixteen months of bargaining produced gains on equity and transparency language, salary minimums of librarians, salary percentage raises for professional staff, an academic freedom clause, a grievance and mediation process, and more. Employees are still evaluating whether the contract suits all employee needs and are planning for the next bargaining period in

2025-26. The employer appears to be adjusting to the situation and using the contract to find mutually agreeable compromises.

Conclusion:
While unions are growing across the United States as a method to improve workplaces and reduce turnover, many employers see the effort as a zero-sum game. This unionization resulted in immediate wins for the employees and potential future gains for the employer through a happier and more stable workforce. Our union and contract both have overwhelming support from library staff, and our improved salary structure and working conditions ensure greater satisfaction among current library workers in addition to making our institution a more attractive workplace for potential employees. In conclusion, unionizing was a long and complicated process but it was worth it.

Unlocking Boundless Horizons: Cunningham Memorial International Fellowship Experiences

Track(s): Health Equity & Global Health

Kadimo Khutsafalo, Librarian, University of Botswana

Background:
Opportunities to undertake international fellowships and experience for health science librarians are rare to many African Librarians, yet the Medical Library Association (MLA) administers a three-week fellowship for medical librarians from countries outside the U.S. and Canada which provides for attendance at the MLA Annual Meeting, observation and supervised work in a number of medical libraries in the U.S. or Canada. As a complementary, the fellowship grants access to MLA sponsored Continuing Education courses a before the fellowship is undertaken. The fellowship serves as catalyst for cross-cultural collaboration, knowledge exchange personal growth and outstanding professional opportunity for a health science librarian to meet other dynamic health science librarians and be introduced to the many activities of U.S. libraries.

Description:
This lightning talk aims to share experiences of medical librarians from Africa (Zambia and Botswana) who have been part of this honoured fellowship. The fellows will share their unique experiences perspectives, challenges, and triumphs, providing insights into the profound impact of the Cunningham Memorial International Fellowship program.

The fellows will further navigate through the various facets of the Cunningham Memorial International Fellowship, highlighting the global connections, the academic and professional insights gained, and the invaluable cultural immersion experienced. The fellowship participants share how their journey has not only expanded their intellectual prospects but has also enlarged their international understanding narrative.

Conclusion:
The talk will conclude with a reflection on the overall impact of the Cunningham Fellowship in their academic and professional journey with an aim to inspire, inform and celebrate this extraordinary diversity of knowledge.

Using a Practice Search Set to Teach Students Advanced Searching for Integrative Reviews

Track(s): Education

Lynn Warner, Associate Librarian, University of Cincinnati

Background:
Students in a PhD in Nursing Program must conduct an integrative review as part of their coursework. Embedded in the course, the health sciences librarian is asked to instruct students how to construct an exhaustive search string to collect evidence. As this is the students' introduction to conducting an integrative review and comprehensive searches, they can feel overwhelmed and unsure of the process.

Description:
After an instruction session on the search building process, the librarian asked the students to reproduce a search using a published integrative review. This gave the students hands-on experience creating a search string, along with the ability to check their work against the set of references used in the published review. Follow up sessions with the students allowed for feedback and further instruction before being asked to create searches for their own topics. This learning activity was drawn on from practice sets developed by a group of Informationists at a separate institution, created to help librarians apply and refine systematic search skills.

Conclusion:
Initial feedback from the students indicates that the practical experience building a search based on a published integrative review increases the students' confidence when tasked with creating searches for their unique topics. This is a Spring 2024 course, and the activity will be fully assessed at the course's conclusion.

Using Find & Replace To Speed Up Translation

Track(s): Information Services

Eden Kinzel, Liaison & Education Librarian, University of Toronto

Background:
Evidence synthesis projects require librarians to search a variety of databases, most often somewhere from three to seven, but sometimes the number can be even higher! Almost always, the chosen databases will be hosted on different platforms thus requiring translation of the search syntax in addition to subject headings (when applicable). The translation process can be time consuming as well as quite tedious and provide opportunities for mistakes. There are a variety of tools a librarian can use to aid in translation that can minimize the time required, the tediousness, and risk of errors, one of them being a word processing program's Find & Replace tool.

Description:
During their time at library school, the presenter took a course in web design and in their first job after graduating, they participated in a large long-term data analysis project. Both of these experiences (plus inspiration from other colleagues in the profession) led them to explore a variety of technologies, both advanced and simple, that may be able to be adapted for library use. Find & Replace can be used to translate field codes or proximity operators, add or remove quotation marks, adjust subject heading syntax

(thinking of MEDLINE to Cochrane adjustments when not on the same platform), and more! This lightning talk will consist of quick demos of each of the aforementioned techniques so those in the audience can get a glimpse of truly how quick translation can be!

Conclusion:
Using Find & Replace during evidence synthesis translations has permitted the presenter to more quickly and accurately engage in database translation. This is not meant to be an endorsement of these tricks as the best or only way translation should occur; there are a variety of tools and methods that work well for the variety of different learning/workflow styles of expert searchers. This can be a stepping stone into more thorough examination of automation of translation or alternatively, a perfectly sufficient stopping point. The presenter hopes this talk will encourage others to share their translation tips, so we’re all able to learn from each other!

What To Do When the SR Team is At Capacity: Grab a Number, Get in Line, and Learn SR Methodology

Track(s): Information Services

Lydia Howes, Assistant Librarian, Research and Education, University of Utah

Tallie Casucci, Associate Librarian, University of Utah

Background:
In 2023 our library's Evidence Review Service (ERS) received a record number of requests for collaboration. However, the requests quickly surpassed our team's capacity to meet demand- 31 projects out of 63 requests were waitlisted for six weeks or more. By the end of December, 16 requests remained on the waitlist. Efforts to empower teams to use their time while on the waitlist to self-learn proper methodology met with mixed results; most teams seemingly did not review the recommended materials. The ERS needed to make changes to the service model to ensure we meet with interested parties promptly and effectively communicate: 1) what the ERS will provide for collaborating teams, and 2) the pre-requisite expectations of review team members.

Description:
Early in 2024, the ERS met to discuss workload challenges and determine the next steps. We decided to modify our request form to include language concerning the ERS' scope of participation and requirements for collaboration. We will maintain our mission of educating teams on review methodology and producing high-quality reviews and search strategies, but will slightly rework the timeline and workflow. Previously, projects were activated after the initial meeting. Now, we've committed to meeting with teams within 7-10 days of receiving requests. During initial meetings, we will discuss the research questions, determine the appropriate type of review, and outline next steps. We will require the completion of reading/task lists designed to ensure teams have a basic understanding of proper review methodology and developing an a priori protocol. The PI and their mentor (if appropriate) will complete the learning checklist before receiving the protocol template and invitation to the ERS workspace. Projects will be activated after teams have relayed the completion of required tasks and an ERS team member has the capacity for a new project. In the revised workflow, wait times will be measured from the time teams complete the learning checklist to project activation .

Conclusion:
Results are not yet available, but in the lightning talk we plan to share the modified request form and required reading lists, ERS statistics for 2023 and the first quarter of 2024, and any other observed outcomes.

COLLECTION DEVELOPMENT SYMPOSIUM ABSTRACTS

We have sorted collection development symposium presentation abstracts in this section by title in alphabetical order.

AI, Machine Learning, and Collections: New Approaches and Tools for Description and Discovery

Moderator:
Michelle Kraft, MLS, AHIP, FMLA, is the Director of Library Services for the Cleveland Clinic Health System. Michelle is a member of a team of Cleveland Clinic librarians, cardiovascular researchers, and IBM staff who are experimenting with AI applications, particularly as they relate to the discovery of research. She also experiments with AI on her own to help users with their AI experiments and to find ways medical librarians can leverage AI for their needs.

Panelists:
Itai Veltzman is the Director of Product Management for Ex Libris/Clarivate. With a focus on data and AI, he leads metadata and resources management for a Unified Library SAAS. He has driven Linked Open Data, AI-based Metadata creation, and other major initiatives and has conducted AI pilots for title matching involving normalization of fields and model selection.

Judson Dunham is Senior Director of Product Management, ScienceDirect, in which capacity he leads a team of product managers responsible for developing and maintaining ScienceDirect, Elsevier’s flagship full text search and discovery platform. He has been in product management at Elsevier for over 15 years, working in and around research discovery and workflow products including ScienceDirect, Scopus and Mendeley.

Philip E. Schreur, PhD, MLIS, is the Deputy University Librarian at Stanford University, in which capacity he oversees daily operations and linked-data project development for the Stanford University Libraries (SUL). He has worked in a variety of areas involving linked data, including using linked data to manage identity across traditional and digital resources and integrating linked data from multiple sources.

Description:
Artificial Intelligence (AI) and Machine Learning (ML) are set to revolutionize library catalogs and vendor platforms. Knowledge workers need to grasp the impacts, benefits, and challenges of integrating these technologies into their information ecosystems and workflows.

Our moderated panel will delve into the theory and practice of AI and machine learning, exploring how they influence the discovery and description of resources and library collections. Among the topics the session will address are: the roles of publishers, vendors, libraries, and human metadata creators in engaging with and leveraging these technologies; the potential of AI tools to broaden library services and expertise; and

key questions those working in collection development and management will have, such as the impact on library collections' discovery processes and how licensing and copyright considerations may inform the training sets for AI tools.

Join us for an insightful exploration of AI and machine learning’s role in shaping the future of description and discovery.

Collective Collecting and the Challenge of Preservation

Moderator:

Melissa De Santis, MLIS, AHIP, is the Director of the Strauss Health Sciences Library, University of Colorado Anschutz Medical Campus. Melissa has worked as a librarian for over 25 years and gained her experience in health sciences library collective collecting and preservation as a member of the Western Regional Storage Trust (WEST) Executive Committee and through work with the Colorado Alliance’s Shared Print Trust and the Rosemont Shared Print Alliance.

Presenters:

Vida Damijonaitis is the Director of Worldwide Sales for the American Medical Association, where she is responsible for all content sales and sales development efforts, including licensing, aggregation, circulation, permissions and new products, for the JAMA Network journals.

Alison Wohlers is the Shared Print Program Manager at the California Digital Library, in which capacity she leads collaborative print management efforts in the University of California Libraries system and the Western Regional Storage Trust, a regional collaboration of over 50 academic and research libraries. Alison is also involved in the leadership of national shared print federations and initiatives.

Description:
Large scale collaborative collection development, management, and preservation is becoming a necessity as environmental, organizational, and informational challenges and opportunities transform libraries. These changes make it imperative that libraries and publishers engage in meaningful discussions about preservation and their complementary roles in maintaining access to print and electronic content and preserving it for the future. Collaborative collection development and preservation can be done at all sizes of institutions, and finding and nurturing partnerships and networks in your collection ecosystem is essential.

Speakers will explore models of collective collecting for print and digital materials; common challenges, including preserving data and open access resources, shared decision-making among partner institutions, and space considerations; and the inherent fragility of providing access to and preserving born-digital materials. This moderated expert panel will empower librarians to understand and identify the potential role of collective collections and preservation in their circumstances. By working together, we will maintain the scholarly record for our current and future users.

Getting Started with Collection Development: An Interactive Workshop on Selecting Resources for Your Library Community

Presenters:

Basia Delawska-Elliott, MLIS, AHIP, is a medical librarian with Providence in Portland, OR. In her current position, she acts in an advisory capacity on collection development, evaluating new resources and suggesting purchases. In addition to her hospital library experience, Basia has worked in academic library acquisitions as a manager of approval plans, as an engagement librarian, supporting research, educational, and clinical needs, and as a member of collection development committees. Since 2020 Basia has been a part of the Nursing and Allied Health Resources and Services (NAHRS) working group updating the NAHRS Nursing Essential Resource List (NNERL), a tool for librarians supporting clinical and academic nursing.

Karen H. Gau, MLIS, is the Health Sciences Collections Librarian at Virginia Commonwealth University, where she evaluates resources for inclusion in VCU Libraries' health sciences collection. She created and maintains a list of collections-related DEIA projects and resources for MLA’s Collection Development Caucus and is co-editor of Health Sciences Collection Development: An Overview of Fundamental Knowledge and Practices (2nd ed.).

Description:
Have you been tasked with purchasing materials for your library but don’t have formal training or are new to collection development? This interactive workshop will get you started with developing the skills and knowledge you need to determine which books, journals, and databases to purchase for your library community. Participants will connect in small groups with other librarians from similar settings to brainstorm practical steps to take during the decision-making process.

Getting to Open: Everything You Need to Know about Crafting Open Access Agreements

Presenters:

Kathi Carlise Fountain, MSLS, is Head of Business Development for Oxford University Press (OUP), in which capacity she pursues collaborations with librarians, negotiates Read & Publish agreements, and serves as the "voice of the customer" within OUP. OUP has a growing number of active customized R&P agreements with libraries in a range of institutions, from large research universities to liberal arts colleges.

Susan K. Kendall, PhD, MS(LIS), is the Head of Collections Strategies and Copyright Librarian at the Michigan State University Libraries where she allocates and manages collections expenditures, negotiates with publishers, and develops a cohesive strategy for funding and supporting traditional and open access collections. She serves on the editorial board of Collections Management and is the editor of Health Sciences Collection Management for the Twenty First Century.

Description:
Open access publishing agreements are trending. You’ve decided to pursue one, maybe on your own or in collaboration with your main campus. Where should you start? How do you choose a publisher to approach? How do you assess the proposals and negotiate intelligently? With so many options, choices can be overwhelming.

This session offers a rare opportunity to learn from both the librarian and publisher sides how to effectively craft open access agreements. Our presenters, librarian, Susan Kendall, and publisher representative, Kathi Carlisle Fountain, are experienced negotiators of Read and Publish agreements. They will walk you through defining your institution’s priorities and needs, the kinds of agreements that are available, and concerns to

anticipate from publishers. You’ll leave the session with a checklist of points that you and a publisher representative can discuss and confidence that you can choose or collaboratively craft an agreement that makes sense for both parties.

DATA SYMPOSIUM ABSTRACTS

We have sorted data symposium presentation abstracts in this section by title in alphabetical order.

Data Allies: Building Institutional Support Networks

Moderator

Sally Gore, MS, MS LIS, is the manager of Research and Scholarly Communication Services for the Lamar Soutter Library, Chan Medical School, University of Massachusetts. In this role, she oversees the library's work with campus basic science and clinical researchers on campus, including expanding support and instruction in data services and leading all scholarly communication endeavors for the Library. Sally is also the Associate Editor of the Journal of eScience Librarianship.

Panelists:

Andrea H. Denton, MILS, is the Research & Data Services Manager at the Claude Moore Health Sciences Library, University of Virginia, in which capacity she designs and delivers data services to researchers at UVA’s academic medical center. She leads training on research data management best practices in acquisition, analysis, and sharing. She also partners with university research development staff to support the pursuit of external funding for large, collaborative projects.

Eugenia Opuda, MLS, MEd, is the Health and Human Services Librarian and an Associate Professor at the University of New Hampshire (UNH). She is the co-author of a chapter on integrating data management and information literacy skills in Teaching Research Data Management and author of the “Inclusion and Exclusion in Data” online training resource published in Sage Skills: Student Success.

Sara M. Samuel, MSI, is an Informationist at the Taubman Health Sciences Library, University of Michigan (U-M). She connects students, staff, faculty, researchers, and clinicians with the information they need to be successful. She is a founding member of the Research Data Stewardship Initiative (RDSI) working group at U-M, which aims to support all U-M researchers in managing and sharing research data. As part of RDSI, she helped refine U-M’s newly implemented Research Data Stewardship Policy and co-chairs the RDSI Community of Practice, which connects U-M community members that are interested in or actively supporting research data.

Description:
Developing partnerships with researchers can be particularly challenging. This session offers you a rare opportunity to learn about these partnerships from both the librarian and the researcher side.

This session will focus on research enterprise-librarian partnerships. Examples will highlight various campus partners and their role in supporting research. Presentations will discuss how the collaboration was initiated and evolved over time, what worked and what didn’t, and key takeaways you can use to expand your networks and develop future collaborations.

You’ll leave the session with knowledge and skills that will help you:

- identify campus partners that support research
- explain the role of campus partners in the research landscape
- recognize the needs of campus partners and how you can support those needs
- apply strategies for engaging with other campus entities who serve the research community

Data Equity: Exploring What It Is and Why It Matters

Presenters:

Nicole Contaxis, MLIS, MA, is a Data Services Librarian and the Head of Data Sharing and Metadata Management at the NYU Langone Health. She leads the NYU Data Catalog and other infrastructure projects and research that focus on open science, data sharing, data curation, and ethics. She is also a Content Expert for the NNLM National Center for Data Services.

Aileen Alfonso Duldulao, PhD, MSW, is the Data Equity Measurement Methodologist for the Race, Ethnicity, Language, Disability & Sexual Orientation Gender Identity (REALD & SOGI) Section, Equity & Inclusion Division, of the Oregon Health Authority. In this role, she co-leads data equity and data justice work with internal and external partners. She is also the data advisor for the Oregon Pacific Islander Coalition and is on the Board of Directors for the Filipino Bayanihan Center.

Description:
Have you been thinking of issues of power, bias, and discrimination in data collection, analysis, interpretation, and reporting? Data can empower practitioners, policymakers, and community members to make better, more informed decisions grounded in evidence, but they can also reinforce deficit narratives, biases, and other long-standing structural inequities when used inappropriately. Whether intentionally or unintentionally, data can be misused and misinterpreted, sometimes causing harm to communities already most marginalized.

In this session, you’ll be introduced to data equity in the context of data ethics. You’ll learn what is meant by data equity and ethics and the implications of not using equitable practices, and you’ll gain ideas on strategies for ensuring that data are meaningful, accessible, and actionable for all communities.

Data Instructors: Providing Data Literacy as Part of Information Literacy

Presenters:

Theresa Burress, MLS, is the Assistant Director of Research & Instruction at the University of South Florida, St. Petersburg, in which capacity she provides research services and instruction for natural sciences departments. She has authored a number of publications on data and data literacy and is particularly interested in topics at the intersection of information and data literacy.

Daria Orlowska, MSLIS, is a Data Librarian and Assistant Professor at Western Michigan University, in which capacity she advises on data management plans, creates data education resources, and provides consultations on all things data. In addition, she helps curate datasets from archival collections that serve as data literacy teaching tools for college undergraduates and K-12 students alike.

Ashley Rockwell, MA, is the Data Literacy & Learning Specialist for the Georgia State University Library Research Data Services Department. She leads the development, implementation, and assessment of the Get Data Ready! and Public Interest Data Literacy (PIDLit) initiatives. The former aims at building data literacy curriculum and programming, the latter on connecting students with community partners and applying data skills to address real-world problems.

Description:
As data is becoming increasingly important in the health and other sciences, it’s become clear that there is great value in integrating data literacy into information literacy instruction. In this session you’ll learn how data literacy and information literacy are related and how you can integrate teaching data literacy skills, including data discovery, data analysis, data visualization, and data preservation, into your information literacy instruction and programs.

Three data librarians will share their approach to data literacy instruction and their strategies for including data literacy, particularly as defined by the ACRL Framework for Information Literacy in Higher Education, in their information literacy instruction. You’ll leave the session with fresh ideas on how you can support all your patrons in understanding the relationship between data and information literacy.

Data Partners: Fostering Librarian-Researcher Partnerships

Panelists:

Levi Dolan, MLIS, is a data services librarian at the Ruth Lilly Medical Library at the Indiana University School of Medicine in Indianapolis, Indiana. His work involves project management, consultation, and instruction that addresses the full research data life cycle. As part of the Ruth Lilly Medical Library's Research Team, he contributes to data management, research metrics, and scholarly communications initiatives.His research partner, Amy Han, PhD, will appear via recording.

Ben Gerber, MD, MPH, is a Professor & Chief, Division of Health Informatics and Implementation Science, Department of Population and Quantitative Health Sciences at the UMass Chan Medical School. He is an internist, professor, researcher, and data scientist. He leads an NIH-funded clinical trial to evaluate clinical pharmacists, community health workers, and telehealth in supporting continuous glucose monitoring. He is a life-long software programmer and co-leads the R Café with Tess Grynoch. The R Café convenes a group of R users on a monthly basis to share skills, show off cool projects, and troubleshoot code.

Tess Grynoch, MLIS, is the Research Data & Scholarly Communications Librarian at the Lamar Soutter Library, University of Massachusetts Chan Medical School. She collaborates with faculty, students, researchers, fellow colleagues, and academic units to support scholarly communication programs, including library-based research data support services. Tess is the President-Elect of the Research Data Access and Preservation Association and a certified Carpentries instructor with NESCLiC. Her research partner is Ben Gerber.

Alisa Surkis, PhD, MLS, is the Deputy Director and Vice Chair for Research of the NYU Health Sciences Library, in which capacity she has led the development of data services. She is also the Director of the NNLM National Center for Data Services, which provides training and resources to develop capacity for data services in the health information community. Since 2018, Alisa has served as the Data Science Core Director for an NIH BRAIN Initiative collaborative grant in which capacity she works with researchers to facilitate FAIR data sharing and collaborates in the development of BrainSTEM, a tool for collection of structured, standardized experimental neuroscience metadata. Her research partner, Robert Froemke, PhD, will appear via recording.

Description:
Providing data management assistance to a researcher in a few brief consultations presents few challenges to a librarian. Working with a researcher in an extended partnership lasting months and years presents unique challenges that experience with brief consultations do not prepare you for.

In this session, three established librarian/researcher pairs in an extended partnership will show how you can work most effectively with a research partner. You’ll learn how to set expectations, identify the roles you will adopt, negotiate recognition for your contribution, communicate effectively, address challenges, and generally promote a satisfying collaboration. You’ll also learn strategies for engaging the research community in your institution.

You’ll leave this session with knowledge and guidance that can help you develop a reputation as the librarian researchers most want to work with and advance in your career.

LEADERSHIP & MANAGEMENT SYMPOSIUM ABSTRACTS

We have sorted leadership & management presentation abstracts in this section by title in alphabetical order.

Building Culture and Community in a Hybrid Workforce: A World Café Conversation

Café Host:

Mark Berendsen, MLIS, is Deputy Director of Galter Health Sciences Library & Learning Center, Northwestern University, Feinberg School of Medicine. In this role, he is responsible for personnel management of library faculty and staff. At MLA|SLA ‘23, Mark co-hosted a World Café session where participants explored ideas and gained a deeper understanding of leadership roles and opportunities for libraries in innovative domains, including AI, virtual reality, data management, data visualization, and global health & health equity.

Description:
Remote and hybrid work arrangements have become commonplace in libraries, hospitals, and health systems. With this dramatic shift toward new modes of work has come opportunities to reshape workplace culture and challenges for building and maintaining community in a hybrid workforce.

Do you want to discuss strategies your organization has implemented to build culture and community and learn about approaches your colleagues are taking? Participants in this highly interactive World Café conversation will connect with peers to discuss how they and their organizations have approached remote and hybrid work experiences, with a strong emphasis on building culture and community. If you’d like to share practices from your organization and benefit from the insights and innovations of your colleagues, this session is for you!

Inclusive Library Leadership: Empathy, Care, and Compassion as Leadership Strengths

Presenter:

Annie Bélanger, MLIS, is the Dean of University Libraries, Grand Valley State University. She is the lead for ACRL’s Inclusive Library Leadership subcommittee and the Board President for Disability Advocates of Kent County. Her research focuses on human-centered, inclusive leadership and skills development for library workforces. Being an accessibility and inclusion advocate and her experiences in academic, corporate, public libraries, and government settings define her career. Annie is Québéçoise and disabled.

Description:
In this interactive session for current and aspiring leaders and managers, you’ll learn how you can center empathy, care, and compassion in your formal and informal leadership roles. Annie Bélanger, a passionate and experienced disability and inclusivity advocate, will introduce you to inclusive library leadership, its definitions, and the traits and practices of inclusive leaders. You’ll begin to develop your own inclusive leadership approach and implement inclusive and equity-minded practices in your work. You’ll leave with tools and resources you can use to continue your growth as an inclusive leader.

Inside and Out: Supporting and Empowering Your Staff by Promoting Their Achievements

Presenter:

Meghan Kowalski, MSLS, is the Outreach and Reference Librarian at the University of the District of Columbia. She regularly presents and publishes on topics related to library leadership, marketing, and instruction. She authors the newsletters Content Prompt and The Weekly Wrap.

Description:
If you are a library leader or manager at any level, you know the value of a committed and enthusiastic staff. In this session, Meghan Kowalski, an experienced team manager and presenter on library management, will help you discover your staff members’ strengths and identify ways to promote their work and help them overcome self-doubt, imposter syndrome, and other hurdles to realizing their potential and bringing attention to their achievements. She will also help you see how promoting staff as individuals with unique skills benefits both your library as a whole and individual staff members.

You’ll learn primarily through a workshop in which you assess how you support and empower your staff and develop actionable steps to promote your staff members’ achievements, skills, and projects inside and outside of libraries.

Leading and Serving in an Unscripted World: Leadership, Librarianship, and Improv

Improv Facilitator:

Patrick Short, MFA, has been leading Applied Improvisation workshops for organizations since Apple requested a workshop in team building in 1989. He’s worked for over 800 organizations, including Intel, Nike, Government Agencies, NGOs, Not-for-Profits, and Educational Institutions. Since the onset of the pandemic, Patrick has taught clients in 42 countries via Zoom. He’s a certified professional in Applied Improvisation by the Applied Improvisation Network and a Certified Provider, Executive Level, by CSz Worldwide. Patrick co-wrote Jill & Patrick’s Small Book of Improv for Business and Yes, And Your Customer Service. In his spare time, he runs CSz Portland, an improv theater best known for ComedySportz, competitive improvisational comedy. In his actual spare time, he plays keyboards, writes songs, eats right, and always flosses.

Description:
Can the methods used by improv professionals in comedy and theater be applied to the day-to-day work of librarians and leaders? They can! Improv teaches cooperation, listening, brainstorming, communication, and other skills that are hugely beneficial in the library environment.

Doing improv can change how you feel about yourself, prepare you for new work roles, help you navigate difficult conversations and respond to new situations, and it can spark insights and creativity in your work as librarians and leaders.

Patrick Short, a highly experienced improv facilitator, will guide you in practicing improv techniques in a safe/no-pressure to perform atmosphere.

The improv session in the 2023 session was a joyful and fun hit. Don’t miss what will undoubtedly be the most fun and transformative session of the conference!
