# Supplementary material for: 124th Annual Conference, Medical Library Association, Inc., Portland, OR, May 18-21, 2024: 2024 Annual Business Meeting and Presidential Inaugural Address, June 5, 2024
Source: J Med Libr Assoc. 2025 Oct 23;113(4):E1–E28. doi: 10.5195/jmla.2025.2356 (PMC12604072; doi:10.5195/jmla.2025.2356)
Supplement: Supplementary file 2 — Appendix B: MLA '24 Poster Session Abstracts [file jmla-113-4-E1-s02.pdf]

Medical Library Association
MLA ’24 Hybrid Conference & Exhibits

Poster Abstracts

MLA ’24
Portland, OR
STRONGER TOGETHER
MAY 18-21 | #MLANET24

Abstracts for contributed content posters are reviewed by members of the Medical Library Association National Program Committee (NPC) and volunteer peer reviewers. Designated NPC members make the final selection of content to be presented at the annual conference.

Information is as provided by abstract submitters; if missing, known affiliations of presenters and authors may have been added.

RESEARCH POSTER ABSTRACTS

We have sorted content in this section by title in alphabetical order.

Adding Author Identifier ORCiD Metadata to PubMed Records – the Publisher’s Role

Track(s): Information Management

Konstantina Matsoukas, Research Informationist III, Memorial Sloan Kettering Cancer Center

Jeanine McSweeney, Associate Librarian for Scholarly Communications, Memorial Sloan Kettering Cancer Center

Robin O'Hanlon, Associate Librarian, User Services, Memorial Sloan Kettering Cancer Center

Objectives:
The aim of this project is to describe and quantify the extent to which authors at our institution are succeeding in having their ORCiD metadata reflected in the Author Identifier field of their papers’ PubMed citation records. We also seek to understand journal publishers’ role in this process, based on the degree of their adoption and support of ORCiD initiatives. The insights gained from this project will then be used to develop strategies focused on overcoming the existing barriers that are currently impeding scholarly authors from achieving this desired and beneficial outcome consistently for all their articles indexed in PubMed.

Methods:
Citation records added to NLM’s PubMed database in 2023 were searched by our institutional affiliation. Search results were screened using EndNote citation management software (Clarivate) and grouped into four categories: 1) All co-authors had their ORCiD (Open Researcher and Contributor ID) author identifier information added to the article’s PubMed record metadata, 2) Only one author’s ORCiD information had been added to the article’s PubMed record metadata, 3) Multiple co-authors (but not all) had their ORCiD information added to the article’s PubMed record metadata, and 4) No ORCiD author identifier information is present at all on the article’s PubMed record metadata. A list of unique journal titles was then generated from the PubMed search results and each journal’s publisher information was identified. Each unique publisher’s ORCiD membership was subsequently confirmed via the ORCiD.org website (https://orcid.org/members), their status as an ORCiD signatory was verified (https://info.orcid.org/requiring-orcid-in-publications/), and their ORCiD-related editorial policies were examined by searching each publisher’s website and/or author guidelines/instructions. This data reflecting each publisher’s degree of ORCiD support/adoption/participation was analyzed and compared to each publisher’s practices for distributing/passing on article ORCiD information to PubMed.

Results:
N/A

Conclusions:
N/A

AI Usage in Medical Libraries: An Exploratory Mixed-Methods Study

Track(s): Innovation & Research Practice

Ariah Long, Associate Fellow, National Library of Medicine

Objectives:
This research examines the rapid integration of Artificial Intelligence (AI) and Large Language Models (LLMs) into health sciences libraries. Conducted during an NLM associate fellowship experience, this small-scale exploratory study examines the evolving role of AI in medical libraries, identifying optimization opportunities and collaboration potential as well as trying out various research methodologies. The research addresses key questions about AI and LLM applications in health sciences libraries:

- What are the applications of AI and LLMs in medical libraries?
- How are medical libraries using and studying these technologies?
- How do these answers compare to those suggested by an AI tool?

Methods:
We used multiple methods to conduct an initial exploration of the intersection of AI and health sciences libraries. Ten medical librarians were contacted by direct email queries and responses to posts shared on MLA caucuses regarding their AI usage. These were posted on the Academic and the Technology in Education MLA caucuses on October 24th, 2023. The same questions were also submitted to the Research and the Libraries in Health Sciences Curriculums Caucus on November 8th, 2023.

The questions posed to the medical librarians and caucuses are as follows:

1. Does your library use any form of AI in your services?
2. What type(s) of services?

Next, we used ClaudeAI to find keywords and themes from five relevant articles related to AI in medical education and research and reviewed the most recent 3 years of MLA annual meeting programs for AI-related content.

Finally, we compared the 1) responses of librarians and caucuses, 2) keywords and themes identified by ClaudeAI from five relevant articles, and 3) opportunities for medical librarians and AI suggested by ChatGPT in a chat on August 24th, 2023 to identify overlap and opportunities for further exploration.

Results:
Seven medical librarians responded via email, and 6 responses were reviewed from MLA caucus postings.

ClaudeAI found 8 keywords and 5 themes that emerged from 5 uploaded articles related to ChatGPT and medical education. ClaudeAI compiled further descriptions for each of these themes; the full chat will be shown on the poster.

We discovered that, within the libraries we contacted, more libraries are exploring the use of AI than are actively using it.

We found that there was an increase of mentions of “artificial intelligence” and “ChatGPT” in MLA Annual Meeting programs from 2021-2023.

Conclusions:
This initial exploration of multiple methods for investigating the intersection of generative AI and LLMs within health sciences librarianship lays the groundwork for future, large-scale investigations into this topic. The various methods tested in this study demonstrate numerous avenues for robust surveying and taking a deeper look at relevant tools that can help students and librarians.

This project fits with MLA 24’s goal to pursue cutting-edge advancements in medical information, research, and technology. As librarians and researchers, AI not only has tools that we can use to our advantage, but we can ethically educate students on technological literacy and prepare them for the evolving landscape of healthcare and research methodology.

An Environmental Scan of Evidence Synthesis Projects Published by Yale University and Yale New Haven Hospital Authors: Preliminary Findings of Scoping Reviews

Track(s): Information Services, Innovation & Research Practice, Professionalism & Leadership

Alyssa Grimshaw, Clinical Research and Education Librarian, Yale University

Holly Grossetta Nardini, Associate Director, Yale University

Objectives:
Evidence synthesis is a widely utilized study design, with publication rates of systematic reviews, meta-analyses, scoping reviews and the like significantly increasing over the past two decades. While our library has a robust and accomplished evidence synthesis service, there are patrons who choose to publish without librarian involvement or who are still unaware of our services. To better understand this user behavior, we investigated systematic and scoping review publications that have been produced at our institution.

Methods:
An exhaustive search was performed across nineteen databases to identify papers associated with Yale University or Yale New Haven Hospital and using keywords or publication types related to scoping and systematic reviews. Final searches were executed on Jan. 25, 2024. To emphasize the influence of Yale University/Yale New Haven Hospital authors on the publication, only papers with a Yale University/Yale New Haven Hospital author listed as either the first or last author were included in this analysis. The analysis focused on extracting data regarding library participation in the review, the departments or schools involved, and fields related to compliance in reporting search strategies and methods.

Results:
The searches resulted in 22,991 citations. Preliminary results show that 1623 papers of 9287 citations meet the inclusion criteria with 3140 left to be screened. There was a substantial increase in systematic and scoping review publications over time, both with and without library involvement. Most of the published evidence synthesis reviews were from authors affiliated with the medical campus rather than the broader University. When reviewing the reporting of search-related fields, we found that most of reviews without librarian involvement are published without a reproducible search strategy and a search date older than six months at time of publication.

Conclusions:
The findings of this study will provide a better understanding of the evidence synthesis publishing landscape at Yale University/Yale New Haven Hospital. The findings could impact the library’s policies and procedures relating to librarian involvement in evidence synthesis projects, future staffing support, and creation of new training modules for patrons that explain proper reporting of searching and methodology in evidence

synthesis projects.

Assessing the Efficiency of Data Science Programs in Enhancing Big Data Analysis Skills among Health Libraries and Information Scientists.

Track(s): Information Management, Information Services, Innovation & Research Practice

Haruna Hussein, AHILA

Rajabu Simba, The University of Dodoma

Objectives:
To assess and evaluate the effectiveness of Data Science programs in improving the capacity and proficiency of professionals working in health libraries and Information Science. The study seeks to ascertain whether these programs adequately equip participants with the necessary skills and knowledge required for proficiently handling and analyzing Big Data in healthcare contexts.

Methods:
The study used a quantitative approach to gather data from 150 participants in health libraries and Information Science fields who completed Data Science programs in Tanzania. The survey evaluated their perceptions, skill sets, and confidence in executing Big Data analysis post-program completion. Statistical tools like descriptive statistics were used to analyze the collected data, providing insights into the effectiveness of Data Science programs in enhancing Big Data analysis skills.

Results:
The study aimed to assess the efficacy of Data Science programs through quantitative analysis of participant responses. Among the surveyed professionals who completed the program (n=150), a significant 82% reported a noticeable enhancement in their skill sets related to Big Data analysis. Moreover, 75% of participants expressed increased confidence in applying advanced analytical techniques acquired from the program. Notably, 90% of respondents indicated satisfaction with the program's content and structure, highlighting its effectiveness in imparting practical skills for handling Big Data in healthcare contexts. The statistical data underscores substantial improvements in skill acquisition

Conclusions:
The survey analysis shows that Data Science programs significantly improve professional skills in Big Data analysis, enhancing competence and confidence. These findings have implications for health libraries and Information Science, fostering a more agile workforce capable of utilizing advanced data analysis techniques for informed healthcare decision-making. However, limitations like survey bias and study scope need to be addressed for further improvement.

Automated Search Translators: Comparative Effectiveness of Polyglot Search, Ovid Translator, and MEDLINE Transpose

Track(s): Clinical Support, Information Management, Innovation & Research Practice

Dan Stuart, Research and Instruction Librarian, Texas Tech University Health Science Center

Objectives:
How effective are automated search translation tools at reproducing MEDLINE search strategies?

Methods:
The current investigation compared search queries executed across three search translation tools—MEDLINE Transpose, Polyglot Search, and Ovid Search Translator—in order to perceive MEDLINE query reproduction success and efficacy through automated means. Five librarian-researchers engaged each of the three translation tools using five separate queries to perceive operability and distinctions among each one. The original five queries were created in PubMed, then iterated within each translation tool alongside a manual translation. Librarians recorded whether each iteration was successful as well as the total number of MEDLINE results.

Results:
Among successful iterations, there was only moderate variation between each tool's results. The majority of translated queries maintained a strong, even identical consistency of successful iterations and record tallies, yet each tool and each librarian's execution of each tool generally retrieved a dissimilar number of results at some point. Iterations across all queries and translator tools combined had a success rate of 82% and an overall z-score of 0.33. Only one of the five queries retrieved a failed iteration. Among all successful iterations, result tallies were more aligned with manually reproduced searches than with the PubMed original searches restricted to MEDLINE results.

Conclusions:
Automated search translator tools offer an innovative and perhaps efficient way of reproducing MEDLINE search strategies. In many cases they can translate original queries to perfectly match and even exceed the quality of manually reproduced queries. Yet there are visible limitations to their accuracy and applicability. Not all original searches, whether executed in PubMed or through other databases, correlate to working iterations. Furthermore, they can sometimes retrieve a disparate number of results. In particular, the tools of MEDLINE Transpose and Ovid Search Translator have issues with accurately translating searches to match the quality of a manual reproduction when more advanced nomenclature is involved.

Collating the Nomenclatural Development of Negative Media Use Behaviors in Databases of Scholarly Works: Considerations for Search Strategies

Track(s): Information Management, Information Services

Kaitlin Tiches, Medical Librarian II, Digital Wellness Lab at Boston Children's Hospital

Objectives:
The project aims to trace the evolution of terminology in negative media use behavior research, focusing on the transition from an initial emphasis on behavioral disorders and “addiction” to more diverted and nuanced perspectives. RQ1: How have the relevant terms identified in our study evolved in their conceptual focus over time?

Additionally, this research assesses the efficacy of commonly accessed databases in capturing the earliest iteration of a nomenclatural variation in published literature, as compared to the web-based indexing tool Google Scholar. RQ 2: How do traditional academic databases compare to Google Scholar in documenting new terminological introductions?

Methods:
For comparative analysis, the databases selected against Google Scholar were Web of Science, PubMed,

and PsychInfo. To ensure consistency, only the Web of Science Core Collection was searched to ensure all fields were keyword searched. Each of the 115 terms, noted from prior interactions with relevant published materials, was subjected to phrase keyword searches within the database, sorted by the oldest date first. The earliest date identified in these initial searches served as a parameter for subsequent Google Scholar searches. This methodological variation for Google Scholar searching reflects restrictions to advanced searching on the website.

After the date of the first usage was determined, a full-text verification was conducted to confirm the presence of the terminology. Once confirmed, the year, title of the material, and the database(s) were reported.

To evaluate the data, a timeline was created of the nomenclatural development. The study calculated the percentages of each database (Web of Science, PubMed, PsychInfo, Google Scholar) that first identified the earliest use of each term or phrase. These findings were then compared to determine the relative efficacy of the traditional databases in capturing the initial emergence of terminology.

Results:
The earliest appearing term describing negative media use, “television addiction,” appeared in 1954; three emerged in 2023: “Compulsive Digital Use,” “Problematic Use of Conversational AI,” and “Short Video Problematic Use.”

The years in which nomenclatural variations arose was not balanced (i.e. 59 emerged since 2010), but from 1954 to 2009, 42.8% of the terms used the words “addiction” or “pathological” compared to just 27.2% of those from 2010 onward.

Over half (63; 54.8%) of the nomenclatural variations were first documented in Google Scholar compared to the selected databases.

Conclusions:
In earlier research, terms that pathologize negative media use, such as “pathological” and “addiction”, were predominant. Recent years, however, have seen a rise in less pathologizing terms like “problematic”. Findings show that 54% of terms appeared earlier on Google Scholar than on other databases. This suggests that commonly accessed academic databases, which often are only able to search within abstracts, are insufficient for nomenclatural collation over time. However, there are major limitations to the search tools available in Google Scholar at this time, and researchers seeking to create timelines of terminology should consider comparative searching across multiple indexing tools.

Creating and Validating an Institutional Search Hedge

Track(s): Information Services

Ben Harnke, Research Informationist, University of Colorado Anschutz Medical Campus Strauss Health Sciences Library

Christi Piper, Research Informationist, University of Colorado Anschutz Medical Campus Strauss Health Sciences Library

Objectives:
An institutional search hedge is designed to find published scholarly output at an institution. Its output allows for an institutional bibliometric analysis, for example, to determine top journals published in. This analysis can aid libraries in collection development, literature search services or in providing metrics to institutional parties. We sought to create and validate an institutional search hedge that identifies published scholarly output by campus faculty in Ovid Medline.

Methods:
In developing this hedge we faced several challenges:

- Four-campus university system
- Non-standard campus affiliations
- Affiliation errors (spelling, etc.)

To build and validate the hedge we implemented a three-step process.

1. Find Unknown Affiliations
2. We designed an iterative search process to identify unknown relevant author affiliations in Ovid Medline.
3. Test Sensitivity

We selected random citations from a set of citations with some chance of containing a relevant affiliation. After screening we calculated a sensitivity score.

Test Precision

We randomly selected 10% of citations located for the years 2012 to May 2023. After screening we calculated a precision score.

Results:
Currently the search hedge locates 48,000 citations for the years 2012 to June 2023. The sensitivity of our search hedge is 98% and the precision is 98%.

Conclusions:
An institutional search hedge can provide valuable scholarly bibliometrics. It is important to validate the hedge to be confident in the results. An institutional search hedge will allow us to complete future projects, such as analyses about systematic review publications and the use of open access journals for publication, with a higher level of accuracy.

Current Status and Future Plan of Research Support Services (RSS) in Academic Medical Libraries in Taiwan

Track(s): Information Services

Tzu-heng Chiu, Professor & University Librarian, Taipei Medical University

Objectives:
Researchers in medical schools and large teaching hospitals have been under pressure to “publish or perish”. In Taiwan, the number of papers published by researchers in these institutions has always been the highest in all fields, and the quality and quantity of papers by teachers and clinicians have a deep impact on their re-employment and promotion. Therefore, these academic medical libraries should provide services to support their users with research need. It is the author’s hope that in addition to outlining current statuses and future plans for the provision of RSS, this study will also identify benchmark libraries for reference.

Methods:
The academic medical libraries in Taiwan as defined by this study include two national health science

research institutes and all medical schools and medical centers in Taiwan, with a total number of 28. The author collected data by means of questionnaire survey, distributed an online questionnaire on December 13, 2023, and collected a total of 24 valid questionnaires on January 4, 2024, with a recovery rate of 85.7%. The first part of the questionnaire is the basic information of the investigated library (category, location, number of staff, and number of professional librarians), and the second part is based on the four stages of the research life cycle (research plan writing, research paper writing, research paper submission, and post-publication), and proposes 18 research support services (RSS), which are investigated whether each academic medical library currently provides them, whether they plan to provide them in the next 1-3 years, or whether they do not plan to provide them (and the reasons for not providing them).

Results:
Of the 24 responding libraries, 14 are in the northern part of Taiwan, 4 in the central part, 4 in the southern part, 2 in the eastern part, with an average of 10.96 full-time staff and 7.5 LIS professionals. At present, the RSS provided by the most libraries are as follows: bibliographic management software (e.g. EndNote) (24/24), journal impact factor and ranking (22/24), Turnitin or iThenticate (21/24), and consultation for predatory journals (21/24); RDM writing consultation is currently only available in 1 library, and only 5 are planned to provide this service in 1-3 years. (See Table 1 for details)

Conclusions:
The survey identified three benchmark academic medical libraries, and 17 of the 18 RSS have been provided, including: the National Institutes of Health Library, the Taipei Medical University Libraries, and the National Yang Ming Chiao Tung University Library, the former two do not currently provide RDM service, and the latter one does not provide Altmetrics service. The medical center libraries that are not affiliated with medical schools (there are 9 in this study) are small in staffing and currently provide fewer than 10 RSS. Further research is ongoing, and the author is conducting in-depth interviews with the directors and business librarians of above academic medical libraries in Taiwan to understand the opportunities and difficulties of promoting research support services.

A Deep Dive into the Data: Categorizing the Raw Data from IPD Plans in ClinicalTrials.gov to Facilitate Comparisons

Track(s): Innovation & Research Practice

Sara Samuel, Informationist, University of Michigan

Diane Lehman Wilson, Administrative Manager, Office of Regulatory Affairs, University of Michigan Medical School

Emily Fleming, University of Michigan

Objectives:
We recently published a study examining our institution’s Individual Participant Data (IPD) plans submitted with ClinicalTrials.gov registrations prior to the new NIH Data Management and Sharing Policy going into effect in January 2023. IPD Plans describe how individual participant data from clinical trials will be shared. To maintain comparability, future research needs to be based on a unified approach to comparing different sets of IPD plans. This project identifies how best to categorize the IPD plan elements to understand if the content and quality of IPD plans has changed since the implementation of the new NIH policy.

Methods:
The data set and accompanying documentation from the initial study will be reviewed to understand the different categorizations for clinical study characteristics and the contents of the IPD Plan. We will then identify which variables in our data set could facilitate comparisons between two IPD Plan data sets. For

each relevant variable, we will then determine what type of result indicates a meaningful change. This approach to understanding our existing data is a stepping stone to a higher impact project in which we will be able to effectively compare two IPD Plan data sets.

Results:
Our poster will provide a visual table display of the identified variables and how each contributes toward demonstrating how IPD Plans have changed over time.

Conclusions: N/A

Delivering on Space as Service: Survey Use to Direct Library Design

Track(s): Innovation & Research Practice

Carrie Adams, Program Director, University of Florida

Christopher Eaton, Health Science Librarian, University of Florida

Objectives:
Our library serves three health sciences colleges (Medicine, Pharmacy, and Nursing) as well as a hospital. The space has not been renovated since 2009 and is currently underutilized. The effects of the COVID-19 pandemic on the hospital system and the library in particular very likely led to substantial changes in user practices. Visitor numbers dropped significantly during the pandemic and have not rebounded in the ensuing years. This study is designed to identify the needs of both current library users as well as non-users in order to implement space design that will improve the utilization of the library.

Methods:
Three core questions drive the research design:

R1: How is the library space currently being utilized?

R2: Why aren’t stakeholders using the library?

R3: What gaps are present that aren’t currently being met?

A survey will be distributed electronically through email lists, via online links on the library’s webpage, and QR codes within the library. Because non-users are not typically a focus on space surveys, it is important to identify additional methods of capturing their feedback and so electronic mailing lists for the various colleges will be used to distribute the survey for a larger swath of the identified service population.

The survey instrument is based on observational data from staff and informal conversations with stakeholders in the organization and questions will be delineated according to respondents’ self-reported library space use. Questions will consist of Likert scales, multiple-choice with freeform text options (using an “other” designation as a choice) as well as open-ended questions. Photo-elicitation will be used for both users and non-users to provide feedback directly related to photographs of various sections of the library as they are in their current form and offer suggestions or preferences related to the space.

Survey information will be analyzed utilizing both quantitative and qualitative methodology.

Results:
Data analysis is not complete, but results will be analyzed and available Feb 2024. We expect to provide data related to user/non-user needs and expectations for library space in the current environment. The results may help inform how smaller health science libraries serving both hospital and academic programs might best utilize limited space to provide more innovative ways to meet patron needs while incorporating a “space as service” concept.

Conclusions:
Library space surveys often focus on current users, as this population is more accessible. However, the lack of feedback from non-users misses a major portion of a library’s service population and may lead to space design decisions that are not as useful to the larger population. Therefore, a considerable focus of this study is related to capturing data from non-users of library space, which is currently limited in health science library research. Additionally, much of the previously published research took place prior to the pandemic, making the data collected from this space-use and -gaps survey an important tool moving forward.

Empowering Expertise: Unveiling the Preferred Pathways of Health Science Librarians in Professional Development

Track(s): Professionalism & Leadership

Margarita Shawcross, Teaching and Learning Librarian, University of Northern Colorado

Objectives:
The purpose of this poster is to share the results of a study conducted to determine the preferred professional development method(s) used by health sciences librarians.

Methods:
A survey was developed using Qualtrics and sent out through various listservs used by health sciences librarians and completed by 159 health sciences librarians. Health science librarians answered questions about use and preference as well as rating the resources on how these methods met their professional development needs.

Results:
The results of this study provide data and insight on which types of professional development health science librarians prefer and which they find most helpful as they become familiar with the nuances that come with serving as a health science librarian. This research led us to conclude that health science librarians believe that professional development is very important to their success as librarians. There was also a preference toward more traditional forms of professional development such as webinars, conferences and reading journal articles and less preference toward newer forms such as social media and Twitter.

Conclusions:
Professional development is important to health science librarians for several reasons including but not limited to the specialization of our work and health science librarians' increasing role in evidence-based practice. But working within a rapidly changing environment, such as the health sciences, and shrinking budgets there is a need to identify low cost and credible options for professional development.

Fellowship Programs' Experience with Library-Publisher Illustrated Curriculum: A Survey

Track(s): Education, Professionalism & Leadership

Bryan Hull, Head, Digital Publishing, Spencer S. Eccles Health Sciences Library, University of Utah

Kathleen Digre, Moran Eye Center, University of Utah

Meagan Seay, Assistant Professor, University of Utah, Moran Eye Center, Department of Ophthalmology

Sachin Kedar, Emory Eye Center

Objectives:
The objective of this survey was to determine the effectiveness of the illustrated curriculum and to gauge the accessibility of the resource to the intended audience.

Methods:
An email containing a survey was sent in July 2023 to all fellowship directors and recently graduated fellows of AUPO-compliant neuro-ophthalmology fellowship. Questions presented sought to gauge the usage of the curriculum, the effectiveness of the learning tool, the ease of searching and organization, and the presentation of material for learning. Survey results were analyzed.

Results:
Twenty-eight participants (20 fellowship directors and 8 fellows) completed the survey. Sixteen respondents (57%) reported that the illustrated curriculum (IC) was a required educational resource for their program and 89% confirmed having access to the IC. Most (43%) accessed the IC on the professional medical society's website, 30% accessed through STAT!Ref, and 22% used both. Participants felt positively about the educational effectiveness, comprehensiveness, and methods of presentation, while ease of search and organization of the IC were felt to be weaker. 56% agreed/strongly agreed the IC was their go-to resource.

Conclusions:
Overall, the opinions from fellowship directors and fellows regarding the illustrated curriculum (IC) were positive. It is seen as an effective and comprehensive resource for neuro-ophthalmology education. Further efforts to improve the the IC, particularly the organization and searchability, may lead to an improved user experience. Future plans are to expand this survey more broadly to the professional medical society's membership.

Googling Depression: A Critical Appraisal of Online Health Information

Track(s): Clinical Support, Education, Health Equity & Global Health

Misa Mi, Director of Medical Library, Oakland University William Beaumont School of Medicine

Objectives:
Evaluate the quality, content, and readability of the most popular depression websites in the United States.

Methods:
Eight of the top depression websites in the United States were identified through a comprehensive, multiple-

query search. A multimeric evaluation was performed using modified methods from previously published research. The websites were evaluated based on user-friendly design, credibility, accessibility, literacy, engagement, content and cultural sensitivity.

Results:
Data analysis revealed all websites had user-friendly designs and accessibility features. Most sites were credible; however, two sites were missing an author, editor and references. The average readability was 11.5 grade level based on the SMOG index. Most websites had a social media presence and allowed for reader engagement. All websites provided basic depression information but none included first-person accounts. Cultural sensitivity fared the worst. Only two sites were offered in multiple languages and only one mentioned at risk minority groups. Cultural stigmas related to depression were never mentioned.

Conclusions:
The results of this study suggest strong implications for development and delivery of credible online educational materials given increased mental health issues triggered or exacerbated by the COVID-19 pandemic. There is a serious need for culturally sensitive depression resources, offered in multiple languages. Further efforts are needed to create easily accessible and easy-to-understand depression resources for all health consumers, regardless of educational and/or cultural background. Improving public understanding of depression through online resources can help reduce the stigma around mental health illness as well as health disparities among diverse patients.

Improving Peer Review of Systematic Reviews by Involving Librarians and Information Specialists: Preliminary Results of a Randomized Controlled Trial

Track(s): Information Services, Innovation & Research Practice

Melissa Rethlefsen, University of New Mexico, University of New Mexico

A. Patricia Ayala, Research Services Librarian, Gerstein Science Information Centre

David Blanco de Tena-Dávila, Professor Lector i Coordinador de Recerca, Universitat Internacional de Catalunya

David Moher, Director, Centre for Journalology, Ottawa Hospital Research Institute

Holly Grossetta Nardini, Associate Director, Yale University

Jamie Kirkham, Professor, Centre for Biostatistics, University of Manchester

Kate Nyhan, Research and Education Librarian for Public Health, Cushing/Whitney Medical Library, Yale University

Lex Bouter, Professor Emeritus of Methodology and Integrity, Amsterdam University Medical Centers

Maurice Zeegers, Professor of Complex Genetics and Epidemiology, Maastricht University

Sara Schroter, The BMJ

Shona Kirtley, enior Research Information Specialist, Centre for Statistics in Medicine, University of Oxford

Tara Brigham, Supervising Librarian in Florida, Mayo Clinic

Whitney Townsend, Health Sciences Informationist, Taubman Health Science Library, University of Michigan

Objectives:
To evaluate the effect of adding librarians/information specialists as methodological peer reviewers to the formal journal submissions process on the quality of search reporting and risk of bias in systematic review (SR) searches in the medical literature.

Methods:
We conducted a randomized controlled trial in The BMJ, BMJ Open, and BMJ Medicine using a pragmatic approach. SRs and related evidence synthesis manuscripts submitted to and sent out for peer review from January 3, 2023 to September 2, 2023 were randomized (allocation ratio, 1:1). Randomization was stratified by journal and used permuted block randomization (block size = 4). Manuscripts in the control group followed usual journal practice for peer review. In the intervention group, manuscripts followed usual practice, plus had a librarian/information specialist peer reviewer invited. Manuscripts were tracked until January 2, 2024. All first revision manuscripts submitted by the 2024 date were analyzed for quality of search reporting using four pre-specified PRISMA-S items and for risk of bias using ROBIS Domain 2. Assessments were done in duplicate and assessors blinded to group allocation. We used intention to treat and per protocol analyses to assess differences between groups in the proportion of adequately reported searches (PRISMA-S) and risk of bias (ROBIS Domain 2). We also assessed differences in the proportion of manuscripts rejected as the first decision between groups. The study protocol was registered (DOI:OSF.IO/W4CK2) and the protocol published (DOI:10.1186/s13063-021-05738-z).

Results:
2,670 manuscripts from the three journals were sent to peer review during study enrollment. 400 met inclusion criteria and were randomized, including 62 manuscripts from The BMJ, 334 from BMJ Open, and 4 from BMJ Medicine. During the study period, 87 intervention and 59 control group manuscripts were rejected as the first decision ($\chi 2$ (1, n=397) = 7.84, p=0.005). By study close, 76 first revisions were submitted in the intervention group and 90 in the control group. Assessments of first revision manuscripts using PRISMA-S and ROBIS Domain 2 are ongoing.

Conclusions:
Assessment is ongoing; results and conclusions will be shared at MLA.

Improving the Library User Experience at a Dual Health Sciences Library

Track(s): Information Services

Naomi Bishop, Associate Librarian, University of Arizona College of Medicine-Phoenix

Victoria Caine, Health Sciences Librarian, University of Arizona College of Medicine-Phoenix

Objectives:
To obtain feedback from Health Sciences students on how to improve their user experience of library space, resources, and tools.

Methods:
Students were given a digital survey through Qualtrics. Questions on the questionnaire focused on the library spaces, access to online content, library staff, and materials in the physical collection.

Results:
Student feedback provides library staff data to consider changes that could be made to the library physical and digital spaces.

Conclusions:
Library staff will use the data to implement suggestions in planning for growth and accessibility.

Knowledge and Awareness of Health Information Professionals Towards Telemedicine Services in Tanzania

Track(s): Health Equity & Global Health

Mary Charles, Knowledge and awareness of Health Information Professionals towards telemedicine services in Tanzania, UDOM

Objectives:
The research investigates the knowledge, attitudes, and application of telemedicine services among Health Information Professionals in Tanzania and also to support supports health professionals by providing timely data capturing and organizing patient data, extracting relevant information, and identifying patterns or trends for better decision-making in healthcare settings.

Methods:
An analytical cross-sectional study approach was utilized involving 50 Health Information Professionals working in healthcare settings in Tanzania selected by snowball sampling and web-based questionnaire via email.

Results:
The study revealed that, 66% of Professionals have high knowledge, 30% have partial, and 4% have low knowledge on of Telemedicine. The majority, 66%, are using telemedicine, while the minority, 34%, are not using it. About 14% said telemedicine can be used in disease outbreaks, 12% in tracking patients, 10% in treating patients, 6% in communication, 5% in healthcare training, 2% save costs. Approximately, 30% apply in hospitals, 20% apply online, and 16% apply in institutions/colleges.

Conclusions:
The majority of the participants were aware of telemedicine applications, having IT support, information sharing, information sources, and awareness, which are significant for telemedicine services knowledge. As a result, health information professionals should receive appropriate and ongoing awareness raising training on telemedicine systems. Moreover, future health services need research on telemedicine, observational and diary methods should be used to collect data on job tasks and competencies of HIP in Institutions and Health facilities

Let’s Chat (GPT)

Track(s): Education, Information Services

Genevieve Milliken, Data Services Librarian, NYU Langone Health

Kellie Owens, Assistant Professor, NYU Langone Health

Yuliya Yoncheva, Research Assistant Professor, NYU Langone Health

Fred LaPolla, Lead, Data Services, NYU Langone Health

Objectives:
The recent arrival of Large Language Models (LLMs) has already had a large impact across multiple academic disciplines; healthcare among them. The power, speed, and flexibility of LLMs makes this new Generative Artificial Intelligence (AI) technology an attractive tool. To meet demand and interest in Generative AI, our health sciences library taught three classes: prompt creation, fundamentals of generative AI and AI ethics.To better understand our community, we distributed a pre-post survey after each workshop. This survey was designed to help instructors assess baseline understandings and misconceptions students were bringing with them when learning about how to use LLMs.

Methods:
This research utilized a survey research methodology to understand user’s comprehension of fundamental concepts related to LLMs. The anonymous survey was distributed in REDCap, and will be redistributed again in the Spring after an expanded curriculum that includes additional workshops. In the survey, we asked seven multiple choice questions, provided a means for participants to rank their confidence with LLMs using a sliding scale, and asked four open-ended response questions. This survey was created through instructor group consensus and was not validated, but aimed to understand learner competency through a pre-post survey distributed before and after attending a workshop.

Results:
When the pre-post survey was distributed in Fall 2023, we received 35 responses for the pre- survey and 4 responses for the post- survey. Basic summary stats showed that the biggest area of misconceptions were around technical terms like “few shot” learning and what can be done with the institution’s internal AI studio. Further, free text responses were related to concerns about inaccuracies in AI responses. During the last distribution of the survey, only four people filled out the post-survey and, while this is too small a sample to draw conclusions, students selected more correct answers in the multiple-choice questions. Data collection will continue in March 2024.

Conclusions:
This research highlights the marked need for training to clear up misconceptions around AI usage at an academic medical center, and that educational workshops may fill this need. Looking forward, we aim to dig deeper to understand student comprehension around how LLMs work, whether or not they are using an appropriate framework for creating prompts, and their personal/professional perspectives on using LLMs in the workplace.

Librarian Contributions to Systematic Review Studies Published in Four Top Chaplaincy and Pastoral Care Journals

Track(s): Education, Innovation & Research Practice

Kerry Dhakal, Associate Professor, Research and Education Librarian, Health Sciences Library, The Ohio State University

Objectives:
To learn about the role and settings where librarians have conducted systematic reviews in the clinical specialty of chaplaincy/pastoral care.

Methods:
The study searched for all systematic reviews published in four top journals in chaplaincy/pastoral care (Journal of Health Care Chaplaincy, Journal of Pastoral Care & Counseling, Journal of Religion and Health and Journal of Palliative Medicine) between 2000-2024 in the PubMed database, using the following search

strategy: ((("J Palliat Med"[Journal]) OR ("J Relig Health"[Journal]) OR ("J Pastoral Care Counsel"[Journal])) OR ("J Health Care Chaplain"[Journal])) Filters: Systematic Review.

The author then reviewed each article for author affiliation data to see if librarians were listed as co-authors or acknowledged as contributing to systematic review studies. Additional data points to be reviewed include author credentials (librarians and non-librarians); author scholarship (librarians and non-librarians), type of librarian, library, chaplaincy/pastoral care unit, and clinical setting. The study will also review search strategies (when available) in systematic review articles where librarians are co-authors/contributors and compare them with search strategies of systematic reviews where librarians are not co-authors/contributors.

Results:
One-hundred and fifty-one systematic review articles resulted in this search. Fourteen of these systematic reviews include librarians as co-authors. The study poster will report observable differences and opportunities in the conduct of the systematic reviews (i.e., search strategy information, guidelines used, systematic review screening tool use) depending on if/when librarians were involved as co-authors or not, as well as analysis of the demographic data focusing on author credentials, author scholarship history, and type of librarian, library, chaplaincy/pastoral care unit, and clinical setting.

Conclusions:
The study hopes to identify and offer recommendations for librarians interested in collaborating on systematic review publications with professionals in the specialty of chaplaincy/pastoral care. The conclusions will be ready to share at time of the conference as the study is currently in the data analysis phase.

Librarian Expertise in a Race in Medicine Faculty Toolkit Project

Track(s): Health Equity & Global Health

Marlowe Bogino, Clinical and Reference Librarian, Rowan University

Karen Mitchell, Library Assistant, Rowan University

Susan Cavanaugh, Director, CMSRU Library Associate Professor, Department of Biomedical Sciences

Background:
Medical school administration convened an Ad Hoc Subcommittee for Curricular Improvement: Racism in Medicine, Racial Inequality in Medicine, and Trauma Informed Care, charged with developing Medical Education Program Objectives (MEPOs) in each area. Simultaneously a faculty survey revealed that faculty felt they did not have the knowledge to fully incorporate these MEPOs into course content or assess students on these objectives.

Objective:
Create a self-guided faculty development toolkit as part of other initiatives to increase faculty knowledge and practice of implementing the newly adopted MEPOs.

Methods:
Methods: Librarians partnered with biomedical science faculty to pursue grant funding for the project. Librarians contributed to identifying resources such as podcasts, webinars, TEDTalks, NPR segments and articles, curated the collection of resources by identifying and applying relevant MeSH terms to each resource, and created and applied a tool using Blooms Taxonomy to categorize the resources as introductory, intermediate and advanced to further curate and assist faculty in using the toolkit. A library staff assistant worked closely with faculty and librarians to create a Libguide to house the toolkit. Librarians

contributed to the development of a survey to study the impact of the toolkit on faculty knowledge, practice, and beliefs which resulted in an ongoing IRB-approved study.

Results:
The research on the impact of this toolkit project is currently in the data gathering phase. The focus of the poster will be to provide a snapshot of the toolkit development and highlight the value-added of librarian expertise.

Conclusions:
This collaboration highlights librarian skills in identifying, organizing, indexing and categorizing information. It also demonstrates librarian knowledge of pedagogy in the application of Blooms Taxonomy to inform the creation of a faculty development toolkit that accommodates a variety of learner levels of knowledge. This collaboration has contributed to solidifying relationships outside of the library, showcased the talent of library staff, and ultimately provided a template for future innovative and unique library enhanced projects.

Making the Most of Artificial Intelligence and Large Language Models: A Novel Approach for Book Recommendation and Discovery in Medical Libraries

Track(s): Information Services, Innovation & Research Practice

David Carson, Health Sciences Librarian, Chapman University

Ivan Portillo, Directory of Rinker Campus Library Services, Chapman University

Objectives:
Artificial intelligence and large language models have received increased attention since ChatGPT launched in 2022. Librarians can benefit from using artificial intelligence to reduce their workload and become more efficient. There is still skepticism of LLMs and their accuracy and reliability. Our objective is to determine if AI and LLMs can assist health science librarians in recommending and discovering book titles as part of their collection development.

Methods:
The researchers will design prompts to evaluate the capabilities of each LLM in performing their recommendations. The chosen LLMs are not only trained on a large corpus but also access the internet to search for relevant information. The prompts chosen by the researchers will ask each LLM to perform as a recommender system and seek domain-specific details (.edu, .gov, .org, etc.). The prompts will include only essential information required to guide each LLM in performing their task, while also clearing any previous search history to eliminate biases or user preference for reproducibility. The results generated from the LLMs will be evaluated on the capabilities of each LLM to provide title recommendations and the quality of the title list generated. Titles recommended by each LLM will be evaluated and compared to authoritative lists, including Doody’s Core lists and holdings from Worldcat.org. Each LLM is also designed to provide references on where data was collected and how recommendations were generated. The references provided will also be evaluated for authority, accuracy, and reliability. Evaluating the references will help determine how each LLM recommends items and determines which type of data it leverages.

Results:
The titles presented in each LLM recommendation, and the final evaluations will help determine if librarians can leverage these tools to reduce workloads and discover new titles to aid them in their collection development. The prompts provided will also be reproducible to help librarians generate similar results in the subject areas of their interests.

Conclusions:
As artificial intelligence and large language models continue to evolve, librarians can utilize these tools to help reduce workloads and become more efficient. In the current iterations of LLMs, the ability to search online data has helped users break away from an outdated corpus and locate current data from specific websites. However, results can still be inaccurate, and generative artificial intelligence can attempt to predict text, which can result in unreliable results. This study looked to provide how pre-designed prompts can help eliminate inaccurate results and help in the discoverability of new resources that can benefit librarians.

Medical and Health Science Librarian Advertised Salaries Compared to the Living Wage and the MLA Salary Survey: An Exploration of Alignment

Track(s): Professionalism & Leadership

Emily Harris, Dental Medicine & Cancer Librarian, Augusta University

David Petersen, Research & Learning Services Librarian, University of Tennessee Graduate School of Medicine

Objectives:
Rising inflation and cost-of-living increases are instigating a renewed focus on librarian salaries. Researchers investigated salaries posted in medical and health science librarian job postings during 2022-2023 and compared the data obtained to results of the 2023 MLA salary survey. To provide additional context to the salary data, researchers utilized The Living Wage Calculator to adjust for cost-of-living differences. It remains important for librarians and those entering the profession to have data for making informed decisions that will impact their career and financial future.

Methods:
Researchers compiled medical and health science librarian job postings from 1 July 2022 – 30 June 2023 from the Medical Library Association’s website, the American Library Association’s Joblist, the medlib-l email listerv, and other MLA caucus listservs. Location, position title, and salary data such as posted minimum, maximum, or exact salary was cataloged in a spreadsheet. Researchers compared the median salary from job postings to the median salary from the most current MLA salary survey (2023). Researchers used The Living Wage Calculator to find the percentage of living wage each position paid based on posted minimum salary. This data was further broken down by NNLM Region to assess regional differences. Additionally, researchers utilized the tool to calculate the percentage of living wage paid for each position when considering a sample family situation of two adults and two children with one adult employed. Researchers recognize that minimum posted salaries may not reflect actual offered salaries; however, this data provides a useful reference point for either new LIS graduates or current librarians seeking a new position.

Results:
Preliminary results suggest that advertised minimum salaries are consistent across all NNLM regions as a percentage of living wage for both individuals and family situations. For non-administrative librarians, the median minimum posted salaries in job postings was $60,000; this figure is under the 25th percentile for reported salaries in MLA’s 2023 salary survey. For non-administrative librarians, the median maximum posted salaries in job postings was $75,235, which is under the median MLA reported salary in 2023.

Conclusions:
Results suggest a difference between advertised salaries and the 2023 median salary as reported in the MLA salary survey. Even when focusing on the maximum posted salary, the results still fail to meet the median salary in MLA’s salary survey. The job postings reflect a much broader pool of medical/health

science librarian positions than MLA institutional members. Positions offering higher wages may have many applicants, while those unable to compete may struggle to recruit qualified candidates. Potential applicants should compare the posted salary to the local cost-of-living/living wage as a negotiating tool and use the posted salary to ascertain whether to apply for the position. LIS graduate students should also examine salary data to inform specialization and career decisions.

Move Over Dr. Google: Assessing Large Language Learning Models’ Ability to Accurately Answer Consumer Health Questions

Track(s): Health Equity & Global Health, Information Management, Innovation & Research Practice

Laurel Scheinfeld, Health Sciences Librarian, Stony Brook University

Sunny Chung, Health Sciences Librarian, Health Sciences Library

Jessica Koos, Director, Health Sciences Library, Stony Brook University

Objectives:
Large Language Learning Models (LLM’s) have become increasingly popular since the launch of ChatGPT, and they have the potential to be used by consumers in searching for health information. However, the quality of this information is unknown. This study sought to evaluate the accuracy of various LLM’s in answering consumer health questions, in order to determine if the health information they yield is reliable.

Methods:
The top ten trending consumer health questions in the United States in 2023 were identified based on data provided by Google. Four LLM’s that are free and accessible were identified, including Bard, ChatGPT, Claude, and LLaMA . Each query was entered separately into the four LLM’s, and the responses as well as included references were recorded on a spreadsheet. The results were compared to information provided on that topic in MedlinePlus in order to determine accuracy. Each response was evaluated for accuracy based on a rating scale developed by Sallam et al (2023). The references provided were also searched to see if they were accurate as well.

Results:
The results are pending as of the submission of this abstract. The data obtained from this study will be represented in various data visualizations, appropriate to a poster format.

Conclusions:
Pending.

New England Salary Survey 2023

Track(s): Professionalism & Leadership

Lisa Adriani, Research & Instruction Librarian, Quinnipiac University

Stephanie Friree Ford, Manager, Library Resources, McLean Hospital

Jessica Kilham, Associate Director, Network of the National Library of Medicine, Region 7

Karen S. Alcorn, Reference and Instruction Librarian; Associate Professor, MCPHS

M Lisa Liang Philpotts, Mass General Hospital

Meredith Solomon, Manager, Outreach & Public Services, Harvard Medical School

Objectives:
To determine what the current salaries are, the trends, and examine health science information professionals’ salaries within multiple states. We will seek broad data about the profession across health science libraries and share it to assist librarians in salary negotiations.

Methods:
A survey was created and adapted from a 2019 salary survey, which was based on a survey conducted in 2013/2014. It was distributed by email to multiple health science librarian listservs covering the states to be studied. It was distributed multiple times to ensure saturated coverage of the region. The data will be collated for all of the states and compared with the earlier data and all states will be compared with the rest of the nation using the data from the MLA 2023 national survey. Results will be shared via a poster presentation.

Results:
95 librarians from the region we surveyed responded to the survey for a response rate of 41.6%. The mean salary by institution and position type will be determined, as well as the number of full-time jobs vs. part-time jobs, and an analysis of types of libraries and positions and their distribution across the region. This information will be compared and the differences and trends through the years from the earlier surveys will be reported. Data is still being evaluated and analyzed at this time.

Conclusions:
Data is still being evaluated and analyzed at this time.

"No More Boxes": Information Experiences of Students Transitioning to Clinical Education

Track(s): Clinical Support, Education, Information Services

Nena Schvaneveldt, Associate Librarian, Spencer S. Eccles Health Sciences Library, University of Utah

Objectives:
Literature about the information practices and experiences of health professions students seldom focuses on their strengths and experiences. Librarians and other health sciences educators benefit from understanding student perspectives on their own education. This study investigates student experiences to address the research questions: What are the information practices of health professions students at the transition to clinical education? How do these students understand how their needs have developed over their education?

Methods:
Students from six health professions programs (dentistry, occupational therapy, medicine, pharmacy, physical therapy, and physician assistant studies) were recruited to participate in the study during the first semester of their clinical phase of education. They participated in semi-structured interviews to reflect upon their information practices. These programs were selected as the entry point to their professions are at the graduate level of education and they have a clear split between didactic and clinical education. During the interviews, participants constructed timelines of how they came to their current methods of using information. After the interviews, they each completed two solicited structured diary entries to capture more recent experiences. These methods center student voices and experiences. The interviews, timelines, and diaries are presently undergoing thematic analysis to determine patterns in the information practices of these students, how they make sense of information problems, and how they developed their practices. Analysis seeks to identify commonalities and differences among disciplines, clinical settings, and other

factors as they emerge from the data. The timelines will be synthesized into a "typical timeline" for how these students learn to use information.

Results:
Preliminary results indicate that students value developing their competency in service of providing excellent patient care. They prefer reliably accurate, easy-to-use sources of information as they navigate a litany of complex problems. They are keenly aware of the tension between academic success and the practicalities of providing care within the US health system, as well as being both authorities and novices in their field. They were able to make connections between earlier experiences - academic as well as personal - to their present context.

Conclusions:
Health professions students in early clinical education are motivated to build their competency in service of patient care. Their preferred sources vary depending on how relevant the student perceives them to be - videos are far more useful to students interested in procedures, and blogs and other popular sources were noted as valuable sources in situations where rigorous research was not available or possible. By understanding more about how these students experience information and how they solve problems, librarians can better engage with them as early clinicians. Linking library instruction and work to student motivations may produce more satisfying outcomes for all parties.

Plagiarism Checking Procedures Among Oncology Research Journals

Track(s): Innovation & Research Practice

Johanna Goldberg, Research Informationist, Memorial Sloan Kettering Cancer Center

Celine Soudant, Research Informationist, Memorial Sloan Kettering Cancer Center Library

Kendra Godwin, Research Informationist II, Memorial Sloan Kettering Cancer Center

Robin O'Hanlon, Associate Librarian, User Services, Memorial Sloan Kettering Cancer Center

Objectives:
The Committee on Publication Ethics (COPE) defines plagiarism as “when somebody presents the work of others (data, words or theories) as if they were his/her own and without proper acknowledgment” (COPE, 2023). This definition leaves significant room for interpretation. One study found that fewer than half of the 100 journals surveyed defined plagiarism in their author instructions, and just three detailed their responses to plagiarism (Sun Y-C, 2021). In this study, we will survey editorial staff of oncology journals to determine their processes and policies for detecting and responding to suspected plagiarism in unpublished and published manuscripts.

Methods:
In August 2023, we searched Journal Citation Reports (JCR, Clarivate) for journals in the Oncology category of the Science Citation Index Expanded (SCIE) Index for 2022, the most recent year available. We exported the list into Excel and then used journal websites, web searches, and tools like JCR and PubsHub (ICON) to identify contact information associated with the journals. We developed an online survey in REDCap and pilot tested it in November 2023 with three editorial staff members and one editorial board member of medical journals. We adjusted the questions based on their feedback and applied for Institutional Review Board exemption, which was granted in January 2024. Next we will email the more than 240 journal representatives an invitation to complete the survey. After receiving responses, we will use REDCap to collect de-identified information, Stata to analyze the quantitative data and qualitative thematic analysis for the open-ended questions.

Results:
We aim to use the collected de-identified data to address the following questions: What do oncology journals consider to be plagiarism? How do oncology journals detect plagiarism? How do oncology journals respond to suspected plagiarism in submitted manuscripts? How do oncology journals respond to suspected plagiarism in published articles? How effective do oncology journal professionals perceive their plagiarism policies and practices to be in preventing and addressing plagiarism?

Conclusions:
We anticipate the forthcoming survey results will offer valuable insights into scholarly editorial anti-plagiarism practices. The findings may inform library subscription decisions regarding plagiarism checking tools. In addition, it will influence how our library staff educate users on editorial anti-plagiarism procedures. These include best practices for analyzing plagiarism-checking software reports, minimization of self-plagiarism, retraction and expression of concern procedures, and policy differences across the discipline. These results may shed light on the effectiveness of current plagiarism detection methods and highlight areas for potential improvement in editorial standards and ethical practices.

Proactive Publishing, Metaliterate Learners, and Open Access, Oh My!

Track(s): Education

Christina Wissinger, Health Sciences Librarian, Penn State University

Kat Phillips, Nursing & Allied Health Librarian, Penn State University

Objectives:
Updating the approach to predatory publishing involves evolving current information literacy instruction to focus on the new approach, coined Proactive Publishing, which adeptly distinguishes Open Access (OA) publishing as an opportunity within the broader scholarly communication conversation. To implement the concept of proactive publishing in practice, information literacy sessions and individual consultations need to start from this empathic approach. Once librarians understand the perceived barriers their users associate with OA, consultations and literacy sessions can be customized to address those concerns and illustrate pathways toward OA publishing.

Methods:
The authors reviewed established theories relating to intrinsic and extrinsic needs and motivation. Maslow's hierarchy of needs (1943), was selected as an psychological hierarchy that could support OA publishing information literacy sessions from the perspectives of Metaliteracy and empathy. The five levels of the Maslow Hierarchy were adapted to scholars at various levels in academic careers. These areas were used to identify ways empathetic OA publishing instruction can be facilitated.

Results:
The following maps Maslow’s hierarch levels to the scholar progression of academic appointments from an empathetic perspective.

Physiological = Obtaining a graduate degree

Safety = Securing a teaching or research position

Belongingness = Publishing and presenting work with respected journals and conferences

Esteem = Obtaining tenure and achieving a national reputation (aka Keynote Status)

Self-actualization = Actively sharing work without the necessity of acclaim (aka Academic Freedom)

Conclusions:
Viewing proactive publishing as part of the empowerment of Metaliterate Learner is an easy concept to include. Moving the discussion towards scholars as powerful producers of information begins to change the perception of OA barriers into points of negotiation and the value of alternative publishing venues supporting the growth of information literate consumers and producers of information.

Revelations from Weeding: Historical Decisions and Implications for Current Collection Management

Track(s): Information Management, Innovation & Research Practice

Ann Dyer, Director, Washington State University Health Sciences Library

Suzanne Fricke, Medical Librarian, Washington State University Health Sciences Library

Objectives:
While medical library resources are increasingly electronic, thoughtful curated print collections remain important for community engagement and public perception of libraries. To achieve this goal continuous deselection of library materials (weeding) is necessary, though time consuming and often thankless.

While published scholarly literature provides weeding recommendations and methods, they are applied inconsistently across individual selectors and libraries. In this case study, librarians pinpoint materials identified in a weeding project that warrant deselection for reasons beyond those elucidated in published weeding methodologies.

Methods:
Academic librarians from biomedical libraries within a land grant university system approach weeding on an as needed and continuous basis to modernize local collections and free up study space. This multi-campus system rests within a larger regional consortium that provides resource sharing and collaborative electronic collections. Collection development in this multi-campus institution relies on a Head of Collection Development, regional campus library directors, and subject selectors with approval plans, supplemented by patron requests.

For this study we began with existing weeding methods including MUSTIE (misleading, ugly, superseded, trivial, irrelevant, or obtained elsewhere) and CREW (continuous review, evaluation, and weeding). We moved on to assess quantitative date and use data, availability through consortium institutions, and qualitative internal and external reviews of individual materials. This process allowed librarians to identify examples that warrant deselection for reasons beyond those easily identified in existing weeding guidance - including retraction, medical bias, personally identifiable information, poor disciplinary reviews, etc. The analysis concluded with an assessment of how these items entered or stayed in the collection, and reflection on quality improvements needed for the future.

Results:
Librarians identified a subset of titles still in many libraries that were retracted by the publisher for medical bias, received poor reviews by disciplinary experts, or contained personally identifiable information.

Deselected books entered and stayed in the collection due to a combination of lack of prioritizing weeding, inadequate initial assessment, acceptance of donations, lack of metadata/alert systems indicating retraction, and past emphasis on collecting grey literature.

Many large medical eBook platforms have systems in place that point toward newer editions and flag retracted titles. This does not exist with general academic eBook aggregators and library management systems.

Conclusions:
In a time of shrinking workforces and consumer mistrust, institutions are tasked with being careful stewards of what they purchase and maintain. While curated print collections can be important for learning and library aesthetics, large collections create a management burden. Librarians should scan for medical bias, personally identifiable information and research ethics and misconduct when reviewing current collections. The development of automated systems for flagging these titles would assist time-strapped librarians in deselection. At the same time, acquisition models that rely on automation require the thoughtful oversight of knowledgeable subject experts more than ever.

So You're Opening a New Medical School? A Preliminary Examination of Academic Medical School Library Resources

Track(s): Information Services, Professionalism & Leadership

Kevin Pardon, Health Sciences Librarian, Arizona State University

Janice Hermer, Health Sciences Librarian, Arizona State University

Objectives:
As health sciences librarians employed by a large research university that recently announced plans to establish a new medical school, we aimed to conduct research to inform our understanding and recommendations on what library support for medical schools looks like today. Our preliminary research consisted of three objectives:

1. Identify and quantify library related databases and resources typically provided
2. Determine how common it is for an institution with a medical school to have a physical medical or health sciences library space
3. Gain a general sense of health sciences library staffing, with a focus on the liaison role

Methods:
Peer and similar research institutions with medical schools were identified and used as the basis for our data collection and analysis. Each institution’s library website was examined to track information related to our three objectives. We compiled and quantified the most widespread health sciences databases and resources provided. We also noted the number of health sciences liaison librarians and other library employees through examination of various staff directories, libguides, or other webpages. Additional observations were also noted to identify potential trends for more thorough exploration.

Results:
Analysis identified the most significant resources typically provided by the institutions. The majority of institutions owned or subscribed to what could be described as a core collection of databases and resources. It was also determined that more often than not a dedicated physical library space did exist. Library staffing varied widely, likely depending on a multitude of factors but typically a minimum of several liaison librarians or other specialized librarians were employed.

Conclusions:
Our initial results reinforced our general perception that a multitude of specific new resource subscriptions,

additional staffing, and dedicated physical space are commonly employed to support a new medical school at a large research university. Further examination of specialized services offered by medical libraries, related degree programs offered at these institutions, as well as an in-depth literature search to identify additional trends, remains necessary for providing more detailed and valuable findings.

Stronger Together - Exploring Medical Students Experiences with ChatGPT

Track(s): Education

Nadine Dexter, Director, Harriet F. Ginsburg Health Sciences Library

Emily Hannum, Research Services Libraian, University of Central Florida College of Medicine

Objectives:
To understand how students at a Southern Medical School are using ChatGPT to support their studies, extracurriculars, and fulfill other academic needs.

Methods:
Medical students in a Southern Medical school were asked to voluntarily complete a 15-minute length survey including Likert scale and open response questions regarding their use of and comfort with ChatGPT. A total of 64 medical students participated.

Results:
A majority of medical student respondents have used ChatGPT despite skepticism of usefulness and accuracy of information obtained. Most medical students are also either supportive of or indifferent to the recommendation of ChatGPT for medical students. The most frequently used sources for verifying ChatGPT information included Google and Clinical Apps such as UpToDate and Epocrates. Some of the major concerns for ChatGPT use included receiving simplistic answers, falsified studies, reflection of user bias, and ethical implications.

Conclusions:
Despite the general skepticism surrounding the benefits of using Large Language Model AI such as ChatGPT, medical students appear to already be incorporating these tools into their education albeit cautiously. This cautious exploration suggests that as AI tools continue to advance so will their application in medical education. Further research on the application of additional AI tools is necessary.

A Survey of Academic Librarians and Their Awareness, Perceptions And Implementation of the ACRL Cultural Competency Standards.

Track(s): Professionalism & Leadership

Rong Tang, Professor, Simmons University

Xan Goodman, Health Sciences Librarian, Associate Professor, University of Nevada, Las Vegas

Objectives:
A team of two librarians conducted a survey of academic libraries. The research aimed to investigate two

questions: How are librarians’ aware of the Association of College and Research Libraries (ACRL) standards on cultural competency? How do librarians implement the standards? The cultural competency standards cover 11 broad areas: 1) Cultural awareness of self/others 2)Cross-cultural knowledge/skills 3)Organizational/ professional values 4)Development of collections, programs, and services 5)Service delivery 6)Language diversity 7)Workforce diversity 8)Organizational dynamics 9)Cross-cultural leadership 10)Professional education/continuous learning 11)Research shall be inclusive and respectful.

Methods:
The survey contained thirty-nine questions. A total of N=279 responses were collected. Descriptive statistics were collected for survey respondents. Additional inferential statistics will be completed to determine associations between professional librarian role, implementation and awareness of standards. Open-text comments are qualitatively analyzed using thematic coding and summarized narratively to draw inferences about survey respondents’ perceptions of the ACRL Cultural Competency Standards.

Results:
A subset of results will be shared during MLA 2024. Preliminary results show the top five practices adopted to practice cultural competence in academic libraries are: 1) Developing diverse collections (141, 80.11% 2) Recruiting diverse candidates (115, 65.34%) 3)Adopting all gender restrooms (107,60.80%) 4)Programming to address concerns of historically underrepresented groups (101,57.39%) and 5)Programming to address cultural issues (97, 55.11%).

Conclusions:
This research represent an initial study into the use of academic standards by a professional library organization related to DEI and the awareness, implementation, and perceptions of these standards. Evidence of the top-five practices related to cultural competence in academic libraries is shared. These practice recommendations will provide practical ways that academic libraries and health sciences libraries might use to implement an aspect of DEI using cultural competency standards.

Together We Thrive: Assessing Library Access and Engagement in Nursing Education

Track(s): Information Services

Lisa Raney, Electronic Resources Librarian, The Christ Hospital Health Network

Jennifer Pettigrew, Electronic Resources Librarian, The Christ Hospital Health Network

Objectives:
In this assessment, we sought to increase utilization of the library’s resources and services among the College of Nursing students, faculty, and staff, restoring usage to pre-pandemic levels or better. We also wanted to identify potential improvements and access issues. Comprehensive surveys were conducted to assess awareness, usage patterns, and access barriers, both before and after implementing targeted interventions. Additionally, we wanted to know if study findings would support the purchase of a single sign-on solution such as OpenAthens to replace IP-authentication, a known access barrier.

Methods:
Library staff conducted a literature review and designed two survey questionnaires, one for students and one for faculty/staff. The surveys contained both Likert-scale quantitative questions with options for additional qualitative data, and two open-ended, qualitative questions. Aside from the demographic questions and the qualitative questions, the surveys were identical. The qualitative questions for students asked what they would change about the Library and what additional services they would like the Library to provide, and the open-ended questions for faculty/staff asked how library resources have supported

teaching and/or student learning, and what additional services would be helpful to support teaching and/or student learning. In November 2022, the surveys were sent to each respective user group with a two-week submission window in collaboration with the College’s Associate Dean of Institutional Effectiveness. Students who completed the student survey had the option to submit their e-mail address to be entered into a drawing to win a $25 gift card. Follow-up surveys were sent in April 2023 to gauge the impact of our Library interventions following the results of our initial survey.

Results:
Nearly 60% of all fall 2022 respondents reported desiring a library app (59% of students and 58% of faculty/staff); and almost 40% of all respondents in spring 2023 wanted an app to access library resources (38% of students and 36% of faculty/staff). 40% of faculty/staff and 15% of students respondents to the spring 2023 survey still reported trouble accessing the library’s intranet page. 30% of student respondents to the fall 2022 survey had never used the library compared to 8% of students who had used library services in spring 2023.

Conclusions:
Library staff was reduced from 2.5 FTE to 1.5 FTE in December 2022 which limited our capacity for planned interventions such as increased classroom instruction and training on library resources. However, limited interventions including an open house and posting information flyers at the College facilities correlated with a 71.3% increased use of library services by college faculty/staff in the five months following the distribution of the first surveys, and a 51.3% increased use of services during the five months after second surveys as evidenced by in-house usage statistics collected since 2019 using Google Forms. The surveys served as an unexpected outreach tool. Based on quantitative and qualitative data, we are pursuing a single-sign-on solution to replace IP-authentication for the College.

Using Bibliometric Analytic Techniques to Measure the Scholarly Impact of a Health Professions Education Teaching Academy

Track(s): Information Services, Innovation & Research Practice

Sarah Cantrell, Associate Director for Research and Education, Duke University Medical Center Library & Archives

Beth Blackwood, Research & Education Librarian, Duke Medical Center Library & Archives

Deborah Engle, Assistant Dean for Assessment and Evaluation, Duke University School of Medicine

Diana McNeill, Professor of Medicine at Duke University Medical Center

Kristin Dickerson, Duke University Medical Center Library & Archives

Objectives:
Health professions education (HPE) academies highlight the mission that is often second to clinical and basic science scholarship on health professions campuses. Due to their popularity, academies have proliferated over the past 3 decades. Yet the evidence of their impact on their organizations remains largely underdeveloped. As such, our objective was to use bibliometric analyses to assess the scholarly impact of our institution's health professions teaching academy. Further, we wished to codify bibliometric techniques for interdisciplinary teaching academies.

Methods:
We performed bibliometric analyses investigating publication trends of academy members in 37 HPE journals published between 2003 and 2023. We used the AAMC SGEA annotated bibliography to identify journals. From this bibliography, we excluded journals related to dentistry or veterinary medicine as these

programs do not exist on our campus. The time frame was selected to assess publication patterns both before and after the academy launch in 2014. To determine the overall quantity of institution-authored articles across the 37 HPE journals, we performed a comprehensive search for institutional affiliation and journals within Web of Science (via Clarivate) and MEDLINE (via PubMed); individual journal websites were also searched when there were gaps in database coverage. Journal Impact Factor (JIF) and quartile data were identified in Clarivate's Journal Citation Reports and included for those journals with an impact factor. Given that the 37 HPE journals include topics beyond education, we established eligibility criteria wherein articles must address topics related to health professions education. Citations were de-duplicated and screened in Covidence with the eligibility criteria in mind. After screening they were cleaned in Open Refine. Descriptive statistics were conducted using Microsoft Excel and Tableau.

Results:
Across the journals analyzed, 876 citations from the 2003-2022 timeframe met inclusion criteria for analysis. There were 209 publications prior to the launch of our academy (2003-2013) ; and 667 after the launch (2014-2022), representing a 219% increase, surpassing the documented doubling time of scientific publishing (Bornmann). Of the 876 citations, 48% were published in Q1 journals and 15% were published in Q2 journals. The process also identified weaknesses in bibliometric analyses due to inconsistent journal coverage across databases. We mitigated this by examining coverage in MEDLINE and Web of Science, and supplementing with searches of individual journal archives.

Conclusions:
Bibliometric analysis offers one way to explore the impact of teaching academies on the institution's educational scholarship productivity. In order to measure scholarly activity from an HPE academy, researchers performing bibliometric analyses must be aware of the indexing limitations within bibliographic databases. Researchers should note the journal coverage within databases, and search other databases and journal archives in order to fill gaps as needed. Researchers should use software to identify and remove duplicates, establish eligibility criteria, and screen out ineligible citations. Further, using journal quartile information in addition to the JIF is important as field normalized metrics are essential in interdisciplinary research. Not all categories of journals have large readership, citable items, or dedicated researchers on

What Impression? Initial Findings on Baccalaureate Nursing Students' Recall and Attitudes Toward Research

Track(s): Education

Jason Wardell, Health & Life Sciences Librarian, University of Dayton

Objectives:
This study aims to ascertain both the recall of information within a single cohort of undergraduate nursing students and their attitudes toward the concept of research, as well as how those might change within a cohort of students following a library instruction intervention and over the course of their studies. Through analysis of this data, the researcher hopes to gain a better understanding of their students' information-seeking needs and competencies.

Methods:
A mixed-methods survey instrument was developed to measure several information literacy competencies, including identifying qualities of evidence-based research, choosing appropriate databases for healthcare-related research, building a search query, and differentiating between popular and scholarly sources. Additionally, the survey included a multiple-choice question to measure attitudes toward research using the NRC Emotion Lexicon. The survey was distributed in a pre/post-test fashion to a cohort of 28 first-year BSN students in the fall of 2023, a day before the scheduled library instruction session and again three weeks

following the session. Thematic analysis was conducted of the qualitative results, and t-Test statistical analysis was conducted of the quantitative Emotion Lexicon results.

Results:
Within this single cohort, there were 26 responses to the pre-test and 16 responses to the post-test. A slight positive change was observed in the categories of identifying databases for healthcare research and constructing a search query, no change was observed in differentiating between popular and scholarly sources and identifying stronger types of evidence, and a negative change was observed in general attitudes toward research.

Conclusions:
Initial data suggests a lack of impact of information literacy instruction in first-year baccalaureate nursing students. This data will be useful as a benchmark for future investigations, specifically within the third-year nursing student cohort. Further data collection will be required to validate these results, with possible adjustments to the timing of the post-test to mitigate potential external factors that might affect student attitudes at the time of surveying. Future considerations will need to be made about the timing and content of information literacy instruction sessions if results are consistent in future tests.

Where are All the Job Candidates?: Geographic Considerations for Recruitment

Track(s): Professionalism & Leadership

Gregg Stevens, Manager of Library Education and Clinical Services, University of Massachussetts Chan Medical School

Allison Piazza, Clinical Medical Librarian, Weill Cornell Medicine

Andy Hickner, Education and Outreach Librarian, Weill Cornell Medicine

David Petersen, Research & Learning Services Librarian, University of Tennessee Graduate School of Medicine

Francisco Fajardo, Assistant Director, Florida International University

Objectives:
Many hiring managers and hiring committees have seen small numbers of applicants for vacancies at their health sciences libraries, making recruitment difficult. Several challenges are often cited for this, including geographic disparities between LIS schools and health sciences libraries. Our objective is to review the relevant literature and datasets, to quantify entry-level health sciences job postings in the United States for one year, and identify any geographic disparities relevant to these challenges. From this analysis, we hope to make some recommendations for both job-seeking candidates and hiring managers.

Methods:
After conducting an extensive literature review on recruitment challenges and opportunities within medical libraries, we explored academic journals, industry reports, and relevant publications to explore job candidate distribution. Medical and health science librarian job postings from MLA’s website, ALA’s joblist, medlib-l, and caucus listservs were compiled from January - December 2023. Entry level postings were determined based on several criteria:

- Only requirement listed is MLS
- Only requirement listed is MLS + knowledge (i.e. health science database experience or EBP or information literacy or OCLC/ILL/ILS)
- Postings that required an MLS + library or health science library experience

- Only requirements are MLS + 1 yr. experience
- Postings that ask for MLS + 1-2 years experience as 1 year officially qualifies

Postings that were duplicates or were for positions involving management, department heads, or unit heads were also excluded. Additionally, listings were deleted if they had no requirements listed as entry-level status was not able to be determined. Postings were then analyzed for geographic location, NLM region, and type of position.

Results:
Based on the medical/health science librarian job postings from 2023, there were 216 total postings including 105 entry-level positions (requiring one year or less of experience), reflecting approximately 49% of all job postings during this period. A plurality of entry-level postings (27%) were located in NNLM Region 1 while the fewest (5%) were from Region 4. Researchers analyzed the entry-level postings finding that instruction (67%) and reference (58%) duties were most prominent. Results from analyses of ALA data are TBD.

Conclusions:
Recruiting qualified entry-level librarians into medical and health science libraries remains a challenge for some institutions. The total number of entry-level postings (105) offers one data point on how many candidates will be needed to fill these positions. Geography is important, as a new LIS graduate living in a region with fewer opportunities may be forced to move in order to obtain a medical library position, and optimal approaches to recruitment will vary depending on the employer’s location. As this highlights just one aspect of the challenge, there are further research directions that may be taken from this analysis.

PROGRAM DESCRIPTION POSTER ABSTRACTS

We have sorted content in this section by title in alphabetical order.

Academic Health Sciences Library Leadership: A Twenty-Year Journey

Track(s): Professionalism & Leadership

Teresa Knott, Associate Dean, VCU Libraries, and Director, VCU Health Sciences Library, Virginia Commonwealth University

Shannon Jones, Director of Libraries, Medical University of South Carolina

Background:
The Leadership Fellows Program (LFP), funded by the National Library of Medicine (NLM) and the Association of Academic Health Sciences Libraries (AAHSL) celebrated its 20th year since its founding in 2002-2003. The yearlong LFP prepares senior level health sciences librarians for the rigors involved in becoming a director with the expectation that they will secure a director position within 2-3 years of completing the program. The curriculum has been routinely updated to keep pace with the changing demands and trends in the profession, emphasizing leadership development with an increased focus on recruiting fellows from diverse and under-represented backgrounds. The LFP is overseen by the AAHSL Future Leadership Committee.

Description:
Annually up to five selected fellows are individually paired with mentors who are experienced library directors in academic health centers throughout the country. Each fellow creates their own individualized learning goals which they intend to accomplish during the year. Written and oral feedback is provided. Assessment instruments, the Clifton StrengthFinder and the Myers-Briggs Type Indicator, are taken by all participants with both individual and group debriefs provided. The LFP consists of three major in-person events during the year including a full-day orientation in conjunction with the AAHSL Annual Meeting, a 3-day intensive Leadership Institute, and a 2-day Capstone event in conjunction with a Graduation ceremony. Over the past two years, the curriculum has embraced leadership diversity as a major element. Five virtual sessions facilitated by fellow/mentor pairs on self-selected leadership topics are conducted. Each fellow participates in a mock interview for a director position after providing a cover letter and CV to an assigned mentor who assumes the role of a leader who is recruiting for a new library director. Other activities include the power of questions, case studies, and activities centered on developing the emotional intelligence of participants.

Program Conclusions:
Including the 2022/2023 class, 102 fellows and 77 different mentors have taken part in the Leadership Fellows Program. Over half of AAHSL libraries have participated in one or more roles. Four of the mentors in the 2022/2023 class were prior fellows, bringing the total number of fellows who have become mentors to nineteen (19). Of the 102 fellows who completed the program, 52% (61) of fellows have become academic health sciences library directors. Two major program evaluations have been completed; one in 2005 and the other in 2013. A third evaluation covering the past 10 years is in development.

Addressing Health Misinformation: Evidence-Based Strategies and Community Engagement at a Virtual Symposium

Track(s): Professionalism & Leadership

Carolyn Martin, Outreach and Education Coordinator, Network of the National Library of Medicine, Region 5

Bobbi Newman, Community Engagement and Outreach Specialist, Network of the National Library of Medicine

Margie Sheppard, Community Engagement Coordinator, Network of the National Library of Medicine, Region 3

Miles Dietz-Castel, Communications Specialist, NNLM Region 6

Veronica Milliner, Engagement Specialist, NNLM All of Us Program Center (NAPC)

Background:
Health misinformation, while always a concern, saw an increase in abundance during the pandemic, making it difficult to discern between what is true and what is not. This virtual symposium was created to provide attendees the information on how to distinguish between types of misinformation, describe current research, identify resources and tools to combat misinformation, and to explore programs, projects, and practices related to health misinformation in communities. Objectives include ways of distinguishing between various categories of misinformation, delving into contemporary research endeavors dedicated to health misinformation, proficiently recognizing and applying resources and tools for mitigating health misinformation, and exploring an array of community-oriented programs, projects, and practices targeting the issue of health misinformation.

Description:
Attendees were offered the opportunity to delve into the research surrounding the health mis/disinformation movements. The event aimed to equip librarians, health educators, and direct care providers with practical, evidence-based solutions to effectively combat the spread of health misinformation in their respective fields. Sessions focused on data, public health, libraries, and general health misinformation.

The three-day virtual symposium featured multiple tracks with keynote speakers who are experts in the health misinformation field. A call for symposium proposals resulted in 49 submissions, of which 28 were accepted. One of the objectives was to amplify voices from historically marginalized communities within their respective fields. To this end, the symposium sought out presenters from underrepresented demographics, including individuals with disabilities, neurodiverse individuals, LGBTQIA+ community members, Black, Indigenous, and People of Color (BIPOC). Notably, 20 of the approved presentations featured contributors from these demographic groups.

The symposium featured a wide range of topics, including strategies for preempting misinformation, gamification to enhance health information literacy skills, empowering underrepresented communities through health literacy, education targeting health professionals, students, and library patrons, and collaborative partnerships devised to combat the proliferation of health misinformation.

Program Conclusions:
The symposium had a total of 1,756 registrations with 771 people participating in the live event. Additionally, the virtual education fair had 731 unique visits, signifying substantial engagement with the resources and content offered.

The post-event survey data is currently undergoing comprehensive analysis. This survey data holds valuable insights that will contribute to a deeper understanding of attendee feedback, preferences, and overall satisfaction with the event.

Advancing Biomedical Publishing and Education: Hospital Librarians at the Forefront of Artificial Intelligence in Academic Healthcare

Track(s): Innovation & Research Practice, Professionalism & Leadership

Leanna Stager, Research Analytics Librarian, Northwell Health

Stacy Posillico, Senior Librarian, Northwell Health

Regina Vitiello, Librarian, Northwell

Jaclyn Morales, Senior Librarian, North Shore University Hospital

Background:
Since its debut, ChatGPT has been driving rapid advancements in the use of artificial intelligence (AI) in healthcare. Clinicians want to integrate this transformative technology into biomedical publishing, clinical research, and medical education, but face a lack of guidance. Throughout 2023, hospital librarians in a large academic healthcare system received numerous inquiries on using AI in teaching, research, and writing. Some sought assistance in retrieving literature recommended by ChatGPT, only to discover that the references were computer-generated hallucinations. Others asked the library to recommend AI platforms that would provide trustworthy information. Recognizing the need to be early-adopter AI leaders, they aimed to explore and demystify AI's current and potential applications in shaping healthcare knowledge

dissemination.

Description:
Three librarians presented a two-part series of online educational sessions sponsored by the system’s research institute. In the first session, the key objectives for learners were to: define what AI is and the role of machine learning, describe the difference between human systems AI and ideal systems AI, and explain the methods used to educate the computer while understanding the benefits and risks of using AI in healthcare, as well as the implications for data integrity. In the second session, the key objectives for the learners were to: explain what a large language model is and how it supports the development of natural language processing tools, discover ChatGPT and other platforms people are using in the writing process, and determine the ethics and moral considerations of when natural language processing tools should be used, including the possibility for introducing bias into the AI outputs. To enhance the presentation, the second session included a panel discussion with four healthcare system researchers and faculty leading the way in using AI. The team is now providing real-time demonstrations of how to use generative AI systems and curating a list of journals’ policies on permitted AI use.

Program Conclusions:
The educational sessions received high praise from participant evaluations sent by the research institute. Fifty percent of attendees came away with an outstanding level of achievement in understanding AI, and half of the attendees also felt that their achievement in understanding ChatGPT and other platforms used in the writing and publishing process was outstanding. Planning is underway for future opportunities to contribute to the responsible and informed adoption of AI technologies by the healthcare system, particularly in scholarly communications. This poster will equip librarians with the knowledge needed to navigate the evolving AI use in healthcare. It will also guide them in supporting efforts to integrate AI within their organization while ensuring alignment with established research and educational standards.

Advocating for Inclusivity in Health Sciences Education with Librarian-Led Textbook Affordability Initiatives

Track(s): Education

Christine Andresen, Associate Professor, Medical University of South Carolina

Background:
Student textbook costs have increased exponentially, 162% between 2000-2021, and the National Center for Education Statistics reports that 81% of medical doctors/75% of health professions doctorates are burdened with student loan debt. As institutions are actively investigating cost-saving measures in the current climate of concern with student debt, librarians emerge as natural leaders of textbook affordability initiatives and are well-positioned to advocate for faculty adoption of zero or low-cost textbooks and ancillary materials to offset some of the financial burden associated with higher education. At an academic health system granting degrees across six colleges, one librarian-led grass roots initiative aimed to raise awareness of this significant problem and present an opportunity for an innovative educational solution.

Description:
Administrators and faculty know that access to instructional resources is essential to student success, but are often unaware of the cumulative cost of those materials each semester and may not know that students have even reported skipping meals in order to afford their textbooks and ancillary materials. The first step to raising awareness about this systemic issue on campus, was to gain access to the master textbook lists for one college and conduct a cost-savings analysis to determine roughly how much money the college theoretically saved each student during the Fall 2023 semester by utilizing library-subscribed resources for required or recommended textbooks. Even as rough estimates the cumulative cost-savings were staggering,

which was a win for library collections and eye-opening for faculty and administrators across campus. The report ignited interest in implementing textbook affordability initiatives and with academic leadership support, a consultation service was launched to support faculty adoption of library-subscribed or OER course materials. Consultations are collaborative with faculty member(s), the librarian, and the college's instructional designer who coordinates with the educational technologist to update the learning management system after adoption.

Program Conclusions:
Faculty and administrator response to the report has been positive and there is growing interest in the consultation service. Next steps include completing the pilot cost-savings analysis for the full academic year, expanding the textbook list analysis of library-licensed required or recommended materials to additional colleges, and working with the Office of Student Financial Literacy to provide updated estimates of school expenses to help students budget. Measured outcomes of our textbook affordability initiatives will include tracking the number of consultation service requests, count of how many courses replace a traditional textbook with library-licensed or OER materials, and changes in student cost-savings. Eliminating this barrier to health sciences education may ultimately result in a more diverse pool of students.

Beyond the Numbers and Shelves: Diversity Inclusion, and Access in Action!

Track(s): Health Equity & Global Health, Professionalism & Leadership

Dot Winslow, Library Assistant, A.T. Still University

Leslie Golamb, Liaison Librarian, A.T. Still University

Maud Mundava, Campus Head/Assistant University Library Director, A.T. Still University

Background:
A.T. Still University of Health Sciences is the founding institution of osteopathic healthcare, dedicated to whole-person healthcare and preparing highly competent professionals who are ready to embrace society's diversity. To better prepare our professionals for the diverse society, commitment to diversity is key to promoting the University's mission and values. In alignment with the University's and library's mission and goals, a task force was created to determine the library's Diversity, Inclusion, and Accessibility needs. This poster will:

1. Describe the process of creating a diversity committee, statement, and plan
2. Demonstrate how the library has successfully aligned the DEIA activities to the university and library strategic plan
3. Explain best practices and strategies to foster and increase awareness of DEIA issues

Description:
Diversity committees and initiatives in academic institutions, particularly in health sciences libraries aligned with the mission and goals of the institution are paramount to fostering equity, inclusion, and accessibility. Additionally, they create opportunities for improved access to community health equity and promote inclusiveness, a welcoming environment, and cultural awareness competencies among diverse campus stakeholders. This poster will:

1. Describe the steps on how to start your diversity, equity, inclusion, and accessibility journey within your library.

2. Demonstrate how the library has successfully aligned the DEIA activities to that of the University and library strategic plan. Share challenges and lessons learned since the implementation of the DEIA committee
    a. explain best practices and strategies to:
    b. foster and increase awareness of DEIA issues
    c. collaborate and form partnerships campus-wide and within the community
    d. how to initiate and start your own diversity committee, plan, and statements

Program Conclusions:
The DEIA plan was completed and is now in the execution phase, monitoring progress and adjusting as needed. Our DEIA committee has been able to plan and implement initiatives:

- Coordinating programing
- Showcasing exhibits
- Diversifying collections
- Upgrading space utilization
- Building staff cultural proficiency and awareness
- Collaborating and forming partnering across the University

The DEIA committee is a recipient of the Library Excellence in Access and Diversity award 2024 (LEAD). We continue to monitor and evaluate the progress of the university and library diversity needs. This initiative has set the framework for strengthening campus-wide partnerships, continued diversification of collections, deeper integration of library resources and services into the curriculum, and space utilization improvements. The AT Still Memorial Library looks

Beyond the Survey: Collecting Well-Rounded Feedback from the Library Service Desk

Track(s): Innovation & Research Practice

Katherine Morley Eramo, Library Administrator, Hirsh Health Sciences Library at Tufts University

Katie Kidwell, Library Assistant Manager, Hirsh Health Sciences Library at Tufts University

Background:
Historically, surveys were our main method of collecting feedback, but participation was low. We either created large surveys on multiple topics that were too time consuming, or sent multiple small surveys which were overwhelming. The responses we did receive took too long to analyze, and we were slow to implement results. So, we started intentionally thinking: What do users want? What do users need? By weaving micro-assessments seamlessly into the library's day-to-day, and locating them at the central hub of the library—the Library Service Desk—we could find out what people wanted for library space, resources, and programming, and quickly act on that information.

Description:
Our first effort with a micro-assessment started with a broad question: "If you could have three wishes for the library, what would they be?" We introduced a month-long campaign where patrons submitted wishes in a box at the Desk. We encouraged outlandish and mundane responses alike. We received many specific and easily-accomplished requests, so when the next opportunity for feedback arose, we kept it simple and specific, again gathering info at the Desk.

Before purchasing furniture, we posted a board with pictures of options, providing dot stickers patrons could place on their favorites, and post-its for different suggestions. We quickly saw what patrons liked and made our purchases accordingly. After the success of these methods, we continued applying similar creative strategies.

We have also reworked classic assessments into micro-assessments. When upgrading our website, a brief UX survey specifically assessing our new navigation menu was posted at the Desk for people to answer quickly on the go. Additionally, the library conducts daily head counts and biannual Affiliation surveys to help influence library decisions.

With the wheel rolling, the library is now always asking and receiving information to inform our programming, services, and space.

Program Conclusions:
The wheel rolls around, propelling itself forward– the more we talk to patrons, the more information we get, the more we can change for the better, the more comfortable patrons feel approaching us, and so on. As a result of micro-assessments, we have been able to:

- Upgrade to furniture patrons preferred

- Hang signage to shape use of spaces like study rooms

- Increase frequency of our study break tea program

- Optimize website wayfinding

- Purchase in-demand charging cords for new electronics

- Adjust library hours, such as shortening Desk hours before winter recess

We have worked to develop a reputation of being very responsive; we ask questions of patrons to influence library happenings, not just for the sake of asking.

Bridging the Gap: Engaging the LIS Student in Health Sciences Librarianship

Track(s): Professionalism & Leadership

Annisija Hunter, MLIS Student, University of Missouri

Sarah Villere, Senior Medical Librarian, Saint Joseph Hospital, Intermountain Health

Background:
Newly graduated LIS students are often inadequately prepared for a job in health sciences librarianship, and opportunities for students to engage with the field prior to graduating are still relatively rare. Despite this gap in the pipeline, health sciences libraries continually need to attract, retain, and effectively train a talented workforce. As background, this poster will consider evidence supporting the need for engaging students in professional settings prior to graduation and review various engagement opportunities that currently exist for students. This poster will focus on one existing award, in which a group of LIS students receive funding to attend and present on an NLM product at a professional conference.

Description:

The poster will be presented through the lens of the Student Development Award winners, and will describe the key components of the program: the application process, PubMed search training, one-on-one mentoring, a coaching session with library leadership, presentation creation, conference participation, and post-conference reports. A timeline of these components will also be included. Using visual graphics, we will show the amount of funding awarded to each participant, and how it was spent. To evaluate the program and determine its impact on the students and the organization, the poster will use the students’ post-conference reflections, discussions with the award coaches, and feedback from the program manager. The poster will highlight key quotations from main stakeholders for visual appeal.

Program Conclusions:
We expect to demonstrate that professional development programs will benefit both LIS students and health sciences librarianship organizations, leading to more interest in the field and qualified applicants in early career opportunities. In order to do this, the poster will heavily feature student outcomes: field-related skills and knowledge development, increased involvement in the field, networking, and increased student confidence and interest. The poster will also feature outcomes from program organizers: the creation of more qualified potential employees, and broader awareness among students of their organization. We hope that lessons learned from programs like the Student Development Award can inform and influence the creation of other unique opportunities for students and improve the future of student engagement.

Bridging the Virtual Gap: Expanding Online Library Access for Alumni

Track(s): Information Management

Nita Mailander, Grand Canyon University

Background:
The University Library serves campus and online nursing and health sciences students ranging from pre-licensure to the doctoral level with a growing alumni population. In support of lifelong learning, the library initially explored expanding online resources remotely to alumni in 2014, which included access to the CINAHL Alumni database. Over the years since the initial launch of alumni online resources, the library has incrementally grown access to alumni content to currently include remote access to fifty-six databases in support of the library’s mission to enrich academic success, scholarship, and lifelong learning.

Description:
With the launch of alumni library remote access in 2014, the library added a hosted EZProxy server to enable remote alumni library authentication to online resources. Providing IT infrastructure allows a seamless, user friendly, low staff maintenance way to provide remote library access for alumni. The library’s current alumni resources include access to fifty-six databases, which are highlighted on the library’s website, as well as on the university’s alumni website. Access to the library’s selection of alumni edition databases has been popular, with continued additional content requests from alumni for expansion. Regular evaluation of usage data for the library’s alumni databases occurs and assessment of potential new resources takes place at license renewal to determine what free or fee-based alumni access models exist that could continue to expand access.

Program Conclusions:
The online access to library resources is evidence of the library’s commitment to support alumni as they continue as lifelong learners after graduation. Whether an institution has face to face, online, or hybrid programs that are offered both virtually and in person, remote library access continues to support all alumni regardless of where they may be physically located. As library vendor business models change, it is important to regularly review options and license terms to ensure alumni access is evaluated for availability

and affordability.

Bringing Health Literacy to Communities Off the Road System

Track(s): Information Services

Anna Bjartmarsdottir, Professor, Alaska Medical Library, UAA/APU Consortium Library, University of Alaska Anchorage

Jennifer McKay, Associate Professor and Head, AK Medical Library, UAA/APU Consortium Library, University of Alaska Anchorage

Background:
In 2022, two medical librarians, at the largest publicly funded undergraduate and graduate four-year institution in the State, partnered with the area's community and university faculty on multiple grant-funded projects with the goal of positively impacting health literacy while combating misinformation. Each of these projects resulted in either curriculum enhancement, program development, or the creation of online materials which were distributed statewide. These projects attempted to alleviate health misinformation in a State, where more than 80% of its communities are inaccessible by road.

Description:
Despite the grant period ending, the work with the academic and community partners continued and future goals will focus on identifying ways to strengthen these partnerships.Grants focused on: 1. Providing evidence-based health information to health care professionals across the state either through in-person trainings or online presentations with the goal of reaching current clients of the region's major hospitals and rural clinics located off the States road system. 2. Translating public health information in 14 different languages around vaccine confidence in close collaboration with a local community group whose mission is to train multilingual community members on health literacy topics. 3. Creating a LibGuide with newly purchased monographs and freely available resources dedicated to health misinformation, which was added to a website developed by the State's library that any resident can access. Presentations were given to the State’s Library Association at the annual conference as well as for the Network of the National Library of Medicine (NNLM).

Program Conclusions:
All three grant projects have concluded but the collaborations are ongoing. Feedback is continuing to be measured by the number of times the items created have been accessed and the verbal feedback from collaborators.

Building an ILL Automation Workflow in a Small Hospital Library using Microsoft Forms and Power Automate

Track(s): Clinical Support, Information Services

Jonathan Ratliff, Library Services Program Manager, Samaritan Health Services

Background:
Without the budget for acquiring resource sharing management software, small hospital libraries may have difficulty managing interlibrary loan (ILL) workflows. Receiving, submitting, logging, and tracking ILL requests can be challenging. At our library, users had no way to submit requests except by email, and library staff tracked requests using a series of mailbox folders. This solution made difficult to track requests and

share requests statuses with multiple staff. The objective of the ILL workflow improvement project was to use existing software including Microsoft Forms and Power Automate to develop an online form for users to submit ILL requests to the library while also implementing an automated list to track the status of every request.

Description:
We developed the ILL workflow improvement in two stages over the course of approximately two months. First, we developed the ILL request form over several iterations, resulting in a two-question form that can be submitted in seconds directly from the library website. At the same time, we created the requests tracking list to mirror the information submitted with the form and added additional fields to track the requests status, transaction numbers, and other data. Secondly, we developed a routine in Power Automate that linked the form and the list together and provided convenience features such as email notifications. Implementing the solution was as simple as publishing the form on our website and sharing it with our users. Users quickly adopted the form, and while email requests are still popular, online form requests are increasing. The tracking list also makes it simple for library staff to add request that come from any source, creating a single source of truth on the status of every request. We evaluated the improvement through feedback from users and analysis of data on the source of requests.

Program Conclusions:
While this improvement will be an ongoing part of our services, we have successfully completed implementation. Response from users has been positive; the form saw its first uses within two days of going live on the website even though we had not yet publicized its availability. All feedback indicated that the form was clear and easy to use. Form use rose steadily over the course of 2023 from 10% of requests to over 37%. As we approach a year following deployment of the form, we plan to perform a complete evaluation of the impact of this new workflow. We also plan implement additional improvements to the form and list automation based on user feedback and the experience of library staff.

Building Author Services: Advancing Publication Support for Health Science Researchers

Track(s): Information Services

Sara Hoover, Scholarly Communications Librarian, The National Institutes of Health Library

Laura Abate, Director, Himmelfarb Health Sciences Library--George Washington University

Ruth Bueter, Associate Director, Library Operations, Himmelfarb Health Sciences Library--George Washington University

Background:
In the early 1990s medical libraries began to adopt a new research support role through the development of systematic review services. Today, there is an increasing need for health science librarians to serve as publication support navigators and to develop services that support additional phases of the research lifecycle. In 2020, our institution developed the Researcher Profile Audit Service which aimed to help researchers improve their profiles in systems such as Scopus, ORCiD, Web of Science, Google Scholar, and more. Since beginning the service, we have provided over 50 researchers with profile audit reports detailing recommendations for improving researcher profiles. In 2023, our library elected to build four additional research support services to provide additional assistance.

Description:
In Fall 2023, our library launched Author Services from [library name]. In addition to the Researcher Profile Audit Service, this program includes four additional services: Conference Poster Archiving, Journal

PreCheck, ORCiD Profile Evaluations, and DMS Plan Consultations. Our Conference Poster Archiving Service allows patrons to request digital archiving for conference poster PDFs in our institutional repository. The Journal PreCheck Service aids with journal selection to avoid submissions to predatory publishers. Our ORCiD Profile Evaluation Service provides a review of ORCiD profiles and recommendations for curation strategies. And our DMS Plan Consultation Service provides support and resource recommendations for creating a data management and sharing plan. While many of these services were already being offered through the form of scholarly communications consultations, developing a roster of services allows users to see the full range of publication support provided by the library.

To highlight these services we built a LibGuide with a tab for each service. We also designed a Google form for each service where library patrons request support and provide a description of their needs related to a specific service. A link to the Author Services LibGuide was added to our library website under the “Services” tab.

Program Conclusions:
We developed Author Services during the Fall of 2023 and these services will be offered to all faculty, residents and students served by the library. Outstanding questions remain about how to integrate author services with other services often provided by health sciences libraries such as 3D printing and Systematic Review support. Author Services provides one example of how information provided by medical libraries as part of the consultation process can be streamlined to provide visible institutional support.

Building Strong Bonds with Students: Creating an Award for Student Excellence in Library Research

Track(s): Information Services

Anna Krampl, Assistant Director - Macon Campus, Mercer University School of Medicine

Kim Meeks, Director, Mercer University

Background:
Since the decision to move the USMLE Step 1 exam to a pass/fail grading system, medical students are looking for ways to gain a competitive edge in residency programs, and many are seeking more research and scholarship opportunities they can include in their CVs. Medical libraries can be an excellent resource in this area and increase library awareness, use, and value by creating a Library Student Research Award.

Description:
In 2013, the Medical Library director and other library administration created the Student Excellence in Library Research. In the award's incipient year, submissions needed to include a statement of support from a faculty mentor, the completed research project, and a bibliography.In the intervening years, the submission criteria shifted to include a reflexive essay, which asked students to explain their outstanding proficiency in using library collections, services, and information-seeking skills. In 2022, the timing of the submissions changed, allowing for more marketing and student applications, which saw the number of submissions peak. The recognition of the award increased with the use of other promotional tools and with the help of Student Affairs, who included the winner in the medical school graduate honors ceremony. Winning essays included discussions on generative search strategies, and best practices concerning PICO and database searching. Most uplifting was the special attention paid to direct consultation with campus librarians.

Program Conclusions:
Traditional marketing of library resources and services can only go so far. Creating a library student research award can effectively boost student awareness and engagement with librarians, resources, and

services. Developing a library research award not only builds connections with the student body and other medical school departments like student affairs and graduate honors but also increases the library's visibility and emphasizes its impact on research and scholarship.

Clarity, Creativity, and Competition: Tailoring Library Research Instruction for High School Audiences

Track(s): Education, Information Services

Kelsey Cowles, University of Pittsburgh, University of Pittsburgh

Rachel Suppok, Research and Instruction Librarian at University of Pittsburgh Health Sciences Library System

Background:
This class is presented annually to approximately 80 high school students participating in a summer research program at our university. The class is expected to teach students the fundamentals of academic research from constructing a research question to searching for literature to appropriately citing materials in a research paper. In order to accomplish this, we designed an interactive workshop that includes lecture portions as well as opportunities for students to practice searching reliable health sciences resources such as PubMed, the library catalog, and MedlinePlus. The workshop concludes with a game-based evaluation that is simultaneously fun for the students while also providing an opportunity for librarian instructors to clear up any remaining areas of confusion.

Description:
In 2022, we inherited responsibility for the library's partnership with the summer research program from a retiring librarian. Previously, the class had consisted of a series of recorded video lectures accompanied by worksheets and a Jeopardy-style review game. We opted to revamp the session by replacing the recorded lectures with live instruction to facilitate active learning and enhance learner engagement, updating the worksheets to utilize more relevant examples, and transforming the review game using Kahoot!, an online game-based learning platform that most high school students are already familiar with. We also clarified learning objectives and overhauled the content to better align with these. In 2023, a library intern collaborated with the library's instructional designer to expand and improve the portion of the class dedicated to citations.

After attending our class, participants return later that week for a hands-on working session during which they refine their research proposals and begin to search for literature with librarian assistance. Our primary means of evaluating student learning is the Kahoot! review game; we also informally assess students' ability to implement this knowledge during this working session. Unfortunately we are not currently provided access to students' final research projects to perform a truly summative assessment.

Program Conclusions:
As a result of the changes described above, we have observed a substantial increase in student engagement throughout the session, noted improvements in students' research proposals, and received positive comments from librarian instructors on the session's perceived quality. We continue to refine the class and update the content each year in response to assessment data and qualitative feedback from students and instructors.

Collaborating for Strength and Knowledge: A Tribal Libraries Program Field Trip to an Academic Health Sciences Library

Track(s): Health Equity & Global Health, Information Services, Professionalism & Leadership

Deirdre Caparoso, Outreach and Community Engagement Librarian, University of New Mexico

Cassandra Osterloh, Tribal Libraries Program Coordinator, New Mexico State Library

Deborah Rhue, Clinical Services Librarian, University of New Mexico Health Sciences Library and Informatics Center

Tracy Garcia, Santo Domingo Pueblo Library, Santo Domingo Pueblo Library

Background:
When the library’s Outreach and Community Engagement Librarian started her position, she sought to establish relationships with libraries throughout the state in accordance with the library’s mission of providing access to health information to all state citizens. After a conversation with the state’s Tribal Libraries Program Coordinator, the librarian developed an all-day seminar to support the health information needs of the state’s indigenous populations by providing training to library staff. Attendees had the opportunity to visit the library and attend sessions offered by multiple health sciences university programs. This poster will describe and review the seminar from multiple perspectives: the Outreach and Community Engagement Librarian, the Tribal Libraries Program Coordinator, a presenter, and a tribal libraries participant.

Description:
The all-day field trip to the health sciences library by the tribal librarians was a first for the library and participants. The Outreach and Community Engagement Librarian developed a schedule, invited speakers from multiple programs, and took care of logistics like working with administration on parking, swag, lunch, as well as seeking internal library staff volunteers to assist. The Tribal Libraries Program Coordinator worked with tribal library staff around the state to determine best dates and needs, as well as functioned as a liaison between the Outreach and Community Engagement Librarian and the tribal public libraries. University librarians from two health sciences libraries presented sessions on PubMed, MedlinePlus, and a Native health database. Local relevant organizations presented information on community health extension programs and a university-sponsored center for Native American health. This was an opportunity for the library to reestablish its relationships with tribal libraries and offer valuable training.

Program Conclusions:
The library would like to offer training days like this again in the future, both to the same group and other libraries throughout the state. Participants had the opportunity to learn about reliable health resources they could pass on to their library patrons and communities. Several tribal library coordinators from around the country have expressed interest in the specifics of the field trip and training in order to reach out to their respective health sciences libraries. Future goals include conducting a survey to assess community needs and feedback about programming.

Cost Savings Through Interdisciplinary Collaboration Implementation of a Clinical Solutions Resource

Track(s): Information Management, Professionalism & Leadership

Brittany Haliani, Director, RWJBarnabas Health

Jeannine Creazzo, Director, Medical Library, Continuing Education, and Research, RWJBarnabas Health

Background:
In 2022, the Senior Vice President (SVP) for The Center for Professional Development Innovation, Research and The Institute for Nursing Excellence, saw redundancy in on-line clinical resources across the system and took the opportunity to review, research and assess these resources to increase standardization. In collaboration with the Library Directors of both Cooperman Barnabas Medical Center (Cooperman) and Robert Wood Johnson University Hospital Somerset (Somerset) and other key stakeholders the decision was made to replace and consolidate resources. The journey began to replace the Nursing Reference Center (NRC) and Elsevier Clinical Skills with one, more comprehensive resource for both nurses and allied health professionals.

Description:
Historically, five of the system hospitals licensed Clinicalkey independently. Two library Directors negotiated their individual Clinicalkey license and oversaw usage. The library Directors observed a need to eliminate individual licensing and initiate a group license for the five hospitals with the intent elicit cost savings. In 2023, a system based library advisory group was created. Membership included Chief Academic Officers, CMOs and Library Directors. The library Directors analyzed usage statistics, calculated yearly percentage increases and projected savings for each campus. Library Directors presented their analysis to the advisory group in addition to system corporate purchasing and legal departments. Then, Directors worked with Elsevier to create a library group licensing contract, a first for the system. This project demonstrated how two librarians collaborated with multidisciplinary stake holders to provide a cost savings opportunity for multiple hospitals.

Program Conclusions:
This project initiated a review of other licensed library products, which have a potential cost savings for the future.

Creating a Data Repository Finder with a Little Help from Our Friends: Assisting Researchers and Managing Volunteers

Track(s): Information Management

Ummea Urmi, Project Coordinator, NYU Langone Health

Collin Schwantes

Nicole Contaxis, Head of Data Sharing and Metadata Management, NYU Langone Health

Background:
With the new update to the NIH Data Management and Sharing Policy (DMSP), more researchers will now need to find repositories to share their data to be compliant with the new DMSP. Therefore, we created a Data Repository Finder (DRF) to help researchers locate an appropriate NIH-supported repository based on their data storage requirements for the study, such as data type, content type, data access, submission fee, submission restrictions, subject of study, and data embargo. Because of the vast number of NIH-supported repositories available and the amount of metadata related to the repositories that needed to be verified before creating the DRF, we needed to recruit volunteers to help collect and clean data for these repositories.

Description:

DRF was built using Drupal, the open-source software behind the Cornell Data Storage Finder. A small group of volunteers first investigated the metadata about repositories included in re3data and NIH BioMedical Informatics Coordinating Committee (BMIC) websites to see which metadata was common and most useful to researchers when finding a repository. After establishing the metadata they wanted to collect, the metadata from re3data was pulled from the API to act as a starting point. In order to clean, supplement, and verify the metadata from re3data, a larger group of volunteers was necessary. Therefore, we created a sign up form to recruit volunteers from members of the NIH DMSP Working Group and Data Discovery Collaboration listserv to collect contact information, the number of repositories they wanted to clean, and interest in a training session. Based on the responses, we assigned volunteers specific repositories to clean and held a virtual training session on the data clean up process. Volunteers received reminders to keep the project on track for launch to coincide with the implementation date of the DMSP. After data was cleaned, the metadata for each repository was manually entered in the Drupal system.

Program Conclusions:
Altogether, 17 people cleaned and collected metadata for 85 repositories. On average, 5 repositories were cleaned by each person. This process of creating workflow for data cleaning, recruiting volunteers, holding a training session, and answering volunteer questions were time consuming. The training session covered the data cleaning and collection process as well as criteria for any repository to be included in the DRF. Collaboration with volunteers for metadata clean up made the clean up process faster and more manageable. Now, the DRF is complete and publicly available online. In the future, we plan to add more repositories to the Drupal system and perform outreach to inform researchers about the DRF.

Creating a Library Video Working Group to Improve the Quality and Consistency of Online Video Tutorials for an Academic Health Center.

Track(s): Education

Brandon Wilkinson, Health Sciences Education & Research Librarian, Oregon Health & Science University

Background:
This poster will present how a Library Video Working Group was formed to address the quality and content of library video tutorials.

Description:
To better serve our user population, the library formed a working group to create process flows for library branded instructional video tutorials. The goal was to improve the quality and consistency of our tutorials and provide a framework to help prioritize and schedule video creation. This poster will outline how a project charter was created, the development of the processes that were implemented, and the final deliverables in the form of an internal video creation library guide for our colleagues, as well as an outward facing guide for our growing collection of videos on database search, research access, and data management. It will also describe the creation of an ongoing process for soliciting suggestions for future videos and prioritizing in which order they are created using a shared editorial schedule.

Program Conclusions:
The Library Video Working Group was able to follow the original design of the developed charter to create a process flow for our library videos, disseminate that process to our colleagues through a libguide, and use it to create a growing collection of high-quality branded video tutorials for our user base. Evaluation method: Usage numbers of views through our library’s institutional YouTube channel.

Creating Effective Training Materials for a New Instruction Tracking System

Track(s): Professionalism & Leadership

Sara Samuel, Informationist, University of Michigan

Kate Saylor, Informationist, Taubman Health Sciences Library, University of Michigan

Jamie Niehof, Engineering Librarian, University of Michigan

Background:
A new online system was created to facilitate tracking instruction activities that happen at the library. All library instructors are able to use the system to fully schedule instruction events that they are leading or they can use it to just track statistics for any instruction they perform. The authors of this paper developed training materials for all library employees to help them understand how to use the new instruction system.

Description:
In order to provide the most effective training materials, we recognized that library instructors would be interacting with the system to complete specific tasks including: creating a new instruction session, claiming an instruction session, entering statistics, and pulling reports about instructional activities. We decided that the training information should be disseminated via a digital handout accompanied by short videos, with a follow up training session via Zoom to answer questions. This approach is scalable, lets individuals determine the amount of interaction they want with the training materials, and provides resources to revisit as needed.

We created an online handout in Google Documents with step by step instructions for how to do each task, including screenshots. Five short videos were also created to provide an orientation of the new system and show “live” demonstrations of each task. The handout and videos were widely shared once the new system was live so that library instructors would know how to use it, and a training session was scheduled for the following month. The goals of the training session were to share how the new system was different from the old system and to answer any questions about using the new system.

Program Conclusions:
The videos were an effective way to introduce librarians to the new system, with a total of 83 views across the 5 videos prior to the training session. The Orientation and Creating a Session videos were the two highest viewed videos, with 24 views and 23 view respectively. The handout had 55 unique viewers prior to the training session. There were 6 questions submitted ahead of time to be answered during the training session and there were over 40 attendees at the training session. Several librarians expressed their thanks for the training materials including this email message: “I want to say that I just went through most of the video training, and it is excellent! Thanks so much!”

Cultivating a Search Hedge Repository for a Systematic Review Service

Track(s): Information Management, Information Services

Shenita Peterson, Public Health Informationist, Emory University

John Nemeth, Clinical Informationist, Emory University Woodruff Health Sciences Center Library

Mia S. White, Medical Education & Technologies Informationist, Woodruff Health Sciences Center Library, Emory University

Sharon Leslie, Nursing Informationist, Woodruff Health Sciences Center Library

Background:
Since 2019 a R1 health sciences library has offered researchers a formalized two-tiered Systematic Review Service (SRS). The SRS team consists of 6+ Informationists who have co-authored over 30 articles since its inception.

The team noticed a significant overlap in the search concepts research teams were investigating. To minimize redundancy of effort the SRS team decided to create an internal search hedge repository.

Taking inspiration from search hedge pages published by McMaster University and the Lister Hill Library at the University of Alabama at Birmingham, we began to craft and cultivate a repository that met the needs of our SRS team.

Description:
The Hedge Development team consisted of members of the Systematic Review Service. The hedges the team decided to develop were recurring concepts from previous systematic review projects or those which presented unique challenges. A sample of some hedge concepts include climate change, elderly, mental health, cancer, and social determinants of health.

An online library research guide was developed that categorized the topics by the closest MeSH term. For example, the Persons heading contains the search hedges for Elderly, Indigenous Persons, and Pregnant Persons. Each page contains a tab with the developed PubMed search and a Keyword search tab. A notes field shows the date of the most recent review by the team. Scope notes explain any significant conditions or omissions that may impact use of the hedge. Additional optional or historical terms to consider are also noted. Each entry states that the hedges are not validated and any terms that were obtained from other libraries are noted. The research guide is publicly available.

Program Conclusions:
Our team created uniformity and standards for terms we routinely search for in systematic reviews, thus increasing our productivity and reproducibility by eliminating search reconstruction for frequently used concepts. We made our hedges available to the public through a research guide with the understanding that although our searches have not been validated, they still have value as a resource for researchers.

Data Deep Dive: Strategic Analysis for Improving Reference Statistics

Track(s): Information Management, Information Services

Jessica Petrey, Associate Director, Clinical Services, University of Louisville

David King, Web & Technology Librarian, University of Louisville Kornhauser Health Sciences Library

Background:
Libraries rely on reference statistics to assess their services, track progress towards strategic initiatives, better plan to meet the evolving needs of users, and provide quantitative evidence of value when communicating with administration and stakeholders.

Meaningful interpretation of statistics for any of these purposes is dependent on complete, consistent, and accurate data entry by library personnel; recognizing the integrity of our own reference statistics had been compromised in all three areas, our library sought to improve our data collection process. This poster

illustrates the process by which we assessed our activity tracking and reporting needs, key problems we identified, changes we implemented as a result, and lessons learned along the way.

Description:
We manually reviewed the collection form for obsolete to remove from menus, as well as categorizations needed to accurately capture current services. Fields and field content were carefully reviewed for redundancy and were merged or renamed for clarity as appropriate. Reports were generated for select fields to assess if and how frequently the menu items were selected. Reports were also run to identify use cases for any selection of “Other,” and blanks and null values were investigated to identify sources of inaccurate or missing data. Settings were adjusted to ensure fields relevant to all activities were required while optional fields were used sparingly to collect information applicable only for certain transactions. Text or numeric entry fields were replaced with selectable menus to minimize risk of typographical errors. In addition to the data-driven changes, categorization was updated to align with reporting requirements of administration, external organizations, and other stakeholders, and feedback from library personnel was used to prioritize clarity and usability. A comprehensive guidance document was developed to facilitate training and standardize understanding of data fields and input options, and a group session was scheduled to orient the team to the updated process.

Program Conclusions:
Preliminary results indicate reduced data entry errors and more accurate generated reports. Improved usability reduced time required to track activities, and aligning with reporting needs facilitated sharing of exported data with minimal cleanup.

Librarians and staff reported increased satisfaction with usability, specifically citing the elimination of redundant or unused variables, decreased overall length, less scrolling with streamlined menus, more logical field organization and wording, and less ambiguity between input options.

Further day-to-day use of the form will allow us to further assess and adjust the form, providing a more accurate overall appraisal. We anticipate that the improved form will facilitate higher quality data collection while requiring less time away from our librarians’ primary responsibilities.

Designing an Evidence-Based Health Care and Medical Informatics Longitudinal Course during UME Curriculum Renewal: UME 1 Program Results

Track(s): Education

Elizabeth Irish, Associate Professor, Schaffer Library of Health Sciences, Albany Medical College

Enid Geyer, Associate Dean for Information Resources and Technology; Associate Professor, Schaffer Library of Health Sciences, Albany Medical College

Rebecca Keller, Director, Medical Education; Professor, Department of Molecular and Cellular Physiology, Albany Medical College

Timothy Palmieri, Assistant Professor, Emergency Medicine, Albany Medical College

Traci Tosh, Assistant Professor, Schaffer Library of Health Sciences, Albany Medical College

Background:
Library faculty (librarians) collaborated with clinical faculty and Medical Education leadership to combine a longitudinal medical informatics course and a longitudinal evidence-based health care course into one

mandatory four-year longitudinal undergraduate medical education (UME) course. Led by a librarian, the planning team created the four-year course framework, then focused on the UME 1 course scheduled for the 2022-23 academic year. The goal of the course is to teach clinical epidemiology principles within an evidence-based medicine framework, including the use of best available evidence resources.

Description:
The team began the course development process by reviewing the sunsetting courses’ syllabi, STEP 1 content coverage, and student evaluations to develop measurable goals and objectives that met the medical college’s goals. Team-based learning (TBL) was selected as the teaching methodology. Sixteen TBL modules were developed to encompass basic clinical epidemiology, critical appraisal, and information retrieval and management principles. Each TBL session was facilitated by a clinician and a librarian. Prior to each session, students submitted an individualized readiness assessment test (iRAT). During the TBL sessions, groups of six students completed the general readiness assessment (gRAT) and the learning activity. A final review session prior to the NBME based exam was offered. The grading breakdown for the pass/fail course was determined by iRAT score (20%), interim exam (30%), and final exam (50%).

Program Conclusions:
The results of the first cohort of students will be presented. Preliminary data includes final course exam results, the Comprehensive Basic Science Examination (CBSE) biostatistics questions results, and CBSSA Comprehensive Basic Science Self-Assessment (CBSSA) diagnostic results. While STEP 1 scores will not be available yet, the college’s internal post-STEP I survey will include a question on the students’ preparation for the biostatistics component. Initial course evaluations indicate an increase in student satisfaction. Anonymized student comments will be incorporated as available. The value of librarians and clinicians co-facilitating the TBL sessions will also be addressed. Finally, course revision based on student and faculty feedback will be addressed.

Designing Tools and Resources to Support Liaison Librarian Success

Track(s): Information Services, Professionalism & Leadership

Courtney Pyche, Public Health Liaison Librarian, University of Florida

Alyson Young, Associate University Librarian, University of Florida

Michele R. Tennant, Bioinformatics Librarian at University of Florida, Retired

Hannah Norton, Chair and Fackler Director, University of Florida

Lauren Adkins, Assistant University Librarian, University of Florida

Background:
This academic health science center library serves the 6 colleges, programs, centers and institutes affiliated with the Health Science Center. The liaison model is unit-based, designed to meet the information needs of clients effectively. Results from an internal study indicated additional infrastructure and administrative support is necessary to improve efficiencies, skillsets, and job satisfaction. A team of liaison librarians came together to design resources and activities to provide such support, including training and mentoring, standard templates and branding for communication, and other administrative support.

Description:
Based on the results from an internal survey, a team of 4 liaison librarians came together to identify methods and resources needed to overcome the challenges and barriers to success that liaisons faced. Survey results suggested that liaisons found many positives to the liaison librarian model, but continued to have

concerns with workload, burn out, perceived lack of respect from clients, training deficiencies, and related infrastructure. The team collaborated to develop recommendations in the areas of training, mentoring, branding and communication, and administrative support. The list was shared amongst the liaison librarians and an open discussion was held virtually to identify priorities and relevant stakeholders and any additional requests for support. The team will begin drafting trainings and communication templates to implement in 2024. After a semester of usage, the resources will be evaluated through open discussion within the liaison group and an anonymous survey.

Program Conclusions:
The results of the liaison librarian perceptions survey demonstrate how important periodic assessment is for even programs that are long standing and deemed successful. The team expects to conduct feedback sessions and an anonymous internal survey with the broader in-unit liaisons to assess improvement in job satisfaction, workload management, and the usefulness of the tools. Results will be analyzed and discussed to continue meeting the professional needs of liaison librarians.

Developing a Pathway to MLA Consumer Health Information Specialization

Track(s): Education, Information Services

Carolyn Martin, Outreach and Education Coordinator, Network of the National Library of Medicine, Region 5

Jamia Williams, Assistant Librarian, University of Utah

Bennie Finch, Education and Outreach Coordinator, NNLM Region 7

Bobbi Newman, Community Engagement and Outreach Specialist, Network of the National Library of Medicine

Jen Ortiz, Health Literacy & Membership Specialist/Librarian for NNLM, UNT Health Science Center

Molly Knapp, Training Development Manager, University of Utah

Background:
As health information professionals, we recognize that accurate health information not only enhances the quality of life but can also be a lifesaver. Medical librarians may know that MLA’s Consumer Health Information Specialization offers training in providing health information services to consumers and recognition for the accomplishment of acquiring new health information skills. Responding to user feedback, NNLM created a set of on-demand modules covering each of the five essential competencies for providing Consumer Health Information making it even easier to get started and acquire the skills and knowledge needed to become a confident, expert provider of health information.

In this presentation, participants will learn about why the class was developed, the types of activities utilized, and class evaluation outcomes.

Description:
The Medical Library Association has outlined eight core competencies and two levels of specialization in the Consumer Health Information Specialization (CHIS).

The goal of CHIS on-Demand is to support easy tracking of the Level I competencies and more flexibility for library and information workers. The team drew from existing classes and integrated new content with an emphasis on addressing a variety of learning approaches. Through self-paced readings, interactive

exercises, quizzes, and videos, five separate modules cover critical core content related to Level I CHIS competencies. Learners can take any or all modules and in any order. Offering a time agnostic class format (on-demand) that focuses on the ‘need to know’ for consumer health information provision removes barriers of access, time zones, connectivity, and scheduling, allowing more learners to access training content.

By supporting all types of libraries to complete CHIS, we support health information access, encourage competent health reference, and support the National Library of Medicine’s goal of reaching “more audiences in new ways”.

Initially, the content was opened to selected users for class evaluation feedback and used in an iterative process to improve the modules. We have continued to incorporate learner feedback to improve the class over time.

Program Conclusions:
Class evaluations are available for each of the five modules. Between June 2022 and November 2023, 776 evaluations were completed. The results were overwhelmingly positive but there have been suggestions that have been incorporated over time through class updates. Some of the key results have been that 95.5% of evaluations indicated that learners would “share skills or resources learned with others” and 94.5 % of evaluators indicated that what they learned could “support my end users’/patrons needs more effectively”. Feedback continues to be included in class updates and a new set of modules to support CHIS Level II is currently under development.

Developing Culturally Competent Consumer Health Literature to Support International Missions.

Track(s): Health Equity & Global Health

Jamie Quinn, Director of the Nursing Learning Resource Center, Baylor University Louise Herrington School of Nursing - Learning Resource Center

Background:
Baylor University, Louise Herrington School of Nursing (LHSON) engages in Global Health Missions as part of the nursing student experience. The missions serve to provide students cross-cultural skills, engage them in learning cultural humility, and give them experience in practicing compassionate nursing care. The health mission identified to support with the Librarian’s without Borders Ursula Poland International Scholarship, was the Nursing to Ndola, Zambia mission trip. Baylor LHSON partnered with Northrise University (NU) located in Ndola, Zambia, for this interprofessional mission trip named Impact Ndola. The LHSON nursing team had opportunity to serve the Ndola community through community-wide outreach involving university students, staff, and faculty along with partners from around the United States.

Description:
The Baylor Nursing Librarian worked with the mission trip lead faculty and students to create culturally appropriate consumer health literature for the populations served to support education provided during Health Fairs for the Northrise University campus community. The consumer health scholarship completed two goals; one, to support the healthcare mission trip specific to Impact Ndola by identifying quality consumer health literature freely accessible that encompassed culturally competent and medically appropriate consumer health education to support infection prevention and control, oral hygiene, and health in children receiving physical health screenings. The second goal supported the Ndola Health Fair by identifying and making printed materials available that deliver culturally competent and medically appropriate consumer health education. Posters were created by LHSON students in collaboration with the health science librarian on consumer health topics. Further collaborations and finalization of educational content took place between LHSON students and Northrise University faculty, students, and staff. Consumer health

topics included non-communicable diseases such as cardiovascular disease, diabetes, breast cancer, cervical cancer, and other diseases. The educational material was utilized by LHSON and NU School of Nursing students to develop multiple podium presentations.

Program Conclusions:
Scholarship supporting community education to Ndola, Zambia residents was successful as students created three posters and presented additional information at health fair podium presentations and educational booths. Consumer health information related to Women’s Health was collected from the World Health Organization and mental health information was collected from the National Library of Medicine’s MedlinePlus, NIH Mental Health, Mental Health America, and Substance Abuse and Mental Health Services Administration (SAMHSA). Consumer health information related to upper respiratory infections was collected from American Academy of Pediatrics, American Academy of Allergy Asthma & Immunology, and the National Library of Medicine’s MedlinePlus.

Developing Research Literacy Skills in Dietetics and Nutrition Interns: Pilot Rotation to Meet the ACEND Competencies in Evidence-Based Practice

Track(s): Education

Jaclyn Morales, Senior Librarian, North Shore University Hospital

Farhana Ahmed Ahmed, Senior Manager, Food and Nutrition Services,, North Shore University Hospital

Luz Elena Valencia-Penagos, Manager, Northwell Health Dietetic Internship, North Shore University Hospital, Northwell

Background:
The Dietetics and Nutrition internship program at a large, academic quaternary hospital, elicited the support and services of the health sciences library to develop a structured seven-week research rotation. The primary goal was to ensure all interns met the Accreditation Council for Education in Nutrition and Dietetics (ACEND) competencies in evidence-based practice. Secondary objectives included enhancing interns' confidence in participating in research or quality improvement projects and improving research literacy skills.

Description:
The health sciences librarian and the manager of the Dietetic and Nutrition Internship program reviewed the structure of an existing four-week research curriculum developed for first year Anesthesiology residents by the health sciences librarians in 2022. The curriculum was adapted to fit the scope and goals of the seven-week research rotation for the internship program. A combination of live remote instruction and asynchronous learning modules, hosted on a LibGuide, covered topics on developing a research question, organizing a quality improvement project, literature searching, citation management, critical appraisal and writing an abstract. At the beginning of the rotation, a quality improvement project was assigned to each intern, which allowed them to actively apply the skills acquired during the rotation to the project. The first three weeks of the rotation featured remote learning sessions with the health sciences librarian and the remaining four weeks focused on asynchronous learning with regular communication between the interns, internship manager, librarian and program mentors. Pre- and post-evaluations were used to assess the interns’ confidence in meeting the ACEND competencies in evidence-based practice; familiarity with the research process and library resources; confidence in participating in a research or quality improvement project.

Program Conclusions:
Based on both pre- and post-evaluations, the Dietetic and Nutrition interns (n=9) expressed enhanced confidence in meeting ACEND competencies related to evidence-based practice, improved research literacy skills, and increased assurance in participating in research or quality improvement projects. An analysis of the open-ended feedback indicated that collaboration and communication with the librarian, internship manager, mentors, and registered dieticians were key benefits to the program structure. The curriculum, reflective of these positive outcomes, will be integrated into the internship year of 2024. The development of the research curriculum for the Dietetics and Nutrition internship program exemplifies how educational collaborations with residency programs can be adapted to support educational objectives of allied health programs.

Development and Implementation of a Book Discussion Group in a Consumer Health Library

Track(s): Education

Jackson Wright, Health and Wellness Library Assistant, Virginia Commonwealth University

Dana Ladd, Health and Wellness Librarian, VCU Libraries

Background:
Health programming is an important service consumer health libraries provide to promote health literacy, the library, and its resources. Staff at a consumer health library that provides monthly health and wellness programming wanted to host health-themed book discussions aimed at not only patients and family members, but also healthcare providers, health sciences students, and graduate and undergraduate students. In the summer of 2021, the librarian and staff of the library planned and implemented a book discussion group open to anyone.

Description:
The library staff first identified topics and themes of relevance to its library visitors that coincided with health month observances. Next, they examined the National Network of the Library of Medicine’s Reading Club website for ideas for book ideas. Staff developed a list of topics and books and scheduled quarterly meetings. After they selected books, they marketed the book discussions via university mass mail, health system intranet, program calendar, the library webpage, and social media. After reading the books, library staff created discussion guides and questions. They offered the book discussion programs via Zoom during the COVID pandemic and then in-person. The library assistant led the book discussion and gave everyone an opportunity to engage. Each discussion began with an overview of the book and an ice-breaker question. The book discussion leader encouraged conversation amongst participants around relevant themes.

Program Conclusions:
Staff conducted three book discussions virtually via Zoom and two in-person. On a scale from 1 (poor) to 5 (great) twelve participants rated the book discussion a 5 and five rated the program a 4. Fifteen participants responded that they are very likely to attend another book discussion; one responded likely, one neutral, and two responded very unlikely. Many participants conveyed gratitude verbally after the discussion or in the survey comment box. One participant from another state wrote that they were glad to be able to attend virtually via Zoom. Staff learned a lot from our early book discussions and overall, the book discussions were a successful way of inviting people into the consumer health library space.

Enhancing User Experience of an Institutional Data Catalog Using Contributor Survey Data

Track(s): Information Management

Ummea Urmi, Project Coordinator, NYU Langone Health

Michelle Yee, Research Data and Metadata Management Librarian, NYU Langone Health

Nicole Contaxis, Head of Data Sharing and Metadata Management, NYU Langone Health

Background:
A medical library at an academic medical center developed a data catalog to help researchers locate and share data for re-use and collaboration. The data catalog consists of datasets that are generated at the institution as well as external datasets, and the datasets include a main contact person for any questions related to their datasets. These main contact people are either the corresponding authors for internally generated datasets or local experts for external datasets. A survey is sent out to these corresponding authors and local experts each year to request feedback regarding their experience with the data catalog. The feedback is then used to improve and update the data catalog to enhance their future experience as contributors to the platform.

Description:
The data catalog team starts working on the contributor survey at the beginning of January each year. This process begins with finalizing a list of ten survey questions, which ask contributors to review their associated datasets, describe their data sharing experiences, and suggest new enhancements for the data catalog. Two to three new questions are rotated in every year to ask for additional insight into their obstacles to data sharing, perspectives on data discovery, and more recently, data sharing plans following the implementation of the new NIH Data Management and Sharing Policy. Contributors are also asked if they would like to volunteer to provide in-depth feedback through a one-on-one discussion. The survey is published in a REDCap form and distributed via email to corresponding authors and local experts. The data catalog team sends two rounds of reminders to contributors. Then, all the responses are reviewed to understand their experience for the past year.

Program Conclusions:
While the data catalog team observed no trends in the quantitative data between the years 2018 and 2023, they have relied on qualitative feedback to help inform design and workflow decisions. Quantitative data provided information such as whether researchers had positive experiences with data sharing, whether they began new collaborations, or whether they experienced barriers to data sharing, and no patterns have been noted with this data. Qualitative data has helped inform decisions that frequently focus on how to ease contributor burdens and pain points such as describing data, responding to access requests, and responding to questions about the data. The team is currently performing thematic analysis on the qualitative data and will share these results in the finalized poster.

Evaluating Library Data Services Consultations

Track(s): Innovation & Research Practice

Lucy Carr Jones, Research Data and Scholarly Communications Librarian, University of Virginia Health Sciences Library

Andrea Denton, Research & Data Services Manager, Claude Moore Health Sciences Library

Hannah Pollard, Scholarly Communication Informationist, University of Colorado Anschutz Medical Campus

Background:
The [Redacted] Health Sciences Library had offered consultations in data management, data analysis, and statistics for seven years, but the library had not previously conducted an assessment of this service. Though many models exist for library workshop surveys, we could locate few examples of patron surveys regarding consultations and none regarding data services consultations specifically. In Fall 2023, we distributed a pilot Qualtrics survey to a random sample of patrons who had requested consultations via our ticketing system in the previous seven months. With the results of this pilot, we refined the survey and will distribute it to our larger sample of patrons in Spring 2024.

Description:
To develop the survey, we first searched the literature for articles on the assessment of consultation services in libraries. Then, we put out a call on research data mailing lists, requesting examples of any surveys on data consultations that librarians had conducted but may not have published. Neither effort yielded any examples similar to what we had planned. We developed a 15-question survey to assess patrons’ impressions of their consultation, including the best features of the consultation, whether they understood the concepts the data specialist or librarian discussed, and what they would have done if this service had not been available. Additional information such as how they found out about the service and why they requested a consultation was also collected. Nine patrons responded to the pilot survey out of 30 contacted (a 30% response rate) and the final survey questions were adjusted based on their responses.

Program Conclusions:
The final survey questions have been prepared and an IRB application is underway. We plan to distribute the survey to library patrons this Spring and present preliminary results and lessons learned from the project at MLA ’24. The survey results will be used to improve data services consultation offerings as well as analyze their impact on our patron base.

Expanding Content in a Health Sciences Library Discovery Service: Changing the Default Search Scope in a Shared Consortium Environment

Track(s): Information Management, Innovation & Research Practice

Deborah Wassertzug, Reference and Instructional Librarian, Himmelfarb Health Sciences Library--George Washington University

Sara Hoover, Scholarly Communications Librarian, The National Institutes of Health Library

Brian McDonald, Web Services Librarian, Himmelfarb Health Sciences Library--George Washington University

JoLinda Thompson, Systems Librarian, Himmelfarb Health Sciences Library--George Washington University

Background:
Many institutions have recently adopted shared library service platforms and discovery platforms that search across multiple libraries at an institution or between multiple libraries that participate in a regional consortium. For medical libraries, this consolidation process engenders questions related to what content pools to make available for discovery to best serve users with health science specific research needs. Is it

more effective to set a default search scope that allows users to see the full range of shared content or to tailor a search scope that prioritizes health science specific content? Our library Assessment Committee evaluated two potential discovery search scopes and made recommendations that could potentially benefit library users.

Description:
In 2018 our library joined a regional academic consortium and transitioned to the Ex Libris Alma/Primo VE platform. This platform allows system administrators to set up multiple discovery search scopes with one designated as the default. Currently, our library uses a default scope which includes local catalog content (our physical collections and purchased e-books) and Primo CDI content (subscription electronic and open access content). Changing the default scope could potentially increase awareness and enhance the use of the consortium loan service by library users and improve relevant results for some searches. The Library’s Assessment Committee designed a qualitative usability testing project that compared results from the default scope and a search scope which includes print content from other consortium institutions. Committee members developed a testing set representing 30 typical searches in the discovery platform, performed the searches in each scope, and compared the results. The scopes retrieved largely the same CDI content, while the consortium scope returned more potentially relevant print content for some searches. The committee recommended switching to the search scope that includes consortium holdings as the default for a 3-month trial period and soliciting feedback from library users with an anonymous form.

Program Conclusions:
The library’s previous discovery platform was customized for the health sciences, and Primo VE was configured to closely match the prior system. Since we transitioned to the shared consortium environment we can now include the broader scope of print content which is available locally and free of cost to users through the consortium loan service. This additional content can enhance search results for some disciplines, particularly public health. Qualitative testing demonstrated that there was not a detrimental impact to most of the typical searches run in the discovery service by including this content. We will continue to solicit feedback from users through the trial period and analyze borrowing metrics to determine if the broader scope remains the default platform setting.

Expanding Horizons: The Journey of Savitt Medical Library's Third Satellite Location in Reno, Nevada

Track(s): Clinical Support, Education, Information Services

Katie Jefferson, Interim Director, University of Nevada Reno

Alexander Lyubechansky, Head of Clinical and Instruction Services, University of Nevada Reno

Michelle Rachal, Medical Librarian, University of Nevada, Reno School of Medicine

Background:
The Savitt Medical Library, at the University of Nevada, Reno School of Medicine (UNR MED) has embarked on a groundbreaking initiative to open its third satellite library at Renown Regional Medical Center (Renown). Despite the significant role of hospital libraries in supporting clinicians, many have shuttered over time due to financial constraints and organizational changes. The Savitt Medical Library's partnership with Renown, the largest hospital in Nevada, began in 2017 with the creation of a branch library, staffed by library liaisons. The success of this collaboration led to the opening of a second satellite library at Moana Clinic in 2020. We are now in the process of establishing a third satellite library to open in early 2024.

Description:
This poster outlines the process of developing, implementing, and evaluating the satellite library service. The

Savitt librarians work closely with the hospital's Research and Education department, as well as other stakeholders, to ensure the tailored provision of services and resources. We also discuss the challenges faced by academic medical libraries within clinical settings. We share insights into this evolving landscape, emphasizing collaborative strategies to overcome challenges and ensure the continued availability of critical information services and resources in clinical settings.

Program Conclusions:
While the new branch's official launch is slated for early 2024, preliminary evaluations already indicate promising outcomes. Bringing library services to clinicians, nurses, and support staff at Renown Regional Medical Center not only addresses their informational needs but also fosters collaboration between institutions. Despite challenges, the initiative underscores the value of library services in enhancing healthcare delivery and uniting healthcare and academic communities.

Exploring Donor Relationship Management in Academic and Health Sciences Libraries: A Pilot Survey on Scale Purification

Track(s): Professionalism & Leadership

Alla Iansavitchene, Clinical Librarian, Corporate Academics, London Health Sciences Centre

Amanda Ross-White, Health Sciences Librarian, Queen's University

Background:
Like other nonprofits, academic and health sciences libraries rely increasingly on philanthropic support to fulfill their missions. However, unlike public libraries, limited research exists on how these libraries engage with donors effectively. Using an established conceptual framework for relationship measurement (Hon & Grunig, 1999; Jo et al., 2004; DeVellis, 2012), we seek to identify how the four pivotal dimensions crucial to donor relationship management — trust, control mutuality, commitment, and satisfaction — are applied within the unique context of libraries, and in particular health sciences libraries. (84 words)

Description:
Using the validated scales developed by Hon & Grunig, we will employ a meticulous judgmental approach to assess items for relevance, logic, and inclusiveness within the field of academic and health sciences libraries. Seeking input from library conference attendees in Canada and the United States, our methodology involves a pilot survey utilizing the scale purification method. In collaboration with experts and guided by Moore and Benbasat's (1991) three-stage framework, the research progresses through the iterative processes of item pool creation, instrument development, and rigorous instrument testing.

The goal is to craft a comprehensive and reliable tool for assessing donor relationships, laying the foundation for optimized donor engagement strategies in academic and health sciences libraries.

This research, encapsulated in the pilot survey, refined and enhanced through expert evaluation, sets the stage for a full-scale forthcoming study, offering strategic guidance to library professionals, administrators, and stakeholders on optimizing donor engagement strategies for sustainable support.

Collaborating with a diverse panel of experts, our research benefits from their valuable insights. In alignment with Elangovan and Sundaravel's (2021) methodology, our experts ensure question clarity, understanding, and response efficacy, enriching the instrument development process. (18

Program Conclusions:
This is the first step in a multi-year project to assess donor relationships in academic and health sciences libraries lays the foundation for a comprehensive and reliable tool geared towards assessing donor

relationships. Referencing established methodologies and contributing to the discourse on scale purification, our work advances the understanding of donor dynamics, aiming to optimize engagement strategies for sustained support in academic and health sciences libraries. Further research will encompass a full scale survey of library professionals and focus groups of individuals engaged in donor relations in academic and health sciences libraries. The insights gained will inform library professionals, administrators, and stakeholders, contributing to the enhancement of sustainable support strategies. (110 words)

Fostering Diversity Awareness in Nursing Students to Improve Health Disparities for Underserved Populations in Texas

Track(s): Education, Information Services

Jamie Quinn, Director of the Nursing Learning Resource Center, Baylor University Louise Herrington School of Nursing - Learning Resource Center

Background:
Baylor University, Louise Herrington School of Nursing (LHSON), is partnering with community shelters; The Family Place, and Austin St. Center, in delivering quality consumer health literature instruction to underserved individuals in the Dallas community. Additional partners include Rio Grande Valley Catholic Charities Respite Center and Texas River Ministry in delivering health education to meet the immediate needs of underserved minorities and in promoting healthy lifestyles for adults and children. To reduce health disparities and promote healthy behaviors, undergraduate pre-licensed nurses and select graduate nurses will be taught how to utilize MedlinePlus and DailyMed and promote these databases while delivering one-on-one nursing care with community members in underserved areas of Dallas and the Rio Grande Valley of Texas.

Description:
Project Goals:

1. Promote awareness of consumer health information by reaching minority community members in a person-centric dissemination model in geographically underserved areas of Texas.

2. Foster distinctiveness of NLM as a reliable, trustable source of health information.

3. Train and develop diverse pre-nursing and select graduate university students to explore extra-curricular activities that increase representation and participation in NLM teaching and outreach.

Goals will be met by training undergraduate and graduate nursing students about MedlinePlus and DailyMed databases and utilizing their assistance in providing resource awareness and training about MedlinePlus and DailyMed to community members residing at the Austin Street Center and the Family Place in Dallas. The website MedlinePlus.gov and DailyMed will be shown via mobile tablets at each location. Nursing students will also be trained to provide brochures and information about MedlinePlus to community members served during the LHSON mission trip to the Rio Grande Valley. The number of community members served will be kept along with the number of students who participate in this project. All level-four undergraduate students are required to take course NUR 4345 so all LHSON undergraduate students will be involved in this project at some point over a year.

Program Conclusions:
Program funding is provided by the Network of the National Library of Medicine, Region 3, Health Information Outreach Award which is in progress and concludes in April 2024. Utilizing Qualtrics surveys, program effectiveness will be evaluated on content delivered to NUR 4345 Population Health Nursing Students and will evaluate content delivered by undergraduate and graduate students to community

members in Dallas and the Rio Grande Valley of south Texas regarding accessibility and usefulness of NLM databases MedlinePlus and DailyMed. These educational opportunities positively impact Black and African American clientele served in the Dallas area and Hispanic clientele served in the Rio Grande Valley area. Expected outcomes will measure the user’s trustworthiness and perceived usefulness of the databases following database demonstrations.

From Conceptualizing to Creation: An Exploratory Look at Building a Health Literacy Toolkit

Track(s): Education

Amy Minix, Neuro-Health Sciences Librarian, Indiana University Bloomington

Sarah Vitelli, ILS Graduate Student, Indiana University Bloomington

Background:
The goal of the toolkit is to create a space for health science students from a variety of backgrounds (optometry, nursing, occupational therapy, etc.) to build health literacy skills. Students can move at their own pace by completing modules that cover a variety of concepts within and adjacent to health literacy. This information would be available through Canvas, the Learning Management Software used at the presenters’ university, and the difference between the current institutional information literacy toolkit is that it wouldn’t be attached to specific courses, but openly available online for students to access. The toolkit will supplement library instruction sessions, but will also address gaps in coverage where librarian support or expertise isn’t currently offered.

Description:
The presenter is in the initial steps of designing the project management flow for this project, but the general timeline for developing the health literacy toolkit involves the following steps:

Mining existing literature and theoretical frameworks to identify core competencies

Establishing the skeletal structure of the health literacy toolkit based on core competencies

Creating template for each core competency, which will be shared as modules (introduction, definitions, strategic implementation, an activity, and suggested help for further support)

Developing materials and consulting with content experts to ensure information is comprehensive and appropriate for a variety of learners

Pilot the toolkit with the Interprofessional Education Center and have students and faculty provide feedback

Adapt materials based on assessment data

Hard launch the health literacy toolkit and iterate as more feedback is shared or information becomes dated

The presenter is planning to apply for a health literacy outreach award, which will partially be used to pay participants for feedback during the soft launch of the toolkit.

Program Conclusions:
Outcomes of success would include qualitative and quantitative data. Quantitative data would include the number of unique users, total time spent on modules, number of completed modules, number of courses using the modules, and other data that informs use. Qualitative data would be captured during the soft

launch, such as user testing and interviews. Future assessments could be completed by aligning discipline-specific competencies against the health literacy toolkit to identify current coverage or gaps in the toolkit. Ongoing feedback from users would also be collected, though the exact format for gathering these responses has yet to be determined.

Generalist Repository Ecosystem Initiative (GREI) to Support NIH Data Sharing and Discovery

Track(s): Information Management, Innovation & Research Practice

Ana Van Gulick, Government and Funder Lead, Figshare

Julie Wood, COO, Vivli

Kristi Holmes, Associate Dean and Director, Northwestern University

Sara Gonzales, Northwestern University

Pearl Go, Data Librarian, Northwestern Feinberg School of Medicine

Background:
In 2023, the NIH implemented a new data management and sharing policy aiming to make research data publicly available and medical librarians have been essential in supporting researchers with related data sharing practices. In 2022, the NIH ODSS launched the Generalist Repository Ecosystem Initiative (GREI), which brings together seven generalist repositories (GRs) to enhance support for NIH data sharing and discovery. GREI recognizes that GRs play a key role in the NIH data sharing landscape to support the FAIR sharing of data in trusted repositories. GREI has a mission to “establish a common set of cohesive and consistent capabilities, services, metrics, and social infrastructure” and seeks to provide needed support to librarians assisting with data sharing in GRs.

Description:
A key component of GREI is “coopetition” (cooperation + competition), a term used to describe the collaboration among the GRs to advance repository functionality. GREI is focused on objectives that reduce the barriers to FAIR data sharing and discovery of data within and across repositories including consistent metadata, persistent identifiers, search functionality, and metrics of data (re)use. The GREI repositories have published a common metadata schema built on DataCite metadata fields along with recommended controlled vocabularies and jointly determined common metrics for reporting on data use and data citations in GRs. As these are implemented across GRs both researchers and librarians will benefit from simplified data deposit, clear GR best practices, higher quality metadata, and enhanced tracking of data impact.

GREI is also focused on training and community engagement and medical librarians are an important stakeholder. Through the creation of training and outreach materials including a GR comparison chart, GR selection flowchart, GR use case catalog, and documentation of GR data sharing best practices, GREI strives to support librarians involved in data sharing training and outreach at their institutions. Gathering community feedback on the GR needs of researchers and librarians is also essential to prioritizing the work of GREI.

Program Conclusions:
GREI has focused on providing training and resources for librarians who are supporting researchers to meet the NIH Data Sharing Policy. We are working to make it easier for librarians to provide this guidance and we will continue in future years to solicit feedback from the medical librarian community, who are providing vital support. Through repository enhancements, common functionality, and training and outreach,

GREI hopes to continue to support medical librarians as key actors in the data sharing landscape.

Global Index Medicus: A Global Database for a Global Perspective

Track(s): Health Equity & Global Health, Innovation & Research Practice

Teresa Jewell, Team Science and Research Services Librarian, University of Washington

Leslie Gascon, Collection & Research Services Librarian, University of Washington Health Sciences Library

Background:
Global Index Medicus (GIM) is a database of scholarly biomedical and public health literature produced by and within low-middle income countries. A significant proportion of GIM's material comes from non-English publications. While GIM provides access to a needed global perspective, researchers and librarians shared they find GIM difficult to use and so do not select it for their projects. GIM's rules are not as clear as those of other databases and include quirks not described in its official documentation. Our goal was to promote and simplify the use of GIM by thoroughly documenting its rules and syntax. This way, librarians and researchers can more easily create or translate searches for GIM, and include it in their projects more frequently.

Description:
Through experimentation and consultation with librarians, one of the presenters assembled a list of GIM’s idiosyncrasies. We developed a research guide on how to create and translate searches for GIM using the Springshare LibGuide platform. We will ask librarians and health sciences researchers to apply our guide to translate a search string from another database or create a new search string. Based on their feedback, we will develop the content and structure of the guide. To further the reach of this crowdsourced documentation and promote the use of GIM, we will announce the finalized guide on evidence synthesis listservs and present a poster at the MLA annual conference.

Program Conclusions:
We expect our testers to provide insight into the challenges and successes of using the GIM research guide to translate or create a search for GIM. We will also request feedback on whether the librarian or researcher is more likely to include GIM in their global health research as a result of the guide. If the testers encounter issues not accounted for in the guide, we will continue developing and revising the guide. Once the guide is serviceable, we will continue maintaining and updating it as needed.

Grounded in Truth: Crafting an AI platform to Respond to Consumer Health Questions Utilizing NLM Products

Track(s): Information Services, Innovation & Research Practice

Alex Henigman, Associate Fellow, National Library of Medicine | University of Missouri

Background:
Purpose: An experimental use case to explore the viability and potential benefits of Generative AI (GenAI) for use in customer response handling.

Background Description: To address challenges associated with GenAI, such as hallucination, an experimental AI chatbot was developed to tailor responses by leveraging three key NLM products: PubMed,

MedlinePlus, and ClinicalTrials.gov. The ultimate goal is to develop an AI platform capable of generating drafts for human review by the institution’s reference services team.

Setting: This specific use case is part of a larger 6-month GenAI pilot. The chatbot’s development took place within an instance within an NIH firewall on the Microsoft Azure OpenAI (AOAI) platform. A Fellow led the project under the supervision of the pilot sponsor along with an interdisciplinary team.

Description:
Developing: The project commenced by analyzing a sampling of consumer health questions submitted to a Help Desk. Drawing upon human responses and policies, the decision was made to focus the chatbot on three NLM products. The Fellow collaborated with computing and cloud representatives to explore the feasibility of creating a system capable of generating draft responses to consumer health queries. The Semantic Kernel with Bot Framework, utilizing Plugins containing NLM product APIs, was developed to ensure the chatbot grounded its responses in the NLM products.

Implementing: Subsequently, the chatbot was deployed and rigorously tested. It demonstrated proficiency in understanding submitted queries, determining the optimal NLM product for information retrieval, performing API calls, and delivering responses grounded in NLM resources. The next phase involves internal and external evaluations of the generated responses.

Evaluating: Evaluation will be based on bias, safety, and the chatbot's responses to 10 de-identified questions from the Help Desk. Key themes for assessment include Instances of Medical Advice, Inclusion of NLM Resources/Gold-Standard Links, Tone, Response Accuracy, Question Relevance, and Safety Considerations. An external survey conducted by information professionals to gauge the chatbot's success.

Program Conclusions:
The exploration of restricting AI systems to trusted resources, such as NLM products, offers a method to ensure generated responses are grounded in accuracy and supported by verifiable references. Survey results will be compiled and presented at MLA '24.

Healthy People 2030: Developing Structured Evidence Queries for Discovery and Education

Track(s): Health Equity & Global Health, Information Services

Samantha Guss, Research and Education Librarian, Virginia Commonwealth University

Anita Kuiken, Sport/Human Dynamics Librarian, Syracuse University

Erin Wentz, Assistant Professor and Electronic Resources Librarian, Massachusetts College of Pharmacy and Health Sciences

Courtney Pyche, Public Health Liaison Librarian, University of Florida

Dani LaPreze, Assistant Professor, University of Louisville, Kornhauser Health Sciences Library

Erin Reardon, Public Health Informationist, Emory University

John Mokonyama MS, MBA, MSLS, AHIP-, Medical Librarian, Penn Medicine, Chester County Hospital

Laura Zeigen, Health Sci Ed/Research Lib, OHSU

Background:
Healthy People 2030 is an initiative by the United States Department of Health and Human Services (HHS)

that started with Healthy People 1990 to improve the health of Americans. Health sciences librarians have previously authored structured evidence queries (SEQs) in PubMed in 2010 and 2020 to encourage and make accessible research that relates to the Healthy People objectives within a formal partnership with HHS. This group of librarians endeavored to create structured evidence queries in PubMed for Healthy People 2030 objectives to continue to facilitate accessibility of research and monitoring of the progress towards the objectives and to document the process for future iterations.

Description:
This program is designed to be a publicly accessible resource. Historically, the structured evidence queries (SEQs) have been shared on the United States Department of Health and Human Services (HHS) site. The group determined, based on the size of the group and the number of objectives, to focus on designing searches for the leading health indicator (LHI) objectives. Spreadsheets were created and helpful resources were identified to organize and to inform the SEQS. Group members volunteered for each LHI and to peer review other searches. Regular working meetings were held to ask questions and collaborate. Simultaneously, the group created a survey and disseminated to relevant caucuses to collect stories of use-cases of previous SEQs to assist in promoting the searches. The group is in the authoring and peer review stage and hopes to begin validating searches soon. In order to encourage use, the group identified ways to promote the SEQs. As there was no formal partnership with HHS in this iteration, they determined that a publicly accessible Open Science Framework Project would be appropriate to share the SEQs. They have also identified stakeholders within academia, government, and non-profit organizations that may be interested in using the results.

Program Conclusions:
The creation of the structured evidence queries is ongoing. After validation, the group plans to promote the Open Science Framework (OSF) page through conferences, webinars, listservs, and direct email. Usage will be evaluated quantitatively and qualitatively through tracking citations of the OSF page, submissions to the survey on use-cases, and through analytics available on OSF. The librarians also plan to create a form that will allow the public to request structured evidence queries for other objectives.

Hello! We're Here!: Marketing your Medical Library

Track(s): Clinical Support, Information Services

Jennifer McKay, Associate Professor and Head, AK Medical Library, UAA/APU Consortium Library, University of Alaska Anchorage

Background:
When you are the sole medical library in your state and you serve healthcare professionals who work in rural clinics and hospitals off the road system, you periodically attempt to promote the library's services to its active and inactive clients as well as brainstorm ways to attempt to attract new clients. The head of this medical library has attempted a variety of methods in marketing medical library services but this work is never done.

Description:
This poster will showcase visually the methods that have resulted in the best return on investment for its population of users, many of whom work in geographically isolated rural clinics. The marketing strategies represented in this poster will be of benefit to those who serve rural clinics/hospitals in a time when misinformation runs rampant and medical libraries have to continually justify its services.

Program Conclusions:
The success of these marketing strategies saw a significant increase in the medical libraries services: article retrieval, literature reviews and client interactions. These will be shared in a graph showing before marketing

and after and how usage has increased.

Highlighting Health and Diversity: Curating Digital Exhibits to Promote Online Resources Across Cultural Months

Track(s): Health Equity & Global Health, Information Services

David Carson, Health Sciences Librarian, Chapman University

Ivan Portillo, Directory of Rinker Campus Library Services, Chapman University

Background:
Libraries have increased their efforts to support and promote diversity, equity, and inclusion at their institutions. One method to accomplish this goal is by providing culturally diverse resources and services to support teaching and learning. Medical and health science libraries may find that culturally diverse health resources are not common in existing resources and materials. Most materials focused on culturally competent healthcare are delivered via handbooks or book chapters, which may be difficult to discover. Historical events and individuals who helped improve the quality of care for diverse populations may also be lost in these resources. To highlight the available resources and historical hallmarks, health science librarians created digital exhibits to promote these available tools across nationally recognized cultural months.

Description:
The idea to curate and develop digital exhibits came as an alternative to the physical exhibits being curated at the main library at our institution. While our health science library did not carry a physical collection covering competent healthcare for diverse populations, there were relevant titles in our ebook collections on caring for diverse populations. To further enhance the digital exhibit, we highlighted individuals and historical events that have led to great achievements or developments in providing quality care to each population being highlighted across cultural months. Rather than creating a LibGuide or webpage to display the resources, we used Scalar to create an interactive and visually rich exhibit. Scalar is an open-access platform designed for universities to publish long-form scholarly content. We currently have digital exhibits for AAPI Heritage Month, Native American Heritage Month, Hispanic Heritage Month, Black History Month, LGBTQIA+ Pride Month, and Women’s History Month. Each exhibit is marketed through digital signage, library newsletters, and listservs directed at Health Science students, faculty, and staff.

Program Conclusions:
Promoting these resources has led to engagement with faculty and the use of the resources in the health science program curriculum. Additional resources beyond ebooks and historical summaries have been included in each exhibit, including topical journals, interactive timelines, practice guidelines, data and statistics, and links to local and national organizations advocating for improved care for underserved populations.

Hospital Workplace Violence Prevention Programs: How Librarians Can Contribute

Track(s): Clinical Support, Education

Fred King, Medical Librarian, MedStar Washington Hospital Center

Mark Marino, Director, Workplace Violence Prevention Program, MedStar Health

Background:
Workplace violence is a serious problem in healthcare settings; healthcare workers are five times more likely to experience nonfatal workplace violence than workers in any other profession. At MedStar Health, a not-for-profit healthcare organization with ten hospitals, 33 urgent care clinics, ambulatory care clinics, and 300 other locations in the Baltimore-Washington region, medical librarians play a significant role in the system’s workplace violence prevention program and on the local and system-wide multidisciplinary workplace violence prevention committees.

Description:
The dearth of evidence-based interventions in healthcare workplace violence prevention presents significant opportunities for medical librarians to facilitate data collection, tool creation, and program development. During program development, medical librarians provided regulatory literature from government agencies overseeing workplace safety, performed targeted literature searches on topics and programs of interest, and provided evidence-based tools for consideration and implementation. The foundational literature and tools that the librarians found served to provide structure for policy protocol implementation and the tools found are being incorporated into the electronic medical record. Now that the Workplace Violence Prevention program is underway, librarians continue to locate and disseminate literature and articles for review both at local and system workplace violence prevention committees; for example, medical librarian presents and discusses a relevant article at the monthly local committee meetings. As the program gathers more data about reducing workplace violence, medical librarians will play a role in the publication of the program’s work and findings.

Program Conclusions:
It is highly unlikely that the work of this committee will come to an end; the incidence of workplace violence can be diminished as healthcare professionals share and discuss data and ideas, but, like the work of infection control, workplace violence prevention programs will always be needed. Medical librarians will continue to serve as the “eyes and ears” in identifying trends and research in healthcare violence prevention.

Impact of the ECHO Librarian Role on Project ECHO's Tele-mentoring Model

Track(s): Innovation & Research Practice

Bonnie Leigh Reifsteck, Project ECHO, University of New Mexico

Kent Norsworthy, Library Services Manager, University of New Mexico

Background:
Project ECHO was launched in 2003 by Dr. Sanjeev Arora, aiming to empower rural health care providers with expert knowledge and best practices through videoconferencing and virtual communities of practice. ECHO has proven effective across disciplines and geographies as a way to reduce disparities, strengthen health systems, and drive collaborative solutions. There are now over 1,200 ECHO hubs running more than 6,200 ECHO programs in 63 countries, reaching participants in 199 countries. We introduced the ECHO Librarian role to our programs in 2020 to enhance the value of each session for ECHO participants and hub team members, and to reinforce ECHO’s brand as a trusted source of evidence-based information.

Description:
The ECHO Digital Librarian role (aka, “embedded librarian”) was first developed by our partners at ECHO Ontario in 2015. We adapted the role in New Mexico as part of our COVID-response ECHO programs. The

Librarian serves as a member of the interprofessional hub team, providing links to references and resources in the Zoom chat during the session. Most ECHO programs run weekly for one hour, and include a didactic presentation and anonymized clinical case presentation. The Librarian reviews presentation slides prior to the session, verifies and formats the references for posting in the Zoom chat, and then searches in real-time for references mentioned by subject-matter experts during discussion. With an overwhelmingly positive response from participants and experts, we expanded the role to begin serving more traditional, long-standing ECHO programs. With introduction of the iECHO platform, we integrated a standardized Resource Library for programs compiling all session references. We track the programs, sessions, and number of participants served; the number of references posted; and the participant feedback on regular post-session surveys. This information about the ECHO Librarian role is published to an internal dashboard that demonstrates the impact of our service.

Program Conclusions:
Since piloting the role in Spring 2020 through the end of 2023, our ECHO Librarians have provided support to 42 unique ECHO programs, serving over 125,000 participants on over 1,000 sessions and posting over 10,000 references. We currently support 20 ongoing ECHO programs, covering topics such as Adverse Childhood Experiences, Behavioral Health, Climate Change and Human Health, Clinical Communication, COVID-19, Endocrinology, Gender Affirming Care, HIV, Infectious Diseases, Palliative Care, Post-COVID, Reproductive Health, Violence Prevention, and Viral Hepatitis. The role has been featured in several peer-reviewed journal articles. We have found that the role is most effective with ECHO programs that are anchored by a strong community of practice and that include research-rich didactics.

Improving Wayfinding and Policy Explanation via a Signage Audit

Track(s): Information Services, Professionalism & Leadership

Hannah Norton, Chair and Fackler Director, University of Florida

Matthew Daley, Web Designer, University of Florida, George A. Smathers Libraries

Background:
Like many other libraries, the Health Science Center Library at [institution] contains a multitude of physical signs across its footprint, using a variety of styles and posted at various points in time, borne out of a desire among staff to communicate various messages to library users at their point of need. Given the fresh perspective of a new library director and past user experience training, a 5-member team of library employees decided to undertake a signage audit, reviewing signs across the library for potential improvements.

Description:
The team, convened by the chair of the library, also included the associate chair, daytime and evening circulation supervisors, and an IT specialist with expertise in graphic design. Through consensus, the team agreed that goals of the signage audit would be to improve the clarity and overall consistency of signage within the library. After a planning meeting, the team walked through the library together, photographing every unique sign, documenting its location (on printed floorplans), and making notes. Where there was immediate consensus that a sign was no longer needed, it was removed during the walk through, but the bulk of the decision-making occurred during follow-up meetings. At follow-up meetings, each sign was reviewed and tagged to be left, removed, redesigned, reprinted, or digitized. More specific actions were also noted in a spreadsheet, with the responsible party identified where possible. The team reviewed a total of 68 signs, with the vast majority needing some sort of intervention (13 to be removed, 27 redesigned, 3 moved, 3 digitized).

Program Conclusions:
Performing the signage audit inspired a larger conversation about sign templates, including use of common logos and headers, desired document formats for editing, and shared digital storage locations that would be usable for all relevant employees. A subsequent project by the library system’s director of marketing and communications raised similar issues: avoiding loose paper signs, using the correct logos, and checking regularly on temporary signs to ensure they are still needed (or are taken down). Additional follow-up at the health science center library will occur this spring to ensure that all sign actions from the audit have been completed and a clear process is in place for making changes to signage in the future.

Inclusivity Benefits Everyone: A Guide for Health Professionals Working with Transgender Populations

Track(s): Health Equity & Global Health, Information Services, Professionalism & Leadership

Jo Dienst, Health Sciences/STEM Librarian, Duquesne University

Reagan Harper, Research and Instructional Design Librarian, Duquesne University

Background:
This online research guide was developed to facilitate a contextualized understanding of the medical needs and desires of transgender and gender-nonconforming communities. According to a report from the 2015 US Transgender Survey, 24% of transgender people had to educate their healthcare providers themselves on the needs of transgender people to receive proper treatment. Legislative concerns around gender are also on the rise, with the ACLU citing 137 bills currently proposed in the United States for gender related healthcare restrictions. Healthcare professionals must be sensitive to the unique needs of transgender patients to provide inclusive and equitable care for all. As information professionals, this guide aims to direct students, staff, faculty, providers, and patients to relevant information and advocacy resources.

Description:
The guide was primarily created using Springshare’s LibGuides platform, though other platforms were used as well. An embedded interactive widget defining the LGBTQIA+ acronym was created using the app Genially, while a historical timeline was created using Knight Lab’s TimelineJS software. This guide was also created following WCAG standards - Level AA.

Background information on the guide includes introductory vocabulary terms relevant to transgender populations, a brief history of gender identity in the United States and western Europe, and ongoing legislation around gender in the United States. The guide also outlines healthcare implications, including definitions of the various levels of gender-affirming care, how those treatments and interactions impact patients, and the risks that affect transgender and gender-nonconforming individuals. Advocacy organizations and resources are also highlighted in order to inspire users to encourage policy reform and access to effective, affirming care.

Additionally, library resources and collections were noted when appropriate to direct users to materials that are available to them. The author then reached out to librarians, on-campus DEI committees, and practitioners with relevant expertise from a variety of disciplines, including nursing, pharmacy, occupational therapy, public health, and education, to gauge and incorporate feedback.

Program Conclusions:
To determine evidence of this guide’s usage, page views were culled since its publishing in June of 2023. Since its publication, the guide has received 381 page views. The authors also plan to advertise the guide through course integration in Canvas for classes that specifically cover this topic.

Incorporating Avoiding Plagiarism into a Drug Information Course

Track(s): Education

Roslyn Kohlbrecher, Pharmacy Librarian, University of Connecticut

Diana Sobieraj, Associate Professor, University of Connecticut

Background:
Maintaining academic integrity is a priority for professional pharmacy programs, but fitting this topic into the curriculum can be a challenge. In the absence of a medical writing course, our institution uses a drug information course to introduce students to the topic of avoiding plagiarism. The professor of this course approached their librarian for ideas on improving the way this topic had historically been presented. The librarian used an existing resource to build a LibWizard tutorial on avoiding plagiarism that is tailored to the PharmD curriculum.

Description:
The tutorial includes 24 slides that present information and are accompanied by knowledge check questions. The tutorial is completed asynchronously by the students and then discussed in a synchronous class session. Concepts covered include but are not limited to: defining plagiarism, unintentional plagiarism, incorporating common knowledge and opinions, referring to sources in written work, using images, fair use, detecting plagiarism, and consequences of plagiarism. We also point out that common knowledge within the pharmacy field is different versus that of the general population. After the appearance of various AI chatbots in recent years, we felt it was important to add a slide on guidelines for using these tools in an academic setting. The tutorial has been used by three PharmD classes since Fall 2021 (286 submissions). It also has come to serve as a source that any student or professor in the school can use as a refresher when plagiarism issues arise.

Program Conclusions:
We will present selected slides from the tutorial and discuss general trends regarding topics that the students understood well and what they appeared to struggle with, including what constitutes plagiarism and incorporating sources via quotation, paraphrase, and summary.

Incorporating Information Literacy into Area Health Education Center (AHEC) Curriculum

Track(s): Education, Health Equity & Global Health

Jason Francis, Health Science Librarian, Weber State University

Diana Meiser, Engineering, Applied Science & Technology Librarian, Weber State University

Frederick Henderson, Director, NUAHEC, Weber State University

Shaun Adamson, Professor, Education Librarian, Weber State University

Background:
To address healthcare shortages in the U.S., National Area Heath Education Centers (AHEC) were developed by the U.S. Department of Health and Human Services, Health Resources and Services Administration in 1972. In order to recruit students, AHEC partnered with our university to develop a curriculum for high school students considering careers in healthcare. From its inception, administrators recognized the need to incorporate health literacy in the program. To date, 2310 college credits have been

awarded to 613 participants in three school districts through a course taught by university faculty. This course addresses current healthcare issues, offers various certifications, explores healthcare careers with job shadowing opportunities, and provides direct interaction with area doctors.

Description:
The Health Sciences Librarian and AHEC administrators developed a course that embeds information literacy (IL) skills within the health science course. Intended for students considering careers in healthcare, this course investigates various healthcare careers and focuses on how social determinants, cultural competency, interprofessional education, and behavioral health can impact population health and the healthcare system. Instruction and practice focus on conducting research and applying health sciences literature in the context of evidence-based practice. Course outcomes integrate Association of College & Research Libraries’ (ACRL) Framework For Information Literacy for Higher Education (2016) and IL Competency Standards for Nursing (2013): articulating the value of information literacy to the health professions; understanding healthcare information presented in formats of varying complexity; understanding the importance of legal/ethical standards to the dissemination and retention of healthcare information; being able to conduct evidenced-based research on healthcare topics; synthesizing information and conducting presentations using statistical data; and setting academic goals and refining career aspirations through skills specific to healthcare careers and research. Goals of this program include matriculating students into healthcare programs with health literacy skills.

Program Conclusions:
Currently, matriculation into health science programs is higher than all other colleges/depts at our university, supporting AHEC goals to recruit students into healthcare. Matriculation rates for our course are at 49% (69% BIPOC students) compared to the university’s concurrent enrollment matrication rate of 23%. Health literacy pre/post tests show dramatic learning gains (learning percent increase of 118% from pre- to post-test for Fall 2023 courses), and pre/post data is currently being analyzed at the outcome level for all courses. This provides students with a solid foundation upon being accepted into a program. The final project is a live group presentation demonstrating mastery of all IL outcomes, ensuring original work rather than AI generated content; performance averages 93.4% for all classes.

Increasing User Engagement by Decreasing Clicks: A Collaboration Between Graduate Medical Education and Librarians

Track(s): Clinical Support

Emma O'Hagan, Clinical Services Librarian, University of Alabama at Birmingham

Lindsey Baird, Clinical Reference Librarian, University of Alabama at Birmingham

Background:
The information needs of graduate medical education learners are unique from traditional students. During this phase of medical education, the priority is gaining clinical experience and competence while becoming a subject expert. Residency programs may direct learners to library resources via various platforms outside of library websites. As clinical librarians at a large academic medical center, we find that faculty and residents curate electronic resources independently of librarians to ensure easy access. On the other hand, librarians create resource guides as a method to share electronic resources, often based on the librarians' best guess of user preferences. Our goal is to improve our clinical resource guides through partnering with stakeholders in clinical departments to identify high-value resources.

Description:

There are 33 residencies and 84 fellowships at our university medical center. To create meaningful resource guides that will be used by learners and clinical faculty we must identify the highest value resources, integrating feedback from stakeholders like Program Directors, Program Managers, Education Coordinators, and Chief Residents. In the past, this process has been slow, sometimes taking weeks or months of emailing back and forth to gain end-user input. To streamline this process, we proposed a new strategy, utilizing collection development tools to generate high-value content lists which will be used to create department-specific surveys. Our first step was to create a list of education administrators and resident stakeholders for each residency program. Next, we will generate lists of essential resources for each program using Doody’s Core Titles lists, Journal Citation Reports, and library usage statistics. Finally, this content will be translated into surveys using LizWizard and circulated among 5 to 10 stakeholders per clinical program. Surveys will also seek input about user preferences related to content not already included in the survey. The results of these surveys will be used to generate new resource guides quickly and efficiently.

Program Conclusions:
The first round of surveys will circulate in March of 2024 and a selection of the new resource guides will be created using the content identified through the surveys by the end of April. Outcomes measured will include usage data for new guides, time required to develop and publish resource guides, and number of guides created. The first new guides will be essential in creating a template for future clinical resource guides. Librarian experiences with the new process and workflow will also be shared. After completing this process, we will use what we have learned to create a further streamlined process to create resource guides for selected fellowship programs.

Integration in a Residency Scholarship Curriculum: Successes and Challenges in a Family Medicine Residency Program

Track(s): Education, Information Services

Julia Stumpff, Instructional Design Librarian, Ruth Lilly Medical Library, Indiana University School of Medicine

Cecelia Vetter, Medical Education and Student Engagement Librarian, Ruth Lilly Medical Library, Indiana University School of Medicine

Emilee Delbridge, Assistant Professor of Clinical Family Medicine, Behavioral Science Faculty, Department of Family Medicine, Indiana University School of Medicine

Background:
The Accreditation Council for Graduate Medicine Education (ACGME) requires Family Medicine (FM) residents to complete two scholarly projects during residency. In early 2020, a [medical school] FM faculty member reached out to request library instruction for second-year residents (PGY2) working on scholarly projects. Tailored library sessions were integrated into the second-year residency curriculum. Two years after implementing this program, liaison librarians and the faculty member surveyed 23 residents to understand what residents learned and what training they needed in the future to assist them with their scholarly projects. Librarians and the FM faculty member then added citation management content to the curriculum. A research study to measure the impact of the library interventions on resident’s library research skills was pilote

Description:
In Fall 2020, a librarian began providing an interactive presentation and handout to equip second-year Family Medicine residents with skills for effective literature searching. The librarian also familiarized residents to library resources and helped them explore materials relevant to their individual scholarly

projects. At the end of the second year, the 23 residents (both 2nd year and 3rd year residents) who received the training were surveyed. Based on the feedback received, the librarians and FM faculty member decided to provide additional instruction on citation management tools. They also piloted a pre/post-test research project with the 3rd cohort of students using a modified version of the Research Readiness Focused Assessment (RRFA4). Before each session, the residents were asked to complete a pre-test. Later in the year, after being provided a tailored video on how to use EndNote, residents were asked to complete a post-test. Pre-test data showed that the FM residents lacked knowledge in locating library resources and were not comfortable with citation management tools. Post-test data showed that most residents were clearer about where to search for sources for their research, were more comfortable using citation managers, and were able to identify more precise and suitable resources for scholarly research.

Program Conclusions:
The pre and post-test data provided helpful feedback to the librarians, but the small number of residents in the FM program and scheduling conflicts limiting instruction time complicated the librarian’s ability to get statistically significant results. The FM residency program cohorts are limited to thirteen residents each year, and due to clinical rotation schedules, multiple introductory library sessions are needed to reach all residents. Creating a recorded video for the EndNote library sessions alleviated scheduling challenges, but collecting data proved to be a challenge, as residents needed several promptings to fill out the post-test evaluation. This academic year, a librarian began meeting with residents in person to teach EndNote and collect post-test data.

It Takes a Village: How New Connections Influenced the Development of a Graphic Medicine Collection

Track(s): Information Management

Janet Chan, Research and Education Librarian, University of South Florida

Krystal Bullers, Research & Education Librarian, USF Health Libraries, University of South Florida

Melinda Berg, Cataloging and Metadata Librarian, University of South Florida

Tsu Yin Lue, Research & Education Staff Assistant, USF

Background:
This graphic medicine (graphic novels with a health or medical focus) collection development project started with the interest of one librarian, from the research and education unit, in graphic medicine as an innovative, engaging, and creative format in which to enrich the student learning experience. This interest led to a cross-departmental collaboration with a librarian from the collection management unit. Although the end objective was the establishment of a graphic medicine collection, the process of researching and garnering support for the collection also resulted in multiple connections with interested parties within the library, the university, and other universities.

Description:
This poster will describe the steps taken to research and implement the graphic medicine collection and the partnerships and connections that helped to support the project along the way. This project began with the interdepartmental collaboration of librarians conducting research on graphic medicine collections in academic health science libraries. The research provided initial data on the prevalence of graphic medicine collections and confirmed that the format has become extremely popular with library patrons. This inspired the librarians to seek to add a collection to their library.

A regional medical librarian conference provided an opportunity to meet fellow librarians presenting on graphic medicine. These connections fostered networking opportunities and director-level interest in

acquiring the collection. To identify use cases and potential users of the collection, we made connections with the medical humanities faculty at our university. The information gathered throughout this process was then compiled into a complete Graphic Medicine Collection Project Proposal that included the benefits of graphic novels in health education, a title list, implementation plan, costs, marketing and outreach ideas, and evaluation metrics.

Program Conclusions:
Our library director approved the project and acquisition of the titles began in January 2024. The connections made throughout the process of adding a graphic medicine collection to our library were instrumental in gaining support for the project and providing the data needed to move forward. As part of our project plan, we established the need to continuously assess the collection's impact on learning, user engagement, and interdisciplinary collaboration by tracking circulation statistics, in-library use, social media engagement, and library class and/or event attendance. Specific project elements and initial data will be presented.

Joining Kindred Spirits to Develop an End-of-Life Resource Guide

Track(s): Clinical Support, Education, Health Equity & Global Health, Information Services

Martha Earl, Director and Professor, UT Graduate School of Medicine

Michael Lindsay, Head of Collections & Access Services, University of Tennessee

Background:
Dealing with end-of-life issues for a loved one is difficult for survivors in many ways. Advanced care planning, wills, palliative care and hospice care are among the issues adding distress for families. Resources on these topics are in disparate places; navigating these issues are challenging even for those familiar with the healthcare system. Following the passing of a family member of a librarian, librarians at Preston Medical Library realized the need to assemble a resource to ease this process. Patients, family members, faculty, residents, and staff served by the University of Tennessee Medical Center (UTMC), a 685-bed teaching hospital.

Description:
Librarians consulted with palliative care nurses, who identified needs for information for advanced directives, critical care decision making, and support and respect for patient wishes. In collaboration, the library prepared a checklist of crucial steps and documents needed for patients and their advocates in making their final preparations. The team added input from hospital pastoral care for resources on bereavement and grief and counseling at the end of life. Palliative care nurses began sharing the completed guide with patients.

Program Conclusions:
Following the guide’s release, palliative care nurses shared the guide with patients. In November of 2022, the guide had 313 uses, making it the single most used library subject guide for that month. Refinements are ongoing, with plans to improve the checklist of information and documents.

Keys to Success: The Evolution of a Mandatory Medical Informatics Longitudinal Course in an Undergraduate Medical Curriculum

Track(s): Education

Elizabeth Irish, Associate Professor, Schaffer Library of Health Sciences, Albany Medical College

Enid Geyer, Associate Dean for Information Resources and Technology; Associate Professor, Schaffer Library of Health Sciences, Albany Medical College

Traci Tosh, Assistant Professor, Schaffer Library of Health Sciences, Albany Medical College

Background:
The successful evolution of a four-year mandatory pass/fail longitudinal medical informatics course in an undergraduate medical (UME) curriculum at a private medical college is explored. The initial goal of the course was to teach information retrieval and management skills to support medical students in becoming self-directed, lifelong learners, but that goal has broadened to incorporate all aspects of evidence-based practice, including, clinical epidemiology, and critical appraisal. In response to this change, library faculty (librarians) roles as part of the education team have also evolved.

Description:
The concept of the four-year mandatory pass/fail longitudinal medical informatics course in the undergraduate medical curriculum (UME) was initially introduced in 1993. The course began with one basic search session delivered in UME 1. Over time, the librarians participated as peer members of institutional educational committees to identify needs, potential collaborations, and professional development opportunities. Course success depended upon the librarians’ ability to adopt new medical education teaching methodologies, technologies, and theories while simultaneously supporting other faculty in doing the same. Another key aspect to the librarian’s success is the relationship formed with the medical education leadership team. During the recent curriculum renewal, a librarian was charged with leading the team responsible for combining the existing medical informatics and evidence-based healthcare courses into one. The new course taught its first cohort in the 2022-23 academic year. The key to longevity and growth is to never remain stagnant by incorporating continuous course reviews, following medical education trends, and staying open to feedback.

Program Conclusions:
In this section we will recap the keys to success and discuss the college’s Curriculum Assessment Team (CAT) observations of the initial curriculum that contributed to the development of the new course. Selected faculty and student feedback will be highlighted. A chart illustrating the growth of the program will be included.

Leading by Example: Strategies for Authentic Leadership

Track(s): Professionalism & Leadership

Rich McIntyre, Emerging Technologies Librarian, UConn Health

Teri Shiel, Head of User Services, UConn

Background:
Objectives: This poster will provide a framework for librarians to understand Authentic leadership principles; to gain practical insights; and to explore real-world examples. Background: Authentic leadership in medical librarianship emphasizes genuine practices aligned with individual values, fostering purpose, trust, clarity, and self-discipline which contributes to a positive work environment and overall library success.

Description:
This poster integrates theoretical insights from the Harvard Leadership Institute and Library Juice Academy with practical experiences, providing a focused exploration of authentic leadership principles in the context

of medical libraries. We developed this poster with a focus on purpose-driven leadership, relationship-building, clarity of communication, and self-discipline, ensuring relevance to the unique challenges faced by medical libraries.

Program Conclusions:
Participants will gain an understanding of authentic leadership and learn from our practical insights and real-world examples, inspiring them to foster positive work environments, encourage future leaders, and advance medical libraries through effective leadership.

Let's Discuss: The History of Medical Libraries and Medical Librarianship

Track(s): Professionalism & Leadership

Margot Malachowski, Education and Outreach Coordinator, Network of the National Library of Medicine

Background:
The Network of the National Library of Medicine Book Discussion offers library staff an opportunity to explore topics with other professionals and earn Medical Library Association Continuing Education Credit. A new book is selected each quarter. Participants have three months to complete the requirements. The objectives are to increase library staff awareness and understanding of health information access issues, and to explore health resources from the [organizations redacted during submission] and other organizations related to the topic. From February 1-April 30, 2024, book discussion participants explored the history of medical libraries and medical librarianship. In online forums and Zoom meetings, participants examined the evolution of the profession and planned for next steps in their own career.

Description:
The Network of the National Library of Medicine (NNLM) Book Discussion series is hosted in Moodle, an open-sourced learning management system. Participants log in at their convenience and respond to discussion prompts in an online forum. The History of Medical Libraries and Medical Librarianship forum was open for three months, from February 1-April 30, 2024. To offer options for live discussion, NNLM hosted Zoom meetings in February, March, and April. In the first month, we discussed the establishment of medical libraries 1836-1898, the establishment of medical librarianship 1898-1945, and the development of the clinical research infrastructure 1945-1962. The discussion shifted in March to the development of the National Library of Medicine 1962-1975 and the creation of the health sciences library infrastructure 1975-1995. In April, we concluded with the transition from paper to digital information 1995-2015 and the development of the digital health sciences library 2015-current day. At each juncture, participants considered how history could inform their own decision-making for their libraries and their careers. Participants were encouraged to share actionable steps in how they could improve library services. Sidebar online forums were available for conversations that veered away from the stated purpose of the book discussion.

Program Conclusions:
At submission, there are 71 registrants for the Book Discussion. The poster will a timeline of the history of medical libraries and medical librarianship, a visual of the number of active participants in relation to the registrants, and word balloons of ideas generated during the three-month discussion.

“Laboring with Hope” to Improve Maternal Health Disparities

Track(s): Information Services, Professionalism & Leadership

Carolyn Martin, Outreach and Education Coordinator, Network of the National Library of Medicine, Region 5

Emily Hamstra, Assistant Director, Network of the National Library of Medicine, Region 5

Background:
According to a recently published JAMA article (Fleszar et al, 2023), maternal deaths have more than doubled in the last 20 years with the highest rates among Native American, Native Alaskan, and Black women. Over the past year, the Network of the National Library of Medicine (NNLM) has hosted multiple programs to improve awareness of maternal health disparities and to create opportunities to share health information. This poster will highlight one program created by NNLM Region 5. Through this program, NNLM Region 5 worked with a public health organization to provide an opportunity for libraries and organizations to share information and learn about maternal health disparities through watching and discussing a documentary film.

Description:
In August 2022, the PNC-MLA Diversity and Health Equity Speaker series hosted Dr. Nakeitra Burse, to present how storytelling, through the documentary film Laboring with Hope, is being used to create programs and services to address maternal health disparities experienced by Black women. Dr. Burse is the Executive Producer of the film Laboring with Hope and is the owner/CEO of Six Dimensions, a certified woman owned, minority owned public health research, development and practice company.

Laboring with Hope is a 30-minute documentary that combines personal stories and expert perspectives to provide a call to action to implement, support, and uplift strategies to improve maternal health and birth outcomes.

Region 5 staff reviewed the feedback, comments, and the engagement of attendees and saw an opportunity to connect the film to its regional members.

Region 5 worked with Dr. Burse to offer members free, unlimited, streaming access to the film to share information about these health disparities and provide reliable health information. Members who host a screening of the film are given a conversation guide, bibliography of peer reviewed articles on the topic, a handout listing information resources on maternal health, and infographics related to access to maternal healthcare.

Program Conclusions:
Since January 2023, we have had 8 member organizations participate in the program. Events have included:

- Incorporating the film into a Nursing course
- Passive programming noon time viewing of the film in the library for medical students, faculty, and staff
- Film viewing and programming during Black Maternal Health Week
- A library collaboration with a campus Women’s Resource Center
- A library collaboration with Health Sciences, Nursing, and Public Health departments to host a viewing and discussion with students

Region 5 members were encouraged to submit a short report regarding their film viewing program. Feedback from attendees of the film and related programming were very positive in highlighting the health disparities among Black women and women of color and inspired many to consider next steps.

Librarian Support for Graduate Medical Education: 5 Year Check-In

Track(s): Education, Information Services

Abby Adamczyk

Background:
In 2019, Graduate Medical Education (GME) Administration and the Library recognized the need for a librarian to support the scholarly objectives of the institution's residency and fellowship programs. Part of the reference/education department at an academic medical library, the librarian would provide research consultation and education to over 1000 residents and fellows in more than 100 programs . Initial program objectives were: expanding outreach to more programs, increasing education sessions, improving program scholarship.

Description:
A GME Librarian was hired in 2019. Initial outreach was boosted by a GME Program Directors Retreat. To facilitate introductions and collaboration with faculty, the librarian developed handouts about resources and services. This and other outreach lead to an increase in education requests. Previously, the library had an ongoing seminar series with one fellowship program. Since 2019, we have presented 48 synchronous seminar sessions for 14 GME programs. These efforts also increased awareness of other library services. From 2014 to 2019, the library averaged 18 resident consultations per year. Since 2019, the library has averaged 49 resident and fellow consultations per year. To reach busy residents, 4 asynchronous online modules were created on topics regularly covered during seminars and consultations. Despite expressed interest in online, asynchronous content, the modules have not seen as much use as predicted. In-person seminars are still the preferred format.

In year 3 we began developing a pilot survey to discover gaps in our outreach and education efforts. An initial survey was distributed in April 2023 with limited response. This data was used to determine wich programs may need more attention and support. The survey data is not reported here due to lack of IRB approval.

Program Conclusions:
In the first 5 years, this program has been successful in increasing library engagement with GME programs through consultations, seminars and training on topics include: general library orientation, library tools for evidence-based medicine, advanced literature searching skills, and citation management. Goals for the future of this position include: 1) repeating the survey with IRB approval and 2) targeted outreach to those programs identified in the initial survey as having gaps in support and less knowledge of the library and 3) exploration of new topics areas such as scholarly communications, data management and artificial intelligence tools.

A Library-CTSI Collaboration to Support Researcher Compliance with the 2023 NIH Policy for Data Management and Sharing

Track(s): Information Services

Andrea Denton, Research & Data Services Manager, Claude Moore Health Sciences Library

Lucy Carr Jones, Research Data and Scholarly Communications Librarian, University of Virginia Health Sciences Library

Bart Ragon, University of Virginia, University of Virginia

Background:
Seeking ways to support teams in the preparation for and the implementation of the new National Institutes of Health (NIH) Policy for Data Management and Sharing (DMSP), a CTSI partnered with a Health Sciences Library to develop training and resources for university researchers.

Description:
Health sciences librarians and the CTSI NIH-NCATS supported Clinical Translational Research Institute convened a Working Group, inviting representatives from central and unit-specific research support offices (e.g. the Comprehensive Cancer Center), research compliance, regulatory affairs, sponsored programs, institutional review boards, libraries, and data science to review and discuss the DMSP requirements. After an initial orientation to the policy, the group reviewed existing public resources and solicited feedback about steps to best support university researchers in compliance. Leveraging the broad expertise of the group, the team provides guidance to researchers on writing the DMS plan and choosing a data repository, and collaborates to develop tools and templates to support implementation of the policy. A library-created website provided policy guidance, including links to NIH-hosted information, resources created by other institutions, and new university-specific templates and suggested proposal language. Librarians led a webinar on the new policy and resources which included a speaker from regulatory affairs to describe the new DMSP requirements, and a tour of the new guide.

Program Conclusions:
The library-hosted guide has been viewed over 5000 times to date and librarians have provided consultations to individual researchers on a variety of topics including data management plan authorship and data submission to discipline-based and generalist data platforms and repositories. Librarians have also provided customized training to departmental staff and administrators. Current plans include developing a user satisfaction survey, reviewing DMSP feedback from submitted proposals, and incorporating lessons learned into the website and future training. The collaboration between the CTSI and the library to support the NIH Data Management and Sharing Policy was a successful partnership.

Mindful Curation: Mastering Resource Evaluation in Academia

Track(s): Information Management

Holly Hubenschmidt, Director of Instruction and Research Services, Webster University Library

Heidi Vix, Director of Resources and Access Services, Webster University Library

Background:
At any academic library, it is important to manage the collection to ensure the current programs and courses are being supported and faculty have materials for research. At Webster University Library, the librarians began a four-year resource review cycle in 2009. By cyclically reviewing different areas of the collection, the library had an opportunity to ensure the materials provided remained relevant to the mission and needs of the patrons. The librarians could also ensure the budget provided was being spent efficiently as the review examined cost per use and overlap analysis to compare with other resources.

Description:
Librarians hoped a cyclical review of databases, stacks, journals, and standing orders would help keep our resources timely and our budget tight.

We began in 2009/2010 with a comprehensive database review with search comparisons, overlap analysis, usage, accreditation need, ongoing platform issues, and cost.

The stacks review in 2010/2011 compiled item age, usage, duplication, and older editions including information like publication date, date of last use, and usage.

In 2012 librarians reviewed approximately 1,000 journal titles, pulling format (print or electronic), package deals, and prices.

The 2013/2014 reference collection and standing order review examined print reference, electronic reference packages, and standing orders. We eliminated most standing orders and retired much of our aging reference collection.

This cycle continued until 2018, when budgetary constraints obliged us to review all subscriptions.

As we resurrect the review cycle, we are broadening our ideas to include a review/possible redesign of the library website and a more intentional, collaborative triennial user survey to inform updates to our strategic plan in addition to database and journal reviews. Weeding of books/eBooks is being incorporated into regular subject librarian duties, and the reference collection is no longer significant enough to merit a cyclical review.

Program Conclusions:
With this process, we saw cost and space savings:

The 2010 database review resulted in savings of 11% of our budget.

Our stacks review resulted in approximately 24,000 items weeded.

The journal review allowed us to cancel 84 titles and update 36 titles to include online access.

The SO/reference review resulted in the cancellation of 218 standing orders and approximately 2,100 withdrawals from the reference collection.

Subject librarians deepened their knowledge of our resources and collections. The second cycle of the review process from 2014 to 2018 progressed to more efficient methods of data collection and prioritizing electronic access.

The Nature of Change: Navigating Organizational Upheaval Through Teamwork

Track(s): Professionalism & Leadership

Lauren Robinson, Associate Director of Research Services, University of Kentucky Medical Center Library

Stephanie Henderson, Association Director of Outreach and Engagement, University of Kentucky

Beth Reeder, Assistant Director of Operations & Public Services, University of Kentucky

Cayla Robinson, University of Kentucky, University of Kentucky

Rebecca Morgan, Health Sciences Library, University of Kentucky

Background:
Since 2020, our university library system has undergone multiple organizational restructurings, while our health sciences library has rapidly changed due to retirements, mergers, and internal restructuring. This constant upheaval has impacted many aspects of how we provide services. We will provide an outline of the steps of the reorganization and the culminating changes to services and programs offered by the health sciences library. We will discuss how teamwork has contributed to our adaptation.

Description:
The university library system underwent two restructurings, and the health sciences library and science library were to merge into one reporting structure. Due to a high-level retirement, the library system pivoted, and the health sciences library became a solitary team under a new Associate Dean model.

Internally, the process started with the closure and merger of an agriculture branch library into the health sciences library. Following the absorption of an additional library, several changes to personnel and job duties ensued. In 2021, the InterLibrary Loan departments from the main campus library and the health sciences library merged into one department and removed the separate ILLiad account for the health sciences library. In 2022, the campus wide liaison librarian structure was revamped to include the health sciences librarians. Liaison core competences and best practices were developed over ten months. The new liaison structure includes a team-based approach which requires upskilling faculty and staff in the health sciences library. In 2023, an external consultant was hired to evaluate strengths and opportunities of the medical center library. As a result, a new organizational structure was implemented. In addition, the agriculture, food and environment library services will be absorbed by the STEM liaison team and moved from the health sciences library.

Program Conclusions:
Since this is an ongoing process, no formal evaluation has taken place. This poster examines the process and planning for evaluation, including communication workflows, team meetings, and time management. With these changes the health sciences librarians have begun discussing the implementation of a fully team-based approach. This approach would transition us away from one liaison to one college model. We plan to build upon our existing collaborative team structure and implement a formalized team model. We hope to evaluate this new model in the years to come.

Outcomes

The next steps will be to establish the new leadership team and begin redesigning the administrative processes within the health science library. Implementation of a formalized team-based model will allow for growth and much needed stability.

No Need to Reinvent the Wheel: Revising Existing Library Tools to Increase Student Engagement with Problem-Based Learning Resources

Track(s): Information Services

Jennifer Silverman, Health Sciences Librarian, University of Southern California

Background:
At the university, dental students participate in an integrated curriculum called Problem-Based Learning (PBL). PBL students study patient cases in small groups to learn about the biomedical sciences. The library has actively supported PBL student research and investigation of Learning Needs for many years through recommended texts, databases, and research guides. In Fall 2023, a project to review and revise these

tools was launched. The purpose of this project was to review current PBL library tools for their continued relevance to student needs and explore new ideas in an effort to increase student engagement with the tools.

Description:
The objective of this project was to find ways to increase student engagement with existing PBL library tools by making them more user-friendly. This was accomplished through collaboration with PBL faculty and making minor navigation changes to the PBL landing page and PBL Libguides. A PBL faculty member explained the need for books displayed on PBL Libguides to be grouped by biomedical subject areas. Suggestions for layout and content edits were also implemented. The homepage of each guide now includes: case-relevant textbooks, a link to the library’s faculty-approved List of Recommended Textbooks, and a link to the new PBL Learning Needs Research Checklist. Moving this content to the homepage of each guide was intended to minimize the number of clicks needed to reach the most relevant PBL resources. The PBL Learning Needs Research Checklist was a new tool created during this time in order to provide students with a one page document outlining a process for how to prioritize the use of library resources to answer their learning needs. A webinar was held to demonstrate how to use the PBL Learning Needs Research Checklist.

Program Conclusions:
For this project, student engagement is measured by the number of unique views of each tool. Compared to Fall 2022, the current data indicates there was an increase in the number of unique views during Fall 2023. It is challenging to definitively conclude whether or not the changes made to the PBL tools directly led to increased student engagement, since they have only existed for one semester, but the increase in number of views suggests more students are engaging with the tools. I will continue to track the data to examine trends over a longer period of time. Health Sciences librarians will be interested to learn if making similar revisions to their own library tools can lead to increased student engagement.

One for All and All for One: Wrangling our Usage of LibGuides

Track(s): Information Management

Brandi Tuttle, Research & Education Librarian, Duke University Medical Center Library & Archives

Erin Simon, Research & Education Intern, Duke University Medical Center Library & Archives

Sarah Cantrell, Associate Director for Research and Education, Duke University Medical Center Library & Archives

Background:
Springshare LibGuides are currently used by the Library to offer customized guides on a variety of topics or geared toward the needs of different patron types. When the Library adopted this system in 2008, no specific guidelines were created to direct the formation and maintenance of guides. The system was easy to use, and a plethora of guides were created by many staff members. Over time and with staffing changes, it became clear that more guidance was needed as some of the guides became outdated, minimally updated, or unused. Further, we observed that we were not using the strengths of the system, such as link checking, asset management, and standardization options.

Description:
This project has a tripartite aim: to clean up the guides, establish guidelines and best practices, and offer continuing education to staff. As part of a larger Library goal to facilitate patrons' ability to learn online any time, one librarian made it a personal goal to deepen their understanding of the LibGuides backend and capabilities. Other steps taken include creating the Database A-Z list, merging duplicate assets, handling broken links, reviewing guide ownership, and offering support to staff. Thus far, more than a third of the

guides and more than half of the assets were purged from the system. Statistics are used to inform some decisions regarding updating or purging guides, links, and information. Some staff inherited guides due to staffing changes but felt no real ownership or connection to the guide which hinders guide maintenance. In order to ensure staff were aware of their guide responsibilities and specific issues, monthly LibGuide Clean-up Parties create space for individuals to work on their guides amongst colleagues. As work in LibGuides continues, best practices and standards for our guides are being developed as well as training for staff in the use of the many helpful features within the system.

Program Conclusions:
This project is working towards creating a standard set of practices and education materials for staff to follow when using LibGuides. All broken links were addressed, and most database links were changed from link assets to database assets via the A-Z Database list. As this project progresses, data will continue to be collected regarding the specific changes made and will be shared as well as other lessons learned.

One Hospital Library’s Support for Their Nursing Research Council: Providing Both the Pebbles and Cement

Track(s): Clinical Support, Education, Information Management, Information Services, Innovation & Research Practice, Professionalism & Leadership

Edward Donnald, Director, Medical Library, Cone Health

Background:
Purpose

This poster highlights resources the medical library has created specifically to support and improve the clinical nurse’s experience with research, scientific inquiry, and dissemination.

Setting/Participants/Resources

The medical library approached the Nursing Research Council (NRC) and offered to collaborate in the creation, promotion, and support of the nursing research activities of a 1,200-bed health system. The librarian accepted a standing position in the NRC and is responsible for several tools and resources used by the NRC.

Description:
This poster highlights several of the ways the medical library has collaborated with the NRC to create and provide multiple resources in support of nursing research. These include enhanced resources on the library’s online portal; building and maintaining a SharePoint site; proposing and building a decisional flowchart for interested nurse researchers looking to get started; providing a turn-key solution for the creation and use of surveys for collecting data; designing, facilitating the creation of and providing ongoing support for an online poster review tool; and providing graphical and visual review services for posters before being printed. The director of nursing research, along with the members of the NRC, continue to be appreciative of the library’s contributions.

Program Conclusions:
The tools and resources provided by the library have improved engagement, reduced anxiousness, and empowered nurses in their journeys, addressing quality improvement and evidence-based practice and adding to the knowledge base for the benefit of others. The administrative functions of the NRC have improved, and nurses are appreciating the tools and resources being provided to elevate their work.

Evaluation Method

The collection of tools and resources exceeds the parameters of a single project and evaluation. The larger longitudinal relationship between the library and the NRC relies on ongoing quantitative feedback from the NRC, which allows for nimble responses for improvements in the tools and resources, with the director of nursing research acting as the primary driver for determining success.

The Ongoing Struggle to Get It Right: Teaching Information Fluency to the First-Year Medical Students with a Case-Based Approach

Track(s): Education

Connie Wong, Stanford University, School of Medicine

Evans Whitaker, Reference Librarian, Lane Medical Library, Stanford University

Alok Patel, Stanford Medicine Children's Health

Amanda Woodward, Web Services Librarian, Stanford Lane Medical Library

Background:
Traditionally, medical librarians delivered PubMed searching as part of the faculty-led information literacy sessions to first-year medical and physician assistant students, with varying success. The sessions were criticized for being dry and failing to resonate with Gen Z learners, who prefer interactive activities and immediate relevance to their clinical studies (Shorey et al., 2021). Recognizing this disconnect, the Library sought to revamp its approach, focusing on creating engaging and practical information literacy sessions for our students.

Description:
Our revamped program comprises two 90-minute sessions during the first week of the Winter Quarter. We adopted a case-based approach, using a single clinical case of an uninsured new-immigrant patient as the thread, weaving cultural competency and knowledge on acquiring the best evidence progressively. In Session One, students are introduced to the "Ask" and "Acquire" steps of Evidence-Based Medicine, focusing on PICO question formation and point-of-care (POC) tools like DxPlain, LexiComp, and UpToDate for differential diagnosis and clinical decision-making. Students work in small groups, refining the patient's and their information needs and using different POC tools to develop plans for this limited-resource patient. Session Two builds on this foundation with students finding evidence using PubMed and critical appraisal tools to support their clinical recommendations when they faced the patient’s discordant test results and medical myths leading to the patient’s non-compliance. A pediatric hospitalist joins the session, sharing real-life testimonies on how the class materials impact his patient care and application with medical journalism practice. By bridging the gap between in-person learning and independent exploration, library guides and asynchronous case-based exercises embedded in the Canvas course site empower students to deepen their understanding at their own pace.

Program Conclusions:
Initial feedback suggested that the revamped sessions successfully engage students by transforming the traditional teacher-centered information literacy lectures into a captivating patient case-based exercise. By focusing on practical skills and immediate clinical relevance, students honed critical information literacy skills and gained tangible tools for serving diverse patient groups with cultural awareness. This student-centered, interactive approach involved the students and librarians tackling a real-life clinical case, which not only helped students solidify their information literacy skills but also saw the Library as a vital resource for their clinical journey. Additional exercises and Library Guides with ongoing updates embedded in the Canvas

course page provide opportunities to offer students new materials beyond the classroom.

Open Access Programming: Building a Strong Foundation

Track(s): Information Services

Sharon Duffy, Research Services Librarian, LSU Health Sciences Center - New Orleans

David Trillo, Instruction & Reference Librarian, Touro University Nevada

Background:
LSU Health Sciences Center Libraries hosted its first International Open Access Week in October 2023. Our goals were twofold: educate our health science community about Open Access publishing and Open Science initiatives; and raise the profile of the Libraries within the institution. It was also an opportunity to collaborate with a partner medical school library.

Events were held at both campuses of the LSUHSC-New Orleans libraries and involved all librarians and staff. The events included open houses on both campuses, an open-access themed escape room, hybrid presentations on open science themed topics, and a documentary viewing. An NNLM Exhibit Grant was used to fund the materials and activities. Librarians from our partner library were invited to participate in all events.

Description:
International Open Access Week is promoted annually by SPARC, a non-profit advocacy organization, to raise awareness about the benefits of open access publishing and open science practices. LSUHSC-NO Libraries took this opportunity to put together a large scale promotion event to reach out to faculty, researchers, staff, and students to educate them about this topic. The Libraries have been moving towards a service-based model and Open Access was seen as a broad topic that could reach all members of our community and generate excitement about the new library services being offered. Librarians from two health science institutions made presentations on a wide variety of open science topics. These were held in person and online and the recordings were posted in the LSU institutional repository. An escape room was put together for a hands-on in-person learning experience about open access publishing. Open houses were held at both campuses with poster displays and information about scholarly publishing. A documentary about the business side of scientific publishing was screened and discussed. The Exhibit Award from NNLM was secured to fund the materials needed for the open house and escape room activities.

Program Conclusions:
The outcomes of this event include the successful production of an engaging escape room for students and faculty along with recorded presentations on open science topics. Posters and display materials will be used in future events. The event was successful at strengthening the professional ties between the two medical library staffs and engaging with our institution. A lesson learned was this particular week in October was not an optimal time for our institution as some programs were not in session. Future events will focus on specific audiences and spreading the events over a month time period. In 2024, we will continue our partnership and hold weekly events at both institutions.

Overcoming the Challenge of Finding Comics Studies in Medline

Track(s): Information Services

William Jacobs, Librarian for STEM and Comics, San Francisco State University

Background:
Comics are increasingly being used to communicate health information. (Kearns, Ciléin, and Nethmi Kearns, 2020) This has created a need to search the literature for prior work. However, the lack of settled vocabulary in the field and a poor fit into MeSH terms means that such searches are not simple, and important studies easily missed.

Kearns, Ciléin, and Nethmi Kearns. "The role of comics in public health communication during the COVID-19 pandemic." Journal of visual communication in medicine 43.3 (2020): 139-149.

Description:
During a recent systematic review in this area, I found each comics-related term to have its own peculiarities of usage in medical studies, and required particular search techniques to compensate. I developed a successful search strategy that I would like to share in this poster.

Program Conclusions:
Viewers of my poster will learn the technical usage of various terms, such as ‘graphic medicine’, ‘cartoons’, and ‘manga’, and the search strategies required to deal with them. They will also be able to access, through a QR-code, a successful Medline search string.

The information will be presented in the form of a comic strip, making good use of the visual poster format.

Practical Teaching, Practical Skills: The Librarian's Role in Clinical and Translational Research Courses for Dental Residents

Track(s): Education, Innovation & Research Practice

Rachel Becker-Simpson, Branch Librarian, University of Rochester Medical Center

Background:
An interdisciplinary teaching team at a university dental center designed a course to introduce dental residents to hard and soft skills needed to conduct clinical and translational oral health research. Course outcomes included developing meaningful research questions, designing clinical and translational studies, understanding applicable research tools, converting the data into publications, and communicating with scientific and non-scientific audiences.

Description:
The course, titled Practical Skills in Conducting Clinical and Translational Research for Oral Health (PS-CTSO) was delivered in workshop format held once per month for 4 months during the fall 2023 semester. The librarian taught 2 interactive sessions, aimed at enhancing students' literature searching skills, knowledge of evidence-based dentistry concepts and understanding of the scholarly publishing process.

Program Conclusions:
PS-CTSO's post-course assessment showed largely favorable responses from students, with a few suggestions regarding breadth of content and optimal session length. The course has been incorporated into the residency curriculum, and will be offered again next fall. The librarian plans to develop more specific, measureable questions to be included in the pre- and post- surveys to better assess students' learning outcomes.

Practical Time-Saving Applications of ChatGPT for Clerkship Faculty

Track(s): Education, Innovation & Research Practice, Professionalism & Leadership

Martin Wood, Director, Medical Library and Medical Informatics Education, Florida State University

Terri Johnson, Informatics Librarian, Charlotte Edwards Maguire Medical Library & Medical Informatics Education, Florida State University

Background:
The purpose of these activities aim to provide a solution to the amount of time required by clerkship faculty to document medical student accomplishments in clerkship rotations and clinical research, especially for the creation of letters of recommendation that students rely on for residency interviews and placement.

Description:
We developed this faculty development opportunity based on feedback from clerkship faculty who expressed that too much time was spent on letters of recommendation for students, forcing them to create standardized letters that they felt did not fully feature student accomplishments. As a study of feasibility, we took de-identified quantitative clinical encounter reports from student documentation systems, coupled with any research that students participated in while in medical education, and used ChatGPT to stitch those two data sources together. We then engaged with ChatGPT to use this new data source to write a unique and varied letters of recommendation for students that allowed clerkship faculty to review and make minor edits.

Program Conclusions:
Outcomes from this feasibility study toward the practical implication of faculty development are forthcoming, and show promise of a significant time savings for clerkship faculty while improving the quality of student letters of recommendation. Further development of ChatGPT, and other generative A.I. tools are expected to lead to additional enhancements to student documentation in the near future.

QR Codes: Bridging the Gap in 24/7 Library Accessibility

Track(s): Information Services

Ashley Curran, Library Branch Manager, NYU Langone Health

David DeSimone, Branch Manager, Robbins Library, NYU Langone Health

Background:
Previously the library was open only during normal business hours, Monday through Friday, 9-5pm. After extensive remodeling we reopened as a 24/7 space, with swipe card access to medical center staff. Library staffing remained unchanged, and it became clear that questions we were receiving at the service desk during business hours were also being asked after hours with no one available to respond. If we are to offer branches that are open 24/7, we need to ensure that support is always available.

Description:
This poster shows the process we used to determine what questions were likely being asked during off-hours, whether a QR code was appropriate for a response and how and where we decided to house this information. It explains the decision-making process we took to ensure that the information could be updated in a simple and timely fashion, without involving multiple departments or medical center IT. We also discuss

the decision to expand this system to a second branch with only one full-time staff member and how we are seeing the potential for this system in a third fully unstaffed branch.

Program Conclusions:
We plan to study the QR code data to gauge the success of this program, especially as it applies to the completely unstaffed branch. This data will include overall usage of the QR codes, time of usage, and location. We will then use the data to inform decision making going forward in how we continue to make our library accessible.

Reaching Pediatricians at the Point-of-Care via ClinicalQ, an EHR-Embedded Clinical Question Support Service

Track(s): Clinical Support

Christopher Stave, Graduate/Clinical Education Librarian, Lane Medical Library, Stanford University

Connie Wong, Medical Education Librarian, Stanford University

Background:
A big challenge for any medical library is creating and maintaining a sustained program of support services for health providers close to the point-of-care. Synchronous, in-person rounding with a clinical librarian is time-intensive and can create significant staffing challenges, particularly when trying to expand the service beyond a small number of clinical teams. Reflecting on these challenges during a strategic planning retreat, the library’s Research & Instruction team proposed an initial six-month pilot from June to November 2023 aimed at exploring and evaluating the efficacy of an electronic health record (EHR) accessible clinical support service for the Pediatric Hospital Medicine (PHM) division and their inpatient clinical teams.

Description:
The key elements of the pilot included: 1) leveraging the expertise and advocacy of the library’s physician clinical liaison to develop and promote the pilot; 2) weekly in-person rounding with multiple clinical team members (medical students, residents, fellows, and attendings) to understand the needs of their workflows; 3) enhancing communication between the library and the clinical teams via Voalte, the hospital’s inpatient communication technology application; 4) direct integration into the hospital’s EHR via an asynchronous link to ClinicalQ, the library’s clinical support service; and 5) responding to questions submitted to the ClinicalQ team via a dedicated listserv.

The evaluation of the pilot will include the following elements: 1) total number of questions asked, both during rounds, via Voalte, email, and the embedded ClinicalQ link; 2) average time spent answering the questions by research librarians; and 3) a ClinicalQ user satisfaction survey.

Program Conclusions:
In addition to evaluation elements described above, the ClinicalQ user satisfaction survey will specifically capture: 1) training levels of respondents (resident, attending, etc.); 2) usage (or not) of ClinicalQ; 3) frequency of usage; 4) time-to-response; and 5) impact on clinical decision making/patient care.

Future directions of the service could include: 1) offering CME for using and reflecting on the information provided by ClinicalQ; 2) promoting the service more broadly throughout the entire Department of Pediatrics; and 3) extending the service to other departments/hospitals.

A poster is an ideal format for visually representing the timeline and key milestones for the roll-out and subsequent evaluation of the ClinicalQ program pilot.

Reflecting on Inclusivity and Representation in Instruction Examples

Track(s): Education

Eden Kinzel, Liaison & Education Librarian, University of Toronto

Background:
Most teaching sessions include example scenarios designed by librarians that they utilize when introducing PICO or another searching concept. These scenarios typically involve a fictional person with some type of medical condition or health-related question that they then will search the literature to answer, but more importantly to demonstrate specific search techniques. In the past, the author of this poster has used scenarios created by previous librarians that have been handed down for years. However, the author noticed many included a lack of diversity in ability, race, ethnicity, gender identity, and sexual orientation, so they created a reflective process they utilize when designing new example scenarios to improve inclusivity in those areas.

Description:
The poster will expand upon the questions the author asks themselves when creating examples for instruction sessions, some of which include: Are all of the names used from the same cultural background? What pronouns have been included in the examples? Are there only heterosexual couples mentioned in the examples? Is the terminology/phrasing inclusive (ex. people who menstruate vs menstruating women)? Is there an opportunity to explicitly involve a marginalized community (ex. What are strategies for making primary care offices trans-inclusive spaces?)? Are the images that accompany the scenarios representative of a range of body sizes, types of ability, and race? This is not a checklist for thoughtlessly ticking some type of box, but an active process requiring reflection, questioning what you’ve done before, and creating an environment that more appropriately represents the lived experience of your audience.

Program Conclusions:
This reflective practice has enabled the author to create more inclusive in class instructional examples. It can also be adapted and applied to other areas of professional practice and may be relevant to other types of librarianship that also use personalized and fictitious scenarios during search demonstrations. While formal evaluation has not yet taken place, anecdotally, the author has found students to be less hesitant to discuss instances of racism, ableism, homophobia, or other types of discrimination as potential search topics. Additionally, they have seemed (and noted to the author that they felt) more comfortable having a post-instruction session consult when their topic was related to an underrepresented community, in particular, questions related to the 2SLGTBQ+ community.

Research Skills Education Framework : Online Library Training in a Clinical Setting

Track(s): Education

Alice Anderson, Director, Library Services, Monash Health

Background:
The Research Skills Education Framework includes library training on core research skills from developing a research question to critical appraisal alongside 'special events' series on research promotion, scholarly publishing and emerging Ai tools for health research.

Key aims of the program include: certification for participants to acknowledge achievement of research skills capabilities; integration of self-directed learning modules that meet learners at the point of need; embedded in existing medical training programs; customisations for different professional groups and levels of research capability.

The educational context is online adult learning in a health care environment, designed and delivered by a hospital library to doctors, nurses and allied health professionals.

Description:
The Research Skills Education Framework was designed following a shift towards online delivery in the pandemic, which became a lasting change. The new program was implemented by adapting synchronous traditional in-person library training to virtual modules with a range of delivery modes. The convenience of online education led to an expansion of modules and introduction of special events streams. The program is now organised into a Research Skills Education Framework which has flexibility for continuous development and improvement.

Detailed analytics have been gathered since inception to provide data about utilisation. In 2023 for example, the library trained 1627 participants, a 74% increase on the previous year, with a further 865 views of on-demand recorded content. Participant evaluations have been used to inform and iteratively improve the program and individual learning modules. An organisational wide library survey in 2022 provided additional feedback from the library’s user base.

Implementation relied on a range of platforms such as MS Teams, Canva, PowerPoint, YouTube and library specific software Lib Guides and Lib Cal. Interactive learning tools are also employed including polls, menti and Easy Generator quizzes. All education in the framework has been designed to overcome a range of challenges relating to organisational context which are common in health care.

Program Conclusions:
The Research Skills Education Framework has been successfully implemented as a high-quality, effective library training program in a clinical environment. It was developed by a hospital library team learning and emerging from the pandemic.

The framework is delivered by librarians to multiple hospital campuses. It includes core modules and special series on non-core topics and emerging issues for researchers. It is delivered as digital education, with meaningful learning experiences in the online environment. The program’s success is evidenced by participation, analytics and evaluations.

The framework is helping to build research skills capability across a busy public health service, particularly for junior clinicians and emerging researchers. It has firmly established the library as educators and research experts.

‘Righting’ the Story: Libraries as Safe Spaces for Narrative Inquiry and Re-writing Stories About Equitable Oral Health

Track(s): Education, Health Equity & Global Health, Innovation & Research Practice

Eileen Harrington, Assistant Director & Health and Life Sciences Librarian, Priddy Library at The Universities at Shady Grove/The University of Maryland, College Park Libraries

Andi Sauer, University of Maryland, College Park

Anubhuti Shukla, Indiana University School of Dentistry

Esperanza Angeles Martinez Mier, Indiana University School of Dentistry

Michelle McQuistan, University of Iowa College of Dentistry

Michelle Moncrieffe, Lecturer, English Department, University of Maryland, College Park

Sheryl L. Syme, University of Maryland School of Dentistry

Background:
Public libraries have long provided access to high quality health information and are seen as a safe, neutral space. Access to regular and basic oral health care remains a challenge for many racial and ethnic minorities, who might also feel intimidated or uncomfortable in standard clinical settings. This project led by a multidisciplinary team of researchers seeks to better understand and address racism in dentistry. The overall objective of the project is to bring together communities, dental schools, undergraduate pre-dentistry/health, and journalism students in public libraries to create holistic and accurate narratives that reveal a fuller account of the dental care experiences of racial and ethnic minorities and the trainee clinicians charged with providing equitable care.

Description:
The project consists of four components: Oral health fairs at public libraries that connect underserved populations to oral health care; provide experiential learning opportunities for dental and dental hygiene students; and serve as a way to recruit participants for community focus groups. We held 10 oral health fairs in California, Indiana, Iowa, and Maryland. Focus groups with community members in public libraries to gain insights into their experiences accessing oral health care. Separate focus groups were held with dental and dental hygiene students to explore their experiences treating patients from underserved populations. We held 8 focus groups. Narrative dentistry workshops that provide space for dental and dental hygiene students to respond to and reflect on narratives developed from focus group data and other sources. We have held one virtual narrative dentistry workshop. Database and curriculum development: we are working on developing an open source database to share the stories and narrative extracts that we gather through this project and allow others to add theirs. We also hope to create activities for use with students in various disciplines to explore themes around access to oral health care, equity in health care, oral health literacy and promotion, and health-related journalism.

Program Conclusions:
We gained several valuable insights about the feasibility and positive impacts of oral health fairs at public libraries. We are currently analyzing the focus group transcripts to glean a deeper understanding of issues around oral health care. Preliminary themes that have emerged center around access to care, managing patient expectations, race and racism/bias, patient advocacy/education, pain, patient fears, trust and cost. Participants described public libraries as ideal locations for them to meet with clinicians. For the narrative dentistry workshop, we had participants complete pre and post-surveys so we will be using those to assess their understanding of systemic barriers related to oral health care for ethnic and racial minorities and what roles they can play in mitigating these.

Rural Hospital Library Navigation System Design Concept

Track(s): Information Services, Innovation & Research Practice

Kuo-Liang Huang, Application of library information multimedia navigation machine in rural hospital library, National Taiwan University, BioMedical Park Hospital, Chu-Tung Campus

Background:
.Our library lacks dedicated computers for collection inquiries and library access statistics. In order to solve this problem, I took advantage of the fact that the hospital does not use touch computers and designed a cost-effective navigation system with information links using Canva. Rural hospital library navigation system

design concept

Description:
Design for Four Major Areas:Tailored to the hospital's evaluation and library needs, the system is structured around four key areas: reader registration, e-book recommendations, new book notifications, and service regulations with multimedia integration.

E-Book Recommendation 系統:Implemented a recommendation system using Google Blog and tags. These tags, categorized by occupation, facilitate quick access to test books or topics of interest for readers.

New Book Notifications:Employed Heyzine Flipbooks to deliver new book notifications with a page-turning e-book effect, enhancing the interactive reading experience.

Service Regulations:

Encompasses various interlibrary services, library rules, and guidelines on utilizing electronic resources outside the hospital. This section enables librarians to promptly display relevant information to readers.

Multimedia Functionality:Integrates touch controls to provide multimedia功能s such as educational videos, classical music, and foreign language audio book links. This enhances the overall user experience and engagement.

Program Conclusions:
By implementing this comprehensive navigation system, our library aims to enhance accessibility, reader engagement, and the overall library experience for our hospital community."

Scoping Out What Our Customers Need: Development of a Scoping Review Toolkit

Track(s): Information Services

Alicia A. Livinski, Biomedical Librarian, National Institutes of Health Library, Office of Research Services, U.S. Department of Health and Human Services (HHS)

Gisela M. Butera, Biomedical Librarian, National Institutes of Health (NIH)

Jaime Friel Blanck, Chief, Consulting and Training Branch, National Institutes of Health Library (NIH), Office of Research Services, U.S. Department of Health and Human Services (HHS)

Nancy Terry, Biomedical Librarian, National Institutes of Health Library (NIH), Office of Research Services, U.S. Department of Health and Human Services (HHS)

Nancy L. Muir, Library Director, National Institutes of Health Library (NIH), Office of Research Services, U.S. Department of Health and Human Services (HHS)

Stacy Brody, Biomedical Librarian, National Institutes of Health Library (NIH), Office of Research Services, U.S. Department of Health and Human Services (HHS)

Tracy C. Shields, Biomedical Librarian, National Institutes of Health Library (NIH), Office of Research Services, U.S. Department of Health and Human Services (HHS)

Background:
Scoping reviews are a distinct evidence synthesis methodology which require unique support and tools to be completed properly. Over the last several years, we have experienced an increase in interest from our users in conducting scoping reviews. To support the increased interest and demand from fellows, trainees, clinicians, and researchers to conduct scoping reviews, a five-member evidence synthesis team from a federal biomedical library developed a toolkit. We aim to describe the scoping review toolkit and how it is used with our users. We will also share data on its use to date and user feedback.

Description:
The scoping review toolkit includes an intake checklist, protocol template and guidance document, video series, and training classes. We share the toolkit and examples of well-written, published protocols with each review team. The intake checklist is used to gather details about the proposed review (e.g., research question, suggested keywords, key articles, eligibility criteria) and includes recommended resources.

The protocol template and guidance document were created through a collaboration with fellows in the Rehabilitation Medicine Department to address challenges encountered while writing their scoping review protocols. The two documents were adapted from Preferred Reporting Items for Systematic Reviews and Meta-Analyses extension for Protocols (PRISMA-P), PRISMA extension for scoping reviews (ScR), and JBI Manual for Evidence Synthesis. The guidance document includes links to resources, published scoping reviews, and tips on information to include. To facilitate sharing, the two documents are in Open Science Framework (OSF).

We collaborated with the instructional design librarian to develop a series of four short videos using Camtasia on writing the protocol through registration in OSF. Additionally, classes on writing review protocols and scoping review methods are taught. These two classes are part of an existing series of evidence synthesis classes taught several ti

Program Conclusions:
In 2024, we will develop a research guide on the library’s website to provide toolkit materials in a central location to our users. A marketing campaign to advertise the research guide and support specifically available for scoping reviews to our users will be planned and implemented. We will share data on access of the template and guidance document in OSF, video usage and user feedback received to date before and after the marketing campaign, and number of classes taught on scoping review methods and protocol writing and attendees. Throughout the year, we will continue to listen to our users on the support they need to conduct rigorous and quality scoping reviews and to improve and expand the toolkit.

Sharing a (Cyber) Cup: Using Virtual Coffee Chats to Reengage Participation and Promote Connections

Track(s): Professionalism & Leadership

Judy Kraemer, Librarian, Anaheim Public Library

Angela Murrell, Research & Learning Librarian, University of Arizona

Background:
While the shift to work from home during the pandemic was a benefit for some librarians, many had to work alone in their libraries, masked and distanced, with conferences canceled and meetings moved online. The personal sense of belonging faded and many felt isolated. Organizations saw a decline in participation. As mandates lifted, professional networking had changed, leaving many feeling left out. Zoom became the new normal, group meetings were no longer the chance to socialize and meet members. People became burnt out and stopped attending optional online meetings. Webinars and virtual meetings, designed to be

inclusive, seemed to lose their appeal. We needed to find a way to reengage members and promote the connections we had pre-pandemic.

Description:
The Coffee Chat was created to reconnect members while also providing a space to discuss member-generated topics of interest in a semi-structured and relaxed environment. We wanted to enable the sharing of member knowledge and experience. Zoom was used to ensure an accessible environment for members regardless of location. Instead of formal presentations, one or more members were asked to facilitate an informal discussion. Facilitators were simply asked to share with a few questions or resources to get the discussion going. The chats were meant to be free flowing discussions with no one person singled out as the "expert." This format allowed those with limited to no allocated research time, such as solo librarians, to volunteer as facilitators.

Participants often discussed topics related to morale and professional wellbeing (e.g., burnout, saying 'no'). These are areas of increasing concern across librarianship, especially for their impact on retention and success of staff from historically marginalized populations. Through the chats, members could find mutual support, problem-solving, and a sense of community and belonging built around the shared wisdom and experiences of all participants.

A Qualtrics survey was used to evaluate the professional development usefulness and networking value of the Coffee Chats.

Program Conclusions:
The assessment revealed that most participants found the Coffee Chats were very or extremely useful in networking with colleagues and feeling connected, and moderately, very, or extremely useful for supporting personal development. One participant commented that they find the chats helpful as a way of "[h]earing from librarians at different places about similar challenges and how they do things."

The Coffee Chats provided opportunities for members to share their experience and knowledge in an accessible environment where anyone could participate regardless of location or dedicated time for preparation. The chats created connections with participants from different backgrounds, in different types of libraries, and at different stages of their careers.

Stronger Together: Librarian Involvement with Residents and Fellows

Track(s): Education

Lisa Marks, Director, Library Services, Mayo Clinic in Arizona

Neera Agrwal, Consultant, Assistant Professor of Medicine, Mayo Clinic

Background:
The MERIT (Institution Name Evidence-based Clinical Practice, Research, Informatics, and Training) presentation is an hour-long session focused on evidence-based medicine, tailored for trainees in various medical programs. It revolves around a clinical question selected by the trainees, aiming to enhance learning and integrate this knowledge into clinical practice. In this process, a trainee resident formulates a specific question, then collaborates with a medical librarian and an expert to thoroughly research medical literature. This research involves critically evaluating the literature using standardized methods to assess its relevance and applicability in clinical settings.

Description:

This poster outlines the librarian's role in a program designed for PGY-3 (Post-Graduate Year-3) residents in internal medicine, neurology, and gastroenterology fellows at our institution. Trainees, inspired by a clinical encounter, employ the PICO format (Patient/Problem, Intervention, Comparison, Outcomes) to formulate a searchable clinical question. They, then, search and critically appraise the medical literature, ultimately presenting their findings to enhance clinical practice. The librarian assists each resident in conducting a literature search, aiming to find evidence-based references pertinent to the case. Trainees address several key questions: the clinical bottom line, the validity of study results, applicability to the specific encounter or scenario, and whether the findings will alter the trainee's clinical practice. A key aspect of the librarian’s role involves teaching trainees to conduct their own literature searches, which are then refined with the librarian's expertise. This process is crucial for teaching the importance of searching for the best articles, so that it may be followed by critical appraisal of articles and presenting information as best practices for clinicians. This program satisfies a key component of the Practice Based Learning Improvement competency for trainees at all levels

Program Conclusions:
The program initially included internal medicine and neurology residents and has expanded to incorporate gastroenterology fellows. The librarian has significantly contributed to numerous MERIT projects across these specialties (The librarian has assisted on approximately 120 internal medicine, 65 neurology, and 10 gastroenterology MERIT projects). Occasionally, at the program director's discretion, residents may be encouraged to publish their findings. In such instances, the librarian contributes to writing the methodology section and has been credited as an author on several publications (5- internal medicine, 13- neurology) of these critically appraised topics.

Stronger Together: Supporting Early Career Librarians and Librarians New to Health Sciences, an MLA Chapter Task Force

Track(s): Professionalism & Leadership

Kristi Torp, NLM Associate Fellow

Casey Phillips, Biomedical Libarian, University of Kansas Medical Center

Early Career Librarian Initiative Task Force, MCMLA Chapter

Background:
Committing time and space designated to support early career librarians’(ECL) growth in their new positions has been a forthcoming issue in health science librarianship. Recently, some MLA Chapters have recognized this and are filling the gap. The executive committee of this chapter recently established the Early Career Librarian Initiative (ECLI) Task Force after being inspired by other chapters’ success in providing similar support.

This poster describes the process of developing this space and resources, specifically for those who are ECL or new to health sciences. Both are designed to improve skills that are critical to their positions as well as learn the nuanced tips-and-tricks that come from years of experience and have been proven to be essential for success.

Description:
The ECLI began through a collaboration of like-minded chapter members, including a former chapter chair who recruited two ECLs to lead the initiative and strategize next steps. First, they researched effective practices from other chapters with ECL-specific support mechanisms, ECL’s needs, and presented a formal proposal requesting approval to form the ECLI task force from the chapter’s executive committee. Understanding ECL needs was conducted during an annual chapter meeting roundtable discussion, where

time and space was devoted to sharing ideas on how the chapter could better support ECLs. A mix of ECLs and seasoned librarians attended the roundtable, many who later joined the executive committee-approved task force.

To support the ECLI’s goal of supporting members new to or ECLs in health sciences, the task force decided the most effective place to start was to develop a resource guide and to establish a mentor and “buddy” relationship program. The task force met monthly as well as in separate groups to complete the work. Poster session attendees will have an opportunity to share tips-and-tricks they think ECLs would benefit from via a sticky-note placed on the poster during the conference, which will be shared at a future chapter executive committee meeting.

Program Conclusions:
The ECLI task-force assessment strategy will analyze the impact the resource guide and the building mentor and “buddy” relationships program has on chapter members. The results of the analysis of this data will inform the future direction of the initiative. It is expected that connecting and collaborating with other chapters with their ECL initiatives will result in closing more gaps that are present when a librarian is new to health sciences librarianship and more sharing of experiences with the health sciences library community.

Stronger Together: Using Library Electives to Fill Curriculum Gaps

Track(s): Education

Lisa Marks, Director, Library Services, Mayo Clinic in Arizona

Cynthia Chelf, Academic Medical Librarian, Mayo Clinic

Tara Brigham, Supervising Librarian in Florida, Mayo Clinic

Background:
At our medical school, library instruction is not fully integrated into the curriculum. There is some presence during the Evidence-Based Medicine (EBM) block towards the end of the 2nd year, however, it’s very minimal. Librarians within this institution have found a way around this issue by creating elective offerings (referred to as “selectives” within our medical school) for students to become familiar with library services and staff on their campus. During this poster presentation we will share how we are reaching the medical students at our institution.

Description:
Selectives are dedicated 1–4-week curriculum blocks that enable students to experience a self-directed approach to the medical curriculum, emphasizing flexibility and customization of the learning experience. Our selectives include: (1) The Undifferentiated Medical Student Podcast, (2) Medical Humanities Reading, (3) Searching the Medical Literature and (4) Health Literacy. Through these selectives, students can gain an understanding of medical specialties through listening to the podcast, gain a different perspective on the human side of medicine through reading, gain a better grasp of searching the medical literature via PubMed, and gain knowledge about the issue of health literacy. After completing the assignments, the selectives conclude with a one-on-one meeting with the librarian at their respective campus, either in-person or virtually. Before the 30-minute meeting, the librarian prepares by reading the assignment, typically a reflective essay. After the meeting, the student provides an evaluation form for the librarian to complete and sign, making the time commitment around 45 minutes to 1 hour per student.

All selectives can be completed remotely, making them popular choices during holiday seasons. Selectives can be completed throughout the 4 years of medical school.

Program Conclusions:
Although library instruction is not integrated into our medical school curriculum, we have found ways to engage with medical students via selectives. As students talk with their classmates about our offerings, our selectives have gained popularity by word of mouth. Students have provided positive feedback on each selective through their reflective essays and meetings. Both qualitative and quantitative data will be provided in the final presentation, including the current academic year.

Supporting Institutional Scholarship Through Journal Hosting

Track(s): Information Services

Heather Brown, Scholarly Communication Librarian | Head of Access Services, University of Nebraska Medical Center

Background:
In addition to providing a platform for scholarly work through an institutional repository, the library provides access to a journal publishing platform to potential university affiliated editors. Though the library does not participate in the publishing processes of the journal, it provides a suite of resources and services to editors. In this poster, attendees will learn the steps in creating an institution-based peer reviewed journal and providing manageable resources and services to journal editors.

Description:
With three peer reviewed journals in the library’s publishing portfolio, a process has been developed to help navigate the creation of a journal. The library is heavily involved with the development of the online platform, liaising with the vendor and graphic design support, providing resources for the journal editors such as ISSN registration and policy creation, and entering content within the online platform. Advising on licensing and copyright, training and support in using the platform, and providing DOIs for published articles are also part of the library’s responsibilities.

Program Conclusions:
The creation of a Memorandum of Understanding (MOU) is being developed to manage expectations for library resources and services in addition to outlining responsibilities of the journal editors.

Supporting Student Wellness Through a Library Social Work Practicum Placement Program

Track(s): Education

Beth Auten, Health & Human Services Librarian, University of North Carolina at Charlotte

Ryan Harris, Associate for Dean for Public Services, UNC Charlotte

Background:
The Health & Human Services (HHS) Librarian was approached by the director of Social Work Practicum Placements to facilitate a practicum placement at an academic library. Placements in community agencies and organizations are required for all senior social work students as part of their degree. Working with the Associate Dean for Public Services, the librarian developed an initial program for a year-long practicum placement. This included a description of proposed work activities a student could engage in at the library. This description was provided to all social work students. An interview was conducted with a student who was interested in the library practicum and was selected to participate in their practicum at the library.

Description:
In coordination with the Associate Dean for Public Services, the HHS Librarian developed initial onboarding for the practicum student including a tour of the library, meetings with key people and teams that they could collaborate with including Student Engagement, Accessibility, and DEI Committees, and shadowing at the Information & Research Desk. They identified potential projects that students could work on with an emphasis on student wellness and health. The librarian supervises practicum students' day-to-day work and they also have meetings throughout the semester with an external social work faculty member to evaluate their progress. Practicum students have worked on a variety of projects including: Creation of a student wellness resource guide; hosting drop-in hours where social work interns can guide students to library, campus, and community resources; and hosting pop-up events across campus to promote and demonstrate the library's self-care collection. When needed, students can refer their peers to specialized support services. Students have participated as a member of the library-wide Student Engagement committee which hosts various wellness events throughout the semester. One student helped develop a cozy game collection for the library's video game lab and planned an opening event promoting these games and how they can support student well-being.

Program Conclusions:
The library has hosted two social work practicum students in the Bachelor of Science in Social Work program during the 22-23 and 23-24 academic years. The first social work intern is now in a master's program for Social Work. We have applied to host a social work student again next year and plan for this to be a long-term ongoing project and would like to have a masters student participate. We will begin exit interviews with social work interns starting this semester to further refine and develop the program. We also plan to include current students conducting their practicum at the library in future interviews with practicum candidates so they can share their perspective about the program with interested students.

Systematic Review Service Support for an Institutional Data Retention Policy

Track(s): Information Management, Information Services

Drew Wright, Weill Cornell Medicine

Michelle Demetres, Evidence Synthesis Librarian, Weill Cornell Medicine

Background:
Weill Cornell Medicine recently updated their Research Data Retention Policy, defining the shared responsibilities of the University and researchers in collecting, retaining, securing, accessing, publishing, and sharing research data. To comply with this policy, all data used to generate publications by Weill Cornell Medicine authors must be deposited into the 'Institutional Data Repository for Research' (WIDRR). WIDRR is a dark archive, not an open data repository, meant to preserve research data. To support this initiative, the library's Systematic Review (SR) Service has developed its service offerings to include post-publication data deposition into WIDRR.

Description:
For all formal systematic/scoping review collaborations where a librarian is listed as a co-author, the collaborating librarian will deposit all necessary data into WIDRR after publication on behalf of the SR research team, unless the team prefers to do so.. The data in WIDRR should be sufficient for replication and audit of the research. Using best practice reporting standards as guides (National Academies' "Finding What Works in Healthcare" TABLE G-1; Appendix G "Comparison of Chapter 5 Guidance on Conducting Systematic Reviews of Comparative Effectiveness Research") we determined that the following data will be collected from the collaborating librarian and from the SR research team. Data from the librarians/exported

from Covidence: (1) search strategies (.txt/.pdf); (2) database export files (.ris); (3) irrelevant studies (.csv); (4) excluded studies (.csv); (5) included studies (.csv); (6) title/abstract Inter-Rater Reliability (.csv); (7) full-text Inter-Rater Reliability (.csv). From the SR research team: (1) data extraction tool (.txt/.pdf); (2) data extraction results (.csv/...); (3) data analysis methods– i.e., meta-analysis/statistics/funnel plot (.txt/...); (4) data analysis results – i.e., meta-analysis/statistics/funnel plot(.csv/...); (5) risk of bias tool (if new/adapted) (.txt/.pdf); (6) risk of bias results (.csv); (7) data extraction tool (.txt/.pdf).

Program Conclusions:
As librarians with multiple roles and expertise, we are positioned to be effective stewards for responsible data retention practices through our systematic review work. With this new policy in place for our systematic review service, we hope to not only be champions for the institutional data retention mandate, but also educators on the need for and practice of data retention. When this addition to our service is more established, we aim to report on its compliance rate.

Systematic Review(ish) Assignments, a Faculty Workshop: From Learning Objective to Successful Classroom

Track(s): Education, Information Services

Anna Ferri, Evidence Synthesis Librarian, Colorado State University

Jenn Monnin, Research Support and Engagement Librarian, West Virginia University Libraries

Background:
Faculty often use research projects as classroom assignments to build students’ skills as researchers. This is a challenging practice for systematic reviews that often results in substantial time, effort, and support from librarians beyond the classroom. We sought to answer how students can gain classroom experience in evidence synthesis without compromising research integrity by developing a workshop for faculty. Our goals are to encourage faculty to recognize the limitations and challenges of review production in the classroom and to identify scaffolded, ‘systematic reviewish’ assignments to mitigate challenges while meeting learning outcomes. Additional purposes are to proactively improve the learning experience for students and encourage more accurate recognition of the knowledge, skills, and abilities necessary for evidence synthesis

Description:
A workshop aimed at instructional faculty was developed with three learning objectives for attendees: 1) recognize the essential components and processes of a systematic review and the challenges these create in the classroom environment, 2) unpack and prioritize the student learning objectives that are being addressed via a systematic review assignment, and 3) select between various alternative assignments to meet said learning objectives within the classroom without compromising the quality of the process necessary to produce a systematic review. The workshop design incorporates discussion prompts and formative assessment to both enhance reflective learning and to encourage greater dialogue between instructional faculty and librarians. This workshop will be offered at two R1 universities in March 2024, one with a medical school and one with a school of veterinary medicine. An accompanying survey will ask attendees to assess the workshop design and the appropriateness of the seven potential learning goals presented and the alternative assignment options provided. This feedback will be presented in the final poster, as well as being used to update workshop materials and enhance a toolkit hosted in Open Science Framework (OSF) for other librarians to adapt and use at their institutions.

Program Conclusions:
This workshop was designed to be flexible and shared under a CC-BY license so that other librarians can adapt and use all workshop and toolkit materials. The OSF project includes the workshop slides, activities and assessments, sharable assignment templates, and a wiki that can be referenced for further information

or shared directly with faculty attendees. Next steps include updating materials based on the solicited feedback from the first two workshops, and then advertising the OSF materials, including the toolkit, to librarians who collaborate on systematic reviews and health science instruction. We intend to gather feedback from librarians who use the materials and/or teach the workshop at their institutions to further improve the content for all.

Teaching Telehealth to Libraries

Track(s): Information Services

Faith Steele, NNLM Region 1 Executive Director, University of Maryland, Baltimore

Darlene Kaskie, Associate Director, Region 6, Network of the National Library of Medicine

George Strawley, Engagement Specialist, NNLM Region 4

Tiffany Chavis, Outreach and Education Librarian, Network of the National Library of Medicine, Region One / University of Maryland, Baltimore

Katie Pierce Farrier, Data Science Strategist, Network of the National Library of Medicine, Region 3

Background:
The pandemic disrupted society as we knew it. Isolating for extended periods, people relied on technology to shop, work, and schedule medical appointments. Yet, one-fifth of the nation’s families do not subscribe to broadband. Recognizing the inequity, libraries serve in bridging the divide, providing free digital access and services to their under-resourced communities, including telehealth access. This poster will discuss the project conception, design and evaluation. Viewers can expect to: Identify and advocate to stakeholders a role that libraries can play in addressing health inequities through telehealth access. Discuss how libraries incorporated digital navigators and community health workers to support telehealth programming. Interpret results of two library telehealth training programs to justify telehealth access service.

Description:
A Telehealth Interest Group was formed from multi-institutional health sciences libraries to develop educational resources and trainings to support public library staff. Telehealth 101: What Libraries Need to Know is an asynchronous online course introducing infrastructure and technology, privacy and ethical considerations, and health information resources for telehealth. Additionally, the group created a webinar series, Bridging the Digital Divide, that featured expert speakers discussing topics and trends related to telehealth such as telehealth consortiums, social workers in libraries, and programming for rural libraries.

Program Conclusions:
In 2023 the Telehealth group launched the Telehealth 101 course and Bridging the Digital Divide webinar series. Two sessions of Telehealth 101 were offered with a total of 150 enrollees. Two webinars for the Bridging the Digital Divide were offered with a total 394 registrants. Based on continued interest, enrollment and evaluation feedback, the Telehealth Interest Group plans to continue and expand offerings in 2024.

Teaching the Art of Reading Research Articles: A Fruitful Pilot Project

Track(s): Education

Christine Andresen, Associate Professor, Medical University of South Carolina

Background:
In academic and clinical settings, nurses must be knowledgeable about the basics of the research process, including the ability to critically appraise research and determine the applicability of research findings. Entry-level nurses are expected to be able to participate in scholarly inquiry, evaluate research, and determine strength of evidence, which aligns with course outcomes in the Accelerated Bachelor of Science in Nursing (ABSN) program. As part of the curriculum, the liaison librarian leads class sessions on finding and reading scholarly articles, while introducing learners to the concept of critiquing research, and excitedly jumped at the opportunity to incorporate a new active learning element by participating in the FeedbackFruits pilot during the Spring 2024 semester.

Description:
The librarian envisions FeedbackFruits 'Comprehension' tool functionality to be an ideal solution to guiding learners through the process of reading and critiquing research articles using a form of social annotation to enhance critical reading skills. Nursing course faculty were consulted to advise on identifying appropriate research articles for the exercise, and the librarian created question cards and discussion threads within the articles using the guided comprehension tool to prompt students to interact and comment on specific parts of the article within the LMS. Using the Feedback Fruits interface, annotation cards are used to gauge student understanding by multiple choice or open answer content, including prompts for learners to summarize a particular section of the article. The librarian is able to customize the activity, ensuring that learner comments and questions in their annotations are visible to peers during the in-class practice activity so that they benefit from seeing each other's different interpretations and encourage engaging in-class discussion, while having the flexibility to make those annotations anonymous during the homework exercise to gauge individual student comprehension.

Program Conclusions:
Outcomes from this pilot project will include a student experience/satisfaction questionnaire, educator summaries of learner comprehension successes along with opportunities for improvement, and will highlight next steps for phase two of the pilot project.

Unboxing the Potential of Instagram: A Graphic Medicine Collection Development Collaboration

Track(s): Professionalism & Leadership

Tiffany Follin, Medical Liaison & Outreach Librarian, Florida Atlantic University

Agrippina Fadel, Marketing and Communications Specialist, Florida Atlantic University

Dawn Frood, Discovery Services Librarian, Florida Atlantic University

Background:
At the start of this initiative, the Libraries' External Relations department already had an established Instagram account that was actively engaging with the university community. Using Instagram allowed a creative, collaborative approach that primarily targeted the 18–34-year-old demographic which comprised roughly 62.9% of the Libraries account. Using humor, superhero costumes, trending music, and strategic soundbites, each resulting reel emphasizes the comic collection, the addition of new material to the graphic novel sub-collection, humanizes the librarians as well as the Libraries, and most importantly: does it by reimagining the library book "unboxing" experience using creative storytelling.

Description:
Since December 2021, the Medical Librarian has steadily built a collection of graphic medicine novels with a small annual fund. "Graphic Medicine is a field that explores and supports the interaction between the

medium of comics and the discourse of healthcare" (Graphicmedicine.org, n.d.). Novels further filled the unique intersectional niche of “quick reads,” “educational,” and “patient/family perspective” that offered a low participation threshold for busy healthcare practitioners and students to engage with.

In July 2023, a selection of seven newly arrived print titles were chosen to incorporate into the first reel utilizing a booth transformation of librarians into superheroes, with a subsequent series of scenes in various library locations. The film session evolved from a loose plan pre-filming to include spontaneous ideas to improve the end content. Additional reels were created based on evaluating the end result of the first reel. The librarians realized the length of reels needed to be shortened and there were missing key elements—students and the community! Reel two engaged students while in an elevator, while reel three leveraged a partnership with a local comic books author. One additional student worker created reel supplemented content to give a unique student perspective to the collection.

Program Conclusions:
Best Practices of creating content were employed to maximize Instagram algorithms and immediate success of reels is loosely measured by views, commenting, and likes. As the graphic medicine collection highlights diverse personal stories within the topics of illness and health, these novels can be incorporated into course materials, book clubs, reader advisories, and life-long learning. Long term success will measure how these titles are utilized and will involve leveraging additional partnerships, such as the College of Medicine's social media accounts, to promote material. In November 2023, the libraries’ debuted its first meeting of a new interprofessional healthcare book club, which is utilizing curated and promoted content to meet required interprofessional core competencies within the health sciences field.

Unconference Experiences:Two Case Studies of Planning and Execution of Non-traditional Virtual and Physical Events with Resources to Organize Your Own

Track(s): Innovation & Research Practice, Professionalism & Leadership

Daniel Verbit

Tony Nguyen, AUL Rutgers Biomedical and Health Sciences Libraries, Rutgers University

Background:
Unconferences are participant-driven events where those attending select topics and guide discussions instead of the pre-planned agenda by an organizer. The emphasis is on interaction between attendees. In this model the participants are given a space to engage on topics which interest them .

Key unconference goals are are to provide a space to:

- Discover new ways of working
- Develop new networks of colleagues
- Practice new learnings
- Deepen practice
- Discuss late breaking advances in the field

In the past, unconferences were organized as in-person events. However, due to the pandemic and geographically dispersed stakeholders, virtual unconferences serve as a unique and transformative gathering, bringing together like-minded people around large-scale conversations that matter to support the emergence of social innovation and transformation.

Description:

Scholarly and gray literature searches were conducted to understand library and organizational experiences in hosting unconferences and identify best practices in facilitating both in-person and virtual unconferences.

Based on an understanding of best practices and previous participation in unconferences, a author planned a half day in-person unconference to engage with association members in a meaningful way on a small scale without a complex prior planning process.

Another organization with geographically dispersed stakeholders, sought to implement technology and adapt best practices to host a virtual symposium over the course of three days each lasting approximately 1.5 hours each day. Following the virtual sessions, facilitation leads offered opinions and insights to streamline the process and increase engagement of participants. Evaluations were sent to participants with two reminders prior to the close of the survey to gauge participant insights should they be offered in the future.

This poster will provide citations and resources for those inspired to plan their own. In our initial research the last time an unconference case study was presented was in 2013.

Program Conclusions:
The in-person unconference was filled to capacity and feedback during and following was overly positive that it was a beneficial experience and worth the time and travel.

Evaluation of the virtual unconference is in-process. However, if accepted, the poster would recommend best practice considerations should other organizations choose to offer an unconference and engage with stakeholders.

USF Health Library Book Club: Creating Connections

Track(s): Information Services

Janet Chan, Research and Education Librarian, University of South Florida

Allison Howard, Research and Education Librarian, USF

Ardis Hanson, Assistant Director, Research & Education, USF

Krystal Bullers, Research & Education Librarian, USF Health Libraries, University of South Florida

Stephanie Tomlinson, Research & Education Librarian, College of Medicine Liaison, USF

Tsu Yin Lue, Research & Education Staff Assistant, USF

Background:
This poster examines the development and adaptations of a book club in an academic health sciences library. The project’s goal is to expand the librarians’ engagement with patrons beyond teaching and learning activities delivered through classes and research consultations. The book club allows interactions with health patrons on a more informal basis and fosters connections with a broader audience within the university community. An additional objective of the book club is to promote interprofessional dialogue between members of our health colleges and programs.

Description:
The book club was initially developed by the librarians over two planning meetings. The planning meetings focused on determining the format (online or in-person), frequency, number of participants who could be accommodated, advertising, registration, availability of book selection, conduct expectations, and timing of the event. The decision was made to conduct a single session to discuss the book, in an online format for 1 hour. The initial session was held at 5 pm with a book selected by the librarians. This first event was a

success with 8 participants representing five programs, who were all engaged in the conversation. Based on this success, the librarians chose to hold one book club meeting per semester.

After each session, a survey is sent to the participants. Based on the survey results, event times, days, and locations have been adjusted for subsequent book clubs with varying success. The librarians curate a short list of health-themed books, then selections are voted on via Instagram and an emailed survey. Since the inaugural book club in February 2023, three sessions have been held, with the next planned for spring 2024.

Program Conclusions:
The book club is a fun and engaging way to bring together the various colleges and programs that the library serves in a relaxed atmosphere. Quantitative and qualitative data from our surveys have confirmed that the book club is a valued offering that our patrons enjoy. Demographic data reveals participants represent multiple colleges and programs we serve as well as the broader university community, contributing to the promotion of interprofessional engagement. Participants enjoyed meeting new people and the discussion of relevant topics including social injustice and life questions. Perhaps the best outcome of the book club was this comment from a participant: “I enjoyed that it was a safe space with questions provided to encourage further discussion about the book.”

Unlocking Classroom Innovation: Teaching, Cultivating Critical Thinking and Fostering Lifelong Learning Skills

Track(s): Education, Innovation & Research Practice

Leslie Golamb, Liaison Librarian, A.T. Still University

Maud Mundava, Campus Head/Assistant University Library Director, A.T. Still University

Background:
Innovation in the classroom is all about thinking outside the box and how to effectively engage and foster evidence-based information skills. Additionally, it is a great way of promoting critical thinking and lifelong learning skills among students. This can be done by introducing new teaching strategies and methods into the classroom. This poster will:

* Provide ideas and tools that can be used to create an inclusive, engaging, and learner-centered learning environment for diverse learners by encompassing a variety of different instructional strategies including the ADDIE model.

* Demonstrate how to apply your innovation using the ACRL framework.

* Demonstrate how to apply student's learning outcomes scaled to Bloom's Taxonomy.

* Describe best practices for collaborating with Instructional Designers.

Description:
The A.T. Still Memorial Library liaison program facilitates partnerships and communication between the library and ATSU academic programs. The role of a liaison is to foster ways to embed library resources and services within the curriculum/classroom and provide information literacy instruction.

This poster will describe how a library liaison successfully partnered with teaching faculty and Instructional designers to plan and implement an interactive 2-day curriculum to teach APA formatting using various technologies and strategies:

* Technology like LibWizard, Asana, and Canvas

* Learning Strategies: Escape Room and Jeopardy Game

* The ACRL framework

* The ADDIE model

* Bloom’s Taxonomy

These tools helped to build a curriculum that is innovative, engaging, and enabling self-reflection. It also enabled students to practice skills through asynchronous learning. Some examples of assignments, and quizzes that were used in the course were an escape room; each week, the assignments built upon themselves to result in a final paper. Each assignment was provided with meaningful feedback. In the last class we did a review of APA format using a Jeopardy game.

Program Conclusions:
After the 4 weeks were over, a survey (using Qualtrics) was sent out to all students to provide feedback. As a new library liaison for this college:

* It enhanced collaboration between the library and the academic community.

* Provided ongoing dialogue, connections, and communication between the library and faculty increasing awareness of library resources and services.

* Strengthened and promoted student’s critical thinking and lifelong learning skills by integrating evidence-based instructional activities into the classroom.

This innovation also resulted in being nominated for the Teaching and Learning Award. Since then, other colleges within the University have asked for the content so they can embed it into their courses as well.

The Use of Microlearning in Health Science Education

Track(s): Education

Carrie Adams, Program Director, University of Florida

Background:
Microlearning has been utilized in health sciences professional education for a number of years, providing benefits related to convenience, access, and availability at the point-of-need along with effectiveness and learner retention. However, it has been utilized less frequently as an instructional method in health sciences libraries.

Description:
This poster will provide a review of the current and past literature related to microlearning use as an instructional strategy in health sciences, including the modalities, best practices, pitfalls, and evidence for effectiveness, learning outcomes, learner satisfaction, self-efficacy, competence, and confidence. This will include a discussion of the possible opportunities for transitioning a microlearning strategy from the professional education realm to libraries.

Program Conclusions:
Identifying successful instructional strategies in other related areas may provide guidance for innovative educational opportunities in health sciences libraries. As there is a significant overlap in populations between health sciences professional education and health sciences library users, microlearning may be applicable for librarians seeking to expand their educational offerings.

Using Google Analytics Data API to present “Most Visited” Resources to Library Website Visitors

Track(s): Information Services

Aaron Pinero, Web Services Manager, Columbia University Health Sciences Library

Background:
Our current website was launched in July 2020. Unlike the previous version of the website, the home page did not present a list of “Quick Links” to selected resources. The rationale behind this decision was to avoid biasing website visitor behavior by highlighting resources selected by library staff, or by making unsupported assumptions about visitors’ preferred resources. However, the library received some negative feedback from website visitors regarding the new website, specifically the lack of any “Quick Links” on the website home page.

Description:
We use Google Analytics to examine how the library website is used. By analyzing outbound link data over the first year following the launch of the new website, we could create a data-supported list of “Quick Links” to present on the website home page. However, that list would reflect website use only at a specific point in time. To generate a dynamic list that is always based on current use trends, we used the Google Analytics Data API (formerly the Google Analytics Reporting API) to regularly pull outbound links reports from Google into the website and generate a list of “Most Visited” resources to display on the website home page.

Program Conclusions:
In the year following the introduction of the Analytics-based “Most Visited” resources (February 2022 – January 2023) there was a decrease in the Pages per Session and Sessions with Search metrics recorded by Google Analytics as compared with the previous year. Examining the data more closely, there was a decrease in searches where the keyword was the name of a “Most Visited” resource. There was also a decrease in page views in the Resources section of the website. These changes suggest that website visitors were making use of the Most Visited resources instead of navigating the website to find the resource links or using the website search to locate those resources.

Using Microsoft Teams to Manage Librarian Workflows for a Busy Evidence Synthesis Service

Track(s): Information Management

Abigail Smith, Assistant Director, Research & Instruction, Upstate Medical University

Rebecca Kindon, Director of Libraries, SUNY Upstate Medical University

Background:
Librarians at our university/academic medical center have seen a dramatic increase in requests for

collaboration on evidence synthesis projects over the past five years. This trend is unsurprising as there has been an acceleration in systematic review publications worldwide. In a retrospective observational study, Hoffman et al. (2021) demonstrated a 20-fold increase in the number of systematic reviews indexed over the last 20 years, which in 2019 is equivalent to 80 new systematic reviews published per day. Faced with more requests than they could manage, the library implemented a waitlist and realized they needed a cost-neutral, way to track librarian workflows throughout the review process to ensure that all requests were accounted for and being attended to.

Description:
Microsoft Teams, a communication platform licensed by our university, was integrated into our library's workflow in 2020 and facilitates collaboration in our hybrid work environment. Each library department has a channel where faculty and staff can discuss topics specific to their area, store files, and access a planner for project management. The library decided to trial the use of the planner in teams to determine whether it could efficiently manage librarian workflows for systematic, scoping, and other review types,

Our evidence synthesis planner allows for progress tracking and task assignment. There are nine categories called "buckets", which are labeled: Waitlist, Consultation, Strategy, Mapping, De-Duplication/Record Management, Inactive with library, Search Update, Manuscript Preparation/Editing, and Publication. These buckets represent the various stages that evidence synthesis projects go through in the library. Each review is entered as a task and assigned a librarian. The assigned librarian then moves the review (task) through each bucket as they progress through the project. This visual representation of our progress eases the reporting out of evidence synthesis workloads, aids in the assignment of new review projects, and signals when it makes sense to take another patron off the waitlist.

Program Conclusions:
The planner in Microsoft Teams has been an effective way to track progress and workflows for our evidence synthesis service, at no added cost to the library. Feedback from library faculty has been mixed, while all users appreciate the organization that the planner has facilitated, some users find it to be yet another "thing to do" in a review process that is already arduous. To mitigate those concerns the planner will be updated by the coordinator of the evidence synthesis service when projects are reviewed at their monthly team meeting.

Value of Advisory Groups

Track(s): Professionalism & Leadership

Linda Walton, Director, NNLM Region 6 University of Iowa

Bobbi Newman, Community Engagement and Outreach Specialist, Network of the National Library of

Darlene Kaskie, Associate Director, Region 6, Network of the National Library of Medicine

Erica Lake, Health Sciences Librarians Outreach Specialist, Network of the National Library of Medicine, Region 6

Jacqueline Leskovec, Outreach Specialist, NNLM Region 6

Miles Dietz-Castel, Communications Specialist, NNLM Region 6

Nora Barnett, Outreach Specialist, NNLM Region 6

Background:
As a regional program there has traditionally been a Regional Advisory Group with limited impact. The primary aim of this study was to determine if State Advisory Groups (SAG) would improve our impact by focusing on locally determined health information needs for the states in our region.

Description:
Invitations to take part in a State Advisory Group were sent with the intent of having a variety of representatives interested in improving health information, including public librarians, state librarians, health sciences librarians, public health professionals, health professionals, faith-based organizations, and community organizations. Those interested applied to be on the SAG with each state having varied representation. Once each SAG was set up, a chair was identified with the staff serving as support. Each of the seven SAG group members spent a year discovering their state's health priorities and developing action plans. This Spring the SAG members will be surveyed to see if they believe the implementation of SAGs provided impact within their communities.

Program Conclusions:
Mental health was identified as a critical health issue in three states, followed by substance abuse, period poverty, health information through technology for elders, and dental health for children. The action plans vary for each SAG. Some projects will develop a model to be implemented in other communities, others are creating resources that will be distributed through libraries, and others are providing programming in various areas of the state. While it’s challenging keeping seven advisory groups engaged throughout the process, we believe we have seen more buy-in from communities, more awareness of our program, we have valuable health information programs throughout the region. The survey will provide us with feedback from our SAG participants to see if they agree with the outcomes.

Working Together to Mint DOIs on Demand for a DSpace Repository

Track(s): Information Management

Lisa Palmer, Repository Librarian, University of Massachusetts Chan Medical School

Tess Grynoch, Research Data & Scholarly Communications Librarian, University of Massachusetts Chan Medical School

Background:
Digital Object Identifiers (DOIs) are a key persistent identifier in the publishing landscape to ensure discoverability and citation of research products. Minting DOIs can be a time-consuming task for repository librarians. This process can be automated since the metadata for DOIs is already in the repository record and DataCite, a DOI minting organization, and Open Repository, a DSpace repository platform, both have application programming interfaces (APIs). Previous software has enabled bulk DOI minting. However, the institutional repository contains a mixture of original materials (dissertations, reports, data, etc.) and previously published materials such as journal articles and preprints.

Description:
An institutional repository librarian and her librarian colleague with Python experience embarked on a pair programming project to create a script to mint DOIs on demand in DataCite for individual items in the institution’s Open Repository instance. The pair met for one hour each week to develop and test the script. The institutional repository librarian lent invaluable insight into both platforms and the metadata variations the code would need to account for. The project was also a great learning opportunity for both librarians to improve their Python coding skills. This project will be evaluated in terms of how the time spent creating the code compares to the time it takes to mint DOIs manually as well as metadata enhancements and accuracy in DataCite.

Program Conclusions:
This poster will share the final Python script, resources used, and highlight the takeaways from this approach for both the institutional repository librarian and the coding librarian. Final evaluation is forthcoming.

You’ve Got Mail: Designing Email Courses for Medical Library Users

Track(s): Education, Information Services, Innovation & Research Practice

Kaitlin Throgmorton, Data Librarian for the Health Sciences, Yale University

Dana Haugh, Web Services Librarian and Coordinator for Marketing & Communications, Yale University

Background:
This program's main objective was to transform synchronous instruction into asynchronous, on-demand content that could be time-released in segments. We did this by using email messaging software to develop two library email courses on two different topics.

Description:
In an increasingly fast-paced context, some users seek new ways to digest large amounts of information. Email courses enable asynchronous, on-demand educational content in a structured and paced format, providing cohesive training to a wide audience with minimal staff time commitment. To explore the practical benefits and to expand user understanding of two broad topics, we developed two email courses within our academic health sciences library: how to write data management plans and how to design effective posters. Our poster will demonstrate the process of planning and developing email courses, outline each course’s structure and delivery timeline, and showcase statistics on subscription trends and assessment. We will also discuss successes and challenges with this format and explain how other libraries could create their own email courses.

Program Conclusions:
For outcomes, we will be reporting on number of modules and participants for each email course, as well as other potential metrics of interest available in the email messaging software. We will also report qualitative comments from email course participants as recorded in our general instructional assessment survey.
